# Riociguat in patients with early diffuse cutaneous systemic sclerosis (RISE-SSc): randomised, double-blind, placebo-controlled multicentre trial

**DOI:** 10.1136/annrheumdis-2019-216823

**Published:** 2020-04-15

**Authors:** Dinesh Khanna, Yannick Allanore, Christopher P Denton, Masataka Kuwana, Marco Matucci-Cerinic, Janet E Pope, Tatsuya Atsumi, Radim Bečvář, László Czirják, Eric Hachulla, Tomonori Ishii, Osamu Ishikawa, Sindhu R Johnson, Ellen De Langhe, Chiara Stagnaro, Valeria Riccieri, Elena Schiopu, Richard M Silver, Vanessa Smith, Virginia Steen, Wendy Stevens, Gabriella Szücs, Marie-Elise Truchetet, Melanie Wosnitza, Kaisa Laapas, Janethe de Oliveira Pena, Zhen Yao, Frank Kramer, Oliver Distler

**Affiliations:** 1 Division of Rheumatology, University of Michigan, Ann Arbor, Michigan, USA; 2 Rheumatology A department, Cochin Hospital, APHP, Paris Descartes University, Paris, France; 3 Division of Medicine, Centre for Rheumatology, University College London, London, UK; 4 Department of Allergy and Rheumatology, Nippon Medical School Graduate School of Medicine, Tokyo, Japan; 5 Department of Experimental and Clinical Medicine, University of Florence, Firenze, Italy; 6 Schulich School of Medicine, Division of Rheumatology, The University of Western Ontario, London, Ontario, Canada; 7 Department of Rheumatology, Endocrinology and Nephrology, Faculty of Medicine and Graduate School of Medicine, Hokkaido University, Sapporo, Japan; 8 Institute of Rheumatology, Department of Rheumatology, 1st Faculty of Medicine, Charles University, Prague, Czech Republic; 9 Department of Rheumatology and Immunology, University of Pécs, Pécs, Hungary; 10 Department of Internal Medicine and Clinical Immunology, Claude Huriez Hospital, Lille University School of Medicine, Lille, France; 11 Clinical Research, Innovation and Education Center, Tohoku University Hospital, Sendai, Japan; 12 Department of Dermatology, Gunma University Postgraduate School of Medicine, Maebashi, Japan; 13 Division of Rheumatology, Department of Medicine, Toronto Western Hospital, University Health Network, Mount Sinai Hospital, University of Toronto, Toronto Scleroderma Research Program, Toronto, Ontario, Canada; 14 Laboratory of Tissue Homeostasis and Disease, Skeletal Biology and Engineering Research Center, Department of Development and Regeneration, KU Leuven, Leuven, Belgium; 15 Rheumatology Unit, Department of Clinical and Experimental Medicine, University of Pisa, Pisa, Italy; 16 Department of Clinical Medicine and Therapy, University of Rome La Sapienza, Rome, Italy; 17 Division of Rheumatology, Department of Internal Medicine, Michigan Medicine University Hospitals, Ann Arbor, Michigan, USA; 18 Division of Rheumatology and Immunology, Medical University of South Carolina, Charleston, South Carolina, USA; 19 Department of Rheumatology and Internal Medicine, Ghent University Hospital, Ghent, Belgium; 20 Division of Rheumatology, Georgetown University Medical Center, Washington, DC, USA; 21 Department of Rheumatology, St. Vincent's Hospital Melbourne, Melbourne, Victoria, Australia; 22 Division of Rheumatology, Department of Internal Medicine, University of Debrecen, Debrecen, Hungary; 23 Department of Rheumatology, CHU Bordeaux, Bordeaux, France; 24 Research & Development, Bayer AG, Wuppertal, Germany; 25 StatFinn Oy, Espoo, Finland; 26 Bayer HealthCare Pharmaceuticals Inc, Whippany, New Jersey, USA; 27 Bayer Healthcare, Beijing, China; 28 Department of Rheumatology, University Hospital, Zurich, Switzerland

**Keywords:** systemic sclerosis, treatment, disease activity

## Abstract

**Objectives:**

Riociguat is approved for pulmonary arterial hypertension and has antiproliferative, anti-inflammatory and antifibrotic effects in animal models of tissue fibrosis. We evaluated the efficacy and safety of riociguat in patients with early diffuse cutaneous systemic sclerosis (dcSSc) at high risk of skin fibrosis progression.

**Methods:**

In this randomised, double-blind, placebo-controlled, phase IIb trial, adults with dcSSc of <18 months' duration and a modified Rodnan skin score (mRSS) 10–22 units received riociguat 0.5 mg to 2.5 mg orally three times daily (n=60) or placebo (n=61). The primary endpoint was change in mRSS from baseline to week 52.

**Results:**

At week 52, change from baseline in mRSS units was –2.09±5.66 (n=57) with riociguat and –0.77±8.24 (n=52) with placebo (difference of least squares means –2.34 (95% CI –4.99 to 0.30; p=0.08)). In patients with interstitial lung disease, forced vital capacity declined by 2.7% with riociguat and 7.6% with placebo. At week 14, average Raynaud’s condition score had improved ≥50% in 19 (41.3%)/46 patients with riociguat and 13 (26.0%)/50 patients with placebo. Safety assessments showed no new signals with riociguat and no treatment-related deaths.

**Conclusions:**

Riociguat did not significantly benefit mRSS versus placebo at the predefined p<0.05. Secondary and exploratory analyses showed potential efficacy signals that should be tested in further trials. Riociguat was well tolerated.

**Key messages**

**What is already known about this subject?**

- There is a need for new therapies for patients with diffuse cutaneous systemic sclerosis (dcSSc).
- The soluble guanylate cyclase stimulator riociguat has antiproliferative, anti-inflammatory and antifibrotic effects in vitro and in animal models of tissue fibrosis and has been shown to increase digital blood flow in patients with Raynaud’s phenomenon.

**What does this study add?**

- The RIociguat Safety and Efficacy in patients with diffuse cutaneous **S**ystemic **Sc**lerosis study failed to meet its primary endpoint of change in modified Rodnan skin score after 52 weeks at p=0.08. However, some secondary and exploratory endpoints showed potential efficacy signals that should be investigated in further trials. Riociguat was well tolerated, with no unexpected safety signals.

**How might this impact on clinical practice or future developments?**

- Although the primary endpoint was not significant, this phase IIb study provides important information that can inform future study design and gave preliminary findings that could be explored in future trials in patients with dcSSc.

## Introduction

Systemic sclerosis (SSc) is an autoimmune connective tissue disease characterised by fibrosis, inflammation and microvascular injury.^1–3^ Systemic organ manifestations include pulmonary arterial hypertension (PAH), interstitial lung disease (ILD), Raynaud’s phenomenon (RP) and digital ulcers (DU).^3 4^ To date, nintedanib is the only approved therapy for the treatment of SSc-ILD.^5 6^ Thus there is a significant unmet need, particularly in diffuse cutaneous SSc (dcSSc).^3^

The soluble guanylate cyclase (sGC) stimulator riociguat increases intracellular cyclic guanosine monophosphate (cGMP).^7^ cGMP activates protein kinases G, which are important in the regulation of vascular tone and remodelling.^8^ Riociguat was approved for treatment of PAH following the phase III Pulmonary Arterial Hypertension Soluble Guanylate Cyclase-Stimulator Trial 1 (PATENT-1) study, which included a subgroup with PAH-SSc, in which riociguat prevented the decline in 6 min walking distance seen with placebo.^9^ In a single-dose pilot study, riociguat increased digital blood flow in patients with RP.^10^ Riociguat has demonstrated antiproliferative, anti-inflammatory and antifibrotic effects mediated by attenuation of transforming growth factor beta-1 signalling in animal models and in vitro studies.^7 8 11–14^ sGC stimulators prevented and treated fibrosis in models of SSc.^12 15^

We hypothesised that riociguat may benefit tissue fibrosis in dcSSc. The RIociguat Safety and Efficacy in patients with diffuse cutaneous **S**ystemic **Sc**lerosis (RISE-SSc) trial compared riociguat with placebo in patients with early dcSSc.^16–18^

## Methods

### Design overview

RISE-SSc (clinicaltrials.gov identifier: NCT02283762^19^) was a randomised, double-blind, placebo-controlled, parallel-group, phase IIb, international, multicentre study, consisting of a screening phase (≤2 weeks), a 52-week, double-blind, main treatment phase and a long-term extension (see online supplementary figure S1 and supplementary file 2). All patients provided written informed consent. Each site’s institutional review board or ethics committee approved the protocol. The study was performed in accordance with the Declaration of Helsinki and Good Clinical Practice.

10.1136/annrheumdis-2019-216823.supp1 Supplementary data

10.1136/annrheumdis-2019-216823.supp2 Supplementary data

### Study participants

Investigators enrolled patients ≥18 years old, fulfilling American College of Rheumatology/European League Against Rheumatism (ACR/EULAR) classification criteria for SSc,^20^ with dcSSc according to LeRoy and Medsger.^21^ Based on European Scleroderma Trials and Research Group (EUSTAR) cohort observations,^16–18^ entry criteria specified disease duration ≤18 months (defined as time from first non-RP manifestation) and modified Rodnan skin score (mRSS) 10–22 units to enrich the study with patients at risk of skin fibrosis progression. Other inclusion criteria were per cent predicted forced vital capacity (FVC%) ≥45% and haemoglobin-corrected per cent predicted diffusing capacity of the lung for carbon monoxide (DL_CO_) ≥40% at screening. Patients receiving concomitant nitrates, nitric oxide donors, phosphodiesterase inhibitors or recent SSc therapies were excluded (see online supplementary file 1, p1–3).

### Randomisation and intervention

Patients were randomised 1:1 to riociguat or matching placebo, individually adjusted every 2 weeks from 0.5 mg to 2.5 mg orally three times daily over 10 weeks and continued throughout the treatment phase. From week 26, rescue therapy was permitted at investigator discretion (see online supplementary file 1, p4). Physical examination, disease status and demographics were obtained at day 0. Disease status was re-evaluated at weeks 12, 26 and 52, with additional assessments of mRSS and pulmonary function at week 39. Raynaud’s condition score was assessed by a patient diary completed daily for seven consecutive days before the first treatment dose and at week 14. Safety assessments included laboratory assessments at screening, on day 0, and at weeks 2, 4, 6, 8, 10, 26, 39 and 52, and evaluation of vital signs, adverse events (AEs) and serious adverse events (SAEs) coded by Medical Directory for Regulatory Activities preferred terms, DU net burden and prior and concomitant therapy at every visit.

### Outcomes and follow-up

The primary endpoint was the change in mRSS from baseline to week 52. To prevent interobserver variability, the same physician, experienced in skin scoring, scored the same patient throughout the study. Skin fibrosis was also analysed by prespecified exploratory analyses of mRSS progression (increase by >5 units and ≥25% from baseline) and regression (decrease by >5 units and ≥25% from baseline). This definition was based on analyses suggesting that a reduction in mRSS of 3.2–5.3 units or 15%–25% from baseline is considered a minimally clinically important difference.^22 23^ In addition, descriptive analysis in prespecified patient subgroups was performed (see online supplementary file 1, p4). Secondary endpoints were tested hierarchically in the order: American College of Rheumatology Composite Response Index for Systemic Sclerosis (ACR CRISS) at week 52^24^ (see online supplementary file 1, p5–6), Health Assessment Questionnaire Disability Index, patient’s global assessment, physician’s global assessment and change in FVC%. An independent, blinded Adjudication Committee reviewed clinical outcomes potentially representing systemic organ manifestations of dcSSc (see online supplementary file 1, p6), and all causes of death.

FVC% and DL_CO_% were assessed overall and (post hoc) in patients with ILD according to medical history and restrictive lung disease (FVC% 50%–75% at baseline).

Effects on RP at week 14 versus day 0 and net digital ulcer burden were prespecified exploratory analyses. For details of other prespecified exploratory analyses and post hoc assessments see online supplementary file 1, p6.

### Statistical analysis

Assuming a standard deviation (SD) of 8 mRSS units,^25^ 80% power, a two-sided significance level of 5% and 1:1 randomisation, 128 patients would be required to detect a placebo-adjusted difference of 4 units for intent-to-treat analysis of mRSS. Endpoints were analysed using mixed model repeated measures, with baseline mRSS as a covariate; treatment arm, region and study visit, the interaction effect between study visit and treatment arm as fixed effects and patient-specific random effects (see online supplementary file 3). The primary endpoint was also analysed by analysis of covariance with baseline mRSS as a covariate, and treatment arm and region as main effects. Endpoints present or not were estimated using Mantel-Haenszel weights. Analyses were performed on all patients randomised and treated with study medication using SAS V.9.2 software (SAS Institute Inc, Cary, North Carolina, USA). Since the primary endpoint was not met, all other p values are nominal, are only shown for predefined but not post hoc analyses, cannot be considered statistically significant and are presented for information only.

10.1136/annrheumdis-2019-216823.supp3 Supplementary data

### Patient involvement

Patients were not directly involved in the design, recruitment or conduct of the study.

## Results

### Study population

**Figure 1:**
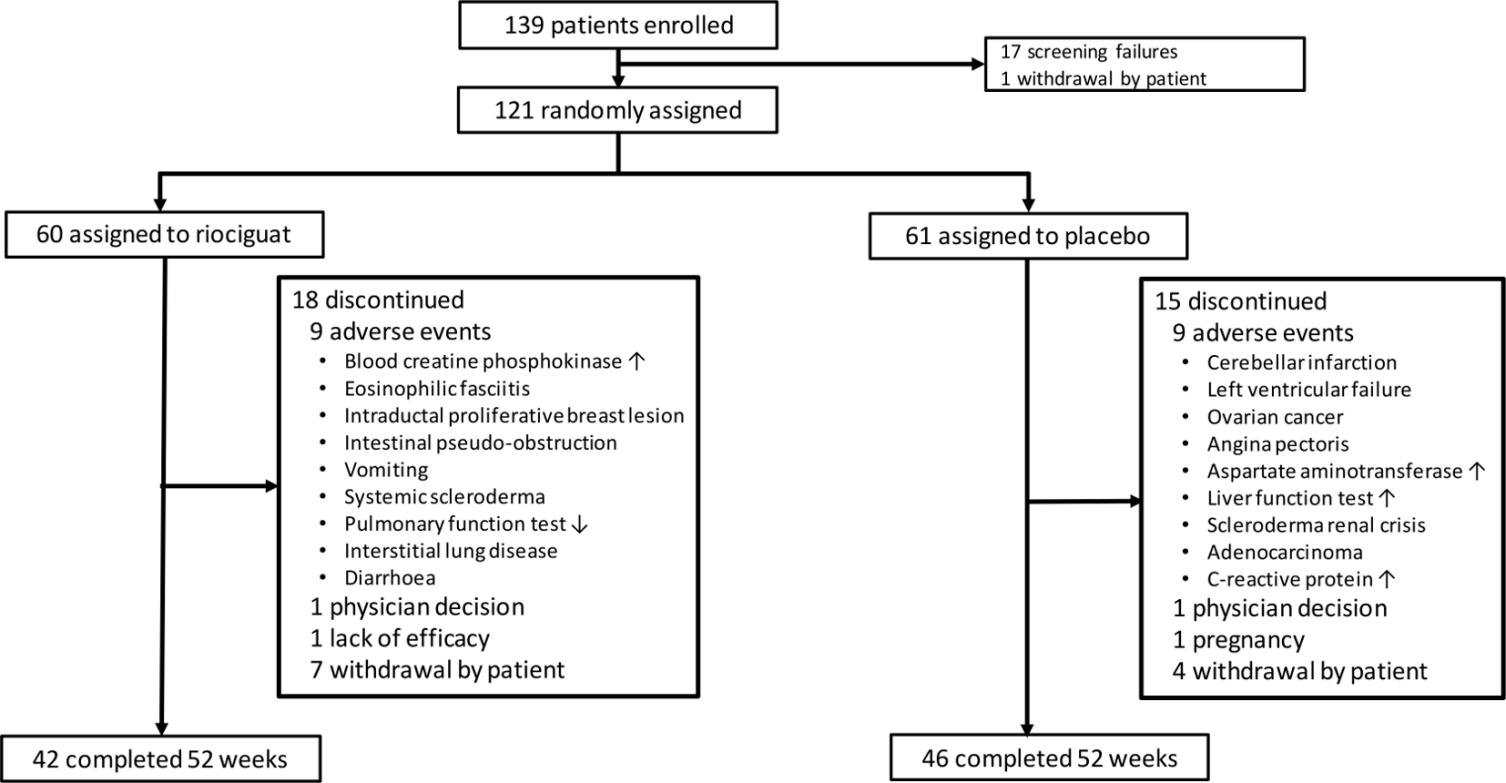
Patient disposition.

In total, 121 patients were randomised (riociguat, n=60; placebo, n=61). The study was completed according to the protocol. Five patients in each group received ≥1 new rescue therapy after week 26. Study discontinuation occurred in 18 (30.0%) riociguat-treated patients and 15 (24.6%) placebo-treated patients (figure 1). At week 52, 34 (80.9%)/42 riociguat-treated patients were receiving riociguat 2 or 2.5 mg three times daily. Patients generally had early dcSSc, with mean mRSS 17 and mean disease duration 8.6 months. Baseline characteristics were generally well balanced across groups (table 1).

**Table 1:** Baseline characteristics of study participants

Characteristics	Overall (n=121)	Riociguat (n=60)	Placebo (n=61)
Mean age (SD), years	50.7 (12.2)	51.9 (11.5)	49.5 (12.9)
Female, n (%)	92 (76.0)	47 (78.3)	45 (73.8)
White, n (%)	89 (73.6)	43 (71.7)	46 (75.4)
Black, n (%)	5 (4.1)	2 (3.3)	3 (4.9)
Asian, n (%)	24 (19.8)	12 (20.0)	12 (19.7)
Native Hawaiian or other Pacific Islander, n (%)	1 (0.8)	1 (1.7)	0
Not reported, n (%)	2 (1.7)	2 (3.3)	0
Mean disease duration (SD), months (from first non-RP manifestation)	9.0 (6.4)	9.5 (7.0)	8.6 (5.8)
Mean mRSS (SD), units	16.8 (3.7)	16.9 (3.4)	16.7 (4.1)
Mean % predicted FVC (SD), %	92.8 (17.8)	90.7 (18.5)	94.8 (17.0)
Mean % predicted DL_CO_ (Hb corr.) (SD), %	76.4 (18.5)	76.0 (19.9)	76.8 (17.2)
Swollen joint count ≥1, n (%)	38 (31.4)	23 (38.3)	15 (24.6)
Mean swollen joint count (SD), n	2.0 (4.7)	3.0 (6.1)	1.1 (2.5)
Tender joint count ≥1, n (%)	51 (42.1)	30 (50.0)	21 (34.4)
Mean tender joint count (SD), n	3.0 (6.2)	3.9 (7.3)	2.1 (4.8)
Digital ulcer count ≥1, n (%)	15 (12.4)	9 (15.0)	6 (9.8)
Mean digital ulcer count (SD), n	0.3 (1.1)	0.3 (0.7)	0.4 (1.4)
Mean digital ulcer count in patients with ulcers (SD), n	2.5 (2.3)	1.7 (1.0)	3.7 (3.2)
Tendon friction rubs ≥1, n (%)	35 (28.9)	15 (25.0)	20 (32.8)
Mean tendon friction rubs (SD), n	3.1 (2.2)	2.4 (1.1)	3.6 (2.7)
ILD by medical history, n (%)	25 (20.7)	12 (20.0)	13 (21.3)
Mean HAQ-DI (SD), units	0.79 (0.68)	0.89 (0.67)	0.69 (0.69)
Anti-RNA polymerase III positive, n (%)	26 (21.5)	10 (16.7)	16 (26.2)
Anti-SCl-70 (anti-topoisomerase I) positive, n (%)	49 (40.5)	26 (43.3)	23 (37.7)
Anti-centromere B positive, n (%)	10 (8.3)	4 (6.7)	6 (9.8)

DL_CO_, diffusing capacity of the lung for carbon monoxide; DL_CO_ (Hb corr.), diffusing capacity of the lung for CO, corrected for haemoglobin; FVC, forced vital capacity; HAQ-DI, Health Assessment Questionnaire Disability Index; ILD, interstitial lung disease; mRSS, modified Rodnan skin score; RP, Raynaud’s phenomenon.

### Skin fibrosis

**Figure 2:**
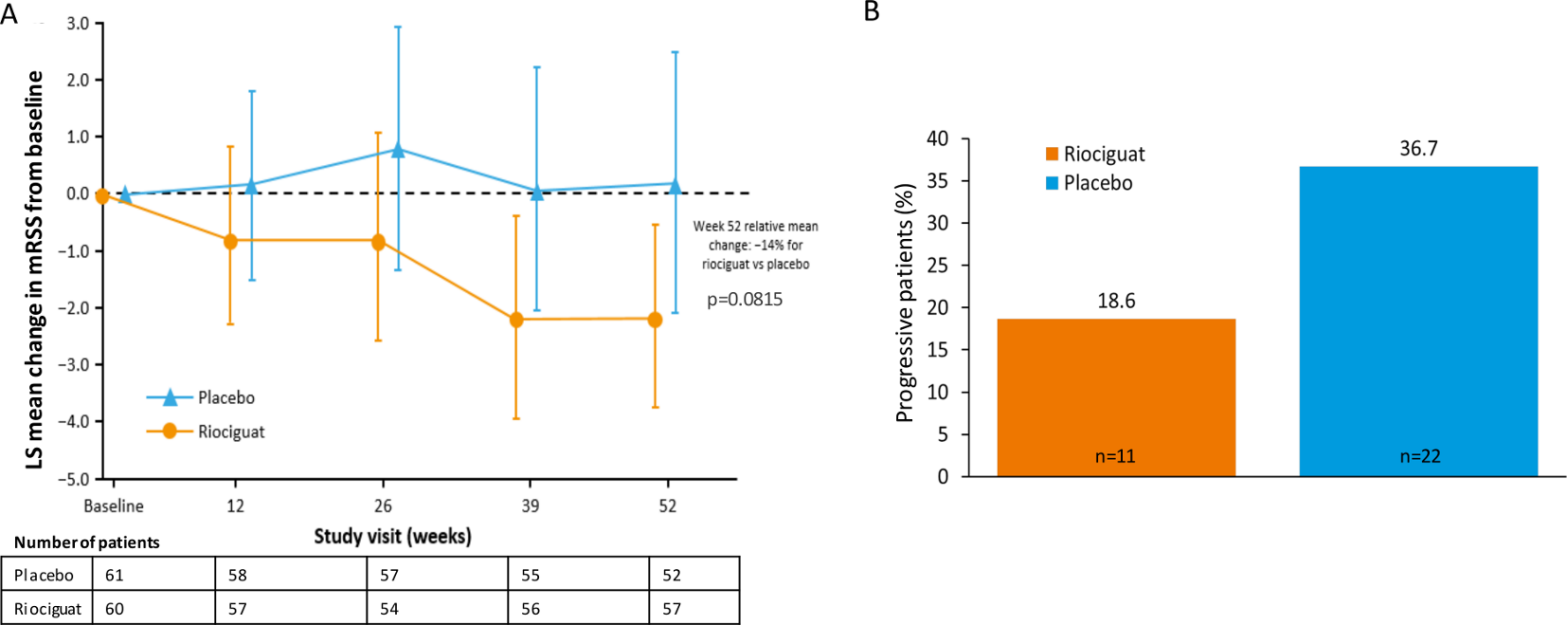
(A) Change from baseline in mRSS during the study. Mixed model with repeated measurement was applied with baseline value, treatment group, region, visit and treatment by visit interaction as fixed effects, and subject as a random effect. Vertical lines represent 95% CI for change. (B) Proportion of patients with mRSS progression (increase in mRSS by >5 units and ≥25% from baseline: prespecified analysis). Treatment comparison (riociguat −placebo): estimate −17.99%, 95% CI −33.57 to −2.40. Mantel-Haenszel estimate of difference: nominal p=0.0237. CI, confidence interval; LS, least squares; mRSS, modified Rodnan skin score.

The primary endpoint was not met at the predefined p<0.05. At week 52, mean mRSS was 14.63 (SD 6.56) with riociguat versus 15.73 (SD 10.48) with placebo: difference of least squares (LS) means –2.34 (standard error (SE) 1.33); 95% confidence interval (CI) –4.99 to 0.30; relative difference –14%; p=0.0815. At week 52, the mean change from baseline in mRSS was –2.09 (SD 5.66) with riociguat and –0.77 (SD 8.24) with placebo (figure 2A). Progression of mRSS (increase by >5 units and ≥25% from baseline) was observed in 11 (18.6%)/59 patients with riociguat and 22 (36.7%)/60 patients with placebo (Mantel-Haenszel estimate of difference: –17.99% (95% CI –33.57% to –2.40%; nominal p=0.0237); figure 2B). Regression rates (decrease by >5 units and ≥25% from baseline) in the riociguat and placebo groups were 27 (45.7%)/59 and 18 (30.0%)/60, respectively (Mantel-Haenszel estimate of difference: 15.29% (95% CI –1.98% to 32.57%; nominal p=0.0827)).

On subgroup analyses, the change in mRSS with riociguat versus placebo showed a nominal p value <0.05 for mRSS 17–22, anti-RNA polymerase III positive/SCl-70 negative, baseline FVC 50%–75% and high-sensitivity C-reactive protein >3.0 mg/L (see online supplementary figure S2).

### Secondary endpoints

ACR CRISS as a measure of improvement did not show significant differences in this trial designed for prevention of worsening. Eighteen per cent of patients in each group had a CRISS improvement probability score ≥0.60 (estimate of difference: 0.20% (95% CI –13.68% to 14.09%; nominal p=0.977)). However, in step 1 of the CRISS analysis, 1 (1.7%) patient in the riociguat group versus 4 (6.6%) in the placebo group met the definition for SSc-related organ involvement. Other secondary endpoints are shown in table 2.

**Table 2:** Difference between riociguat group and placebo group in change from baseline to week 52 in secondary endpoints

Endpoint	Riociguat (n=60)	Placebo (n=61)	Estimate of difference (95% CI)	Nominal p value*
ACR CRISS				
No improvement, n (%)	1 (1.7)	4 (6.6)	0.20% (–13.68 to 14.09)†	0.977
≥3 missing criteria, n (%)	6 (10.0)	7 (11.5)		
CRISS probability ≥60%, n (%)	11 (18.3)	11 (18.0)		
CRISS probability <60%, n (%)	49 (81.7)	50 (82.0)		
Mean HAQ-DI (SD), units				
Baseline	0.89 (0.67)	0.69 (0.69)	–0.07 (–0.23 to 0.08)‡	0.3529
Change at week 52	0.05 (0.38) (n=56)	0.13 (0.42) (n=52)		
Mean patient global assessment (SD), units				
Baseline	3.93 (2.50)	3.77 (2.34)	0.79 (–0.12 to 1.69)‡	0.0887
Change at week 52	0.69 (2.75) (n=45)	–0.02 (2.23) (n=46)		
Mean physician global assessment (SD), units				
Baseline	4.33 (2.11)	4.02 (2.00)	0.83 (0.11 to 1.54)‡	0.0241
Change at week 52	–0.07 (2.16) (n=45)	–0.75 (2.09) (n=47)		
Mean % predicted FVC (SD), %				
Baseline	90.74 (18.52)	94.82 (17.03)	–0.20 (–3.40 to 3.00)‡	0.901
Change at week 52	–2.38 (7.52) (n=55)	–2.95 (9.73) (n=51)		

*Since the primary endpoint was not met, all other p values cannot be considered statistically significant and are presented for information only.

†Mantel-Haenszel estimate.

‡Mixed model repeated measures applied with baseline value, treatment group, region, visit and treatment by visit interaction as fixed effects, and subject as a random effect.

ACR, American College of Rheumatology; CI, confidence interval; CRISS, Composite Response Index for Systemic Sclerosis; FVC, forced vital capacity; HAQ-DI, Health Assessment Questionnaire Disability Index.

### Lung function

**Figure 3:**
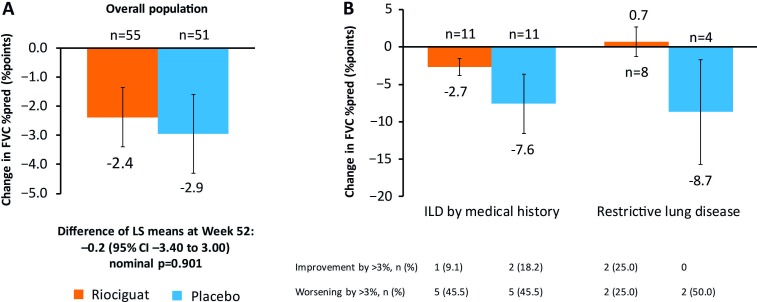
(A) Change in FVC% from baseline to week 52 in overall population. (B) Change in FVC% from baseline to week 52 in patients with lung fibrosis at baseline by diagnostic subgroups (post hoc). Data points are mean (SE). Numbers close to axes are numbers of patients with data at week 52. CI, confidence interval; FVC, forced vital capacity; ILD, interstitial lung disease; LS, least squares; SE, standard error.

Overall, the change in FVC% between baseline and week 52 was −2.38% (SD 7.52) with riociguat and −2.95% (SD 9.73) with placebo (difference of LS means −0.20 (SE 1.61); 95% CI −3.40 to 3.00; nominal p=0.901; figure 3A). Two patients in each group developed new ILD. At baseline, 12 (20.0%) patients receiving riociguat and 13 (21.3%) patients with placebo had SSc-ILD by medical history, and 11 (18.3%) and 7 (11.5%), respectively, had baseline FVC% 50%–75%. Baseline characteristics by lung fibrosis diagnosis are shown in online supplementary table S1. Depending on the diagnosis, the mean change in FVC% from baseline to week 52 was −7.6 to −8.7% with placebo and +0.7 to −2.7% with riociguat (figure 3B).

DL_CO_% decreased by −2.31% (SD 10.08) with riociguat and −4.09% (SD 12.19) with placebo (difference of LS means 2.01 (SE 2.14); 95% CI −2.24 to 6.25; nominal p=0.3502). In patients with ILD by medical history the changes in DL_CO_% were –4.55 (SD 8.12) with riociguat (n=11) and –7.63 (SD 13.37) with placebo (n=12). In those with baseline FVC% 50%–75%, DL_CO_% increased by 2.26 (SD 15.16) with riociguat (n=8) and fell by –7.32 (SD 17.24) with placebo (n=5).

### Raynaud’s phenomenon and digital ulcers

At baseline, 9 (15.0%) patients had DUs in the riociguat group versus 6 (9.8%) in the placebo group. New DUs were reported in 2 (3.3%) patients in the riociguat group and 6 (9.8%) in the placebo group at week 14, and in 5 (8.3%) patients and 12 (19.7%) patients, respectively, at week 52. There were 4 and 26 new DUs with riociguat and placebo, respectively, at week 14; and 12 and 72 new DUs, respectively, at week 52 (see online supplementary figure S3). Concomitant medication with an indication for DU was used by 7 (11.7%) patients receiving riociguat and 10 (16.4%) patients with placebo. Changes from baseline to week 14 in Raynaud’s attack duration, frequency and symptoms favoured riociguat but nominally did not differ significantly between riociguat and placebo (see online supplementary table S2). The average Raynaud’s condition score improved by ≥50% in 19 (41.3%)/46 patients with riociguat and in 13 (26.0%)/50 patients with placebo. At week 52, reductions in net DU burden were –0.09 (SD 0.50) and –0.08 (SD 1.47) with riociguat and placebo, respectively (difference of LS means –0.11 (SE 0.14); 95% CI –0.38 to 0.17; nominal p=0.4444). No case of critical digital ischaemia occurred in either group.

### Other endpoints

Findings from prespecified exploratory analyses and post hoc assessments are provided in online supplementary file 1, p12–19.

### Adverse events

Overall, 58 (96.7%) patients in the riociguat group and 55 (90.2%) in the placebo group experienced an AE (see online supplementary table S8). Most AEs in the riociguat group were mild to moderate, and most were gastrointestinal events (eg, gastro-oesophageal reflux disease, diarrhoea or nausea) or nervous system disorders (eg, dizziness, headache). Symptomatic hypotension was reported in 7 (11.7%) patients with riociguat and 6 (9.8%) patients with placebo. SAEs were reported in 9 (15.0%) patients in the riociguat group and 15 (24.6%) in the placebo group (table 3). Eleven patients in each group had AEs resulting in discontinuation of study drug (see online supplementary table S9). No events of serious haemoptysis were reported. One patient in the riociguat group died from myocardial infarction 117 days after the last administration of riociguat and one patient in the placebo group died from left ventricular failure 157 days after the last administration of placebo. Neither death was considered related to study drug.

**Table 3:** Serious adverse events

Patients reporting event, n (%)		
Event	Riociguat (n=60)	Placebo (n=61)
Any SAE	9 (15.0)	15 (24.6)
Any study drug-related SAE	0	2 (3.3)
Discontinuation of study drug due to SAE	2 (3.3)	7 (11.5)
Angina pectoris	1 (1.7)	1 (1.6)
Atrial fibrillation	1 (1.7)	0
Abdominal pain	1 (1.7)	0
Intestinal pseudo-obstruction	1 (1.7)	0
Inflammation	1 (1.7)	0
Lung infection	1 (1.7)	0
Pneumonia	1 (1.7)	2 (3.3)
RP	1 (1.7)	1 (1.6)
Musculoskeletal discomfort	1 (1.7)	0
Pain in extremity	1 (1.7)	0
Dyspnoea	1 (1.7)	0
Intraductal proliferative breast lesion	1 (1.7)	0
Pericarditis	0	2 (3.3)
Left ventricular failure	0	1 (1.6)
Ventricular tachycardia	0	1 (1.6)
Gastric haemorrhage	0	1 (1.6)
Gastro-oesophageal reflux disease	0	1 (1.6)
Nausea	0	1 (1.6)
Vomiting	0	1 (1.6)
Infected skin ulcer	0	1 (1.6)
Anaemia	0	1 (1.6)
Exposure during pregnancy	0	1 (1.6)
Osteolysis	0	1 (1.6)
Scleroderma	0	1 (1.6)
Acute myeloid leukaemia	0	1 (1.6)
Gastric adenocarcinoma	0	1 (1.6)
Ovarian cancer	0	1 (1.6)
Cerebellar infarction	0	1 (1.6)
Syncope	0	1 (1.6)
Scleroderma renal crisis	0	1 (1.6)
Acute pulmonary oedema	0	1 (1.6)
Skin ulcer	0	1 (1.6)
Surgical/medical prophylaxis	0	1 (1.6)

MedDRA preferred terms are shown.

MedDRA, Medical Directory for Regulatory Activities; RP, Raynaud’s phenomenon; SAE, serious adverse event.

Of those with ILD by medical history, AEs were reported in 10 (83.3%)/12 patients with riociguat and 12 (92.3%)/13 patients with placebo. AEs reported more frequently with riociguat than with placebo were predominantly dizziness and gastrointestinal events (see online supplementary table S10). The incidence of respiratory, thoracic and mediastinal AEs was similar with riociguat (4 patients; 33.3%) and placebo (4 patients; 30.8%). SAEs were reported in 1 (8.3%)/12 and 3 (23.1%)/13 patients, respectively. Safety in patients with baseline FVC% 50%–75% showed no overall excess of AEs with riociguat (see online supplementary table S11).

## Discussion

RISE-SSc investigated the effects of riociguat on disease progression in patients with early dcSSc. mRSS was selected as the primary endpoint as it correlates with biopsy measures of skin thickness and reflects disease prognosis and visceral involvement.^1 26^ mRSS does, however, have challenging and unpredictable changes over the disease course and attempts to enrich trial populations with patients likely to progress have not been successful. Nevertheless, it is a validated surrogate marker of disease progression^27^ and is accepted by authorities as an endpoint for skin fibrosis.^22^ RISE-SSc was the first trial in SSc with the EUSTAR inclusion criteria designed to enrich the population with patients likely to show progression of skin fibrosis. Between baseline and week 52, 36.7% of placebo-treated patients showed skin fibrosis progression, which is much higher than in similar trials,^25 28–30^ showing that our enrichment strategy was successful. This is consistent with other evidence that patients with baseline mRSS 15–22 and early disease showed higher progression rates than unselected cohorts.^17 18 31^

There are several potential reasons why the primary endpoint was not met in this study. First, RISE-SSc was designed to detect a placebo-adjusted change of mRSS between riociguat and placebo with 80% power. For the low baseline mRSS expected in this study, a 4-unit change would represent a change of 23%. The between-groups difference was 2.3, which was less than expected. This low treatment effect was probably the main reason why the primary endpoint was not met. In addition, the higher than expected numbers of skin fibrosis regressors^18^ and stable patients reduced the sensitivity of RISE-SSc to detect a significant change of mRSS. This is consistent with previous trials, in which mRSS improvements were observed in the majority of patients receiving placebo.^32 33^ Other possible explanations include the very large variation in mRSS scores during the study.

As expected, the combined secondary endpoint did not favour riociguat because the ACR CRISS evaluates disease improvement, whereas RISE-SSc was designed to detect prevention of progression. ACR CRISS is not expected to be positive in such a trial design.^24^

Some measures of mRSS, lung function in patients with evidence for pre-existing ILD and the prevention of new DU and RP symptoms suggest potential signals for efficacy. It is important to note that the descriptive analyses of predefined secondary and exploratory endpoints should not be interpreted as efficacy of riociguat, but as a potential signal that can be investigated in further studies.

AEs reported more frequently with riociguat than placebo were mainly gastrointestinal events, dizziness or peripheral oedema. These events are consistent with the effects of riociguat, such as relaxation of smooth muscle cells in the vasculature (often associated with blood pressure decrease) or the gastrointestinal tract and did not increase the incidence of withdrawal due to AEs. SAEs were less common with riociguat than with placebo, no riociguat-treated patient experienced an SAE considered related to study treatment, and fewer discontinued study medication because of an SAE with riociguat than with placebo. Riociguat was, therefore, well tolerated in early dcSSc, particularly when compared with traditional immunosuppressive agents.^34 35^ Tolerability was also good in patients with ILD, which is important given the increased rates of death and SAEs with riociguat in a study in patients with pulmonary hypertension associated with idiopathic interstitial pneumonia.^36^

Discontinuation rates (≈30% with riociguat and ≈25% with placebo) were higher in RISE-SSc than with active treatment in recent trials of abatacept (23%)^37^ or tocilizumab (9%)^38^ in SSc. RISE-SSc recruited patients with very early disease (compared with these trials, which recruited patients with ≤36 and≤60 months from onset of SSc, respectively). The early discontinuation may be related to the expectation of worsening of SSc in early disease (based on natural history), where AEs may lead the investigator to withdraw the patient (see online supplementary table S9), especially in a placebo-controlled trial. Indeed, another trial with a comparable very early disease population showed a discontinuation rate of 40% in the active treatment (CAT-192) group.^39^ Another explanation might be anxiety associated with early disease in the participants; however, these are speculations and should be explored in other trials in patients with very early disease. AEs in the riociguat and placebo groups contributed substantially to the discontinuations in the current study.

In conclusion, RISE-SSc failed to meet its primary endpoint and is therefore a negative trial. However, it provides important findings for the identification of patients at high risk of skin fibrosis progression that could inform future studies in patients with dcSSc. In addition, there are potential efficacy signals in early dcSSc and these may be explored further with additional randomised controlled trials.
