# Supplementary material for: Riociguat in patients with early diffuse cutaneous systemic sclerosis (RISE-SSc): randomised, double-blind, placebo-controlled multicentre trial
Source: Ann Rheum Dis. 2020 Apr 15;79(5):618–25. doi: 10.1136/annrheumdis-2019-216823 (PMC7213318; doi:10.1136/annrheumdis-2019-216823)
Supplement: Supplementary data [file annrheumdis-2019-216823supp001.pdf]

SUPPLEMENTARY DATA FILE 1

Participating Countries in RISE-SSc

We conducted the main phase between 15 January 2015 and 15 December 2017 in 60 outpatient hospital centres in 15 countries: Australia, New Zealand, Canada, USA, Belgium, the Czech Republic, France, Germany, Hungary, Italy, Switzerland, United Kingdom, The Netherlands, Turkey and Japan.

Exclusion Criteria

Patients who met any of the following criteria were excluded from enrolment in the study.

1. Medical and surgical history

- Limited cutaneous SSc at screening.
- Major surgery (including joint surgery) within 8 weeks prior to screening.
- Patients with a history of malignancy in the last 5 years other than non-melanoma skin cell cancers cured by local resection or carcinoma in situ.
- Known hypersensitivity to the study drug (active substance or excipients).

2. Hepatic-related criteria

- Hepatic insufficiency classified as Child-Pugh C:
    - Patients with isolated aspartate aminotransferase (AST) or alanine aminotransferase (ALT) >3 × upper limit of normal (ULN) or bilirubin >2 × ULN could be included under the condition of additional monitoring during the trial.

3. Renal-related criteria

- Estimated glomerular filtration rate (eGFR) < 15 mL/min/1.73 m^2 (Modification of Diet in Renal Disease [MDRD] formula) or on dialysis at the screening visit:
    - Patients entering the trial with eGFR 15−29 mL/min/1.73 m^2 underwent additional monitoring of renal function.

Khanna D, *et al. Ann Rheum Dis* 2020; 79:618–625. doi: 10.1136/annrheumdis-2019-216823

- Because the MDRD formula is thought to cause significant bias for Japanese patients, the equation for Japanese patients is: 194 × serum creatinine (mg/dL)$^{-1.094}$ × age$^{-0.287}$ × 0.739 (if female).
- Any prior history of renal crisis.

4. Cardiovascular-related criteria

- Sitting systolic blood pressure <95 mmHg at the screening visit.
- Sitting heart rate <50 beats per minute at the screening visit.
- Left ventricular ejection fraction <40% prior to screening.

5. Pulmonary-related criteria

- Any form of pulmonary hypertension as determined by right heart catheterisation.
- Pulmonary disease with percent predicted FVC <45% or per cent predicted diffusing capacity of the lung for carbon monoxide (DLCO) (haemoglobin corrected) <40% of predicted at screening.
- Active state of haemoptysis or pulmonary haemorrhage, including those events managed by bronchial artery embolisation.
- Any history of bronchial artery embolisation or massive haemoptysis within 3 months before screening. (Massive haemoptysis was defined as acute bleeding >240 mL in a 24-hour period or recurrent bleeding >100 mL/day over consecutive days.)

6. Laboratory examinations

- Patients with: haemoglobin <9.0 g/dL, white blood cell count <3000/mm^3 (<3 × 10^9/L), platelet count <100 000/mm^3 (<100 × 10^9/L).

7. Prior and concomitant therapy

- Concomitant use of nitrates or nitric oxide donors (such as amyl nitrate) in any form, including topical; phosphodiesterase (PDE) 5 (PDE5) inhibitors (such as sildenafil, tadalafil, vardenafil); and non-specific PDE5 inhibitors (theophylline, dipyridamole).

Khanna D, *et al. Ann Rheum Dis* 2020; 79:618–625. doi: 10.1136/annrheumdis-2019-216823

- Concomitant therapy with prostacyclin analogues. Oral beraprost for the treatment of digital ulcers/Raynaud's disease, and short-term/intermittent therapy of up to 21 days with intravenous prostacyclin analogues for digital/vascular lesions was allowed.
- Treatment with methotrexate, cyclophosphamide, hydroxychloroquine, cyclosporine A, azathioprine, mycophenolate mofetil, rapamycin, colchicine, D-penicillamine, tacrolimus, mizoribine or intravenous immunoglobulin within 4 weeks before the screening visit.
- Treatment with etanercept within 2 weeks; infliximab, leflunomide, certolizumab, golimumab, adalimumab, abatacept or tocilizumab within 8 weeks; or anakinra within 1 week prior to the screening visit.
- Previous treatment with chlorambucil, bone marrow transplantation or total lymphoid irradiation.
- Treatment with rituximab or other anti-CD20 antibodies within the last 6 months before screening.

8. Other

- Pregnant women or breastfeeding women.
- Women of childbearing potential not willing to use adequate contraception and not willing to agree to 4-weekly pregnancy testing from Visit 1 (first administration of study drug) onwards until 30 (+5) days after last study drug intake.
- Any other condition or therapy that would make the patient unsuitable for this study and will not allow participation for the full planned study period.
- Previous assignment to treatment during this study.
- Participation in another clinical study with an investigational drug or medical device within 30 days prior to randomisation (phases I–III clinical studies).

Study Randomisation, Blinding and Intervention

Patients were randomised 1:1 to riociguat or placebo using the IxRS interactive voice response system (Bayer AG, Berlin, Germany), with permutated blocks sized as a multiple of 2. Data remained

Khanna D, *et al. Ann Rheum Dis* 2020; 79:618–625. doi: 10.1136/annrheumdis-2019-216823

blinded until database lock unless a suspected adverse reaction occurred. Patients randomised to placebo underwent sham adjustment during dose adjustment. All packaging was designed to maintain blinding for investigators and patients, and riociguat and placebo tablets looked, smelled and tasted identical. Study data remained blinded until database lock unless a suspected adverse reaction occurred. An independent Data Monitoring Committee reviewed all data for safety.

Rescue Medication

From week 26, rescue therapy (methotrexate, mycophenolate mofetil, cyclophosphamide, azathioprine or hydroxychloroquine) was permitted at investigator discretion for worsening of skin disease, pulmonary function, inflammatory joint disease or myositis.

Subgroup Analyses of Primary Endpoint

Descriptive analyses of the primary endpoint were performed for the following subgroups:

- region (North America, Europe and Australia/New Zealand, East Asia)
- gender (males/females)age (age <65 years/age ≥65 years)
- mRSS at baseline (10−16 units/17−22 units)
- disease duration at baseline (0−6 months, 7−12 months, 13−18 months)
- antibody at baseline (SCL-70, RNA polymerase III, both, neither)
- ILD (defined with preferred terms: interstitial lung disease and pulmonary fibrosis) at baseline (yes/no)
- FVC%, predicted at baseline (<50, 50−75, >75)
- hsCRP elevated at baseline (≤3.0 mg/L, >3.0 mg/L; and ≤10.0 mg/L, >10.0 mg/L)
- use of corticosteroids at baseline (yes/no)
- tendon friction at baseline (yes/no).

Additional Information on Study Endpoints

The key secondary endpoint was the American College of Rheumatology CRISS at week 52.[24] Application of the CRISS algorithm in a randomised clinical trial was a 2-step process. In Step 1, patients were evaluated to determine whether they had met the criteria for not having improved. A

Khanna D, *et al. Ann Rheum Dis* 2020; 79:618–625. doi: 10.1136/annrheumdis-2019-216823

patient was considered not improved and was assigned a probability score of improving of 0.0, irrespective of improvement on other core items, if he/she developed:

1. New scleroderma renal crisis.
2. Decline in per cent predicted FVC ≥15% (relative), confirmed by another FVC% within a month, HRCT to confirm interstitial lung disease (ILD) (if previous HRCT did not show ILD) and per cent predicted FVC <80% (attributable to SSc).
3. New onset of left ventricular failure (defined as ejection fraction ≤45%) or new onset of PAH requiring treatment (attributable to SSc).

For the remaining patients, the probability of improvement was calculated in Step 2, based on the changes in mRSS, per cent predicted FVC, HAQ-DI, patient's global assessment, and physician's global assessment, in which each measure had a probability score between 0 and 1.

The probability of improving (a score between 0.0 and 1.0, inclusive) was calculated for each patient using the equation:

$$\frac{exp[-5.54-0.81*\Delta_{MRSS}+0.21*\Delta_{FVC\%}-0.40*\Delta_{Pt-glob}-0.44*\Delta_{MD-glob}-3.41*\Delta_{HAQ-DI}]}{1+exp[-5.54-0.81*\Delta_{MRSS}+0.21*\Delta_{FVC\%}-0.40*\Delta_{Pt-glob}-0.44*\Delta_{MD-glob}-3.41*\Delta_{HAQ-DI}]}$$

where Δ_{MRSS} indicates the change in mRSS from baseline to week 52, $\Delta_{FVC\%}$ denotes the change in percent predicted FVC from baseline to week 52, $\Delta_{Pt-glob}$ indicates the change in patient global assessment, $\Delta_{MD-glob}$ denotes the change in physician global assessment, and Δ_{HAQ-DI} is the change in HAQ-DI.

If a patient had one or two missing components, then previous non-missing value of that component was be used. Patients with three or more missing CRISS components were assigned a probability of 0.0.

Clinical Outcomes Potentially Representing Systemic Organ Manifestations Related to dcSSc

Clinical outcomes potentially representing systemic organ manifestations related to dcSSc were defined in the protocol as follows: new renal crisis; worsening of cardiac disease considered secondary to dcSSc; new-onset pulmonary hypertension requiring treatment; pericardial disease

Khanna D, *et al. Ann Rheum Dis* 2020; 79:618–625. doi: 10.1136/annrheumdis-2019-216823

requiring intervention or exhibiting clinical decompensation; arrhythmias and/or cardiac conduction defects requiring treatment; worsening of gastrointestinal disease requiring hospitalisation or new requirement for parenteral nutrition; critical digital ischaemia requiring hospitalisation; or digital gangrene.

Prespecified Exploratory Analyses and Post-Hoc Assessments

Health-related quality of life using the Short Form 36 (SF-36) Questionnaire version 2.0 and the Scleroderma Health Assessment Questionnaire (S-HAQ) and Patient-Reported Outcomes Measurement Information System (PROMIS)-29 scores (in English-speaking countries) were prespecified exploratory analyses. Clinically significant improvement in HAQ-DI (decrease from baseline ≥0.21 at week 52[40]) and a composite endpoint of disease progression (increase of mRSS ≥4, or absolute decrease of FVC% ≥10%, or new organ involvement as defined in ACR CRISS Step 1) were assessed post hoc.

Supplementary Figure S1. RISE-SSc Trial Design.

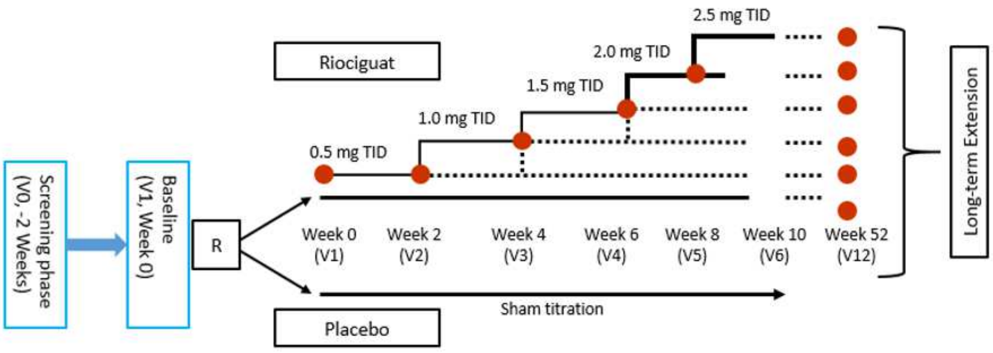

R, randomised; TID, three times daily.

Supplementary Figure S2. mRSS Subgroup Analysis at Week 52 (Prespecified Analysis).

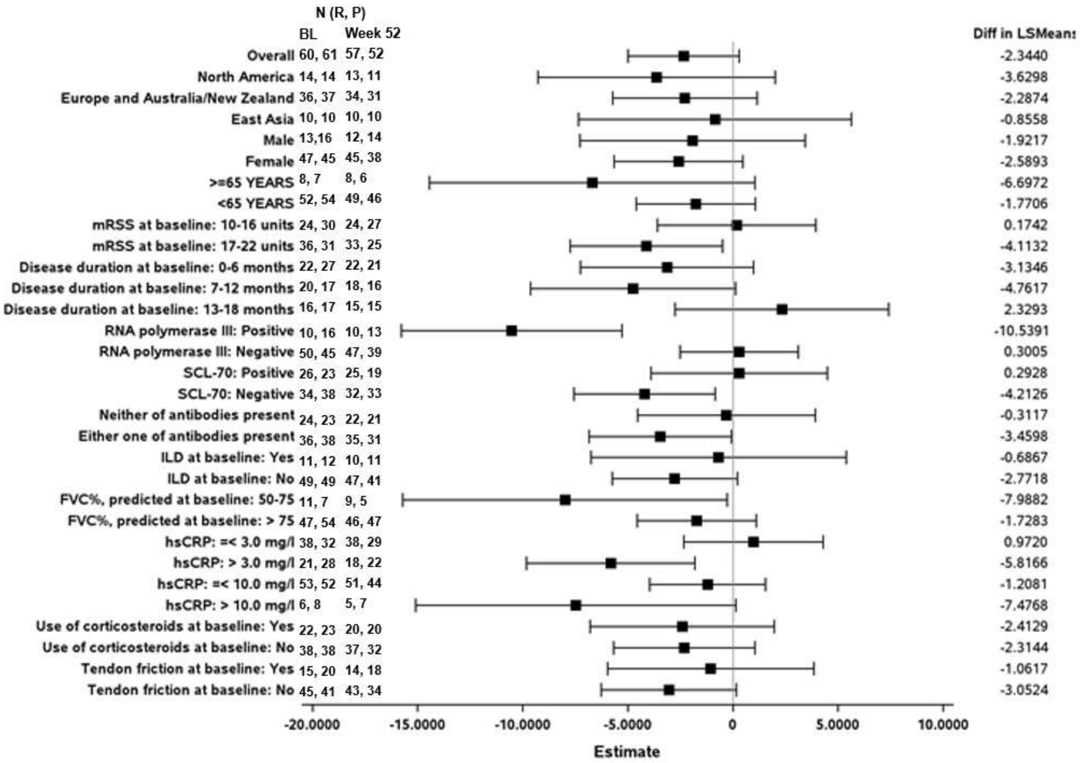

Each square corresponds to the difference in LS means between riociguat and placebo for each subgroup and the line represents the 95% CI. Estimates at week 52 are shown.

Note. hsCRP levels of 3 and 10 mg/L are cut-off levels for assessment of cardiovascular risk or diagnosis of acute infections respectively.

BL, baseline; CI, confidence interval; FVC, forced vital capacity; hsCRP, high-sensitivity C-reactive protein; ILD, interstitial lung disease; LS, least squares; mRSS, modified Rodnan skin score; P, placebo; R, riociguat; RNA, ribonucleic acid; SCL-70, anti-topoisomerase I antibodies.

Khanna D, *et al. Ann Rheum Dis* 2020; 79:618–625. doi: 10.1136/annrheumdis-2019-216823

Supplementary Figure S3. Development of New Digital Ulcers.

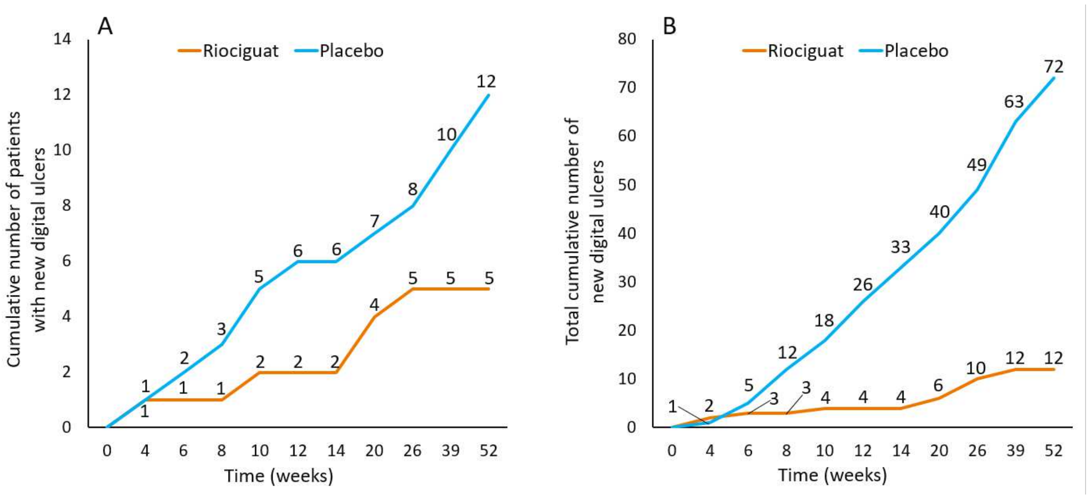

A. Cumulative numbers of patients with new digital ulcers. **B.** Cumulative numbers of new digital ulcers.

Digital ulcers are defined as full-thickness skin lesions with loss of epithelium, including lesions covered by eschar. Ulcers should be >3 mm in maximal diameter.

New digital ulcers are defined as ulcers not existing at baseline.

Please note that patients receiving concomitant nitrates, nitric oxide donors, phosphodiesterase inhibitors and long-term prostacyclin analogues therapy were not included in the study, which might have influenced the study population.

Nominal p-values were not calculated for post-hoc analyses.

Khanna D, *et al. Ann Rheum Dis* 2020; 79:618–625. doi: 10.1136/annrheumdis-2019-216823

Supplementary Table S1. Baseline Characteristics of Study Participants with ILD

	**ILD by medical history**		**FVC% 50–75% at baseline**	
**Characteristic**	**Riociguat (n=12)**	**Placebo (n=13)**	**Riociguat (n=11)**	**Placebo (n=7)**
Mean age (SD), *y*	58 (8.7)	50 (15.2)	47 (11.5)	49 (16.7)
Female, n (%)	9 (75.0)	10 (76.9)	9 (81.8)	6 (85.7)
White, n (%)	7 (58.3)	8 (61.5)	9 (81.8)	7 (100)
Median (range) disease duration, *months*	6.7 (0.6–44.4)	11.2 (0.9–17.6)	13.8 (6.8–18.0)	12.6 (5.2–16.8)
Mean mRSS (SD), *units*	15.1 (3.9)	16.6 (4.5)	19.3 (2.5)	17.3 (4.7)
Mean % predicted FVC (SD), *%*	82.8 (23.1)	91.0 (21.9)	69.2 (7.9)	70.1 (6.6)
Mean % predicted DL_{CO} (Hb corr.), (SD), *%*	75.7 (22.6)	69.6 (16.2)	67.1 (15.5)	68.7 (15.1)

DL_{CO} (Hb corr.), diffusing capacity for carbon monoxide corrected for haemoglobin; FVC, forced vital capacity; ILD, interstitial lung disease; mRSS, modified Rodnan skin score; SD, standard deviation.

Khanna D, *et al. Ann Rheum Dis* 2020; 79:618–625. doi: 10.1136/annrheumdis-2019-216823

Supplementary Table S2. Change in Raynaud´s Attacks from Baseline to Week 14

	**Riociguat**			**Placebo**			**Nominal p-value**[†]
	**Baseline, mean (SD) (range)**	**Absolute change**	**Relative change (%)***	**Baseline, mean (SD) (range)**	**Absolute change**	**Relative change (%)***	
Duration of attacks per day, *min*	38.7 (54.8) (0.0–228.6) (n=58)	–12.9 (n=52)	–33	73.0 (139.8) (0.0–728.6) (n=60)	–14.4 (n=52)	–20	N/A
Attacks per day, n	2.5 (2.7) (0.0–11.6) (n=58)	–1.2 (n=52)	–49	2.0 (2.2) (0.0–12.3) (n=60)	–0.6 (n=52)	–28	N/A
Raynaud's condition score, *units* (range 0–10)	3.1 (2.5) (0.0–8.4) (n=56)	–0.9 (n=45)	–30	2.7 (2.6) (0.0–9.6) (n=60)	–0.4 (n=49)	–13	0.4132
Patient assessment, *units* (range 0–100)	29.1 (26.3) (0.0–94.0) (n=58)	–10.1 (n=49)	–35	26.9 (26.7) (0.0–100.0) (n=60)	–0.8 (n=52)	–3	0.0622
Physician assessment, *units* (range 0–100)	31.5 (24.2) (0.0–83.0) (n=58)	–12.8 (n=50)	–40	36.9 (28.3) (0.0–94.0) (n=61)	–9.6 (n=54)	–26	0.2780
Pain (attack symptom, *units*; range 0–100)	24.6 (25.6) (0.0–82.6) (n=51)	–6.9 (n=40)	–28	21.5 (26.4) (0.0–90.0) (n=57)	–1.8 (n=44)	–9	N/A
Numbness (attack symptom, *units*; range 0–100)	26.0 (25.6) (0.0–89.3) (n=51)	–5.7 (n=39)	–22	22.0 (24.2) (0.0–91.4) (n=57)	–0.2 (n=44)	–1	N/A

Tingling (attack symptom, *units*; range 0–100)	20.9 (23.1) (0.0–81.6) (n=51)	–3.0 (n=39)	–14	16.9 (22.5) (0.0–80.0) (n=57)	+1.4 (n=43)	+8	N/A

*Percentage calculated as mean change from baseline to week 14/mean baseline value × 100.

†Since the primary endpoint was not met, all other p-values cannot be considered statistically significant and are presented for information only.

N/A, not applicable (post hoc analyses); SD, standard deviation.

Prespecified Exploratory Analyses and Post-Hoc Assessments

At week 14, S-HAQ patient-reported interference with daily activities by RP declined by −0.34 (SD 0.71) with riociguat and by −0.19 (SD 0.73) with placebo (difference of LS means –0.12 [SE 0.11]; 95% CI, –0.33 to 0.10; nominal p=0.295).

Adjudicated clinical outcome events related to SSc were reported in 4 patients (6.7%) in the riociguat group and 6 (9.9%) in the placebo group (online supplementary table S3). Changes in PROMIS-29 scores (online supplementary table S4), SF-36 scores (online supplementary table S5) or S-HAQ scores (online supplementary table S6) did not differ substantially between treatment groups. Improvement in HAQ-DI was reported in 11/56 (19.6%) riociguat patients and 7/52 (13.5%) of placebo patients. Time to the composite endpoint of progression was longer with riociguat than with placebo.

Khanna D, *et al. Ann Rheum Dis* 2020; 79:618–625. doi: 10.1136/annrheumdis-2019-216823

Supplementary Table S3. Prespecified Analysis of Adjudicated Clinical Outcome Events

Patients Reporting Event, n (%)		
**Event**	**Riociguat (n=60)**	**Placebo (n=61)**
Any	4 (6.7)	6 (9.8)
New renal crisis	0	1 (1.6)
Scleroderma renal crisis	0	1 (1.6)
Worsening of cardiac disease defined as new or worsened clinically symptomatic and significant heart disease considered secondary to dcSSc	0	1 (1.6)
Left ventricular failure	0	1 (1.6)
Pericardial disease requiring intervention or exhibiting clinical decompensation	0	1 (1.6)
Category not recorded	0	1 (1.6)
Pericarditis	0	1 (1.6)
Arrhythmias or conduction defects requiring treatment	0	2 (3.3)
Sinus tachycardia	0	1 (1.6)
Ventricular tachycardia	0	1 (1.6)
Worsening gastrointestinal disease requiring hospitalisation	2 (3.3)	0
Abdominal pain	1 (1.7)	0
Intestinal pseudo-obstruction	1 (1.7)	0
New requirement for total parenteral nutrition	1 (1.7)	0
Abdominal pain	1 (1.7)	0
Non-SSc-related events	1 (1.7)	2 (3.3)
Atrial fibrillation	1 (1.7)	0
Pericarditis	0	1 (1.6)
Vomiting	0	1 (1.6)

Khanna D, *et al. Ann Rheum Dis* 2020; 79:618–625. doi: 10.1136/annrheumdis-2019-216823

Unknown	2 (3.3)	1 (1.6)
Atrial fibrillation	1 (1.7)	0
Atrioventricular block	1 (1.7)	0
Gastroesophageal reflux disease	0	1 (1.6)

dcSSc, diffuse cutaneous systemic sclerosis; SSc, systemic sclerosis.

Khanna D, *et al. Ann Rheum Dis* 2020; 79:618–625. doi: 10.1136/annrheumdis-2019-216823

Supplementary Table S4. Change in PROMIS-29 Scores from Baseline to Week 52

Score	Riociguat		Placebo	
	**Mean (SD) score at baseline, *units* (n=22)**	**Mean (SD) change at week 52, *units* (n=20)**	**Mean (SD) score at baseline, *units* (n=22)**	**Mean (SD) change at week 52, *units* (n=18)**
Physical function	40.75 (6.49)	–0.81 (4.84)	43.82 (7.77)	–3.73 (7.24)
Anxiety	50.64 (8.85)[†]	–2.21 (9.18)[‡]	50.21 (9.89)	0.51 (7.43)
Depression	48.94 (7.57)[†]	–1.92 (6.80)[‡]	46.37 (8.21)	4.32 (6.94)
Fatigue	56.66 (10.68)[†]	–2.95 (7.39)[‡]	52.61 (11.59)	2.40 (10.27)
Sleep disturbance	51.58 (4.86)	0.83 (3.25)	51.30 (4.73)	1.57 (5.19)
Satisfaction with social role	41.80 (9.06)	3.62 (9.85)	44.49 (11.85)	–1.00 (7.92)
Pain interference	58.02 (8.90)	1.41 (8.30)	55.88 (9.44)	–0.02 (7.18)

[†]n=21.

[‡]n=19.

LS, least squares; PROMIS-29, Patient-Reported Outcomes Measurement Information System; SD, standard deviation.

Khanna D, *et al. Ann Rheum Dis* 2020; 79:618–625. doi: 10.1136/annrheumdis-2019-216823

Supplementary Table S5. Change in SF-36 Scores from Baseline to Week 52

Score	Riociguat		Placebo		Nominal p-value*[†]
	**Mean (SD) score at baseline, *units* (n=60)**	**Mean (SD) change at week 52, *units* (n=56)**	**Mean (SD) score at baseline, *units* (n=61)**	**Mean (SD) change at week 52, *units* (n=52)**	
Bodily pain	53.5 (27.53)	–1.21 (22.23)	57.64 (24.89)	4.17 (22.62)	0.1432
General health	48.03 (20.54)	–4.23 (17.24)	52.92 (21.42)	–5.54 (18.63)	0.8918
Mental health	66.67 (20.08)	0.27 (21.03)	69.43 (18.93)	–0.77 (17.86)	0.9724
Physical functioning	59.92 (26.13)	–2.32 (16.35)	66.39 (25.79)	0.99 (18.11)	0.2245
Role emotional	75.83 (27.73)	–5.36 (31.96)	72.95 (26.16)	–1.92 (28.47)	0.7347
Role physical	59.90 (30.54)	–6.36 (24.06)	62.91 (30.27)	–4.93 (25.28)	0.6886
Social functioning	71.46 (26.95)	–2.23 (24.78)	71.11 (26.17)	–0.48 (27.34)	0.7178
Vitality	47.71 (21.15)	0.67 (14.14)	50.79 (22.13)	0.12 (18.38)	0.8321
Mental component score	47.93 (10.73)	–0.50 (11.57)	47.51 (10.29)	–0.57 (10.35)	0.8613
Physical component score	41.38 (10.32)	–1.42 (6.16)	43.88 (10.33)	–0.34 (6.93)	0.2209
Mental health enhanced score	9.82 (7.06)	0.03 (7.91)	8.82 (6.46)	0.23 (6.31)	0.9170
Health utility index	0.65 (0.13)	–0.01 (0.10)	0.66 (0.12)[‡]	0.00 (0.10)[§]	0.6979

*Refers to estimated treatment difference for LS means (riociguat – placebo at week 52).

[‡]n=60.

[§]n=51.

LS, least squares; SD, standard deviation; SF-36, Short Form 36.

[†]Since the primary endpoint was not met, all other p-values cannot be considered statistically significant and are presented for information only.

Khanna D, *et al. Ann Rheum Dis* 2020; 79:618–625. doi: 10.1136/annrheumdis-2019-216823

Supplementary Table S6. Change in S-HAQ Scores from Baseline to Week 52

Score	Riociguat		Placebo		Nominal p-value*†
	Mean (SD) score at baseline, *units* (n=60)	Mean (SD) change at week 52, *units* (n=56)	Mean (SD) score at baseline, *units* (n=61)	Mean (SD) change at week 52, *units* (n=52)	
Pain in past week	1.02 (0.88)	–0.01 (0.78)	0.85 (0.82)	–0.04 (0.66)	0.5952
Intestinal problems in past week	0.49 (0.80)‡	0.12 (0.60)§	0.37 (0.67)	0.12 (0.67)	0.6803
Breathing problems in past week	0.48 (0.75)	0.05 (0.37)	0.27 (0.51)	0.19 (0.56)	0.1267
Raynaud's in past week	0.72 (0.86)‡	0.00 (0.76)§	0.70 (0.83)	–0.09 (0.67)	0.6623
Finger ulcers in past week	0.32 (0.70)	0.08 (0.72)	0.30 (0.68)	0.11 (0.74)	0.5205
Overall disease rating	1.01 (0.86)	0.10 (0.78)	1.05 (0.88)	–0.10 (0.83)	0.4910

Data are expressed as mean (SD).

*Refers to estimated treatment difference for LS means (riociguat – placebo at week 52).

‡n=59.

§n=55.

LS, least squares; SD, standard deviation; S-HAQ, Scleroderma Health Assessment Questionnaire.

†Since the primary endpoint was not met, all other p-values cannot be considered statistically significant and are presented for information only.

Khanna D, *et al. Ann Rheum Dis* 2020; 79:618–625. doi: 10.1136/annrheumdis-2019-216823

Post-hoc assessment: effects of differences between regions on primary endpoint

An analysis for mRSS with region by treatment interaction term was performed. Three approximately equal-size regions were defined (Europe/Australia/New Zealand, North America, East Asia/Japan). Numerically, Europe/Australia/New Zealand had lower changes in both treatment groups, but no statistically significant differences were found (supplementary table S7). We concluded that the observed differences between regions do not explain the non-significant primary endpoint.

Supplementary Table S7. Mixed Model Repeated Measures (Method #1) for Change from Baseline to Week 52 in mRSS, Including Treatment by Region Interaction (Full Analysis Set), Least Square Means

Treatment	Interaction term	LS mean of change	Standard error of change	95% CI for change
Riociguat	North America	−2.45	1.41	(−5.28, 0.38)
	Europe /Australia/New Zealand	−0.65	0.89	(−2.44, 1.14)
	East Asia/Japan	−2.21	1.70	(−5.62, 1.20)
Placebo	North America	1.13	1.74	(−2.35, 4.62)
	Europe/Australia/New Zealand	0.04	1.15	(−2.26, 2.33)
	East Asia/Japan	1.02	2.05	(−3.07, 5.10)

For the statistical evaluation, a MMRM model was applied with baseline value, treatment group, region, visit, treatment by visit and treatment by region as fixed effects, and subject as a random effect. Method #1: all observations are used. CI, confidence intervals; LS, least squares; MMRM, mixed model repeated measures; mRSS, modified Rodnan skin score.

Post-hoc assessment: effects of discontinuation on primary endpoint

To investigate whether the high discontinuation rate may have contributed to the failure to reach statistical significance, we performed, as a sensitivity analysis, tipping point analysis and pattern-mixture modelling to evaluate whether missing values had a large impact on the results. Those analyses were consistent with the primary one. The numbers of drop-outs were similar in both groups. It therefore seems unlikely that the high discontinuation rate contributed to the lack of a significant effect on mRSS.

Supplementary Table S8. Summary of Adverse Events

Patients Reporting Event, n (%)		
**Event**	**Riociguat (n=60)**	**Placebo (n=61)**
Any AE	58 (96.7)	55 (90.2)
Any study drug-related AE	40 (66.7)	29 (47.5)
Any AE related to procedures required by the protocol	4 (6.7)	2 (3.3)
Maximum intensity for any AE		
Mild	17 (28.3)	21 (34.4)
Moderate	35 (58.3)	24 (39.3)
Severe	6 (10.0)	10 (16.4)
Maximum intensity for study drug-related AE		
Mild	25 (41.7)	18 (29.5)
Moderate	15 (25.0)	8 (13.1)
Severe	0	3 (4.9)
Discontinuation of study drug due to AE	11 (18.3)	11 (18.0)
Most common AEs		
Gastroesophageal reflux disease	15 (25.0)	7 (11.5)
Dizziness	13 (21.7)	7 (11.5)
Arthralgia	12 (20.0)	8 (13.1)
Headache	11 (18.3)	12 (19.7)
Diarrhoea	10 (16.7)	8 (13.1)
Cough	9 (15.0)	5 (8.2)
Vomiting	8 (13.3)	6 (9.8)
Dyspnoea	8 (13.3)	5 (8.2)
Palpitations	8 (13.3)	3 (4.9)
Nausea	7 (11.7)	7 (11.5)
Fatigue	7 (11.7)	6 (9.8)
Hypotension	7 (11.7)	4 (6.6)

Khanna D, *et al. Ann Rheum Dis* 2020; 79:618–625. doi: 10.1136/annrheumdis-2019-216823

Dyspepsia	7 (11.7)	2 (3.3)
Peripheral oedema	6 (10.0)	2 (3.3)
Dysphagia	6 (10.0)	1 (1.6)
Skin ulcer	4 (6.7)	8 (13.1)
Upper respiratory tract infection	4 (6.7)	8 (13.1)
Any AE of special interest	7 (11.7)	6 (9.8)
Serious haemoptysis	0	0
Symptomatic hypotension*	7 (11.7)	6 (9.8)

Table shows AEs reported in ≥10% of patients in either group, and all AEs of special interest.

*This included any patients in whom symptoms, eg, headache or dizziness, were reported as hypotension in the case report form.

AE, adverse event.

MedDRA preferred terms are shown.

Khanna D, *et al. Ann Rheum Dis* 2020; 79:618–625. doi: 10.1136/annrheumdis-2019-216823

Supplementary Table S9. AEs Leading to Discontinuation of Study Drug

Patients Reporting Event, n (%)		
**Event**	**Riociguat (n=60)**	**Placebo (n=61)**
Any AE leading to discontinuation	11 (18.3)	11 (18.0)
Angina pectoris	0	1 (1.6)
Left ventricular failure	0	1 (1.6)
Upper abdominal pain	1 (1.7)	0
Diarrhoea	1 (1.7)	0
Dysphagia	1 (1.7)	0
Haematochezia	1 (1.7)	0
Intestinal pseudo-obstruction	1 (1.7)	0
Vomiting	1 (1.7)	0
Exposure during pregnancy	0	1 (1.6)
Aspartate aminotransferase increased	0	1 (1.6)
Blood creatinine phosphokinase increased	1 (1.7)	0
C-reactive protein increased	0	1 (1.6)
Liver function test increased	0	1 (1.6)
Pulmonary function test decreased	1 (1.7)	0
Eosinophilic fasciitis	1 (1.7)	0
Muscular weakness	1 (1.7)	0
Musculoskeletal pain	0	1 (1.6)
Myositis	0	1 (1.6)
Systemic scleroderma	1 (1.7)	0
Acute myeloid leukaemia	0	1 (1.6)
Intraductal proliferative breast lesion	1 (1.7)	0
Ovarian cancer	0	1 (1.6)
Cerebellar infarction	0	1 (1.6)
Scleroderma renal crisis	0	1 (1.6)

Khanna D, *et al. Ann Rheum Dis* 2020; 79:618–625. doi: 10.1136/annrheumdis-2019-216823

Interstitial lung disease	1 (1.7)	0

AE, adverse event.

MedDRA preferred terms are shown.

Khanna D, *et al. Ann Rheum Dis* 2020; 79:618–625. doi: 10.1136/annrheumdis-2019-216823

Supplementary Table S10. Summary of Adverse Events According to Presence or Absence of ILD by Medical History

Patients Reporting Event, n *(%)*				
	**Riociguat Group**		**Placebo Group**	
**Event**	With ILD (n=12)	Without ILD (n=48)	With ILD (n=13)	Without ILD (n=48)
Any adverse event	10 (83.3)	48 (100.0)	12 (92.3)	43 (89.6)
Dizziness	4 (33.3)	9 (18.8)	3 (23.1)	4 (8.3)
Gastro-oesophageal reflux disease	3 (25.0)	12 (25.0)	1 (7.7)	5 (10.4)
Diarrhoea	2 (16.7)	8 (16.7)	1 (7.7)	7 (14.6)
Palpitations	2 (16.7)	6 (12.5)	2 (15.4)	1 (2.1)
Dyspepsia	2 (16.7)	5 (10.4)	0	2 (4.2)
Pain	2 (16.7)	1 (2.1)	0	0
Arthralgia	1 (8.3)	11 (22.9)	2 (15.4)	5 (10.4)
Headache	1 (8.3)	10 (20.8)	3 (23.1)	8 (16.7)
Dyspnoea	1 (8.3)	7 (14.6)	1 (7.7)	4 (8.3)
Cough	1 (8.3)	7 (14.6)	0	5 (10.4)
Fatigue	1 (8.3)	6 (12.5)	2 (15.4)	4 (8.3)
Vomiting	1 (8.3)	6 (12.5)	2 (15.4)	4 (8.3)
Dysphagia	1 (8.3)	5 (10.4)	0	1 (2.1)
Nasopharyngitis	1 (8.3)	4 (8.3)	0	5 (10.4)
Hypotension	0	7 (14.6)	1 (7.7)	3 (6.3)
Nausea	0	6 (12.5)	3 (23.1)	4 (8.3)
Peripheral oedema	0	6 (12.5)	1 (7.7)	1 (2.1)
Peripheral swelling	0	5 (10.4)	1 (7.7)	3 (6.3)
Upper respiratory tract infection	0	4 (8.3)	2 (15.4)	6 (12.5)
Pruritus	0	4 (8.3)	2 (15.4)	3 (6.3)
Urinary tract infection	0	4 (8.3)	2 (15.4)	0

Khanna D, *et al. Ann Rheum Dis* 2020; 79:618–625. doi: 10.1136/annrheumdis-2019-216823

Skin ulcer	0	4 (8.3)	1 (7.7)	7 (14.6)
Abdominal pain	0	3 (6.3)	0	5 (10.4)
Pyrexia	0	2 (4.2)	2 (15.4)	2 (4.2)
Hypertension	0	0	3 (23.1)	1 (2.1)
Sjögren’s syndrome	0	0	2 (15.4)	1 (2.1)

ILD was identified by medical history at baseline.

Table shows adverse events reported in ⩾10% of patients in any group.

One patient (8.3%) with ILD receiving riociguat experienced an SAE (pneumonia). Three patients (23.1%) with ILD receiving placebo experienced an SAE. The SAEs reported were angina pectoris, pericarditis, ventricular tachycardia, gastric haemorrhage, osteolysis, gastric adenocarcinoma and syncope, each in 1 patient (7.7%) (some patients experienced >1 SAE).

Khanna D, *et al. Ann Rheum Dis* 2020; 79:618–625. doi: 10.1136/annrheumdis-2019-216823

Supplementary Table S11 Summary of Adverse Events According to Presence or Absence of ILD

Defined by FVC% 50−75% at Baseline

Patients Reporting Event, n *(%)*				
	**Riociguat Group**		**Placebo Group**	
**Event**	With ILD (n=11)	Without ILD (n=49)	With ILD (n=7)	Without ILD (n=54)
Any adverse event	11 (100)	47 (95.9)	5 (71.4)	50 (92.6)
Dizziness	3 (27.3)	10 (20.4)	1 (14.3)	6 (11.1)
Arthralgia	3 (27.3)	9 (18.4)	2 (28.6)	6 (11.1)
Gastro-oesophageal reflux disease	2 (18.2)	13 (26.5)	2 (28.6)	5 (9.3)
Headache	2 (18.2)	9 (18.4)	2 (28.6)	10 (18.5)
Vomiting	2 (18.2)	6 (12.2)	1 (14.3)	5 (9.3)
Dysphagia	2 (18.2)	4 (8.2)	0	1 (1.9)
Interstitial lung disease	2 (18.2)	2 (4.1)	0	2 (3.7)
Abdominal pain	2 (18.2)	1 (2.0)	0	5 (9.3)
Conjunctivitis	2 (18.2)	0	0	0
Dyspnoea	1 (9.1)	7 (14.3)	1 (14.3)	4 (7.4)
Dyspepsia	1 (9.1)	6 (12.2)	0	2 (3.7)
Fatigue	1 (9.1)	6 (12.2)	2 (28.6)	4 (7.4)
Hypotension	1 (9.1)	6 (12.2)	0	4 (7.4)
Nausea	1 (9.1)	6 (12.2)	1 (14.3)	6 (11.1)
Peripheral oedema	1 (9.1)	5 (10.2)	0	2 (3.7)
Pruritus	1 (9.1)	4 (8.2)	2 (28.6)	3 (5.6)
Insomnia	1 (9.1)	2 (4.1)	1 (14.3)	1 (1.9)
Blood creatine phosphokinase increased	1 (9.1)	1 (2.0)	1 (14.3)	0
Paraesthesia	1 (9.1)	0	1 (14.3)	3 (5.6)
Anxiety	1 (9.1)	0	1 (14.3)	1 (1.9)

Khanna D, *et al. Ann Rheum Dis* 2020; 79:618–625. doi: 10.1136/annrheumdis-2019-216823

Muscular weakness	1 (9.1)	0	1 (14.3)	0
Diarrhoea	0	10 (20.4)	1 (14.3)	7 (13.0)
Cough	0	9 (18.4)	0	5 (9.3)
Palpitations	0	8 (16.3)	1 (14.3)	2 (3.7)
Nasopharyngitis	0	5 (10.2)	1 (14.3)	4 (7.4)
Peripheral swelling	0	5 (10.2)	1 (14.3)	4 (7.4)
Upper respiratory tract infection	0	4 (8.2)	1 (14.3)	7 (13.0)
Pain in extremity	0	4 (8.2)	1 (14.3)	1 (1.9)
Urinary tract infection	0	4 (8.2)	1 (14.3)	1 (1.9)
Constipation	0	3 (6.1)	1 (14.3)	3 (5.6)
Pyrexia	0	2 (4.1)	1 (14.3)	3 (5.6)
Musculoskeletal pain	0	2 (4.1)	1 (14.3)	2 (3.7)
Upper abdominal pain	0	2 (4.1)	1 (14.3)	1 (1.9)
Hot flush	0	2 (4.1)	1 (14.3)	0
Decreased appetite	0	1 (2.0)	1 (14.3)	2 (3.7)
Fall	0	1 (2.0)	1 (14.3)	2 (3.7)
Exertional dyspnoea	0	1 (2.0)	1 (14.3)	1 (1.9)
Pulmonary function test decreased	0	1 (2.0)	1 (14.3)	0
Weight increased	0	1 (2.0)	1 (14.3)	0
Sjögren's syndrome	0	0	2 (28.6)	1 (1.9)
Hypertension	0	0	1 (14.3)	3 (5.6)
Depression	0	0	1 (14.3)	2 (3.7)
Weight decreased	0	0	1 (14.3)	2 (3.7)
Myositis	0	0	1 (14.3)	1 (1.9)
Pericarditis	0	0	1 (14.3)	1 (1.9)
Skin tightness	0	0	1 (14.3)	1 (1.9)
Arrhythmia	0	0	1 (14.3)	0
Ventricular tachycardia	0	0	1 (14.3)	0

Khanna D, *et al. Ann Rheum Dis* 2020; 79:618–625. doi: 10.1136/annrheumdis-2019-216823

Blepharitis	0	0	1 (14.3)	0
Cellulitis	0	0	1 (14.3)	0
Dermatomyositis	0	0	1 (14.3)	0
Increased upper airway secretion	0	0	1 (14.3)	0
Lip injury	0	0	1 (14.3)	0
Liver function test increased	0	0	1 (14.3)	0
Lower gastrointestinal haemorrhage	0	0	1 (14.3)	0
Peripheral neuropathy	0	0	1 (14.3)	0
Skin lesion	0	0	1 (14.3)	0
Syncope	0	0	1 (14.3)	0

Table shows adverse events reported in ⩾10% of patients in any group.

FVC%, forced vital capacity per cent predicted; ILD, interstitial lung disease.

Two patients (18.2%) with ILD receiving riociguat experienced an SAE: abdominal pain and intraductal proliferative breast lesion, each reported in 1 patient (9.1%). One patient (14.3%) with ILD receiving placebo experienced a total of three SAEs: pericarditis, syncope and ventricular tachycardia (incidence of each event: 14.3%).

Khanna D, *et al. Ann Rheum Dis* 2020; 79:618–625. doi: 10.1136/annrheumdis-2019-216823
