# Supplementary material for: Riociguat in patients with early diffuse cutaneous systemic sclerosis (RISE-SSc): randomised, double-blind, placebo-controlled multicentre trial
Source: Ann Rheum Dis. 2020 Apr 15;79(5):618–25. doi: 10.1136/annrheumdis-2019-216823 (PMC7213318; doi:10.1136/annrheumdis-2019-216823)
Supplement: Supplementary data [file annrheumdis-2019-216823supp002.pdf]

**Document Type:**	Study Protocol
**Official Title:**	A Randomized, Double-Blind, Placebo-Controlled Phase II Study to Investigate the Efficacy and Safety of Riociguat in Patients With Diffuse Cutaneous Systemic Sclerosis (dcSSc)
**NCT Number:**	NCT02283762
**Document Date:**	17 Apr 2018

A Randomized, Double-Blind, Placebo-Controlled Phase II Study to Investigate the Efficacy and Safety of Riociguat in Patients With Diffuse Cutaneous Systemic Sclerosis (dcSSc)

This protocol version is an integration of the following documents / sections:

- **Original protocol,** Version 1.0, dated 04 SEP 2014
- **Amendment no. 1** (described in Section 13.1)
  forming integrated protocol Version 2.0, dated 04 FEB 2015
- **Amendment no. 4** (described in Section 13.2)
  forming integrated protocol Version 3.0, dated 24 FEB 2016
- **Amendment no. 5** (described in Section 13.3)
  forming integrated protocol Version 4.0, dated 31 AUG 2016
- **Amendment no. 6** (described in Section 13.4)
  forming integrated protocol Version 5.0, dated 02 MAY 2017

  Amendment no. 7 (described in Section13.5)
  forming integrated protocol Version 6.0, dated 17 APR 2018

This protocol has also been amended for specific countries:

- **Amendment no 2** (UK), dated 29 JAN 2015 (not submitted).
- **Amendment no 3** (UK), dated 12 MAR 2015

These local amendments do not form part of this integrated global protocol.

Title page - amended

A Randomized, Double-Blind, Placebo-Controlled Phase II Study to Investigate the Efficacy and Safety of Riociguat in Patients With Diffuse Cutaneous Systemic Sclerosis (dcSSc)

Riociguat in diffuse cutaneous systemic sclerosis (dcSSc)

Test drug:	BAY63-2521 / Riociguat		
Study purpose:	Safety and efficacy		
Clinical study phase:	II	Date:	17 APR 2018
EudraCT no.:	2014-001353-16	Version no.:	6.0
Study no.:	BAY 63-2521 / 16277		

Sponsor[1]: **Non-US: Bayer AG, D-51368 Leverkusen, Germany**
US territory: Bayer Healthcare Pharmaceuticals Inc., 100 Bayer Boulevard, P.O. Box 915, Whippany NJ 07981-0915, USA

Sponsor's medical expert[2]: PPD
Bayer Healthcare Company Ltd
Bayer Center,
No.27, Dong San Huan North Road, Chaoyang District,
Beijing, 100020 , China
Tel: PPD
Fax: PPD

The study will be conducted in compliance with the protocol, ICH-GCP and any applicable regulatory requirements.

Confidential
The information provided in this document is strictly confidential and is intended solely for the guidance of the clinical investigation. Reproduction or disclosure of this document - whether in part or in full - to parties not associated with the clinical investigation, or its use for any other purpose, without the prior written consent of the sponsor is not permitted.

Throughout this document, symbols indicating proprietary names (®, TM) may not be displayed. Hence, the appearance of product names without these symbols does not imply that these names are not protected.

[1] Per Amendment 5
[2] Per Amendment 1

Signature of the sponsor's medically responsible person

The signatory agrees to the content of the final clinical study protocol as presented.

Name: PPD Role: Global Clinical Lead

Date: ______________________ Signature: ______________________

Signature of principal investigator

The signatory agrees to the content of the final clinical study protocol as presented.

Name:

Date: ______________________ Signature: ______________________

Signed copies of this signature page are stored in the sponsor's study file and in the respective center's investigator site file.

Synopsis - amended

**Title**	A Randomized, Double-Blind, Placebo-Controlled Phase II Study to Investigate the Efficacy and Safety of Riociguat in Patients With Diffuse Cutaneous Systemic Sclerosis (dcSSc)
**Short title**	Riociguat in diffuse cutaneous systemic sclerosis (dcSSc)
**Clinical study phase**	II
**Study objective(s)**	To evaluate the efficacy and safety of 52 weeks of treatment with riociguat versus placebo in patients with diffuse cutaneous systemic sclerosis (dcSSc)
**Test drug(s)**	BAY 63-2521 / Riociguat
**Name of active ingredient**	BAY 63-2521 / Riociguat
**Dose(s)**	0.5 mg, 1 mg, 1.5 mg, 2 mg and 2.5 mg administered 3 times a day (TID); dose titration starting with 0.5 mg (planned up-titration every 2 weeks)
**Route of administration**	Oral
**Duration of treatment**	Total of 52 weeks, consisting of a 10-week titration period and 42 weeks of maintenance.
**Reference drug(s)**	Placebo
**Name of active ingredient**	Not applicable
**Dose(s)**	Matching placebo tablets to BAY 63-2521 / Riociguat 0.5 mg, 1 mg, 1.5 mg, 2 mg and 2.5 mg administered TID; dose titration starting with 0.5 mg matching placebo tablet.
**Route of administration**	Oral
**Duration of treatment**	52 weeks
**Background treatment**	Not applicable
**Indication**	dcSSc

**Diagnosis and main criteria for inclusion**	Men or women aged 18 years and older<br>Disease duration of ≤ 18 months (defined as time from the first non−Raynaud's phenomenon manifestation), SSc as defined by 2013 the American College of Rheumatology (ACR) / European League Against Rheumatism (EULAR) classification, and presence of dcSSc based on Leroy criteria<br>≥ 10 and ≤ 22 modified Rodnan skin score (mRSS) units at the screening visit<br>Forced vital capacity (FVC) ≥ 45% of predicted at screening<br>Diffusion capacity of the lung for carbon monoxide (DL_{CO}) ≥ 40% (hemoglobin-corrected) of predicted at screening<br>Women of childbearing potential must have a negative serum pregnancy test at the screening visit.
**Study design**	Randomized (1:1), double-blind, placebo-controlled, parallel-group, multicenter, multinational study
**Methodology**	This study is designed to investigate the efficacy and safety of riociguat (0.5 mg, 1 mg, 1.5 mg, 2 mg and 2.5 mg TID) in patients with dcSSc. The study consists of 2 parts: a main treatment phase for efficacy and safety (placebo-controlled) and a long-term extension phase for collection of exploratory long-term outcomes.
**Type of control**	Placebo
**Number of patients**	Up to approximately 130 randomized patients[3]
**Primary variable**	Change in mRSS from baseline to Week 52
**Plan for statistical analysis**	The change in mRSS from baseline to all assessments post baseline up to Week 52 will be analyzed using mixed model repeated measures (MMRM) with baseline mRSS as a covariate, fixed effects treatment arm and region, the interaction effect between study visit and treatment arm, and patient-specific random effects. The main estimand of interest is the treatment difference between the riociguat and placebo treatment arms at Week 52.

[3] Per Amendment 5

Protocol amendment summary of changes table

Amendment no. 7 (17 APR 2018)

This amendment is considered to be non-substantial based on the criteria set forth in Article 10(a) of Directive 2001/20/EC of the European Parliament and the Council of the European Union because it neither significantly impacts the safety or physical/mental integrity of participants nor the scientific value of the study.

Overall rationale for the amendment

Overall rationale: The protocol amendment 7 was prepared based on the main treatment phase results of the study to better characterize the enrolled patient population and their autoantibody profile.

This change does not affect the design or the overall concept of the study.

In addition, a change of responsible Global Clinical Lead was made.

Section # and name	Description of change	Brief rationale
Signature of the sponsor's medically responsible person	The Global Clinical Lead was updated to PPD	Change of responsible Global Clinical Lead
7.6.4 Autoantibody screen	The addition of the anti-centromere was included in the autoantibody screen.	Further autoantibodies will allow better characterization of the enrolled patient population and their autoantibody profile.

Table of contents

Title page - amended 2

Signature of the sponsor's medically responsible person 3

Signature of principal investigator 4

Synopsis - amended 5

Protocol amendment summary of changes table 7

Table of contents 8

Table of in-text tables 12

Table of in-text figures 12

List of abbreviations 13

1. Introduction 16
1.1 Background 16
1.1.1 Scleroderma (systemic sclerosis) 16
1.1.2 Riociguat 17
1.1.2.1 Mechanism of Action 17
1.1.2.2 Preclinical Data - amended 18
1.1.2.3 Clinical Data 19
1.2 Rationale of the study 19
1.3 Benefit-risk assessment - amended 20

2. Study objectives - amended 21

3. Investigators and other study personnel - amended 22

4. Study design 24
4.1 Design overview -amended 24
4.1.1 Screening phase (up to 2 weeks) 26
4.1.2 Main treatment phase (Week 0 to Week 52) - amended 26
4.1.3 Long-term extension phase 27
4.1.4 Dose Interruptions - amended 28
4.1.5 Termination visit and safety follow-up visit - amended 29
4.2 Primary variable 29
4.3 Justification of the design 29
4.4 End of study 30

5. Study population 30
5.1 Eligibility 30
5.1.1 Inclusion criteria - amended 30
5.1.2 Exclusion Criteria - amended 30
5.1.3 Justification of selection criteria 32
5.2 Withdrawal of patients from study - amended 33
5.2.1 Withdrawal - amended 33
5.2.1.1 Withdrawal from the study 33
5.2.1.2 Discontinuation of study medication 33
5.2.1.3 Handling of withdrawals or discontinuations from study medication 34
5.2.2 Replacement - amended 35

5.3 Patient identification ... 36

6. Treatment ... 36
6.1 Treatments to be administered ... 36
6.2 Identity of study treatment ... 36
6.3 Treatment assignment ... 37
6.4 Dosage and administration ... 38
6.4.1 Selection of doses in the study - amended ... 38
6.5 Blinding - amended ... 38
6.6 Drug logistics and accountability ... 39
6.7 Treatment compliance ... 39
6.8 Post-study therapy ... 40
6.9 Prior and concomitant therapy - amended ... 40

7. Procedures and variables ... 43
7.1 Schedule of procedures ... 43
7.1.1 Tabulated overview - amended ... 44
7.1.2 Timing of assessments ... 49
7.1.2.1 Visit 0 – Screening phase – Screening visit ... 49
7.1.2.2 Visit 1 – Main treatment phase - Baseline (Day 0, Week 0) ... 50
7.1.2.3 Visits 2 through to 6 – Main treatment phase – Dose-titration period - amended ... 51
7.1.2.4 Visits 7 through to 11 – Main treatment phase - Maintenance period - amended ... 52
7.1.2.5 Visit 12 (Week 52) - amended ... 53
7.1.2.6 Visits 13 through to 17 – Long-term extension phase - Dose-titration period - amended ... 55
7.1.2.7 Visits 18 through Visit n (every 12 weeks) – Long-term extension phase – Open-label extension period - amended ... 55
7.1.2.8 Termination Visit - amended ... 56
7.1.2.9 Safety follow-up visit - amended ... 57
7.1.2.10 Premature discontinuation of study drug treatment - amended ... 58
7.2 Population characteristics ... 58
7.2.1 Demographic ... 58
7.2.2 Medical history ... 58
7.3 Efficacy - amended ... 58
7.4 Pharmacokinetics / pharmacodynamics - amended ... 60
7.5 Safety ... 62
7.5.1 Adverse events ... 62
7.5.1.1 Definitions - amended ... 62
7.5.1.2 Classifications for adverse event assessment ... 63
7.5.1.2.1 Seriousness ... 63
7.5.1.2.2 Intensity ... 63
7.5.1.2.3 Causal relationship ... 63
7.5.1.2.4 Action taken with study treatment ... 65
7.5.1.2.5 Other specific treatment(s) of adverse events ... 65
7.5.1.2.6 Outcome ... 65
7.5.1.3 Assessments and documentation of adverse events ... 65
7.5.1.4 Reporting of serious adverse events ... 65
7.5.1.5 Expected adverse events ... 66
7.5.1.6 Adverse events of special safety interest ... 67

7.5.1.7 Overdose of Study Medication 67
7.5.2 Pregnancies 67
7.6 Other procedures and variables 67
7.6.1 Pregnancy testing 67
7.6.2 Laboratory parameters - amended 68
7.6.3 Exploratory Biomarkers - amended 69
7.6.4 Autoantibody screen 70
7.6.5 Blood pressure and heart rate measurement - amended 70
7.6.6 Modified Rodnan skin scoring (mRSS) 70
7.6.7 Electrocardiogram 71
7.6.8 Pulmonary function testing 71
7.6.9 Skin biopsy 71
7.6.10 Digital ulcer net burden 72
7.6.11 Digital gangrene 72
7.6.12 Raynaud's attacks assessment 73
7.6.13 Patient-reported outcomes (PROs) / Health-related quality of life (HRQoL) questionnaires 73
7.6.14 Tender and swollen joint count 74
7.6.15 Patient's and physician's global assessment - amended 74
7.6.16 Patient interference with skin assessment 75
7.6.17 Tendon friction rubs 75
7.6.18 Oxygen saturation measurement using forehead pulse oximetry 75
7.7 Appropriateness of procedures / measurements 75

8. Statistical methods and determination of sample size 75
8.1 General considerations 75
8.2 Analysis sets - amended 76
8.3 Variables 76
8.4 Statistical and analytical plans 76
8.4.1 Demographic and other baseline characteristics 76
8.4.2 Efficacy - amended 76
8.4.2.1 Primary endpoint 76
8.4.2.2 Secondary and exploratory endpoints 78
8.4.3 Safety - amended 80
8.4.4 Pharmacokinetics 81
8.5 Planned interim analyses - amended 81
8.6 Determination of sample size - amended 81

9. Data handling and quality assurance 83
9.1 Data recording 83
9.2 Monitoring 83
9.3 Data processing 84
9.4 Audit and inspection 84
9.5 Archiving 84

10. Premature termination of the study 85

11. Ethical and legal aspects 85
11.1 Ethical and legal conduct of the study 85
11.2 Patient information and consent 86
11.3 Publication policy 87

11.4 Compensation for health damage of patients / insurance 87
11.5 Confidentiality 87

12. Reference list - amended, ... 89

13. Protocol amendments 93
13.1 Amendment 1 93
13.1.1 Overview of changes to the study 93
13.1.2 Changes to the protocol text 93
13.2 Amendment 4 117
13.2.1 Overview of changes to the study 117
13.2.2 Changes to the protocol text 117
13.3 Amendment 5 132
13.3.1 Overview of changes to the study 132
13.3.2 Changes to the protocol text 133
13.4 Amendment 6 146
13.4.1 Overview of changes to the study 146
13.4.2 Changes to the protocol text 146
13.5 Amendment 7 164

14. Appendices 165
14.1 Child-Pugh Classification of Liver Disease 165
14.2 Definition of Systemic Sclerosis Renal Crisis - amended 166
14.3 Tender and Swollen Joint Count Assessment Form 167

Table of in-text tables

Table 6–1 Identity of investigational product 37
Table 6–2 Prohibited prior and concomitant therapy - amended 41
Table 7–1 Schedule of evaluations - amended 44
Table 7–2 Profile Day PK Sampling (Visit 1 and Visit 12) 48
Table 7–3 PK Sampling (Visits 2 – 6 and Visits 13 – 17) 48
Table 7–4 PK Sampling (Visit 11) 49
Table 8–1 References for estimate of variability 82
Table 8–2 Power calculations: power varying from 70% to 80% in 2% stages 82

Table of in-text figures

Figure 4–1 Main treatment phase design 25
Figure 4–2 Long-term extension phase design 25

List of abbreviations

ACR	American College of Rheumatology
ADMA	asymmetrical dimethylarginine
AE	adverse event
ALT	alanine aminotransferase (also known as SGPT, qv)
ANCOVA	analysis of covariance
AP	alkaline phosphatase
aPTT	activated partial thromboplastin time
AST	aspartate aminotransferase (also known as SGOT, qv)
ATC	Anatomical Therapeutic Chemical
AUC	area under the plasma concentration versus time curve
BMI	body mass index
BP	blood pressure
BPM	beats per minute
cGMP	cyclic guanosine monophosphate
CK	creatine kinase
C_{max}	maximum plasma concentration
COL1A1	collagen type 1 alpha 1
COL1A2	collagen type 1 alpha 2
COL3A1	collagen type 3 alpha 1
COMP	cartilage oligomeric matrix protein
CREST	calcinosis, Raynaud's phenomenon, esophageal dysmotility, sclerodactyly, and telangiectasias
CRISS	(American College of Rheumatology) Composite Response Index for Clinical Trials
CRO	clinical research organization
CSRG	Canadian Scleroderma Research Group
CTEPH	chronic thromboembolic pulmonary hypertension
CXCL-4	platelet factor-4
CYP	cytochrome P450
DBP	diastolic blood pressure
dcSSc	diffuse cutaneous systemic sclerosis
dL	Deciliter
DL_{CO}	diffusion capacity of the lung for carbon monoxide
DMC	data monitoring committee
EC	Ethics Committee
ECG	Electrocardiogram
eCRF	electronic case report form
eg	*exempli gratia*, for example
eGFR	estimated glomerular filtration rate
EU	European Union
EULAR	European League Against Rheumatism
EUSTAR	EULAR Scleroderma Trials and Research
FAS	full analysis set
FDA	Food and Drug Administration
FVC	forced vital capacity
g	Gram

GCP	Good Clinical Practice
GGT	gamma glutamyl transpeptidase
GLDH	Glutamate dehydrogenase
GMP	Good Manufacturing Practice
h	Hour
HAQ-DI	Health Assessment Questionnaire disability index
HDPE	high density polyethylene
HPF	High power field
HR	heart rate
HRCT	High resolution computed tomography
HRQoL	health-related quality of life
hsCRP	high-sensitivity C-reactive protein
IB	investigator's brochure
ICH	International Conference on Harmonization
ie	*id est,* that is
IEC	independent ethics committee
IHC	Immunohistochemistry
ILD	Interstitial lung disease
IND	Investigational New Drug
INR	international normalized ratio (prothrombin time expressed in relation to normal value)
IRB	institutional review board
ITT	intent to treat
IxRS	telephone-based or web-based response system
L	Liter
lcSSc	limited cutaneous systemic sclerosis
LPLV	Last Patient Last Visit
m^2	square meter
MCP	Metacarpophalangeal joint
MDRD	modification of diet in renal disease
MedDRA	Medical Dictionary for Regulatory Activities
mg	Milligram
min	Minute
mL	Milliliter
mol	Molarity
mmHg	millimeters of mercury
MMRM	mixed model repeated measures
mRNA	messenger ribonucleic acid
mRSS	modified Rodnan skin score
NO	nitric oxide
NSAID	nonsteroidal anti-inflammatory drug
PAH	pulmonary arterial hypertension
PD	pharmacodynamics
PDE	Phosphodiesterase
PDE5	phosphodiesterase 5
p-ERK	phosphorylated extracellular regulated kinase
P-gp	P-glycoprotein
PIP	Proximal interphalangeal joint
PK	pharmacokinetic(s)

popPK	population PK
PPS	per protocol set
PRO	patient-reported outcome(s)
PROMIS	Patient-Reported Outcomes Measurement Information System
p-VASP	phosphorylated vasodilator-stimulated phosphoprotein
RBC	red blood cells
s	Second
SAE	serious adverse event
SAP	statistical analysis plan
SAS	Statistical Analysis System
SBP	systolic blood pressure
SD	standard deviation
SDMA	symmetrical dimethylarginine
sE-Selectin	soluble E-selectin
SF-36	Short Form 36
SFU	safety follow-up
sGC	soluble guanylate cyclase
SHAQ	Scleroderma Health Assessment Questionnaire
SNAP	S-nitroso-N-acetyl-penicillamine
sPECAM-1	soluble platelet endothelial cell adhesion molecule
SpO2	peripheral oxygen saturation
SSc	systemic sclerosis
SUSAR	suspected unexpected serious adverse reaction
TEAEs	treatment-emergent AEs
TGF-β	transforming growth factor-beta
TID	*ter in die*, 3 times a day
TSP1	thrombospondin 1
TV	termination visit
UCLA SCTC GIT	University of California, Los Angeles, Scleroderma Clinical Trial Consortium Gastrointestinal Scale
UK	United Kingdom
ULN	Upper limit of normal
US/ USA	United States/ United States of America
VAS	visual analog scale
WBC	white blood cell
WHO-DD	World Health Organization Drug Dictionary

1. Introduction

1.1 Background

1.1.1 Scleroderma (systemic sclerosis)

Systemic sclerosis (SSc) is a rare, orphan disease featuring chronic, fibrosing, autoimmune responses characterized by small vessel vasculopathy, autoantibody production, and fibroblast dysfunction leading to increased deposition of extracellular matrix.[1]

Systemic sclerosis is further divided into 2 subtypes defined by the extent of skin involvement: limited cutaneous systemic sclerosis (lcSSc) and diffuse cutaneous systemic sclerosis (dcSSc).[2] According to LeRoy and colleagues, classification as limited or diffuse disease is based on the extent of skin tightening.[3] In limited disease (formerly called CREST [calcinosis, Raynaud's phenomenon, esophageal dysmotility, sclerodactyly, and telangiectasias] syndrome), skin tightening is confined to the fingers, hands, and forearms distal to the elbows, with or without tightening of skin of the feet and of the legs distal to the knees. Proximal extremities and the trunk are not involved. In dcSSc, the skin of the proximal extremities and trunk is also involved. Both dcSSc and lcSSc are associated with internal organ involvement; however, patients with dcSSc are at greater risk for clinically significant major organ dysfunction. Diffuse cutaneous SSc is one of the most fatal rheumatic diseases, and is associated with substantial morbidity and many detrimental effects on health-related quality of life (HRQoL).

Systemic sclerosis is an orphan disease with an estimated prevalence of 50,000 in Europe and $276/10^6$ patients in the United States of America (USA).[4] With a 2010 USA census of 309 million people, there are < 100,000 patients in the USA with SSc. Approximately 60% have limited SSc and 35 to 40% have diffuse SSc.

The tissue fibrosis in SSc is caused by an increased production of extracellular matrix components by activated fibroblasts [5] and affects the skin and various internal organs such as the heart, lungs, kidneys and gastrointestinal tract. Tissue fibrosis contributes significantly to the high morbidity and the increased mortality of SSc.[6] The vascular alterations show a predilection for the small arteries and arterioles. Vascular dysfunction (ie, Raynaud’s phenomenon) is one of the earliest alterations of SSc and may represent the initiating event in its pathogenesis. Severe alterations in small blood vessels of skin and internal organs, including fibrosis and perivascular cellular infiltration with diverse inflammatory cells, are almost always present in SSc.

Survival in SSc is dependent on the extent of internal organ involvement and the disease course.[2] Because internal organ and skin involvement are more extensive and progress faster in dcSSc compared to lcSSc, the average 6-year survival rate is 50% in lcSSc compared to 30% in dcSSc. Twelve-year survival is 50% for lcSSc and 15% for dcSSc.[3] Death is generally attributed to progressive pulmonary fibrosis, pulmonary hypertension, extensive gastrointestinal deterioration, and scleroderma heart disease. Except when pulmonary hypertension develops, limited disease is associated with a better prognosis.

No definitive diagnostic test for SSc is available; therefore, several diagnostic classifications have been developed. Until recently, the standard classification used for diagnosis was the 1980 preliminary criteria for the classification of SSc, developed by the American College of Rheumatology (ACR).[7] Due to concern regarding the sensitivity of the 1980 criteria, as well as advances in knowledge about SSc in the past 30+ years, the ACR and the European

League Against Rheumatism (EULAR) established a committee to provide a joint proposal for new classification criteria for SSc. The goals of the committee were to develop criteria that 1) encompass a broader spectrum of SSc, including patients whose disease is in the early stage as well as those in the late stage; 2) include vascular, immunologic, and fibrotic manifestations; 3) are feasible to use in daily clinical practice; and 4) are in accordance with criteria used for diagnosis of SSc in clinical practice. The updated classification criteria were published in 2013.[1] Using consensus methods, the committee determined that skin thickening of the fingers extending proximal to the metacarpophalangeal joints is sufficient for the patient to be classified as having SSc; if that feature is not present, the following 7 additive items can be used, with varying weights for each: skin thickening of the fingers, fingertip lesions, telangiectasia, abnormal nailfold capillaries, interstitial lung disease or pulmonary arterial hypertension (PAH), Raynaud's phenomenon, and SSc-related autoantibodies.

Currently, no therapy has been proven to reverse the vascular and fibrotic damage in patients with scleroderma.[2] However, due to the high medical need, a number of drugs, such as methotrexate, mycophenolate mofetil, cyclophosphamide, azathioprine, and cyclosporine, are used off-label in an attempt to slow the progression of fibrosis. Current treatment options only target various SSc-related symptoms. No disease-modifying drug is available for SSc. In the European Union (EU), only bosentan is approved "to reduce the number of new digital ulcers in patients with SSc and ongoing digital ulcer disease," thus addressing also only one aspect of the disease.

Based on the positive results of riociguat in patients with PAH and chronic thromboembolic pulmonary hypertension (CTEPH) together with the compound's known anti-proliferative and antifibrotic effects as seen *in vitro* and in animal models, patients with SSc may benefit from treatment with riociguat.

The current study will be the first study testing riociguat in this indication. The efficacy and safety of riociguat in patients with dcSSc will be evaluated.

1.1.2 Riociguat

1.1.2.1 Mechanism of Action

Riociguat is the first-in-class of a new group of compounds, soluble guanylate cyclase (sGC) stimulators. Riociguat directly stimulates sGC, thereby increasing levels of the signaling molecule cyclic guanosine monophosphate (cGMP). The cGMP molecule plays a pivotal role in regulating cellular processes, such as vascular tone, proliferation, fibrosis, and inflammation. Two key features of riociguat include: 1) it directly stimulates sGC independently of nitric oxide (NO), and 2) it sensitizes sGC to low levels of NO. Because of the broad spectrum of cGMP actions and specific mode of action of riociguat, the efficacy of sGC stimulators for the treatment of patients with SSc was investigated. Both, the vascular effects of riociguat and the direct antifibrotic effects could be beneficial for the treatment of patients with SSc. More specifically, it was shown that sGC stimulators decrease collagen production in dermal fibroblasts from SSc patients.[8] In addition, sGC stimulators block fibroblast to myofibroblasts differentiation of dermal fibroblasts.[9] Thus, sGC stimulators could block production of collagen and extracellular matrix components, which may explain, at least in part, the antifibrotic effects of sGC stimulators.

1.1.2.2 Preclinical Data - amended

To support the use of riociguat for treatment of SSc, *in vitro* and *in vivo* investigations were undertaken, providing ample evidence of beneficial effects of cGMP elevation and the potential of sGC stimulators in the treatment of fibrotic diseases.

In vitro evidence for antifibrotic effects of cGMP elevation in the skin, which is a prerequisite to the effects of sGC stimulators for the treatment of SSc, includes the following findings:

- Collagen production was evaluated in human dermal fibroblasts derived from hypertrophic scars as well as from normal cells from the patient's donor site. Collagen production in fibroblasts, regardless of source, was substantially stimulated by TGF-β1 (transforming growth factor-beta) exposure. Increase of cGMP by either the NO donor SNAP (S-nitroso-N-acetyl-penicillamine) or the cGMP analog 8-Bromo-cGMP offset the stimulation of collagen production [10]
- sGC stimulation dose-dependently inhibited collagen mRNA expression and collagen release in dermal fibroblasts from SSc patients.[8,9]

In vivo evidence for anti-fibrotic effects of cGMP elevation in fibrotic diseases includes the following:

- In a mouse model of pulmonary fibrosis, daily administration of riociguat ameliorated pulmonary inflammation and fibrosis and improved survival [11]
- In a rat model for hypertension and organ damage, riociguat significantly ameliorated fibrotic tissue remodeling and degeneration in hearts and kidneys.[12,13]

To further evaluate the treatment potential of riociguat for SSc, riociguat was tested in 2 widely accepted mouse models for SSc, namely the a) bleomycin-induced skin fibrosis model, which resembles an early, inflammatory-driven stage of SSc and b) in the TSK-1 model which resembles a later, non-inflammatory, stage of the SSc.

As also the vascular effects of riociguat could be beneficial for the treatment of patients with SSc, especially with regard to prevention and healing of digital ulcers. To this end, wound healing was analyzed in the TSK-1 skin fibrosis mouse model.

In summary, investigations of the anti-fibrotic efficacy of sGC stimulators riociguat and BAY 41-2272, another sGC compound[4], showed that these agents act dose-dependently:

- reduced collagen production in human dermal fibroblasts
- reduced fibroblast-to-myofibroblast differentiation of human dermal fibroblasts
- prevented TGF-ß-induced skin fibrosis *in vivo*
- prevented skin fibrosis in bleomycin model
- reduced established skin fibrosis in genetic TSK-1 model
- promoted wound healing in TSK-1 mice.

[4] Per Amendment 1

1.1.2.3 Clinical Data

Riociguat was recently approved in a number of regions / countries including the USA, Canada, Switzerland, Australia, Europe, Turkey, South Korea and Japan, for the treatment of 2 forms of pulmonary hypertension: PAH and/or CTEPH.

Further details can be found in the investigator's brochure (IB), which contains comprehensive information on the study drug.

1.2 Rationale of the study

Diffuse cutaneous SSc is one of the most severely incapacitating and life-threatening rheumatic diseases. Fibrosis of the skin and internal organs is a hallmark of the disease and the progression of the fibrosis disrupts tissue architecture and causes organ failure, resulting in high morbidity and mortality among patients with SSc.

Based on preclinical *in vitro* and *in vivo* data demonstrating that riociguat was safe and efficacious in different models of fibrotic diseases including scleroderma, the hypothesis is that riociguat may bring significant clinical benefit to patients with scleroderma by acting not only by improving vascular disease but, most importantly, by its inhibition of biochemical fibrotic pathways.[8] Based on the preclinical data, it is anticipated that riociguat may slow down the progression of the fibrotic disease by acting through TGFβ-mediated pathways and, by its vasoactive properties, decrease the number and severity of Raynaud’s attacks as well as decrease digital ulcer net burden.

This study aims to recruit patients with dcSSc. The rationale is: a) there is a better understanding of the natural history of dcSSc than lcSSc, and b) dcSSc is a subset of SSc in which more rapid progression in disease occurs with worse prognosis, and where there is a high unmet need for effective treatment.

To better design this study with regard to the inclusion criteria, a recent study [14] was taken into consideration and predictive parameters were identified for the progression of skin fibrosis within 1 year in patients with dcSSc. An observational study using the EUSTAR (EULAR Scleroderma Trials and Research) database was assessed. Worsening of skin fibrosis was defined as increase in modified Rodnan skin score (mRSS) > 5 points and ≥ 25% from baseline to the second visit. Inclusion criteria were dcSSc, ACR criteria fulfilled, mRSS ≥ 7 at the baseline visit, valid data for mRSS at the second visit, and available follow up of 12 (±2) months. In the univariate analysis, patients with progressive fibrosis were compared to non-progressors and predictive markers with p<0.2 were included in the logistic regression analysis. The prediction models were then validated in a second cohort.

A total of 637 dcSSc patients were eligible. Univariate analyses identified joint synovitis, short disease duration (≤ 15 months), short disease duration in females/patients without creatine kinase (CK) elevation, low baseline mRSS (≤ 22/51), and absence of esophageal symptoms as potential predictors for progressive skin fibrosis. In the multivariate analysis, by employing combinations of the predictors, 17 models with varying prediction success were generated, allowing cohort enrichment from 9.7% progressive patients in the whole cohort to 44.4% in the optimized enrichment cohort. Using a second validation cohort of 188 dcSSc patients, short disease duration, low baseline mRSS, and joint synovitis were confirmed as independent predictors of progressive skin fibrosis within 1 year showing a 4.5-fold increased prediction success rate.

From the 17 models generated by the multivariate analysis, the combination of 2 predictors, short disease duration, and low baseline mRSS, which allowed for a cohort enrichment of around 20%, was chosen.

1.3 Benefit-risk assessment - amended

Diffuse cutaneous systemic sclerosis is a progressive, rare, orphan disease characterized by vasculopathy (Raynaud's phenomenon, digital ulcers) and progressive fibrosis of the skin and internal organs, ultimately leading to death. There are currently no disease-specific pharmacotherapies approved, and therefore, there is a high unmet medical need in this indication.

Considering the mechanism of action of riociguat and the results of the preclinical studies demonstrating beneficial effects of riociguat in a) decreasing collagen synthesis by dermal fibroblasts, b) inhibiting fibroblast to myofibroblast differentiation, c) decrease in skin thickness in *in vivo* models, and d) improving wound healing in the skin of an animal model, the hypothesis is that riociguat treatment may bring the following benefits to patients with dcSSc:

- Antifibrotic effect, as measured by a treatment effect between *verum* and placebo, as measured by the mRSS and by change from baseline in skin thickness;
- Decrease number and severity of Raynaud's attacks;
- Decrease digital ulcer net burden.

Additional benefits that may occur include reduction in arthralgia and joint tenderness and slowing the progression of internal organ fibrosis.

Relevant risks for the dcSSc population may be unmasking of pulmonary vascular occlusive disease, hypotension, gastrointestinal motility disorders, serious bleeds, and embryo-fetal toxicity.

During the pivotal riociguat PATENT-1 trial for PAH, patients with PAH-SSc were included and the overall benefit-risk profile was positive.

Taking into account the seriousness of the disease as well as the medical need for an effective and safe therapy, on balance the expected benefit to patients with this life-threatening condition outweighs potential risks.

Recently, a potential safety issue has been reported for patients with pulmonary hypertension associated with idiopathic interstitial pneumonias (PH-IIP) and, therefore, the benefit-risk assessment for this indication has changed from positive to unfavorable. Following the data monitoring committee (DMC) recommendation, the study treatment has been terminated. Also per DMC recommendation patients are continuing in a safety follow-up for 4 months after study drug discontinuation and the study is expected to close in September 2016.

A careful review, assessment and analysis will be done by the sponsor with unblinded and clean data originating from clinical study 13605 (RISE-IIP; final clinical study report expected in 2017). Information of health care professionals (via Dear Healthcare Professional Letter), label updates and an update of the risk management plan have been initiated in July 2016 in collaboration with the European Medicines Agency as well as national competent authorities.

The benefit-risk profile for conditions being studied in ongoing clinical studies also has been carefully re-evaluated. In the case of the present study in the dcSSc indication, 2 ad-hoc meetings of the DMC for this study took place, on 23 MAY 2016 and on 25 MAY 2016, followed by a third meeting on 15 AUG 2016. The DMC concluded that this study may continue without amendment or modification. Overall, the benefit-risk balance for this indication remains positive.[5]

2. Study objectives - amended

The overall objectives of this study are to evaluate the efficacy and safety of 52 weeks of treatment with riociguat versus placebo in patients with dcSSc.

The primary objective of this study is:

- To assess the efficacy of riociguat administered 3 times a day (TID) as compared with placebo in terms of change in the mRSS from baseline to Week 52

The secondary objectives of this study are to assess the efficacy of treatment with riociguat administered TID as compared with placebo in terms of:

- Efficacy[6]:
    - Key secondary objective:
        - American College of Rheumatology Combined Response Index for Systemic Sclerosis (CRISS) at Week 52.[7]
    - Further secondary objectives:
        - Health Assessment Questionnaire disability index (HAQ-DI) domain (separately from the Scleroderma Health Assessment Questionnaire [SHAQ] as part of the calculation of the CRISS algorithm)
        - Patient's global assessment
        - Physician's global assessment
        - Change in FVC (forced vital capacity) % predicted
- Further clinical outcomes (to be adjudicated by an Independent Central Adjudication Committee):
    - New renal crisis
    - Worsening of cardiac disease, defined as new or worsened clinically symptomatic and significant heart disease, considered secondary to dcSSc, including congestive heart failure requiring hospitalization, new onset pulmonary hypertension requiring treatment, pericardial disease requiring intervention or exhibiting clinical decompensation, and arrhythmias and/or conduction defects requiring treatment
    - Worsening of gastrointestinal disease requiring hospitalization or new requirement for parenteral nutrition

---

[5] Per Amendment 5
[6] Per Amendment 4
[7] Per Amendment 5

- Critical digital ischemia requiring hospitalization, or digital gangrene

Additional information regarding the events to be adjudicated may be requested to be sent to the Adjudication Committee. All events listed above, in addition to all cases of death, regardless of causality or seriousness will be reviewed by the Adjudication Committee.

3. Investigators and other study personnel - amended

Sponsor's medical expert[8]:

Name: PPD

Address: Bayer Healthcare Company Ltd
Bayer Center,
No.27, Dong San Huan North Road, Chaoyang District,
Beijing, 100020, China
Tel: PPD
Fax: PPD

Coordinating investigators:

Title/Name: PPD

Address: PPD
[redacted] USA

Telephone No: PPD

Fax No: PPD

Title/Name: PPD

Address: PPD
[redacted]
Switzerland

Telephone No: PPD

All other study personnel not included in this section are identified in a separate personnel list (not part of this clinical study protocol) as appropriate. This list will be updated as needed; an abbreviated version with personnel relevant for the centers will be available in each center's investigator site file.

[8] Per Amendment 1

Whenever the term “investigator” is noted in the protocol text, it may refer to either the principal investigator at the site or an appropriately qualified, trained, and delegated individual of the investigative site.

The principal investigator of each center must sign the protocol signature sheet before patient recruitment may start at the respective center. Likewise, all protocol amendments/integrated protocols must be signed and dated by the principal investigator before becoming effective at the respective center.

A complete list of all participating centers and their investigators, as well as all required signature documents, will be maintained in the sponsor study file.

External data evaluation bodies

The independent DMC will be involved in the review of data for safety. Detailed information on the roles and responsibilities of the DMC will be described in the DMC Charter.

Blinded adjudication of further clinical outcomes as described in Section 2 will be performed by an independent Central Adjudication Committee as described in the Adjudication Committee charter.

4. Study design

4.1 Design overview -amended

In this multinational, multicenter, randomized (1:1), double-blind, placebo-controlled, parallel-group study, a total of approximately 200 patients are planned for enrollment in order to randomize up to[9] approximately 130 patients to study drug treatment (approximately 65 patients to the riociguat group and 65 to the placebo group).

The study design consists of a main treatment phase followed by a long-term extension phase as follows (see Figure 4–1 and Figure 4–2):

- **Screening phase:** up to 2 weeks
- **Main treatment phase:** 52 weeks of double-blind treatment, consisting of:
  - Dose titration period of up to 10 weeks
  - Maintenance period of up to 42 weeks
- **Long-term extension phase**
  - Dose-titration period of up to 10 weeks (double-blind)
  - Open-label extension period
- **Termination visit and safety follow-up visit**

[9] Per Amendment 5

Figure 4–1 Main treatment phase design

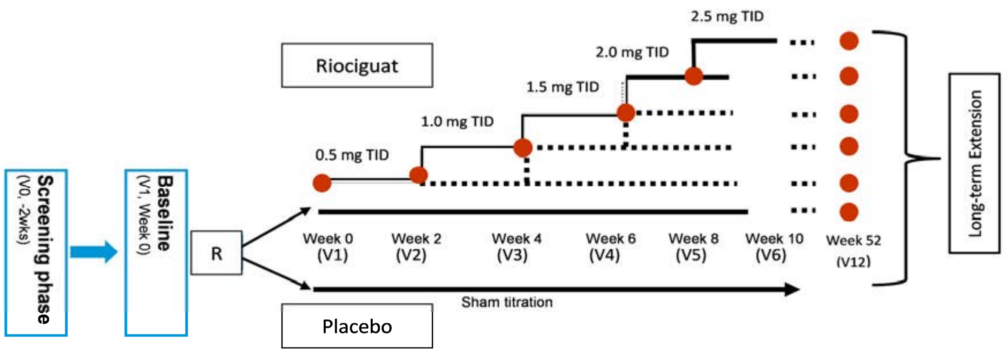

Abbreviations: V = visit; R = randomization; TID = 3 times a day.

Figure 4–2 Long-term extension phase design

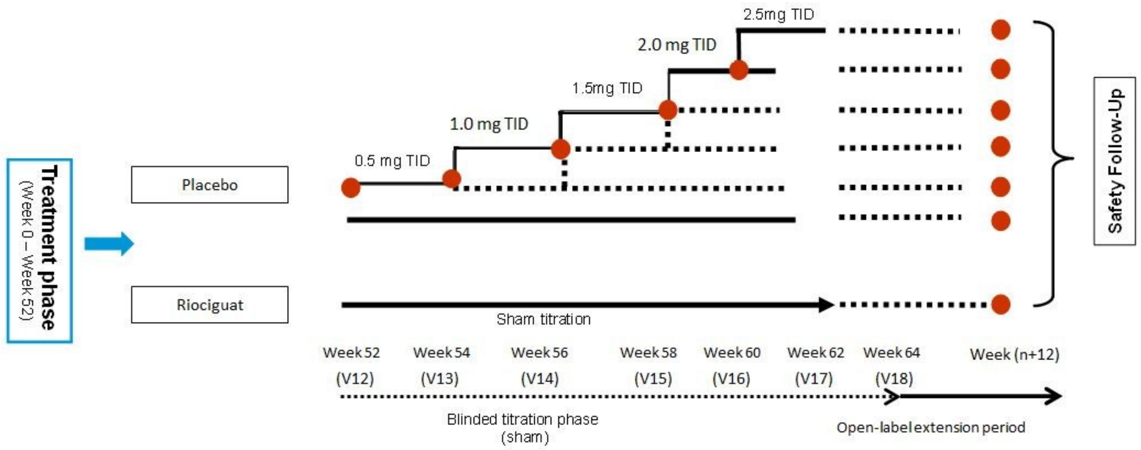

Abbreviations: TID = 3 times a day; V = visit.

4.1.1 Screening phase (up to 2 weeks)

After providing written informed consent, patients will undergo a screening evaluation to determine their eligibility (see Section 7.1 for a detailed schedule of assessments). Patients will complete a diary including details on the number and duration of Raynaud's attacks per day for a period of 7 consecutive days leading up to Visit 1 / Day 0.

4.1.2 Main treatment phase (Week 0 to Week 52) - amended

At the baseline visit, patients who have met all of the inclusion and none of the exclusion criteria will be randomized via a telephone-based or web-based response system (IxRS) to receive treatment with either active study drug (riociguat) or placebo (Day 0, Visit 1).

<u>Dose-titration period (Visits 1 to 6)</u>

During the first 10 weeks of the main treatment phase, patients will undergo dose titration.

The starting dosage is 0.5mg TID. The dose should be increased by 0.5 mg increments no sooner than 2 weeks apart to 1 mg, 1.5 mg, 2 mg, and 2.5 mg TID, resulting in a maximum total daily dose of 7.5 mg. Patients will be maintained on a lower dose if higher doses are not tolerated (minimum dosage of 0.5 mg TID, total daily dose 1.5 mg; see Section 5.2.1).

To maintain blinding of the treatment arms, patients randomized to the placebo group will undergo sham titration from Visit 1 onwards during the dose-titration period according to the rules of the dose titration scheme. At Visit 6, the last visit of the Titration Phase, no further increase in study medication will be possible (see Figure 4–1)[10].

Dose titration algorithm

The study medication dose for the next titration step will be determined every 2 weeks according to the patient's well-being and the peripheral systolic blood pressure (SBP) measured at trough before intake of the morning dose as well as based on any of the 2 measurements (supine or sitting) performed 2 hours after dose intake to assess orthostatic changes [11]according to the following algorithm (dose titration scheme):

- If SBP is ≥ 95 mmHg and the patient has no signs or symptoms of hypotension, the dosage should be increased by 0.5 mg TID.
- If SBP is < 95 mmHg, the dosage should be maintained provided the patient <u>does not show any signs or symptoms of hypotension</u>.
- If SBP is < 95 mmHg, and the patient exhibits signs or symptoms of hypotension, the current dosage should be decreased by 0.5 mg TID.

During the dose-titration period a dose reduction can be performed according to patient well-being / safety concerns at investigator's discretion irrespective of SBP. If the investigator requests an increase or decrease in study medication dose via IxRS, the subsequent dose modification will not exceed ± 0.5 mg TID. Dose increases are allowed after a down-titration provided it is not done sooner than two weeks apart.

[10] Per Amendment 4
[11] Per Amendment 6

Maintenance period (Visits 7 to 12)

The overall duration of the main treatment phase is 52 weeks, including the dose titration period. At the end of the dose titration period (Week 10) the patient's maintenance dose will be determined using the treatment algorithm above. No further increase in dose will be allowed. The established dose will then be taken as the "optimal dose" to be administered for the remaining duration of the main treatment phase (up to Week 52).

Dose reductions for safety reasons (eg, in case of any treatment-emergent adverse event [TEAE]) are allowed, but a subsequent dose increase during the maintenance period is not permitted.

From Week 26 (Visit 10), patients will have the opportunity to add "rescue[12] therapy", consisting of immunosuppressant drugs, to their randomized study medication if they meet certain criteria for worsening skin or pulmonary disease. Please refer to Section 6.9 for further details.

4.1.3 Long-term extension phase

At Visit 12 (Week 52), patients randomized to the placebo arm in the main treatment phase will initiate active treatment with riociguat, as part of the long-term extension phase. During the first 10 weeks, a titration/sham-titration will be performed to maintain the study blind, after which the extension phase becomes open-label. During the extension phase, clinical outcomes will continue to be measured for exploratory long-term treatment effects. Adverse event information will continue to be collected during the extension phase as well.

The long-term extension phase will continue up to 6 years after the Last Patient Last Visit (LPLV) in the main treatment phase. Subsequent treatment should be discussed between the patient and his/her doctor during the final visit at the end of the extension phase. At the end of this study, it is expected that the patient will receive standard treatment at the discretion of the physician.

Dose-Titration period (Visits 12 to 17; Week 52 to 62)

At Visit 12 (Week 52), all patients will be assigned in IxRS to treatment with riociguat. To maintain the study blinding, neither the patient's prior treatment assignment nor dose will be unblinded. During the first 10 weeks of the long-term extension phase, patients previously on placebo will be up-titrated on riociguat according to the dose titration algorithm described above in the main treatment phase (Section 4.1.2). Patients randomized to riociguat in the main treatment phase will undergo sham titration to maintain the blinding during this period.

Open-label Extension period (Visit 18; Week 64 onward)

After completing the double-blind dose-titration period, from Week 64 onwards the study is open-label and the investigator will be able to see the current treatment dose. All patients will return to the clinic for visits every 3 months ± 2 weeks. Ongoing titration of riociguat within the range of 0.5 mg to 2.5mg TID will remain at the discretion of the investigator, and dose reductions for safety reasons are allowed.

[12] Per Amendment 1

4.1.4 Dose Interruptions - amended

Although not intended, patients may interrupt their intake of study medication for various reasons (eg, hospitalization in a remote hospital without study medication access, safety reasons or side effects). If treatment is interrupted, the following rules should be applied:

Main treatment phase:

- ≤3 consecutive days without treatment (9 missing doses) in the dose-titration period: restart at the same dose
- >3 consecutive days in the dose-titration period: discontinue[13] the patient from study medication
- >3 days but ≤14 consecutive days without treatment during the maintenance phase: treatment can be restarted at the discretion of the investigator at 0.5 mg TID lower than the last dose
- >14 consecutive days without treatment in the maintenance phase: discontinue[14] the patient from study medication.

Long-term extension phase:

- ≤3 consecutive days without treatment (9 missing doses) in the dose-titration period: restart at the same dose
- >3 days but ≤14 consecutive days without treatment in the dose-titration period: treatment can be restarted at the discretion of the investigator at 0.5 mg TID lower than the last dose
- >14 consecutive days without treatment in the dose-titration period: discontinue[15] the patient from study medication.
- Interruptions in the **open-label extension period**[16]:
    - >13 and ≤28 consecutive days: treatment can be restarted at the discretion of the investigator at 0.5 mg TID lower than the last dose.
    - >28 consecutive days: discontinue the patient from study medication.

[13] Per Amendment 4
[14] Per Amendment 4
[15] Per Amendment 4
[16] Per Amendment 4 and Amendment 5

4.1.5 Termination visit and safety follow-up visit - amended

A termination visit should be performed for patients who discontinue[17] from study medication for any reason except death or lost to follow-up, and should occur as soon as possible after the patient receives his/her last dose of study drug. In general, at the Termination Visit the same safety and efficacy relevant measurements and procedures should be performed as at Visit 12. If the discontinuation occurs before Visit 8 (Week 14), the skin biopsy and the blood samples for biomarkers are to be collected. No blood samples for pharmacokinetics (PK) have to be taken and vital signs post dose are not needed because no new medication will be dispensed to the patient. If the Termination Visit will be performed after Visit 12 (Week 52) the patient's and physician's global assessment, patient interference with skin assessment, and tender and swollen joint count assessment must not be performed.

A safety follow-up visit (30 [+5] days after the last dose of study medication) will be performed for all patients.

If the patient discontinues study drug[18] prematurely during the main treatment phase (Week 0 – Week 52) he/she will be invited to come for limited assessments (mRSS, pulmonary function test [FVC and DL_{CO} (diffusion capacity of the lung for carbon monoxide) including hemoglobin measurement at the local laboratory][19], patient-reported outcomes [PROs], and patient's and physician's global assessment[20]) at Week 12, Week 26, Week 39, and Week 52, depending on the timing of discontinuation[21]. The collection of these assessments is important for endpoint analysis which considers all assessments post baseline up to Week 52.

A detailed schedule of evaluations is presented in Section 7.1.1.

4.2 Primary variable

The primary efficacy outcome measure is the change in mRSS from baseline to Week 52.

4.3 Justification of the design

Riociguat has been safe and well tolerated in previous clinical studies at multiple doses between 0.5 and 2.5 mg TID in patients with PAH. Based on the positive results of riociguat in patients with PAH and CTEPH together with the compound's known antifibrotic effects as seen in *in vitro* and in animal models, patients with symptomatic dcSSc may benefit from treatment with riociguat. Currently, there is no approved therapy for dcSSc and no suitable active comparator in this target population. Therefore, the purpose of this study is to evaluate the efficacy and safety of riociguat versus placebo in patients with symptomatic dcSSc. The follow up time of 12 months has been chosen for the primary endpoint since it is considered an appropriate observation period to detect significant changes in the modified Rodnan Skin Score (mRSS) and is therefore used in many clinical studies with skin fibrosis.[15,16,17] Moreover, the 12-month trial duration is supported and validated by a 3-round Delphi consensus of measures for clinical trials of SSc [18]. See also Section 8.6 for further details.

[17] Per Amendment 4
[18] Per Amendment 1
[19] Per Amendment 4
[20] Per Amendment 5
[21] Per Amendment 4

4.4 End of study

For each participating EU country, the end of the study according to the EU Clinical Trial Directive will be reached when the last visit of the last patient for all centers in the respective country has occurred.

The end of the study as a whole will be reached as soon as the end of the study according to the above definition has been reached in all participating countries (EU and non-EU).

5. Study population

5.1 Eligibility

5.1.1 Inclusion criteria - amended

Patients must meet the following criteria to be eligible for enrollment in the study:

1. Written informed consent
2. Men or women aged 18 years and older
3. Systemic sclerosis, as defined by ACR/EULAR 2013 criteria[1]
4. dcSSc according to the LeRoy criteria[3] ie, skin fibrosis proximal to the elbows and knees in addition to acral fibrosis[22].
5. Disease duration of ≤ 18 months (defined as time from the first non−Raynaud's phenomenon manifestation)
6. ≥ 10 and ≤ 22 mRSS units at the screening visit
7. FVC ≥45% of predicted at screening
8. DL_{CO} ≥40% of predicted (hemoglobin-corrected) at screening
9. Negative serum pregnancy test in a woman of childbearing potential at the screening visit
10. Women of childbearing potential must agree to use adequate contraception when sexually active. "Adequate contraception" is defined as any combination of at least 2 effective methods of birth control, of which at least 1 is a physical barrier (eg, condom with hormonal contraception like implants or combined oral contraceptives, condom with intrauterine devices). This applies since signing of the informed consent form until 30 (+5) days after the last study drug administration.
11. Ability to comply with the clinical visits schedule and the study-related procedures

5.1.2 Exclusion Criteria - amended

Patients who meet any of the following criteria will be excluded from enrollment in the study:

1. Medical and surgical history
    - Limited cutaneous SSc at screening
    - Major surgery (including joint surgery) within 8 weeks prior to screening

[22] Per Amendment 4

- Patients with a history of malignancy in the last 5 years other than non-melanoma skin cell cancers cured by local resection or carcinoma in situ
- Known hypersensitivity to the study drug (active substance or excipients)

2. Hepatic-related criteria
   - Hepatic insufficiency classified as Child-Pugh C (see Appendix 14.1 for classification table)
     - Patients with isolated aspartate aminotransferase (AST) or ALT >3xULN (upper limit normal) or bilirubin >2xULN can be included in the trial under the condition of additional monitoring during the trial (see section 7.6.2)[23]
3. Renal-related criteria
   - Estimated glomerular filtration rate (eGFR) < 15 mL/min/1.73m^2 (Modification of Diet in Renal Disease [MDRD] formula) or on dialysis at the screening visit. Patients entering the trial with eGFR 15-29 mL/min/1.73m^2 will be undergo additional monitoring of renal function (see section 7.6.2)[24]
     - Because the MDRD formula is thought to cause significant bias for Japanese patients, the equation for Japanese patients is:
       194 x serum creatinine (mg/dL) -1.094 x Age -0.287 x 0.739 (if female).
   - Any prior history of renal crisis (see Appendix 14.2 for definition)
4. Cardiovascular-related criteria
   - Sitting SBP <95 mmHg at the screening visit
   - Sitting heart rate <50 beats per minute (BPM) at the screening visit
   - Left ventricular ejection fraction <40% prior to screening
5. Pulmonary-related criteria
   - Any form of pulmonary hypertension[25] as determined by right heart catheterization
   - Pulmonary disease with FVC <45% of predicted or DL_{CO} (hemoglobin-corrected) <40% of predicted at screening
   - Active state of hemoptysis or pulmonary hemorrhage, including those events managed by bronchial artery embolization
   - Any history of bronchial artery embolization or massive hemoptysis within 3 months before screening. (Massive hemoptysis is defined as acute bleeding >240 mL in a 24-hour period or recurrent bleeding >100 mL/day over consecutive days)
6. Laboratory examinations
   - Patients with: hemoglobin <9.0 g/dL, white blood cell (WBC) count <3000/mm^3 (<3 × 10^9/L), platelet count <100,000/mm^3 (<100 × 10^9/L)[26]

---

[23] Per Amendment 1
[24] Per Amendment 1
[25] Per Amendment 4 and Amendment 5

7. Prior and concomitant therapy
    - Concomitant use of nitrates or NO donors (such as amyl nitrate) in any form, including topical; phosphodiesterase (PDE) 5 (PDE5) inhibitors (such as sildenafil, tadalafil, vardenafil); and nonspecific PDE5 inhibitors (theophylline, dipyridamole)
    - Concomitant therapy with prostacyclin analogs. Oral beraprost for the treatment of digital ulcers / Raynaud's disease, and short-term / intermittent therapy of up to 21 days with intravenous prostacyclin analogs for digital / vascular lesions is allowed
    - Treatment with methotrexate, cyclophosphamide, hydroxychloroquine, cyclosporine A, azathioprine, mycophenolate mofetil, rapamycin, colchicine, D-penicillamine, tacrolimus[27], mizoribine[28] or intravenous immunoglobulin within 4 weeks before the screening visit
    - Treatment with etanercept within 2 weeks; infliximab, leflunomide, certolizumab, golimumab, adalimumab, abatacept or tocilizumab within 8 weeks; or anakinra within 1 week prior to the screening visit
    - Previous treatment with chlorambucil, bone marrow transplantation, or total lymphoid irradiation
    - Treatment with rituximab or other anti-CD20 antibodies within the last 6 months before screening
8. Other
    - Pregnant women or breast feeding women
    - Women of childbearing potential not willing to use adequate contraception (as defined in the aforementioned inclusion criteria, Section 5.1.1) and not willing to agree to 4-weekly pregnancy testing from Visit 1 (first administration of study drug) onwards until 30 (+5) days after last study drug intake.
    - Any other condition or therapy that would make the patient unsuitable for this study and will not allow participation for the full planned study period
    - Previous assignment to treatment during this study
    - Participation in another clinical study with an investigational drug or medical device within 30 days prior to randomization (phases I–III clinical studies)

5.1.3 Justification of selection criteria

The selection criteria were carefully selected to exclude patients from the study who may potentially be exposed to specific risks after administering the study drug as well as patients with conditions that may have an impact on the aims of this study.

---

[26] Per Amendment 6
[27] Per Amendment 1
[28] Per Amendment 1

5.2 Withdrawal of patients from study - amended

5.2.1 Withdrawal[29] - amended

5.2.1.1 Withdrawal from the study [30]

Patients *must* be withdrawn from the study for the following reasons:

- At their own request or at the request of their legally acceptable representative at any time during the study and without giving reasons, a patient may decline to participate further (withdrawal from study). The patient will not suffer any disadvantage as a result.
- Participation in another clinical trial after Visit 12. Participation in observational registries is allowed.

5.2.1.2 Discontinuation of study medication[31]

Patients *must* be discontinued from the study medication[32] for the following reasons:

- If, in the investigator's opinion, continuation of the study would be harmful to the patient's well-being
- Occurrence of AEs or intercurrent diseases which the investigator judges unacceptable for continuation of participation in the study
- Occurrence of adverse drug reactions, which in the investigator’s opinion have a negative impact on the patient’s individual risk-benefit ratio. (Investigators are obliged to reassess the patient’s individual risk-benefit ratio on a continuous basis. Factors such as anticipated treatment effect, progression of underlying disease, occurrence of side effects, and alternative treatment options must be considered.)
- If a subject, during the course of the study, develops
  a) renal disease that leads to eGFR <15/mL/min/1.73m^2,
  b) hepatic insufficiency classified as Child-Pugh C [33].
- Non-compliance with the conditions for the trial or instructions by the investigator
- If treatment is interrupted for >3 consecutive days (9 doses)[34] during the dose titration period of the main treatment phase
- If treatment is interrupted for >14 consecutive days during the maintenance period of the main treatment phase or the dose-titration period of the long-term extension phase
- If treatment is interrupted for >28 consecutive days during the open-label extension period (of the long-term extension phase)[35]
- In case of pregnancy or breast feeding.

[29] Per Amendment 4
[30] Per Amendment 4
[31] Per Amendment 4
[32] Per Amendment 4
[33] Per Amendment 5
[34] Per Amendment 1
[35] Per Amendment 4 and Amendment 5

- In case a female patient of childbearing potential is not compliant with 4-weekly pregnancy testing or with contraceptive measures.
- In case no further dose reduction is possible and the patient does not tolerate the lowest possible dosage (0.5 mg TID) of study drug

Patients *may* be discontinued from the study medication[36] for the following reasons:

- At the specific request of the sponsor and in liaison with the investigator (eg, safety concerns)
- A relative decline in FVC % predicted by ≥ 10% (eg, a change of FVC from 60% to 54%) or a relative decline in FVC % predicted between 5 to < 10% with associated relative decline in DL_{CO} % predicted by ≥ 15%, provided that the decline in FVC results in FVC <75% of predicted (confirmed by repeat pulmonary function testing within 1 month). Please refer to Section 6.9 for the option of adding rescue[37] therapy.
- An absolute increase in mRSS by > 5 units and ≥ 25% (Please refer to Section 6.9 for the option of adding rescue[38] therapy), life-threatening or organ-threatening event attributable to SSc (such as renal crisis, digital gangrene, development of new PAH on right heart catheterization) or not attributable to SSc.

5.2.1.3 Handling of withdrawals or discontinuations from study medication[39]

A patient who, for any reason (eg, failure to satisfy the selection criteria), terminates the study before the time point used for the definition of “dropout” (see below) is regarded a “screening failure”. Restarting the defined set of screening procedures to enable the “screening failure” patient’s participation at a later time point is not allowed.

One re-assessment of laboratory parameters is allowed during the screening phase to re-assess the eligibility of patients.

A patient who discontinues study participation prematurely for any reason is defined as a “dropout” if the patient has already been randomized.

Any randomized patient removed from the trial should undergo the assessments at the termination visit. The patient will remain under medical supervision until discharge or transfer is medically acceptable and will complete the 30-day safety follow-up period.

If the patient discontinues study drug[40] prematurely during the main treatment phase (Week 0 – Week 52) he/she will be invited to come for limited assessments (mRSS, pulmonary function test [FVC and DL_{CO} including hemoglobin measurement at the local laboratory][41], PROs, and patient’s and physician’s global assessment)[42] at Week 12, Week 26, Week 39, and Week 52, depending on the timing of discontinuation[43]. This will be in addition to the termination visit assessments (see Section 7.1.2.8), provided that the patient does not object to

[36] Per Amendment 4
[37] Per Amendment 1
[38] Per Amendment 1
[39] Per Amendment 4
[40] Per Amendment 1
[41] Per Amendment 4
[42] Per Amendment 5
[43] Per Amendment 4

collection of study data after withdrawal of consent. If the discontinuation occurs before Visit 8 (Week 14), the skin biopsy and blood samples for biomarkers are to be collected.

All reasons for discontinuation[44] (including lost to follow up), will be recorded in the electronic case report from (eCRF) and site medical record. Study medication assigned to the discontinued[45] patient may not be assigned to another patient.

In the event that a patient is lost to follow-up, every possible effort must be made by study site personnel to contact the patient to obtain complete data and determine the reason for discontinuation[46].

A patient may withdraw from further participation in the study and, unless he/she objects to further data collection, the following information should be obtained and documented in the eCRF[47]:

- Vital status (at Week 52)
- Outcome of serious adverse events (SAEs)
- Efficacy endpoints at Weeks 12, 26, 39, and 52 (depending on timing of discontinuation of study medication)

The information should be collected in the eCRF until the safety follow-up visit 30 (+5) days after the last intake of study drug. Serious adverse events that occur within 30 (+5) days after the last dose of study drug will be followed up until resolution, if possible.

If patients refuse to be contacted via telephone (eg, patient objects to further release of information or are lost to follow-up), every effort should be made to obtain vital status (dead or alive) information at Week 52 through consultation of public databases, wherever allowed by local regulations[48].

The measures taken for follow up must be documented in the site medical record. Follow-up data will be collected for all randomized patients except those who specifically withdraw consent for release of such information, except as noted above. Thus, it is imperative to obtain complete data for all randomized patients, including information on efficacy and safety endpoint events, whether or not they receive study drug, or discontinue study drug prematurely.

Details for the premature termination of the study as a whole (or components thereof [eg, centers, treatment arms, dose steps]) are provided in Section 10.

5.2.2 Replacement - amended

There will be no replacement of randomized patients who withdraw from the study or prematurely discontinue study medication[49].

[44] Per Amendment 4
[45] Per Amendment 4
[46] Per Amendment 4
[47] Per Amendment 4
[48] Per Amendment 4
[49] Per Amendment 4

5.3 Patient identification

After a patient signs the informed consent form, the patient identification number for each patient will be provided to the investigators via IxRS. Patients will be identified by a unique 9-digit number consisting of the following:

- First 2 digits = Unique country code
- Next 3 digits = Unique study center number within the country
- Last 4 digits = Unique current patient number within the study center of a given country; sequential number reflecting the order in which the patients signed the informed consent form at the center (4 digits)

Patient identification numbers must be used in sequence and no number should be skipped, substituted, or reused.

6. Treatment

6.1 Treatments to be administered

Test drug:	Riociguat
Dosage:	0.5 mg, 1 mg, 1.5 mg, 2 mg, and 2.5 mg (dose titration)
Route of administration:	Oral
Time and frequency of administration:	TID

The optimal dose should be determined during the initial 10-week dose titration period based on monitoring of the patient's SBP and well-being.

The recommended starting dose is 0.5 mg TID. The intervals between drug intake should be 6−8 hours. The dosage should be increased by 0.5 mg increments no sooner than 2 weeks apart to 1 mg, 1.5 mg, 2 mg, and 2.5 mg TID, resulting in a maximum total daily dose of 7.5 mg. Patients should be maintained on lower doses if higher doses are not tolerated (minimum dose of 0.5 mg TID, see Section 5.2.1). After the dose titration period, riociguat should be continued at the optimal dose for the duration of the maintenance period.

6.2 Identity of study treatment

Riociguat

Riociguat is supplied in various strengths as film-coated, immediate-release tablets (see Table 6–1). Riociguat and placebo tablets will have similar appearance at all dose strengths.

All study drugs will be labelled according to the requirements of local law and legislation. Label text will be approved according to the sponsor's agreed procedures, and a copy of the labels will be made available to the study site upon request.

For all study drugs, a system of numbering in accordance with all requirements of Good Manufacturing Practice (GMP) will be used, ensuring that each dose of study drug can be traced back to the respective bulk ware of the ingredients. Lists linking all numbering levels will be maintained by the sponsor's clinical supplies Quality Assurance group.

A complete record of batch numbers and expiry dates of all study treatment, as well as the labels, will be maintained in the sponsor study file.

Table 6–1 Identity of investigational product

International non-proprietary name (INN)	Riociguat
Sponsor's internal reference number	BAY 63-2521
Formulation	Film-coated tablets
Galenical form	Round immediate-release tablets, diameter 6 mm
Composition	Active ingredient: *Methyl 4,6-diamino-2-[1-(2-fluorobenzyl)-1H-pyrazolo [3,4 b]pyridine-3-yl]-5-pyrimidinyl (methyl)carbamate*<br>Empirical formula: $C_{20}H_{19}FN_8O_2$<br>Molar mass: 422.42 g/mol (molarity)<br>Excipients: lactose, microcrystalline cellulose, magnesium stearate, crospovidone* , hypromellose*, and sodium lauryl sulphate*<br>Coating: hydroxypropyl cellulose, hypromellose, propylene glycol, iron oxide (red and yellow), and titanium dioxide.
Strength	0.5 mg, 1 mg, 1.5 mg, 2 mg, 2.5 mg
Material numbers	83296470 BAY 63-2521 TABL 0.5 mg 511 COAT<br>83296535 BAY 63-2521 TABL 1 mg 512 COAT<br>83296543 BAY 63-2521 TABL 1.5 mg 513 COAT<br>83296578 BAY 63-2521 TABL 2 mg 504 COAT<br>83296608 BAY 63-2521 TABL 2.5 mg 515 COAT
Packaging	High-density polyethylene (HDPE) bottles

Abbreviation: TABL = tablet
*Not in placebo tablets

Matching placebo

Matching placebo tablets will appear identical to active riociguat tablets but will not contain active study drug product (riociguat) or any of the excipients marked with an asterisk as outlined in Table 6–1.

Storage requirements

All investigational drugs used during the trial will be stored at the investigational sites at room temperature in a place inaccessible to unauthorized personnel, ie, in a locked cabinet.

No special storage conditions are required.

6.3 Treatment assignment

This is a randomized, double-blind, placebo-controlled, parallel-group, multinational, multicenter study. Patients who complete all screening procedures and meet all eligibility criteria are to be randomized via IxRS in a 1:1 ratio to receive either riociguat or placebo.

The patient randomization scheme will use permutated blocks sized as a multiple of 2. Each block will be allocated to a country or group of nearby countries within a region. No other stratification is planned.

An IxRS site manual will be provided to the investigative centers.

6.4 Dosage and administration

6.4.1 Selection of doses in the study - amended

Riociguat has been safe and well tolerated in previous clinical studies at multiple doses between 0.5 and 2.5 mg TID in patients with various forms of pulmonary hypertension, including PAH, CTEPH, and, in a small patient population, SSc-related pulmonary hypertension. The intervals between drug intake should be 6 to 8 hours[50].

Data from the phase III clinical program for riociguat in patients with pulmonary hypertension (PATENT and CHEST studies) showed that a starting dose of 1 mg TID was safe and effective. Because a few patients in both studies had their dosage reduced to 0.5 mg TID, the sponsor recognized that the 0.5 mg TID dosage may offer additional tolerability for some patients and adopted this additional dose recommendation. In the present study, the titration will start at 0.5 mg TID.

For further details refer to the IB.

6.5 Blinding - amended

Apart from the open-label extension period in the long-term extension phase, the study will be conducted in a double-blind fashion. Active riociguat and placebo tablet formulations will be identical in appearance (size, shape, color) and smell. The packaging and labeling will be designed to maintain blinded conditions for the investigator's team and the patients. The study data will remain blinded until database lock and authorization of data release according to standard operating procedures.

Appropriate measures will be taken to maintain blinding of the study team while PK and biomarker analysis is ongoing. Data with a potential to unblind (eg, plasma concentrations) are not to be handled by or shown to the study team members and will only be uploaded into the clinical database after official unblinding. The process of data flow will be documented separately (see also Section 7.4 and Section 7.6.3.).[51]

Statistical analysis as described in the SAP (Statistical Analysis Plan) will be done after database lock and authorization of data release according to standard operating procedures. [52]

Unblinding by drug safety personnel of the sponsor

For regulatory reporting purposes, drug safety personnel of the sponsor are permitted to unblind individual cases.

In compliance with applicable regulations, in the event of a suspected unexpected serious adverse reaction (SUSAR) (see Section 7.5.1.5), the patient's treatment code will usually be unblinded before reporting to the health authorities, Independent Ethics Committees (IECs)/ Institutional Review Boards (IRBs), and investigators (see Section 7.5.1.4) if the SUSAR was related to the blinded treatment.

---

[50] Per Amendment 4
[51] Per Amendment 5
[52] Per Amendment 5

Emergency unblinding by the investigator

In case of emergency, investigators are permitted to unblind individual cases via the IxRS. However, investigators are obligated to restrict such unblinding to cases of emergency where the unblinding result is of importance for the acute treatment strategy.

Investigators should note that the occurrence of a serious adverse event (SAE) should not routinely precipitate the immediate unblinding of the label.

Date, time, and reason for unblinding must be captured in the relevant eCRF. Please refer to the IxRS and eCRF completion guideline for instructions.

Any patient who discontinues study medication will remain under medical supervision until discharge or transfer is medically acceptable. Patients who withdraw from the study or discontinue study medication should take part at the termination visit (refer to Section 7.1.2.8) and the safety follow-up visit (refer to Section 7.1.2.9)[53]. Further therapy is at the discretion of the investigational site.

Unblinding for ongoing safety monitoring

In order to allow ongoing safety monitoring during the conduct of the study by an external DMC, members of the committee will receive unblinded safety data. The involvement of an external statistical analysis center in this process will ensure that unblinded information is not available for third parties. Details of the process are described in the DMC charter.

6.6 Drug logistics and accountability

All study drugs will be stored at the investigational site in accordance with Good Clinical Practice (GCP) and GMP requirements and the instructions given by the clinical supplies department of the sponsor, and will be inaccessible to unauthorized personnel. Special storage conditions and a complete record of batch numbers and expiry dates can be found in the sponsor study file; the site-relevant elements of this information will be available in the investigator site file. The responsible site personnel will confirm receipt of study drug via IxRS and will use the study drug only within the framework of this clinical study and in accordance with this protocol. Receipt, distribution, return and destruction (if any) of the study drug must be properly documented according to the sponsor's agreed and specified procedures.

Written instructions on medication destruction will be made available to affected parties as applicable.

6.7 Treatment compliance

A drug dispensing log will be completed for each patient. The overall compliance with study drug intake should be between 80% and 120% of the calculated dose. The date of dispensing the study drug to the patient will be documented.

Patients will receive study medication dispensed per visit schedule (see Section 7.1.1).

To record treatment compliance, patients will be instructed to bring all study drug packaging, including unused study drug and empty packaging, to the investigative site at each study visit. Tablets will be counted for a compliance check.

[53] Per Amendment 4

If a dose of study drug is missed, the patient should take a dose immediately and continue with the TID intake as recommended on the following day. The dose should not be doubled to make up for a missed dose within the same day.

6.8 Post-study therapy

In case patients discontinue study drug treatment during this study, further therapy is at the discretion of the investigator.

6.9 Prior and concomitant therapy - amended

A summary of the prohibited prior and concomitant therapy, outlined in the exclusion criteria (Section 5.1.2), is provided in Table 6–2. During the trial, concomitant treatment is allowed with oral corticosteroids (≤10 mg/day of prednisone or equivalent), non-steroidal anti-inflammatory drugs (NSAIDs), angiotensin converting enzyme (ACE) inhibitors, or calcium channel blockers if stable for ≥2 weeks before and including at baseline. These treatments will also be allowed as new-onset during the study at the discretion of the investigator to treat SSc-specific adverse events (eg, Raynaud's phenomena, joint inflammation, new onset renal crisis).

During the long-term extension phase (ie, after completion of all efficacy- and safety-related procedures at Visit 12), except for the contraindicated nitrates or NO donors and PDE5 inhibitors, the addition of any other concomitant medication is at the discretion of the investigator. [54]

[54] Per Amendment 5

Table 6–2 Prohibited prior and concomitant therapy - amended

Therapy	Timeframe
Chlorambucil, bone marrow transplantation, or total lymphoid irradiation	Any previous therapy[a, b]
Rituximab or other anti-CD20 antibodies	Within 6 months prior to the screening visit[a, b]
Infliximab, certolizumab, golimumab, adalimumab, abatacept, leflunomide or tocilizumab	Within 8 weeks prior to the screening visit[a, b]
Methotrexate, cyclophosphamide, hydroxychloroquine, cyclosporine A, azathioprine, mycophenolate mofetil, rapamycin, colchicine, D penicillamine, tacrolimus[55], mizoribine[56] or intravenous immunoglobulin	Within 4 weeks prior to the screening visit[a, b]
Etanercept	Within 2 weeks prior to the screening visit[a,b]
Anakinra	Within 1 week prior to the screening visit[a,b]
Nitrates or NO donors (such as amyl nitrate) in any form, including topical; phosphodiesterase (PDE) 5 (PDE5) inhibitors (such as sildenafil, tadalafil, vardenafil); and nonspecific PDE5 inhibitors (theophylline, dipyridamole)	Concomitant therapy with study drug
Prostacyclin analogs (oral beraprost for digital ulcers / Raynaud's disease and short-term / intermittent therapy of up to 21 days with intravenous prostacyclin analogs for digital / vascular lesions is allowed)	Concomitant therapy with study drug

a Concomitant use of these treatments with study medication is also prohibited, except for certain agents from Week 26 onwards; please see "Rescue[57] Therapy" below.
b Concomitant use of these medications will be allowed during the long-term extension phase[58]

In addition, as described in Section 8.4.1 of the IB, "Interaction with other medicinal products and other forms of interaction", caution is advised during the intake of any of the following concomitant medications:

- The concomitant use of riociguat with strong multi-pathway cytochrome P450 (CYP) and P-glycoprotein 1 (P-gp)/breast cancer resistance protein inhibitors such as azole antimycotics (eg, ketoconazole, itraconazole) or human immunodeficiency virus protease inhibitors (eg, ritonavir) is not recommended, due to the pronounced increase in riociguat exposure.
- The concomitant use of riociguat with strong CYP1A1 (CYP family 1, subfamily A, polypeptide 1) inhibitors, such as the tyrosine kinase inhibitor erlotinib, and strong P-gp inhibitors, such as the immunosuppressive agent cyclosporine A, may increase

[55] Per Amendment 1
[56] Per Amendment 1
[57] Per Amendment 1
[58] Per Amendment 5

riociguat exposure. These drugs should be used with caution. Blood pressure should be monitored and dose reduction of riociguat considered.

Other medications and considerations (detailed information can be found in the current IB)

- Pre- and concomitant treatment with the proton pump inhibitor omeprazole (40 mg once daily) reduced riociguat mean area under the plasma concentration versus time curve (AUC) by 26% and mean maximum plasma concentration (C_{max}) by 35%. This is not considered clinically relevant.
- Co-administration of the antacid aluminum hydroxide / magnesium hydroxide reduced riociguat mean AUC by 34% and mean C_{max} by 56%. Antacids should be taken at least 1 hour after riociguat.
- Plasma concentrations in smokers are reduced by 50–60% compared with nonsmokers. For this reason, it is highly recommended to strongly advise patients to stop smoking while taking this medication[59]. A dose reduction should be considered in patients who stop smoking whilst participating in the study.

Rescue[60] therapy

From Week 26 (Visit 10), patients with the following will have the opportunity to add rescue therapy to their randomized study medication. "Rescue therapy" is defined as treatment with an immunosuppressant drug, under the following situations:

- Worsening of skin disease (defined as >5 units and ≥25% increase in mRSS), or
- Relative decline in FVC % predicted by ≥10%, or relative decline in FVC % predicted between ≥5% and <10% with associated relative decline in DL_{CO} % predicted by ≥15%, provided that the decline in FVC results in FVC <75% of predicted (confirmed by repeat pulmonary function testing within 1 month).

The decision to initiate rescue therapy is based on investigator discretion in eligible patients. Rescue therapy may include any of the following 4 agents: methotrexate, mycophenolate mofetil, cyclophosphamide, or azathioprine.

- Worsening of inflammatory joint disease or myositis.[61]

Rescue therapy can be initiated at investigator's discretion in eligible patients. In addition to the 4 agents methotrexate, mycophenolate mofetil, cyclophosphamide, or azathioprine, hydroxychloroquine will be allowed for the treatment of inflammatory joint disease and myositis. [62]

These treatments are not provided by the Sponsor.

A survey conducted by the Scleroderma Clinical Trials Consortium (SCTC) and Canadian Scleroderma Research Group (CSRG) to gain a consensus from SSc experts worldwide recommended these agents in the management of SSc after failure of first-line therapy.[19]

[59] Per Amendment 1
[60] Per Amendment 1– this footnote covers all changes in the section from escape to rescue
[61] Per Amendment 5
[62] Per Amendment 5

These agents also are featured in EULAR recommendations for the treatment of SSc[20] and in a recently published review of old and new therapies for SSc used in clinical practice.[21]

7. Procedures and variables

7.1 Schedule of procedures

The study will be divided into the following phases: screening phase, main treatment phase and long-term extension phase. Please refer to Table 7–1 for a schedule of evaluations.

7.1.1 Tabulated overview - amended

Table 7–1 Schedule of evaluations - amended[63]

	Scr	Main Treatment Phase (Double-blind)[v]												Long-term Extension Phase							
	Scr Phase	Dose-Titration Period						Maintenance Period						Dose-Titration Period					Open-label Extension Period	Safety Follow-up	
**Visit**	**V0**	**V1**	**V2**	**V3**	**V4**	**V5**	**V6**	**V7**	**V8**	**V9**	**V10**	**V11**	**V12**[a]	**V13**	**V14**	**V15**	**V16**	**V17**	**V18 – Vn (q 12 wk)**	**TV**[b]	**SFU**
**Week**	-2	0	2	4	6	8	10	12	14	20	26	39	52	54	56	58	60	62	64 – n		
**Day**	**-14 to -1**	**0**	**14 ± 2**	**28 ± 2**	**42 ± 2**	**56 ± 2**	**70 ± 2**	**84 ± 2**	**98 ± 2**	**140 ± 2**	**182 ± 2**	**273 ± 14**	**364 -14 to +2**	**378 ± 2**	**392 ± 2**	**406 ± 2**	**420 ± 2**	**434 ± 2**	**448 – (n + 84) V18 ± 2 Vn ± 14**		**30 + 5**
Written informed consent	X																				
Inclusion / exclusion criteria	X	X																			
Demographic data	X																				
Medical and surgical history	X	X																			
Other history (alcohol)	X																				
Smoking history	X	X									X		X						X	X	
Height and weight[c]	X	X						X			X		X						X	X	X
Physical examination	X	X						X			X		X						X	X	X
mRSS	X							X			X	X	X						X	X	
Assessment of Raynaud's attacks	X[d]	X[e]						X[f]	X[e]												
Digital ulcer net burden assessment	X	X	X	X	X	X	X	X	X	X	X	X	X	X	X	X	X	X	X	X	

[63] Visit window for V12 changed per Amendment 4; visit window for V18 changed per Amendment 5

Table 7–1 Schedule of evaluations - amended[63]

	Scr	Main Treatment Phase (Double-blind) [v]												Long-term Extension Phase							
	Scr Phase	Dose-Titration Period						Maintenance Period						Dose-Titration Period					Open-label Extension Period	Safety Follow-up	
**Visit**	**V0**	**V1**	**V2**	**V3**	**V4**	**V5**	**V6**	**V7**	**V8**	**V9**	**V10**	**V11**	**V12[a]**	**V13**	**V14**	**V15**	**V16**	**V17**	**V18 – Vn (q 12 wk)**	**TV[b]**	**SFU**
**Week**	-2	0	2	4	6	8	10	12	14	20	26	39	52	54	56	58	60	62	64 – n		
**Day**	**-14 to -1**	**0**	**14 ± 2**	**28 ± 2**	**42 ± 2**	**56 ± 2**	**70 ± 2**	**84 ± 2**	**98 ± 2**	**140 ± 2**	**182 ± 2**	**273 ± 14**	**364 -14 to +2**	**378 ± 2**	**392 ± 2**	**406 ± 2**	**420 ± 2**	**434 ± 2**	**448 – (n + 84) V18 ± 2 Vn ± 14**		**30 + 5**
Tender & swollen joint count	X												X							X[g]	
Oximetry[w, 64]										X	X	X	X	X	X	X	X	X	X	X	X
Vital signs (BP, HR)[h]	X	X	X	X	X	X	X	X	X	X	X	X	X	X	X	X	X	X	X	X	X
Vital signs post-dose (BP, HR)[i,j]		X[i]										X[j]	X[i]								
BP and HR for assessment of orthostatic changes[x, 65]												X	X	X	X	X	X	X			
Blood sample for safety[k, u]	X	X	X[u]	X[u]	X[u]	X[u]	X				X[66]	X	X					X	X	X	X
Blood sample for PK		X[l]	X[m]	X[m]	X[m]	X[m]	X[m]					X[n]	X[l]	X[m]	X[m]	X[m]	X[m]	X[m]			
Blood sample for biomarkers		X							X											X[r]	
Blood sample for Autoantibody screen	X																				
Pregnancy test (if applicable)[o]	X	X		X		X		X		X			X		X		X		X	X	X
12-lead ECG[p]	X	X						X			X	X	X						X	X	X

[64] Per Amendment 6
[65] Per Amendment 6
[66] Per Amendment 1

Table 7–1 Schedule of evaluations - amended[63]

	Scr	Main Treatment Phase (Double-blind) [v]												Long-term Extension Phase							
	Scr Phase	Dose-Titration Period						Maintenance Period						Dose-Titration Period					Open-label Extension Period	Safety Follow-up	
**Visit**	**V0**	**V1**	**V2**	**V3**	**V4**	**V5**	**V6**	**V7**	**V8**	**V9**	**V10**	**V11**	**V12[a]**	**V13**	**V14**	**V15**	**V16**	**V17**	**V18 – Vn (q 12 wk)**	**TV[b]**	**SFU**
**Week**	-2	0	2	4	6	8	10	12	14	20	26	39	52	54	56	58	60	62	64 – n		
**Day**	**-14 to -1**	**0**	**14 ± 2**	**28 ± 2**	**42 ± 2**	**56 ± 2**	**70 ± 2**	**84 ± 2**	**98 ± 2**	**140 ± 2**	**182 ± 2**	**273 ± 14**	**364 -14 to +2**	**378 ± 2**	**392 ± 2**	**406 ± 2**	**420 ± 2**	**434 ± 2**	**448 – (n + 84) V18 ± 2 Vn ± 14**		**30 + 5**
Pulmonary function tests[q]	X							X			X	X	X						X	X	
Skin biopsy		X							X											X[r]	
Questionnaires[s]		X							X		X	X	X						X	X	
Patient's and physician's global assessment		X									X		X							X[g]	
Patient interference with skin		X									X		X							X[g]	
Tendon Friction Rubs[t]		X							X												
Randomization (IxRS)		X																			
Dispensation of study drug		X	X	X	X	X	X	X	X	X	X	X	X	X	X	X	X	X	X		
Drug accountability			X	X	X	X	X	X	X	X	X	X	X	X	X	X	X	X	X	X	
Adverse events recording	X	X	X	X	X	X	X	X	X	X	X	X	X	X	X	X	X	X	X	X	X
Prior and concomitant therapy	X	X	X	X	X	X	X	X	X	X	X	X	X	X	X	X	X	X	X	X	X
Survival status																					X

Abbreviations: BP = blood pressure; ECG = electrocardiogram; TV = Termination Visit; SFU = 30-Day Safety Follow-up (30 [+5] days after the last dose of study drug); HR = heart rate; IxRS = telephone-based or web-based response system; mRSS = modified Rodnan skin score; PK = pharmacokinetics; q 12 wk = every 12 weeks; Scr = Screening.
Note: Day 0 (Visit 1) is the baseline visit.
a Visit 12 (Week 52) is the last visit in the Maintenance Period of the Main Treatment phase and the first (baseline) visit in the Long-term Extension phase.

b To be performed as soon as possible after discontinuation[67] of study medication.
c Height to be measured only at the screening visit
d Patient to be provided with 7-day diary, to complete daily for 7 consecutive days leading up to Visit 1
e Patient and physician assessment of Raynaud's disease (see Section 7.6.12)
f Patient to be provided with 7-day diary, to complete daily for 7 consecutive days leading up to Visit 8.
g Must not be performed if TV is after V12.
h Blood pressure and heart rate will be measured at all visits with the patient in a sitting position, after the patient has been at rest for at least 5 minutes. The same arm is always used for these measurements (see Section 7.6.5).
i Vital signs to be measured within 20 minutes prior to each post-dose PK sampling (see Table 7–2)
j Vital signs to be measured within 20 minutes prior to PK sampling (see Table 7–2)
k Coagulation tests to be performed at a local laboratory
l Samples for PK to be taken 1 – 2 hours and 3 – 4 hours after the morning dose of study medication (see Table 7–2)
m Sample for PK to be taken up to 1 hour before the morning dose of study medication (trough) (see Table 7–3)
n Samples for PK to be taken up to 1 hour before the morning dose of study medication (trough) and 2 – 3 hours after the morning dose of study medication (peak) (see Table 7–4)
o For women of childbearing potential only. Serum pregnancy test is required only at screening; pregnancy testing (urine) will be done every 4 weeks and should continue until 30 days after the last dose of study drug. When pregnancy testing coincides with a visit, the test should be performed at the site, but otherwise should be performed at home.
p Standard ECG (12-lead ECG) will be recorded with the patient in the supine position, after the patient has been at rest for at least 5 minutes (see Section 7.6.7).
q Pulmonary function tests include forced vital capacity (FVC) and diffusion capacity of the lung for carbon monoxide (DL_{CO}).
r Must be done at a TV if this visit occurs before Visit 8.
s Questionnaires include Scleroderma Health Assessment Questionnaire (SHAQ), Short Form 36 (SF-36), Patient-Reported Outcomes Measurement Information System (PROMIS)-29, and University of California, Los Angeles, Scleroderma Clinical Trial Consortium Gastrointestinal Scale (UCLA SCTC GIT) 2.0.
t Assessment of the presence or absence of tendon friction rubs
u Additional monitoring may apply for patients with impaired renal and liver function at Visit 2, 3, 4, 5 as needed (see Section 7.6.2)[68]
v All patients who discontinue study drug prematurely during the main treatment phase (Week 0 – Week 52) will be invited to come for limited assessments (see Sections 7.1.2.10 and 7.6.2).[69]
w Oxygen saturation will be measured using forehead pulse oximetry. At visits with blood samples for PK and blood pressure measurements for the assessment of orthostatic changes, post-dose measurements will be performed in addition to pre-dose measurements (see Section 7.6.18, Table 7–2, Table 7–3, Table 7–4)[70]
x Measure supine and sitting blood pressure and heart rate to check for orthostatic changes approximately 2 hours after intake of study medication. (see Section 7.6.5, Table 7–2, Table 7–3, Table 7–4).[71]

[67] Per Amendment 4
[68] Per Amendment 1
[69] Per Amendment 4
[70] Per Amendment 6
[71] Per Amendment 6

Pharmacokinetics sampling flow charts - amended

Profile day (Visit 1 and Visit 12)

- 2 hours **after** administration of the morning dose of study medication[72]
- 3 – 4 hours **after** administration of the morning dose of study medication

Table 7–2 Profile Day PK Sampling (Visit 1 and Visit 12)[73]

Visit 1 and Visit 12 (profile days)			Cycle of procedures				
**Order of procedures** ↓	Starting point of procedures	Time interval (h)	**00**	00	**1:45 – 2 h**	**2h**	**3 – 4 h**
	At Visit 12 only: oxygen saturation using forehead pulse oximetry		X				
	Blood pressure, heart rate		X				
	Administration of morning dose of study medication			X			
	At Visit 12 only: oxygen saturation using forehead pulse oximetry				X[a]		
	Blood pressure, heart rate; at Visit 12 only: supine and sitting position				X[a]		
	Blood sample for PK					X	
	Blood pressure, heart rate (within 20 minutes prior to PK sample)						X
	Blood sample for PK						X

a At Visit 12 only: measure oxygen saturation, blood pressure and heart rate in supine and sitting position (see Section 7.6.5 and Section 7.6.18).
Abbreviations: h = hours; PK = pharmacokinetic.

Visits 2 to 6 and Visits 13 to 17

- Up to 1 hour **before** administration of the morning dose of study medication (trough)

Table 7–3 PK Sampling (Visits 2 – 6 and Visits 13 – 17)[74]

Visit 2 to Visit 6 and Visit 13 to Visit 17			Cycle of procedures			
**Order of procedures** ↓	Starting point of procedures	Time interval (h)	**–1 h – 00**	**00**	00	2h
	Blood sample for PK		X			
	Visit 13-17 only: oxygen saturation using forehead pulse oximetry			X		
	Blood pressure, heart rate			X		
	Administration of morning dose of study medication				X	
	Visit 13-17 only: oxygen saturation using forehead pulse oximetry					X
	Visits 13-17 only: Blood pressure, heart rate in supine and sitting position					X

[72] Per Amendment 6
[73] Per Amendment 6
[74] Per Amendment 6

Abbreviations: h = hours; PK = pharmacokinetic.

Visit 11

- Up to 1 hour **before** administration of morning dose of study medication (trough)
- 2 hours **after** administration of morning dose of study medication (peak)[75]

Table 7–4 PK Sampling (Visit 11)[76]

Visit 11			Cycle of procedures				
**Order of procedures**	Starting point of procedures	Time interval (h)	**–1 h – 00**	**00**	00	**1:45-2 h**	**2 h**
	Blood sample for PK		X				
	Oxygen saturation using forehead pulse oximetry			X			
	Blood pressure, heart rate			X			
	Administration of morning dose of study medication				X		
	Oxygen saturation using forehead pulse oximetry					X[a]	
	Blood pressure, heart rate in supine and sitting position					X[a]	
	Blood sample for PK						X

a Measure oxygen saturation, blood pressure and heart rate in supine and sitting position (see Section 7.6.5 and Section 7.6.18).
Abbreviations: h = hour; PK = pharmacokinetics.

7.1.2 Timing of assessments

If not stated otherwise, all assessments and procedures will be performed by or under the supervision of an investigator.

For timing of assessments and procedures, please refer to Section 7.1.1.

7.1.2.1 Visit 0 – Screening phase – Screening visit

Screening evaluations will be performed only after the patient has provided written informed consent. The following evaluations will be performed and information obtained up to 14 days (but not less than 8 days) before randomization and the start of study drug treatment:

- Patient information and obtaining of written informed consent
- Eligibility: Assessment of inclusion and exclusion criteria (see Section 5.1)
- Demographic data, including sex, ethnicity, date of birth, smoking history and alcohol consumption (See Section 7.2.1)
- Medical and surgical history (see Section 7.2.2)
- Prior and concomitant therapy (see Section 6.9 for prohibited medication)

[75] Per Amendment 6
[76] Per Amendment 6

- Physical examination, including height and weight (Note: body mass index [BMI] will be calculated automatically in the eCRF)
- Vital signs (blood pressure and heart rate). Blood pressure and heart rate will be measured at all visits with the patient in a sitting position, after the patient has been at rest for at least 5 minutes. The same arm is always used for these measurements (See Section 7.6.5)
- mRSS (see Section 7.6.6)
- Provision of 7-day diary to patients which they will complete daily for 7 consecutive days leading up to Visit 1 (see Section 7.6.12)
- Digital ulcer net burden assessment (see Section 7.6.10)
- Tender and swollen joint count assessment (see Section 7.6.14)
- Blood samples for safety (see Section 7.6.2)
- Blood sample for autoantibody screen (see Section 7.6.4)
- Pregnancy test (serum) for all women of childbearing potential (see Section 7.6.1)
- 12-lead electrocardiogram (ECG) for centralized reading. A standard 12-lead ECG will be obtained with the patient in the supine position after resting for at least 5 minutes (see Section 7.6.7)
- Pulmonary function tests (FVC and DL_{CO}) (see Section 7.6.8)
- Continuous assessment of AEs will start immediately after signing the informed consent form (see Section 7.5.1)

7.1.2.2 Visit 1 – Main treatment phase - Baseline (Day 0, Week 0)

The following assessments will be performed at Visit 1 (Day 0 of study drug treatment):

<u>Before intake of study medication</u>

- Reconfirmation of eligibility (assessment of inclusion/exclusion criteria; Section 5.1)
- Questionnaires (see Section 7.6.13)
- Update of medical and surgical history, if needed (see Section 7.2.2)
- Smoking history
- Physical examination, including weight
- Vital signs (blood pressure and heart rate). Blood pressure and heart rate will be measured at all visits with the patient in a sitting position, after the patient has been at rest for at least 5 minutes. The same arm is always used for these measurements (see Section 7.6.5).
- Patient and physician assessments of Raynaud's attacks, and patient to return completed 7-day diary (see Section 7.6.12)
- Digital ulcer net burden assessment (see Section 7.6.10)
- Patient's and physician's global assessment (see Section 7.6.15)
- Patient interference with skin assessment (see Section 7.6.16)

- Assessment for the presence or absence of tendon friction rubs (see Section 7.6.17)
- Blood samples for safety (see Section 7.6.2)
- Blood sample for biomarkers to be obtained before the start of study drug treatment (see Section 7.6.3)
- Pregnancy test (urine) for all women of childbearing potential (see Section 7.6.1)
- 12-lead ECG for centralized reading. A standard 12-lead ECG will be obtained with the patient in the supine position after resting for at least 5 minutes (See Section 7.6.7)
- Skin biopsy to be obtained before the start of study drug treatment (see Section 7.6.9)
- Recording and assessment of AEs (see Section 7.5.1)
- Concomitant therapy
- Randomization (IxRS) to study drug treatment with either riociguat or placebo (see Section 6)
- Dispensation of study drug and first intake by patient

<u>After intake of study medication</u>

- Blood sample for PK (see Section 7.4)
  - PK blood sample between 1 – 2 hours after administration of the morning dose of study medication
  - PK blood sample between 3 – 4 hours after administration of the morning dose of study medication
- Vital signs (blood pressure and heart rate) should be measured within 20 minutes prior to each post-dose PK sampling with the patient in a sitting position and after the patient has been at rest for at least 5 minutes. The same arm is always used for these measurements (see Section 7.6.5).

7.1.2.3 Visits 2 through to 6 – Main treatment phase – Dose-titration period - amended

At these study visits, the patient should attend the clinic without having taken the morning dose of study medication.

During the dose titration period of the main treatment phase, the following assessments will be performed according to the Schedule of evaluations (Section 7.1.1):

- Vital signs (blood pressure and heart rate). Blood pressure and heart rate will be measured at all visits with the patient in a sitting position, after the patient has been at rest for at least 5 minutes. The same arm is always used for these measurements (see Section 7.6.5).
- Digital ulcer net burden assessment (see Section 7.6.10)
- Recording and assessment of AEs (see Section 7.5)

- Blood samples for additional safety for patients with impaired renal and liver function at Visit 2, 3, 4, 5 as needed[77] (see Section 7.6.2)
- Blood samples for safety Visit 6 (see Section 7.6.2)
- Blood sample for PK up to 1 hour before the morning dose of study medication (see Section 7.4)
- Pregnancy test (urine) for all women of childbearing potential (Visit 3 and Visit 5) (see Section 7.6.1)
- Concomitant therapy
- Dose titration according to the titration algorithm (see Section 4.1.2)
- Dispensation of study drug and drug accountability

7.1.2.4 Visits 7 through to 11 – Main treatment phase - Maintenance period - amended

At these visits, the patient should attend the clinic without having taken the morning dose of riociguat or placebo.

During the maintenance period of the main treatment phase, the following assessments will be performed according to the Schedule of evaluations (Section 7.1.1):

- Questionnaires (Visit 8, 10, and 11) (see Section 7.6.13)
- Smoking history (Visit 10)
- Physical examination, including weight (at Visit 7 and Visit 10) (Note: BMI will be calculated automatically in the eCRF)
- Oxygen saturation measurement using forehead pulse oximetry at Visit 9 only. At Visits 10 and 11 only, oxygen saturation will be measured before and after intake of study drug (see Section 7.6.18, Table 7–2, Table 7–3, and Table 7–4)[78]
- Vital signs (blood pressure and heart rate). Blood pressure and heart rate will be measured at all visits with the patient in a sitting position, after the patient has been at rest for at least 5 minutes. The same arm is always used for these measurements (see Section 7.6.5)
- Assessment of orthostatic changes: measure blood pressure and heart rate in supine position and sitting position approximately 2 h after intake of study drug (Visit 11 only, see Section 7.6.5).[79]
- mRSS (Visits 7, 10 and 11) (see Section 7.6.6)
- Digital ulcer net burden assessment (see Section 7.6.10)
- Provision of 7-day diary to patients at Visit 7, which they will complete daily for 7 consecutive days leading up to Visit 8 (see Section 7.6.12)

[77] Per Amendment 1
[78] Per Amendment 6
[79] Per Amendment 6

- Patient and physician assessment of Raynaud's attacks, and patient to return completed 7-day diary (Visit 8) (see Section 7.6.12)
- Patient's and physician's global assessment (Visit 10) (see Section 7.6.15)
- Patient interference with skin assessment (Visit 10) (see Section 7.6.16)
- Assessment for the presence or absence of tendon friction rubs (Visit 8) (see Section 7.6.17)
- Blood samples for safety (Visit 10 and 11) (see Section 7.6.2)
- Blood sample for PK at Visit 11 up to 1 hour before the morning dose of study medication (see Section 7.4)
- Blood sample for biomarkers (Visit 8) (see Section 7.6.3)
- Pregnancy test (urine) for all women of childbearing potential (Visits 7 and 9) (see Section 7.6.1) and every 4 weeks in between as required
- 12-lead ECG for centralized reading (Visits 7, 10 and11). A standard 12-lead ECG will be obtained with the patient in the supine position after resting for at least 5 minutes (see Section 7.6.7)
- Pulmonary function tests (FVC and DL_{CO}) (Visits 7, 10 and 11) (see Section 7.6.8)
- Skin biopsy (Visit 8) (see Section 7.6.9)
- Recording and assessment of AEs (see Section 7.5)
- Concomitant therapy
- Dispensation of study drug and drug accountability

After intake of study medication (at Visit 11 only):

- Blood sample for PK: between 2 – 3 hours after administration of the morning dose of study medication (see Section 7.4)
- Vital signs (blood pressure and heart rate) within 20 minutes prior to the PK sampling with the patient in a sitting position and after the patient has been at rest for at least 5 minutes. The same arm is always used for these measurements (see Section 7.6.5).

7.1.2.5 Visit 12 (Week 52) - amended

This is the last visit of the main treatment phase, and the first visit of the long-term extension phase. At this visit, the patient should attend the clinic without having taken the morning dose of riociguat or placebo.

Before intake of study medication:

- Questionnaires (see Section 7.6.13)
- Smoking history
- Physical examination, including weight (Note: BMI will be calculated automatically in the eCRF)

- Oxygen saturation will be measured before and after intake of study drug (see Section 7.6.18, Table 7–2, Table 7–3, and Table 7–4).[80]
- Vital signs (blood pressure and heart rate). Blood pressure and heart rate will be measured at all visits with the patient in a sitting position, after the patient has been at rest for at least 5 minutes. The same arm is always used for these measurements (see Section 7.6.5)
- Assessment of orthostatic changes: measure blood pressure and heart rate in supine position and sitting position approximately 2 h after intake of study drug (see Section 7.6.5).[81]
- mRSS (see Section 7.6.6)
- Digital ulcer net burden assessment (see Section 7.6.9)
- Patient's and physician's global assessment (see Section 7.6.15)
- Patient interference with skin assessment (see Section 7.6.16)
- Tender and swollen joint count assessment (see Section 7.6.14)
- Blood samples for safety (see Section 7.6.2)
- Pregnancy test (urine) for all women of childbearing potential (see Section 7.6.1)
- 12-lead ECG for centralized reading. A standard 12-lead ECG will be obtained with the patient in the supine position after resting for at least 5 minutes (see Section 7.6.7)
- Pulmonary function tests (FVC and DL_{CO}) (see Section 7.6.8)
- Recording and assessment of AEs (see Section 7.5)
- Concomitant therapy
- Drug accountability
- Start of long-term extension phase
- Assignment of patients to treatment with riociguat via IxRS, dispensation of study drug and intake by patient

After intake of study medication:

- Blood sample for PK (see Section 7.4)
    - PK blood sample between 1 – 2 hours after administration of the morning (first) dose of study medication
    - PK blood sample between 3 – 4 hours after administration of the morning (first) dose of study medication
- Vital signs (blood pressure and heart rate) should be measured within 20 minutes prior to each PK sampling with the patient in a sitting position and after the patient has been at rest for at least 5 minutes. The same arm is always used for these measurements (see Section 7.6.5).

[80] Per Amendment 6
[81] Per Amendment 6

7.1.2.6 Visits 13 through to 17 – Long-term extension phase - Dose-titration period - amended

At these study visits, the patient should attend the clinic without having taken the morning dose of riociguat.

During the double-blind dose-titration period of the long-term extension phase, the following assessments will be performed according to the Schedule of evaluations (Section 7.1.1):

- Oxygen saturation will be measured before and after intake of study drug (see Section 7.6.18, Table 7–2, Table 7–3, and Table 7–4)[82]
- Vital signs (blood pressure and heart rate). Blood pressure and heart rate will be measured at all visits with the patient in a sitting position, after the patient has been at rest for at least 5 minutes. The same arm is always used for these measurements (see Section 7.6.5)
- Assessment of orthostatic changes: measure blood pressure and heart rate in supine position and sitting position approximately 2 h after intake of study drug (see Section 7.6.5).[83]
- Digital ulcer net burden assessment (see Section 7.6.10)
- Blood samples for safety (Visit 17; see Section 7.6.2)
- Blood sample for PK up to 1 hour before the morning dose of study drug (see Section 7.4)
- Pregnancy test (urine) for all women of childbearing potential (on-site at Visits 14 and 16) (see Section 7.6.1)
- Recording and assessment of AEs (see Section 7.5)
- Concomitant therapy
- Dose titration according to the titration algorithm (see Section 4.1.2)
- Dispensation of study drug and drug accountability

7.1.2.7 Visits 18 through Visit n (every 12 weeks) – Long-term extension phase – Open-label extension period - amended

At these study visits, the patient should attend the clinic without having taken the morning dose of riociguat.

During the open-label extension period, the following assessments and procedures will be performed according to the Schedule of evaluations (Section 7.1.1):

- Questionnaires (see Section 7.6.13)
- Smoking history
- Physical examination, including weight

[82] Per Amendment 6
[83] Per Amendment 6

- Oxygen saturation measurement using forehead pulse oximetry (see Section 7.6.18).[84]
- Vital signs (blood pressure and heart rate). Blood pressure and heart rate will be measured at all visits with the patient in a sitting position, after the patient has been at rest for at least 5 minutes. The same arm is always used for these measurements (see Section 7.6.5)
- mRSS (see Section 7.6.6)
- Digital ulcer net burden assessment (see Section 7.6.10)
- Blood samples for safety (see Section 7.6.2)
- Pregnancy test (urine) for all women of childbearing potential (every 4 weeks) (see Section 7.6.1). When pregnancy testing coincides with a visit, the test should be performed at the site, but otherwise should be performed at home.
- 12-lead ECG for centralized reading. A standard 12-lead ECG will be obtained with the patient in the supine position after resting for at least 5 minutes (see Section 7.6.7)
- Pulmonary function tests (FVC and DL_{CO}) (see Section 7.6.8)
- Recording and assessment of AEs (see Section 7.5)
- Concomitant therapy
- Dispensation of study drug and drug accountability

7.1.2.8 Termination Visit - amended

The termination visit will be performed if the patient is discontinued[85] from study medication for any reason except death or lost to follow-up, and should occur as soon as possible after the patient receives his/her last dose of study drug. Assessments and procedures at this visit include the following:

- Questionnaires (see Section 7.6.13)
- Smoking history
- Physical examination, including weight
- Oxygen saturation measurement using forehead pulse oximetry (see Section 7.6.18).[86]
- Vital signs (blood pressure and heart rate). Blood pressure and heart rate will be measured at all visits with the patient in a sitting position, after the patient has been at rest for at least 5 minutes. The same arm is always used for these measurements (see Section 7.6.5)
- mRSS (see Section 7.6.6)
- Digital ulcer net burden assessment (see Section 7.6.10)
- Tender and swollen joint count assessment (not needed after V12, see Section 7.6.14)[87]

[84] Per Amendment 6
[85] Per Amendment 4
[86] Per Amendment 6

- Patient's and physician's global assessment (not needed after V12[88], see Section 7.6.15)
- Patient interference with skin assessment (not needed after V12[89], see Section 7.6.16)
- Blood samples for safety (see Section 7.6.2)
- Pulmonary function tests (FVC and DL_{CO}) (see Section 7.6.8)
- Blood sample for biomarkers (if TV occurs before Visit 8) (see Section 7.6.3)
- Skin biopsy (if TV occurs before Visit 8) (see Section 7.6.9)
- Pregnancy test (urine) for all women of childbearing potential (if 4 weeks since the previous visit) (see Section 7.6.1)
- 12-lead ECG for centralized reading. A standard 12-lead ECG will be obtained with the patient in the supine position after resting for at least 5 minutes (see Section 7.6.7)
- Recording and assessment of AEs (see Section 7.5)
- Drug accountability
- Concomitant therapy

See also Section 7.1.2.10 for further planned assessments.

7.1.2.9 Safety follow-up visit - amended

Patients must be evaluated for safety 30 (+ 5) days after the last dose intake of study medication. Assessments will include the following:

- Physical examination, including weight
- Oxygen saturation measurement using forehead pulse oximetry (see Section 7.6.18).[90]
- Vital signs (blood pressure and heart rate). Blood pressure and heart rate will be measured at all visits with the patient in a sitting position, after the patient has been at rest for at least 5 minutes. The same arm is always used for these measurements (see Section 7.6.5)
- Recording and assessment of AEs (see Section 7.5)
- Blood samples for safety (see Section 7.6.2)
- Pregnancy test (urine) for all women of childbearing potential
- 12-lead ECG for centralized reading. A standard 12-lead ECG will be obtained with the patient in the supine position after resting for at least 5 minutes (see Section 7.6.7)
- Concomitant therapy
- Survival status (see Section 5.2.1)

---

[87] Per Amendment 1
[88] Per Amendment 1
[89] Per Amendment 1
[90] Per Amendment 6

7.1.2.10 Premature discontinuation of study drug treatment - amended

In the event of premature discontinuation of study drug treatment, patients must undergo the same procedures as outlined for the termination visit (Section 7.1.2.8) and then undergo evaluation at the safety follow-up visit, 30 (+5) days later (Section 7.1.2.9).

The reason for premature discontinuation of study drug treatment or early withdrawal from the study must be recorded in the eCRF (please refer to Section 5.2).

If the patient discontinues study drug[91] prematurely during the main treatment phase (Week 0 – Week 52) he/she will be invited to come for limited assessments (mRSS, pulmonary function test [FVC and DL_{CO} including hemoglobin measurement at the local laboratory][92], PROs, and patient's and physician's global assessment)[93] at Week 12, Week 26, Week 39 and Week 52, depending on the timing of discontinuation[94]. This will be in addition to the termination visit assessments (see Section 7.1.2.8), provided that the patient does not object to collection of study data after withdrawal of consent. If the discontinuation occurs before Visit 8 (Week 14), a skin biopsy will also be performed.

7.2 Population characteristics

7.2.1 Demographic

The following demographic data will be recorded:

- Date of birth (month and year) (age)
- Sex
- Ethnicity
- Smoking status, including number of cigarettes per day
- Alcohol consumption

7.2.2 Medical history

Medical history findings (ie, previous diagnoses, diseases or surgeries) meeting all criteria listed below will be collected:

- Pertaining to the study indication
- Started before signing of the informed consent
- Considered relevant to the study
- Medical history related to concomitant therapy

Detailed instructions on the differentiation between (i) medical history and (ii) AEs can be found in Section 7.5.1.1.

7.3 Efficacy - amended

Primary efficacy outcome measure

[91] Per Amendment 1
[92] Per Amendment 4
[93] Per Amendment 5
[94] Per Amendment 4

- Change in mRSS from baseline to Week 52

Secondary efficacy outcome[95] measures[96]

Key secondary endpoint:

- CRISS at Week 52[97], consisting of five variables: mRSS, FVC % predicted, physician and patient global assessments, and HAQ-DI score (from SHAQ patient reported outcome)

Further secondary endpoints:

- HAQ-DI domain (separately from the SHAQ as part of the calculation of the CRISS algorithm).
- Patient's global assessment
- Physician's global assessment
- Change in FVC (forced vital capacity) % predicted

Further clinical outcomes (to be adjudicated by an Independent Central Adjudication Committee)

- New renal crisis
- Worsening of cardiac disease, defined as new or worsened clinically symptomatic and significant heart disease, considered secondary to dcSSc, including congestive heart failure requiring hospitalization, new onset pulmonary hypertension requiring treatment, pericardial disease requiring intervention or exhibiting clinical decompensation, and arrhythmias and/or conduction defects requiring treatment.
- Worsening of gastrointestinal disease requiring hospitalization or new requirement for parenteral nutrition
- Critical digital ischemia requiring hospitalization, or digital gangrene

Additional information regarding the events to be adjudicated may be requested to be sent to the Adjudication Committee. All events listed above, in addition to all cases of death, regardless of causality or seriousness will be reviewed by the Adjudication Committee.

Other exploratory measures:[98]

- mRSS progression rate (defined as increase in mRSS by > 5 units and > 25% from baseline) and mRSS regression rate (defined as decrease in mRSS by > 5 units and > 25% from baseline)
- HRQoL using SF-36 and the SHAQ.
- Digital ulcer net burden (defined as total number of ulcers at time point minus number of ulcers at baseline) and proportion of patients who do not develop new ulcers

---

[95] Per Amendment 1
[96] Per Amendment 4
[97] Per Amendment 5
[98] Per Amendment 4

- Change in DL_{CO} (carbon monoxide diffusing capacity) % predicted
- Need for Rescue[99] Therapy (see definition in Section 6.9)
- All-cause Mortality
- Gastrointestinal involvement as assessed by University of California, Los Angeles, Scleroderma Clinical Trial Consortium Gastrointestinal Scale (UCLA SCTC GIT) 2.0 instrument
- Patient-Reported Outcomes Measurement Information System (PROMIS)-29 (subset of patients)
- Patient interference with skin
- Presence or absence of tendon friction rubs
- Pain visual analog scale (VAS)
- Change in BMI
- Joint involvement using tender and swollen joint counts
- New items for Raynaud's response index excluding items included in secondary outcome measures
    - Patient and physician assessment of Raynaud's phenomenon; pain, numbness, and tingling during a Raynaud's phenomenon attack; Raynaud's condition score, average number of attacks/day; and duration of attacks

Endpoints long-term extension phase (Week 52 to study completion)

- mRSS
- Pulmonary function testing
- PROs / HRQoL
- Worsening end-organ disease (cardiac, renal, pulmonary, gastrointestinal & digital ischemia)

7.4 Pharmacokinetics / pharmacodynamics - amended

At these study visits the patients should present themselves to the hospital without taking the morning dose of study medication. The trough PK sample should always be obtained at up to 1 hour before administration of the morning dose of study medication and approximately 5 to 15 hours after the evening dose[100].

It is essential that the exact time of blood sampling and time of dose administration of the 3 preceding doses of study medication are documented accurately in the eCRF. For samples taken before riociguat administration, also the time of the first dose taken at the visit needs to be documented[101].

[99] Per Amendment 1
[100] Per Amendment 5
[101] Per Amendment 5

For investigation of exposure behavior, plasma concentrations of BAY63-2521 and its metabolite (M1) will be determined at the times given below using a sparse sampling approach in all participating patients. If the investigator decides to take additional PK samples, those may be used for PK analysis as well.[102]

Blood samples for PK will be taken at the following times:

Profile day (Visit 1 and Visit 12)

- 2 hours **after** administration of the morning dose of study medication[103]
- 3 – 4 hours **after** administration of the morning dose of study medication

Visits 2 through to 6 and Visits 13 through to 17

- Up to 1 hour **before** administration of morning dose of study medication (trough)

Visit 11

- Up to 1 hour **before** administration of morning dose of study medication (trough)
- 2 hours **after** administration of morning dose of study medication (peak)[104]

For details regarding the timing of PK sampling and flow charts detailing the order of procedures at these visits, please refer to Section 7.1.1.

Plasma concentration time points will be compared to the available plasma concentration profiles in healthy volunteers and selected patient populations. Pharmacokinetic and exposure-response analysis might be performed using population approaches (popPK (population PK) and popPK/pharmacodynamic (PD), eg, by non-linear mixed effect modeling). Analysis and report will be done under separate cover. This evaluation might be started prior to database lock. If this is applicable, appropriate measures will be taken to maintain blinding of the study team, eg, data will be stored separately, and members of the study team will neither have access to the randomization list nor to individual data.[105]

Sample collection, processing and storage

Further details on collection, labeling, storage and shipping of samples are provided in a separate laboratory manual.

Pharmacokinetic (PK) samples will be analyzed under the responsibility of the Bayer HealthCare Bioanalytics Laboratory, Bayer Pharma AG, GDD-GED-DMPK Bioanalytics, 42096 Wuppertal, Germany. The bioanalyst will be unblinded and have access to the randomization list.[106]

Any residual PK samples will be destroyed at end of study.

[102] Per Amendment 5
[103] Per Amendment 6
[104] Per Amendment 6
[105] Per Amendment 5
[106] Per Amendment 5

7.5 Safety

7.5.1 Adverse events

7.5.1.1 Definitions - amended

Definition of adverse event (AE)

In a clinical study, an AE is any untoward medical occurrence (ie, any unfavorable and unintended sign [including abnormal laboratory findings], symptom or disease) in a patient or clinical investigation subject after providing written informed consent for participation in the study. Therefore, an AE may or may not be temporally or causally associated with the use of a medicinal (investigational) product.

A surgical procedure that was planned prior to the start of the study by any physician treating the patient should not be recorded as AE (however, the condition for which the surgery is required may be an AE).

In the following differentiation between medical history and AEs, the term "condition" may include abnormal eg, physical examination findings, symptoms, diseases, laboratory, ECG.

- Conditions that started before signing of informed consent and for which no symptoms or treatment are present until signing of informed consent are recorded as medical history (eg, seasonal allergy without acute complaints).
- Conditions that started before signing of informed consent and for which symptoms or treatment are present after signing of informed consent, at *unchanged intensity*, are recorded as medical history (eg, allergic pollinosis).
- Conditions that started or deteriorated after signing of informed consent will be documented as AEs. This includes any conditions related to the underlying disease[107].

Definition of serious adverse event (SAE)

An SAE is classified as any untoward medical occurrence that, at any dose, meets any of the following criteria (a – f):

a. Results in death

b. Is life-threatening

The term 'life-threatening' in the definition refers to an event in which the subject was at risk of death at the time of the event, it does not refer to an event which hypothetically might have caused death if it were more severe.

[107] Per Amendment 5

c. Requires inpatient hospitalization or prolongation of existing hospitalization

A hospitalization or prolongation of hospitalization will not be regarded as an SAE if at least one of the following exceptions is met:

- The admission results in a hospital stay of less than 12 hours
- The admission is pre-planned
(ie, elective or scheduled surgery arranged prior to the start of the study)
- The admission is not associated with an AE
(eg, social hospitalization for purposes of respite care).

However, it should be noted that invasive treatment during any hospitalization may fulfill the criterion of 'medically important' and as such may be reportable as an SAE dependent on clinical judgment. In addition, where local regulatory authorities specifically require a more stringent definition, the local regulation takes precedence.

d. Results in persistent or significant disability / incapacity

Disability means a substantial disruption of a person's ability to conduct normal life's functions.

e. Is a congenital anomaly / birth defect

f. Is another medically important serious event as judged by the investigator

7.5.1.2 Classifications for adverse event assessment

All AEs will be assessed and documented by the investigator according to the categories detailed below.

7.5.1.2.1 Seriousness

For each AE, the seriousness must be determined according to the criteria given in Section 7.5.1.1.

7.5.1.2.2 Intensity

The intensity of an AE is classified according to the following categories:

- Mild
- Moderate
- Severe

7.5.1.2.3 Causal relationship

The assessment of the causal relationship between an AE and the administration of treatment is a clinical decision based on all available information at the time of the completion of the eCRF.

The assessment is based on the question whether there was a "reasonable causal relationship" to the study treatment in question.

Possible answers are "yes" or "no"

An assessment of “no” would include:

1. The existence of a clear alternative explanation, eg, mechanical bleeding at surgical site.

or

2. Non-plausibility, eg, the subject is struck by an automobile when there is no indication that the drug caused disorientation that may have caused the event; cancer developing a few days after the first drug administration.

An assessment of “yes” indicates that there is a reasonable suspicion that the AE is associated with the use of the study treatment.

Important factors to be considered in assessing the relationship of the AE to study treatment include:

- The temporal sequence from drug administration: The event should occur after the drug is given. The length of time from drug exposure to event should be evaluated in the clinical context of the event.
- Recovery on drug discontinuation (de-challenge), recurrence on drug re-introduction (re-challenge):
- Subject’s response after de-challenge or subjects response after re-challenge should be considered in the view of the usual clinical course of the event in question.
- Underlying, concomitant, intercurrent diseases:
  Each event should be evaluated in the context of the natural history and course of the disease being treated and any other disease the subject may have.
- Concomitant medication or treatment:
  The other drugs the subject is taking or the treatment the subject receives should be examined to determine whether any of them may be suspected to cause the event in question.
- The pharmacology and PK of the study treatment:
  The PK properties (absorption, distribution, metabolism and excretion) of the study treatment, coupled with the individual subject’s pharmacodynamics should be considered.

Causal relationship to protocol-required procedure(s)

The assessment of a possible causal relationship between the AE and protocol-required procedure(s) is based on the question whether there was a “reasonable causal relationship” to protocol-required procedure(s).

Possible answers are “yes” or “no”

7.5.1.2.4 Action taken with study treatment

Any action on study treatment to resolve the AE is to be documented using the categories listed below.

- Drug withdrawn
- Drug interrupted
- Dose reduced
- Dose not changed
- Dose increased
- Not applicable
- Unknown

7.5.1.2.5 Other specific treatment(s) of adverse events

- None
- Remedial drug therapy
- Other

7.5.1.2.6 Outcome

The outcome of the AE is to be documented as follows:

- Recovered/resolved
- Recovering/resolving
- Recovered/resolved with sequelae
- Not recovered/not resolved
- Fatal
- Unknown

7.5.1.3 Assessments and documentation of adverse events

The investigator has the obligation to report AEs. All non-serious events will be assessed and recorded during the specified observational phase (from signing the informed consent form up to the follow-up visit 30 (+5) days after last study medication intake), whether believed to be related or unrelated to the treatment. AE forms will be included in the eCRFs. The record will include clinical symptoms or final diagnosis when available, date of appearance, duration, severity and relationship to treatment. A record will also be kept of the action taken and the follow-up until resolution of the AE.

7.5.1.4 Reporting of serious adverse events

The definition of SAEs is given in Section 7.5.1.1.

Investigator's notification of the sponsor

All investigators will be thoroughly instructed and trained on all relevant aspects of the investigator's reporting obligations for SAEs. This information, including all relevant contact

details, is summarized in the investigator site file. This information will be updated as needed.

All SAEs occurring during the observation period defined in Section 7.5.1.3 must immediately (within 24 hours of the investigator's awareness) be reported to the recipient detailed in the manual. An SAE form must also be completed within 24 hours of the investigator awareness and forwarded to the designated recipient. Each SAE must be followed up until resolution or stabilization by submission of updated reports to the designated recipient.

SAEs occurring after the protocol-defined observation period will be processed by the sponsor according to all applicable regulations.

Notification of the IECs / IRBs

Notification of the IECs / IRBs about all relevant events (eg, SAEs, suspected, unexpected, serious adverse reactions [SUSARs]) will be performed by the sponsor and/or by the investigator according to all applicable regulations.

Notification of the authorities

The processing and reporting of all relevant events (eg, SAEs, SUSARs) to the authorities will be done by the sponsor according to all applicable regulations.

Sponsor's notification of the investigational site

The sponsor will inform all investigational sites about reported relevant events (eg, SUSARs) according to all applicable regulations.

7.5.1.5 Expected adverse events

For this study, the applicable reference document is the most current version of the IB/summary of product characteristics.

Overview listings of frequent events that have occurred so far in the clinical development are shown in the current IB. If relevant new safety information is identified, the information will be integrated into an update of the IB and distributed to all participating sites.

The expectedness of AEs will be determined by the sponsor according to the applicable reference document and according to all local regulations.

In this study, the following events are outcome events and/or consistent with the underlying condition and will be considered as disease related in the defined study population:

- New renal crisis
- Worsening of cardiac disease considered secondary to dcSSc
- Worsening of gastrointestinal disease requiring hospitalization or new requirement for parenteral nutrition
- Critical digital ischemia requiring hospitalization, or digital gangrene

For the purposes of this trial, these events will not be subject to systematic unblinding and expedited reporting process, if reported as serious adverse drug reactions. They will be captured in the Global Pharmacovigilance database, in the eCRF and undergo regular central adjudication and unblinded DMC review.

7.5.1.6 Adverse events of special safety interest

Symptomatic hypotension and serious hemoptysis have been defined as AEs of special interest and must be documented accordingly in the corresponding AEs of special safety interest pages in the eCRF. Events of asymptomatic hypotension and non-serious hemoptysis should not automatically be upgraded by the reporting investigator to serious. Declaration of an event as serious should only occur when 1 or more of the serious criteria (as defined in Section 7.5.1.1) are applicable.

7.5.1.7 Overdose of Study Medication

In case of overdose, standard supportive measures should be adopted as required. In case of pronounced hypotension, active cardiovascular support may be required. If symptoms develop after the investigational drug has been administered, any therapy that becomes necessary has to be guided by the predominant symptoms. Patients must remain under medical supervision until all relevant adverse effects have resolved in the event of overdose; patient continuation remains at the discretion of the investigator.

7.5.2 Pregnancies

The investigator must report to the sponsor any pregnancy occurring in a study subject or his female partner during the subject’s participation in this study. The report should be submitted within the same timelines as an SAE, although a pregnancy per se is not considered an SAE.

For a study subject, the outcome of the pregnancy should be followed up carefully, and any outcome of the mother or the child should be reported.

Bayer usually does not gather information of drug exposure via the father, however, if those cases are reported, all efforts should be made to obtain similar information on course and outcome, subject to the partner’s consent.

For all reports, the forms provided are to be used.

7.6 Other procedures and variables

7.6.1 Pregnancy testing

Pregnancy testing is to be performed at the screening visit (serum only), Visit 1 (Day 0; urine) and at 4-weekly intervals thereafter (urine), at a termination visit, and until 30 (+ 5) days after the patient stops intake of study drug. If the interval falls on a visit date, pregnancy testing is to be performed locally at the site, while at all other dates the patient is to perform a urine dipstick test at home. The results are to be documented in the eCRF and in the patient file.

The result of every home urine pregnancy test should be actively requested by the site as soon as the test is due. The patient must report a positive result to the site without delay. The results are to be documented in the eCRF and in the patient file in a timely manner. It is not acceptable to obtain the result only at the next scheduled visit at the site. In the event of pregnancy, a referral to a gynecologist for confirmation of pregnancy must be organized as soon as possible. In addition, the pregnancy must be reported to the sponsor as described in Section 7.5.2 and further consequences with regard to ongoing participation in the study must

be discussed with the patient. In the event uncertainties about a pregnancy test outcome exist, the patient should contact the site immediately to discuss further steps (eg, exclusion of pregnancy by serum pregnancy test).

Based on country-specific regulations, further measurements may be performed.

Women of childbearing potential must agree to use adequate contraception when sexually active.

"Adequate contraception" is defined as any combination of at least 2 effective methods of birth control, of which at least 1 is a physical barrier (eg, condom with hormonal contraception like implants or combined oral contraceptives, condom with intrauterine devices). Patients must agree to utilize 2 methods of contraception simultaneously, of which at least 1 should be a physical barrier (eg, condom, diaphragm or cervical cap).

7.6.2 Laboratory parameters - amended

The following safety laboratory parameters will be analyzed in a central laboratory (apart from coagulation tests, which will be analyzed at a local laboratory) at the screening visit (Visit 0), Visit 1 (Day 0), Visit 6 (Week 10), Visit 10 (Week 26)[108], Visit 11 (Week 39), Visit 12 (Week 52), Visit 17 (Week 62), every 12 weeks during the Open-label extension period, termination visit, and safety follow-up visit:

- Hematology: WBC differential count, erythrocytes, hemoglobin, hematocrit and platelets
  The hemoglobin value required for the pulmonary function tests performed at visits with limited assessments (see Section 7.1.2.10) will be measured at the local laboratory. The hemoglobin value will be entered in the eCRF and used for the calculation of predicted DL_{CO} only. [109]
- Coagulation tests: activated partial thromboplastin time (aPTT) and international normalized ratio (INR) (to be performed at a local laboratory)
- Clinical chemistry: aspartate aminotransferase (AST), alanine aminotransferase (ALT), alkaline phosphatase (AP), gamma glutamyl transferase (GGT), CK, sodium, potassium, magnesium, calcium, phosphate, creatinine, urea, uric acid, total bilirubin, total protein, serum albumin.

Patients with isolated AST or ALT >3xULN or bilirubin >2xULN at screening and/or baseline will have additional analysis of coagulation tests, AST, ALT, AP, GGT, total bilirubin and serum albumin until normalization and/ or stabilization at Visit 2 (Day 14), Visit 3 (Day 28), Visit 4 (Day 42), Visit 5 (Day 56), as needed. Worsening of the parameters in the order of 2-fold from baseline values will be considered clinically significant [22] and additional diagnostic procedures may be requested[110]. See Section 5.2.1.2 for discontinuation criteria related to liver function.[111]

Patients entering the trial with eGFR 15-29 mL/min/1.73m^2 will be undergo additional serum creatinine and eGFR calculation until normalization and/ or stabilization at Visit 2 (Day 14), Visit 3 (Day 28), Visit 4 (Day 42), and Visit 5 (Day 56), as needed. A decrease in eGFR

[108] Per Amendment 1
[109] Per Amendment 4
[110] Per Amendment 1
[111] Per Amendment 5

equal or greater than 5ml/min/1.73m^2 in these patients (eGFR 15-29 ml/min/1.73m^2) during the study or a reduction of 25% or more in 12 months will be considered clinically significant[112] [23,24]. See Section 5.2.1.2 for discontinuation criteria related to eGFR values.[113]

At latest, at the end of the study when all planned analyses are completed, all blood samples will be destroyed. The name and address for the central laboratory service provider can be found in the documentation supplied by the vendor.

Note: One re-assessment of laboratory parameters is allowed during the screening phase to re-assess the eligibility of patients.

7.6.3 Exploratory Biomarkers - amended

Exploratory biomarkers will be evaluated at Visit 1 (Day 0) and Visit 8 (Week 14) and at a termination visit if this visit occurs before Visit 8. Skin biopsy and blood samples (serum and plasma) will be collected and processed to measure the following biomarkers (see Section 7.6.9 for details of skin biopsy):

- Skin biomarkers:
    - CCI [redacted]
    - [redacted]
- Serum/plasma biomarkers:
    - CCI [redacted]
    - [redacted]

Blood samples for inflammatory, vascular, and fibrosis as well as skin samples will be analyzed by an external central laboratory; details will be provided in the Laboratory Manual.

The evaluation of biomarkers might be started prior to database lock; if this is applicable, appropriate measures will be taken to maintain blinding of the study team (see also Section 6.5 and Section 7.4). [114]

The results of the biomarker analysis will be reported separately to the clinical study report.[115]

[112] Per Amendment 1
[113] Per Amendment 5
[114] Per Amendment 5
[115] Per Amendment 5

7.6.4 Autoantibody screen

Blood samples will be sent to the central laboratory at the Screening Visit (Visit 0) for anti-nuclear antibody immunofluorescence and to detect the presence of SSc-associated anti-nuclear autoantibodies, such as anti-Scl-70 (anti-topoisomerase 1), anti-RNA polymerase III, and anti-centromere.

7.6.5 Blood pressure and heart rate measurement - amended

Blood pressure and heart rate will be measured at all visits with the patient in a sitting position, after the patient has been at rest for 5 minutes. The same arm is always used for these measurements.

At certain visits where a blood sample for PK is scheduled after the morning dose of study medication, an additional vital signs measurement (blood pressure and heart rate) will be taken within 20 minutes prior to PK sampling. Please refer to Section 7.1.1.

Methodology: Noninvasive measurement preferably with a validated electronic device in accordance with published guidelines (eg, American Heart Association Recommendations for Blood Pressure Measurement in Humans and Experimental Animals [25]).

Assessment of orthostatic changes[116]

To assess orthostatic changes, blood pressure and heart rate will be measured with the patient in a supine position, after the patient has rested for 5 minutes. Immediately thereafter, the patient should sit up and blood pressure and heart rate will be measured after the patient has rested for 2 minutes.

These measurements should be performed approximately 2 hours after study medication intake.

Frequency: Visit 11 (Week 39), Visit 12 (Week 52), during the Dose-Titration Period of the Long-Term Extension Phase (Visits 13 to 17).

7.6.6 Modified Rodnan skin scoring (mRSS)

The mRSS is a validated physical examination method for estimating skin induration. It correlates with biopsy measures of skin thickness and reflects prognosis and visceral involvement, especially in early disease. It is scored on 0 (normal) to 3+ (severe induration) ordinal scales over 17 body areas, with a maximum score of 51 and is used to categorize severity of SSc.[26] It has been extensively used as a primary and secondary outcome measure in randomized clinical trials.[27,28]

This assessment should be performed by a physician who is experienced and trained in skin scoring. To prevent inter-observer variability, the same physician must perform skin scoring for the same patient throughout the entire study.

Frequency: Screening Visit (Visit 0), Visit 7 (Week 12), Visit 10 (Week 26), Visit 11 (Week 39), Visit 12 (Week 52), and every 3 months thereafter in the long-term extension phase and termination visit.

[116] Per Amendment 6

7.6.7 Electrocardiogram

Standard ECGs (12-lead ECG) will be recorded with the patient in the supine position after the patient has rested for at least 5 minutes.

All ECG printouts will be identified with the patient identification as well as date and time of recording and will be attached to the patient's file. All ECG printouts will be examined locally by the investigator on the day of recording for safety and quality. Any clinically relevant abnormality will be documented as an AE.

Electrocardiograms will then be transferred electronically and assessed centrally by an expert in cardiology who will provide expert assessment and interpretation of all ECGs obtained.

The name and address for the ECG service provider can be found in the documentation supplied by the vendor. Electrocardiogram procedures will be described in a separate manual.

Frequency: screening visit (Visit 0), Visit 1 (Day 0), Visit 7 (Week 12), Visit 10 (Week 26), Visit 11 (Week 39), Visit 12 (Week 52), every 3 months (12 weeks) in the long-term extension phase, termination visit and safety follow-up visit.

7.6.8 Pulmonary function testing

The following lung function measurements will be performed locally:

- FVC and % of predicted
- DL_{CO} (mmol/min/kPa) and % of predicted (hemoglobin-corrected)

Printouts from the analyzer must be stored in the patient file and the measured values transferred to the eCRF.

The hemoglobin value required for the pulmonary function tests performed at visits with limited assessments (see Section 7.1.2.10) will be measured at the local laboratory. The hemoglobin value will be entered in the eCRF and used for the calculation of predicted DL_{CO} only.[117].

Frequency: screening visit (Visit 0), Visit 7 (Week 12), Visit 10 (Week 26), Visit 11 (Week 39), Visit 12 (Week 52), and every 3 months (12 weeks) in the long-term extension phase and termination visit.

7.6.9 Skin biopsy

As stated in Section 7.6.3 above, biomarker analysis will be performed using skin and blood samples.

Skin biopsies will be obtained from the mid dorsal surface of the forearm (150 ± 20 mm proximal to the ulnar styloid) at Visit 1 (Day 0) and at Visit 8 (Week 14) and at a termination visit if this visit occurs before Visit 8.

If affected skin is located in that area, then the affected skin should be used. A biopsy should be performed regardless of whether or not affected skin is present. The skin biopsy obtained after treatment should be taken from the same arm, approximately 25 mm distance away from the initial biopsy, so as to avoid healing of the initial biopsy interfering with mRNA measurement. The location of the biopsy (right or left forearm) and distance from the ulnar

[117] Per Amendment 4

styloid will be documented. If in the opinion of the investigator the mid dorsal forearm is not appropriate, another location can be chosen. The alternative site and the reason for changing the site will be documented.

At each time point (Visit 1 and Visit 8), two 4-mm skin punch biopsies next to each other will be obtained (one for RNA extraction and the other for histology/immunohistochemistry). The skin to be biopsied will be cleaned before biopsy and the remainder of the procedure will be performed using clean technique, per standard dermatological practice. The skin at the biopsy site will be marked by drawing a circle of about 10 mm diameter using a dermatological ink skin marker.

Skin will be biopsied with a 4-mm Baker style skin punch, provided by the Sponsor. The punch will be centered at the 10-mm diameter circle. The biopsy site will be closed by Steri-strips or sutures (at the discretion of the investigator) to provide hemostasis and ensure proper healing.

At the discretion of the investigator, patients may be instructed to apply a small amount of antibiotic ointment and a sterile bandage to the site daily; these supplies will be provided by the investigator. At the discretion of the investigator the biopsy site can be dressed with an appropriate dressing. If sutures are used the sutures will be removed after 5 to 14 days.

7.6.10 Digital ulcer net burden

Digital ulcers are defined as a full thickness skin lesion with loss of epithelium including lesions covered by eschar. Ulcers should be > 3mm in maximal diameter. Healing is defined by re-epithelialization with loss of pain and exudate. Pitting scars and hyperkeratotic lesions are always excluded.

Digital ulcer net burden will be assessed by the following methods:

- Ulcer count – this ideally will be performed by the same healthcare professional at every visit:
  - Total ulcer counts
  - Distal counts: distal (fingertip) – any ulcer including skin area distal to proximal interphalangeal (PIP) joint
- Ulcer burden:
  - Number of ulcers at time point minus baseline number of ulcers
- Visual analog score (VAS) for patient-reported severity of digital ulcers as part of the SHAQ (see section 7.6.13)

Frequency: Digital ulcer net burden assessment at every visit. VAS as part of SHAQ: Visit 1 (Day 0), Visit 8 (Week 14), Visit 10 (Week 26), Visit 11 (Week 39), Visit 12 (Week 52), Visit 18 (week 64), and every 3 months thereafter in the long-term extension phase and termination visit.

7.6.11 Digital gangrene

Digital gangrene will be recorded as a separate event and may co-exist with digital ulcers that affect the same digit and fulfil the criteria in section 7.6.10 above.

Gangrene is defined as diffuse necrosis (pathologic death of deeper, eg, subcutaneous structures) due to obstruction, loss, or diminution of blood supply; it is of black color and

painful, and may be localized to parts of the finger or involve an entire finger. It may be wet or dry, reflecting a degree of adjacent tissue perfusion, time course of necrosis and presence or type of associated secondary infection.

7.6.12 Raynaud's attacks assessment

Raynaud's attacks will be assessed using the composite of the following 6 individual outcome measures in order to minimize the measurement variability and placebo response: Raynaud's condition score, patient assessment of Raynaud's phenomenon, physician assessment of Raynaud's phenomenon, attack symptoms, duration of attacks, and average number of attacks per day.[29]

The Raynaud's condition score is a daily patient self-assessment of Raynaud's phenomenon activity using a 0-10 ordinal scale from 'no difficulty' to 'extreme difficulty'. It incorporates the cumulative frequency, duration, severity and impact of Raynaud's phenomenon attacks, reflecting the overall degree that Raynaud's has affected use of the patient's hands.[30]

The Raynaud's condition score, details of the frequency and duration of Raynaud's attacks, and attack symptoms of pain, numbness and tingling, each represented by a 0 – 100 VAS, will be incorporated into the daily diary that patients will be asked to complete for 7 days at the time points detailed below.

The patient and physician assessment measures the severity of Raynaud's phenomenon in the past week using a 0–100 VAS and will be assessed at the clinic visits outlined below.

Frequency: Daily diary to be completed by patient for 7 consecutive days leading up to Visit 1 (Day 0), and 7 consecutive days leading up to Visit 8 (Week 14). The patient and physician assessments of Raynaud's phenomenon will be performed at Visit 1 and Visit 8.

7.6.13 Patient-reported outcomes (PROs) / Health-related quality of life (HRQoL) questionnaires

Three PROs—the SF-36 v2.0, SHAQ, and UCLA SCTC GIT 2.0—will be completed by all patients in the study. The PROMIS-29 PRO will be completed by patients in a subset of sites belonging to English-speaking countries as this instrument has not yet undergone translation into multiple languages. To minimize bias, patients will be assured that all data will be treated confidentially, will not be reported to their patient files, and that the answers will not have any influence on study drug treatment. In addition, patients will complete the questionnaires on their own, or with the help of relatives, friends or study staff if restricted by digital lesions, before being seen by the clinician at the respective visit. Subsequently, a member of the site investigator's team will enter the responses into the eCRF.

Short Form 36 (SF-36) Questionnaire v2.0

The SF-36 is a multi-purpose, generic, HRQoL instrument that is composed of 1 multi-item scale that assesses 8 health concepts: physical function, physical role limitations, bodily pain, general health perceptions, vitality, social functioning, role limitations due to emotional problems, and emotional well-being.[31] The instrument has 36 items and requires approximately 5 minutes to complete.

Scleroderma Health Assessment Questionnaire (SHAQ)

The SHAQ consists of 8 domains from the HAQ-DI, a HRQoL instrument that measures self-reported function in 8 domains of activity in 20 weighted responses and a VAS of pain experienced in the past week. It additionally measures 5 domains specific to scleroderma using a continuous VAS: Raynaud's phenomenon, digital tip ulcers, lung symptoms, gastrointestinal symptoms, and a global patient assessment.[32] The VAS subscales of the SHAQ were shown to be significantly correlated with objective parameters,[32] and was responsive to change in a cohort and in a Raynaud's phenomenon trial in SSc.[30, 32] The SHAQ requires approximately 5 minutes to complete.

University of California, Los Angeles, Scleroderma Clinical Trial Consortium Gastrointestinal Scale (UCLA SCTC GIT) 2.0

The UCLA SCTC GIT 2.0 instrument is a self-reported PRO consisting of 34 items, which are revised from the 52-item instrument SSC-GIT version 1.0, and 7 multi-item scales that include reflux, distension/bloating, diarrhea, fecal soilage, constipation, emotional well-being, and social functioning. The validity and reliability of this tool has been supported in a study performed in individuals with SSc, which also demonstrated good symptom correlation with gastrointestinal diagnoses.[33] The UCLA SCTC GIT 2.0 requires approximately 2 minutes to complete.

Patient-Reported Outcomes Measurement Information System (PROMIS)-29

The PROMIS-29 is a validated instrument to measure the health status of SSc patients, demonstrating moderate to high correlation with other instruments validated in SSc, including the SF-36 physical component score and HAQ-DI.[34] It incorporates 7 core domains from the PROMIS questionnaire, which specifically relate to physical, mental, and social health aspects of chronic illness: pain, fatigue, depression, anxiety, sleep, and physical function, as well as one 11-point rating scale for pain intensity.[34] It contains 8 items with 29 weighted responses in total, and requires approximately 5 minutes to complete.

Frequency of all PROs: Visit 1 (Day 0), Visit 8 (Week 14), Visit 10 (Week 26), Visit 11 (Week 39), Visit 12 (Week 52), Visit 18 (week 64), and every 3 months thereafter in the main treatment phase and long-term extension phase and at a termination visit.

7.6.14 Tender and swollen joint count

This physician-reported tool evaluates 28 joints for swelling and tenderness. This outcome measure should be performed by the same physician to assess the burden of joint disease from SSc-associated polyarthritis and myopathy.

A sample form is shown in Appendix 14.3

Frequency: Screening Visit (Visit 0) and Visit 12 (Week 52) and termination visit (not needed after V12).

7.6.15 Patient's and physician's global assessment - amended

This tool incorporates a self-assessment and physician assessment on the patient's overall health in the prior 1 week using a 0–10 ordinal scale and a rating of overall SSc-related health transition compared with 1 month prior and 1 year prior.

Frequency: Visit 1 (Day 0), Visit 10 (Week 26) and Visit 12 (Week 52), termination visit (not needed after V12), and visits with limited assessments.[118]

7.6.16 Patient interference with skin assessment

This self-assessment evaluates the degree in which skin involvement has interfered with the patient's daily activities over the last month on an ordinal scale of 0 (does not limit activity) to 10 (very severe limitation).

Frequency: Visit 1 (Day 0), Visit 10 (Week 26) and Visit 12 (Week 52) and termination visit (not needed after V12).

7.6.17 Tendon friction rubs

Anatomical sites including the hands, wrists, elbows, shoulders, knees and ankles will be examined for the presence or absence of tendon friction rubs at the two time points detailed below.

Frequency: Visit 1 (Day 0) and Visit 8 (Week 14).

7.6.18 Oxygen saturation measurement using forehead pulse oximetry[119]

Oxygen saturation measurements will be performed in all subjects using non-invasive forehead pulse oximetry expressing the SpO_2 (peripheral oxygen saturation) percentage. At visits with blood pressure measurements to assess orthostatic changes, oxygen saturation will be measured pre-dose and post-dose (see Table 7–2, Table 7–3, and Table 7–4). At all other visits, oxygen saturation will be measured pre-dose only.

If the patient receives supplemental oxygen, the amount [L/min] will be recorded in the eCRF.

Frequency: Visit 9 (Week 20), Visit 10 (Week 26), Visit 11 (Week 39), Visit 12 (Week 52), Visits 13 to 17 (Week 54 to 62), Visit 18 to Visit n (Open-label Extension Period), Termination Visit, and 30-day Safety Follow-up.

7.7 Appropriateness of procedures / measurements

All safety and efficacy variables, as well as the methods to measure them, are standard variables/ methods in clinical studies and/ or clinical practice. They are widely used and generally recognized as reliable, accurate and relevant.

8. Statistical methods and determination of sample size

8.1 General considerations

All variables will be analyzed descriptively with appropriate statistical methods: categorical variables by frequency tables and continuous variables by sample statistics, ie, mean, standard deviation, minimum, median, quartiles (if data are clearly non-normal) and maximum.

If not mentioned otherwise, all statistical tests will be performed with a type I two-sided error rate of α=5%.

[118] Per Amendment 5
[119] Per Amendment 6

Statistical analyses will be performed using SAS (Statistical Analysis System); the version used will be specified in the statistical analysis plan (SAP).

8.2 Analysis sets - amended

Efficacy and safety analyses will be performed in patients valid for the full analysis set (FAS). This is defined as all patients randomized and treated with study medication. Investigators will be instructed not to make the call to the IxRS system until they are certain the patient is valid to be treated with study medication, on the day that study medication is to start. In that way, we expect all patients who are randomized also to be treated, so the FAS population would then be consistent with the full intent-to-treat (ITT) definition.

For the primary and secondary exploratory efficacy and safety endpoints, as a supportive analysis, a per protocol set (PPS) will also be defined, including patients who meet the major inclusion and exclusion criteria at randomization that may affect efficacy, who are not taking excluded concomitant medications during the study that could have an effect on efficacy (not including rescue[120] medication after Week 26), have the mRSS assessed at baseline and at least once during the main treatment phase and who are at least 80% compliant with study medication. A full definition of valid for PPS will be given in the SAP.

8.3 Variables

Efficacy variables are provided in Section 7.3 and safety variables are described in Section 7.5.

8.4 Statistical and analytical plans

8.4.1 Demographic and other baseline characteristics

Demographic variables and baseline characteristics will be summarized by treatment group for both analysis populations, that is, the FAS and PPS.

Medical history findings and AEs will be coded by Medical Dictionary for Regulatory Activities (MedDRA) codes and medications by Anatomical Therapeutic Chemical (ATC) codes (World Health Organization Drug Dictionary [WHO-DD]).

8.4.2 Efficacy - amended

In the efficacy analyses, countries and centers will be clustered by geographic region. The decision on country pooling will be made before unblinding. Statistical analyses will be adjusted for these countries / geographic regions.

8.4.2.1 Primary endpoint[121]

The primary analysis will be the change in mRSS from baseline to all assessments post baseline up to Week 52 using mixed model repeated measures (MMRM) with baseline mRSS as a covariate, fixed effects treatment arm and region, the interaction effect between study visit and treatment arm, and patient specific random effects to account for both heterogeneity among patients and correlation among measurements taken on the same patient with an unstructured covariance assumption. The main estimand of interest is the treatment difference

[120] Per Amendment 1
[121] Per Amendment 4

between the riociguat and placebo treatment arms at Week 52. The null hypothesis to be tested is no difference between riociguat and placebo at this visit.

The model statement is:

$$Y_{ij} = \mu + \beta X_i + t_k + r_l + v_j + (tv)_{jk} + s_i + \varepsilon_{ij}$$

where Y_{ij} is the change from baseline in mRSS to visit j for subject i that is in treatment group k and region l; μ is the intercept, β is the baseline covariate effect, X_i is the baseline mRSS for subject i, t_k is the fixed effect of treatment k, r_l is the fixed effect of region l, v_j is the fixed effect of visit j, $(tv)_{jk}$ is the interaction effect of treatment k by visit j, s_i is the random effect of subject i and $\varepsilon_{ij} \sim \text{Normal}(0, \sigma^2)$ represents the residual variance component with $\text{corr}(\varepsilon_{ij}, \varepsilon_{ij'}) = \rho_{jj'},\ j \neq j'$

As a sensitivity analysis, the MMRM using the above model will also be applied to assessments post-baseline up to termination of study medication or initiation of rescue medication. In addition, the MMRM on all assessments will be repeated including additional effects time on rescue medication and time on study medication. MMRM assumes missing at random; mRSS by time of patient discontinuation from study medication[122] will be summarized and further methods using multiple imputation that allow for missing data not at random will be investigated. Details of such methods will be described in the SAP.

An additional univariate sensitivity analysis will look at change in mRSS from baseline to Week 52 / last visit using analysis of covariance (ANCOVA) with baseline mRSS as a covariate, treatment arm and region as main effects. Last visit is defined as the last visit on treatment which in the case of premature termination of study medication is the termination visit. In the case of rescue[123] medication, the last visit is the visit where the decision is made to start rescue[124] medication with efficacy being assessed prior to escape medication starting. As a robustness analysis, a stratified (by region) non-parametric Wilcoxon test comparing riociguat and placebo will also be performed. In addition, and consistent with the primary analysis, a further ITT sensitivity endpoint analysis of change in mRSS to Week 52 / last visit will include all measurements recorded up to Week 52 or the termination visit, including those while rescue[125] medication was taken or if the patient returned after termination for a later visit up to Week 52.

Rules for the imputation of missing values where the patient discontinues the study (withdrawal of consent or death) or the study medication[126] for the univariate analysis, null / alternative hypothesis statements, and model statements in mathematical notation will be specified in the SAP. Two-sided 95% confidence intervals of treatment (riociguat compared to placebo) differences will be calculated in addition to the statistical significance testing.

[122] Per Amendment 4
[123] Per Amendment 1
[124] Per Amendment 1
[125] Per Amendment 1
[126] Per Amendment 4

8.4.2.2 Secondary and exploratory endpoints[127]

General aspects

The other secondary and exploratory efficacy variables measured on a continuous or semi-continuous scale will analyzed using the MMRM, ANCOVA, and stratified Wilcoxon test, as for the mRSS above.

The time to first mRSS progression and regression and clinical outcome parameters will be analyzed using the log-rank test comparing between treatment groups.

Any binary outcomes, such as the predicted CRISS outcome, will be analyzed using Mantel-Haenzel weights, stratified by region. Also as a sensitivity analysis in addition to the time to event analysis, the occurrence of the events mRSS progression and regression and clinical outcome parameters will be analyzed using this method.

Details on any descriptive subgroup analyses will be specified in the SAP. These will include gender and region.

Hierarchical testing of secondary endpoints[128]

The following secondary endpoints will be tested in a hierarchical fashion at a 2-sided 5% level, only if the primary endpoint of mRSS is shown to be statistically significant at a 2-sided 5% level.

The following order of testing will be applied:

- CRISS
- HAQ-DI (health assessment questionnaire-disability index)
- Patient's global assessment
- Physician's global assessment
- FVC % predicted

For example, if CRISS is statistically significant at a 2-sided 5% level, then the HAQ-DI will be tested, if not the testing procedure will be stopped. This step is repeated further down the list of the 5 secondary endpoints until a non-statistically significant endpoint is reached.

These particular secondary endpoints were chosen to be tested in this hierarchical fashion, because the mRSS and the above additional, secondary variables were considered to have the greatest face validity when designing the CRISS [41].

[127] Per Amendment 4
[128] Per Amendment 4

American College of Rheumatology Combined Response Index for Systemic Sclerosis[129]

The key secondary endpoint will be the CRISS at Week 52.[130]

Application of CRISS algorithm in a randomized clinical trial is a 2-step process. Firstly, evaluate if patients have met the criterion for not-improved. If yes, these patients are assigned a probability score of 0.0. For the remaining patients, calculate the probability based on change in 5 measures: mRSS, FVC %, HAQ-DI, patient's global assessment, and physician's global assessment, where each measure has a probability score between 0 and 1 [41].

In Step 1, a patient is clinically evaluated to determine whether the patient has improved or not. Expert consensus on the definition of a patient who is not improved during a trial is the following:

A patient is considered not improved and is assigned a probability score of improving equal to 0.0, irrespective of improvement on other core items, if he/she develops:

1. New scleroderma renal crisis,
2. Decline in FVC % predicted ≥15% (relative), confirmed by another FVC % within a month, high resolution computed tomography (HRCT) to confirm interstitial lung disease (if previous HRCT did not show interstitial lung disease) and FVC % predicted below 80% predicted (attributable to SSc),
3. New onset of left ventricular failure (defined as ejection fraction ≤45%) or new onset of pulmonary arterial hypertension requiring treatment (attributable to SSc).

If the patient is determined to exhibit improvement in Step 1 (ie, not assigned a 0.0), Step 2 involves computing the predicted probability of improving (a score between 0.0 and 1.0, inclusive) for each patient using the equation:

$$\frac{exp[-5.54-0.81*\Delta_{MRSS}+0.21*\Delta_{FVC\%}-0.40*\Delta_{Pt-glob}-0.44*\Delta_{MD-glob}-3.41*\Delta_{HAQ-DI}]}{1+exp[-5.54-0.81*\Delta_{MRSS}+0.21*\Delta_{FVC\%}-0.40*\Delta_{Pt-glob}-0.44*\Delta_{MD-glob}-3.41*\Delta_{HAQ-DI}]}$$

where Δ_{MRSS} indicates the change in mRSS from baseline to Week 52, Δ_{FVC} denotes the change in FVC % predicted from baseline to Week 52, $\Delta_{Pt\text{-}glob}$ indicates the change in patient global assessment, $\Delta_{MD\text{-}glob}$ denotes the change in physician global assessment, and $\Delta_{HAQ\text{-}DI}$ is the change in HAQ-DI[131]. Note that all changes are absolute changes ($Time_2 - Time_{baseline}$) and that the physician's and patient's global assessment was measured on the Likert scale ranging from 0 to 10, where 0 = excellent, and 10 = extremely poor.

Hence, the CRISS is comprised of 5 variables: mRSS, FVC % predicted, patient's global assessment, physician's global assessment, and HAQ-DI from the SHAQ patient reported outcome. Combined, the 5 variables explained 89.3% of the variability in the data. Individually, when used in a single-variable logistic regression model, mRSS explained 66.3% of the variation, FVC % predicted explained 36.1% of the variation, physician global assessment explained 24.5% of the variation, patient global assessment explained 23.7% of the variation, and HAQ-DI explained 28.5% of the variation. A change in mRSS, FVC % predicted and HAQ-DI are strong indicators of whether a patient is likely to be improved or not. In each scenario, a decrease of mRSS or HAQ-DI from baseline to Week 52[132]and an

[129] Per Amendment 4
[130] Per Amendment 5
[131] Per Amendment 5
[132] Per Amendment 5

increase in FVC % predicted corresponds to very high probabilities of improving. For patient's global and physician's global assessment, the association between probability of improving and change in these 2 core components is less evident. Subjects for which the predicted probability is greater or equal to 0.60 are considered improved, while subjects for which the predicted probability is below 0.60 are considered not improved. The 2 groups (riociguat vs. placebo) can then be compared in a 2x2 table using appropriate significance tests. The predicted probabilities obtained using the CRISS can also be assessed as a continuous variable and the distributions of the probability of improving for patients on drug vs. placebo can be compared using non-parametric tests [41]. In this study, the CRISS outcome will be analyzed using Mantel-Haenzel weights, stratified by region, and additionally, the stratified Wilcoxon test will be used as non-parametric test.

However, Step 2 assumes that there is complete data; ie, all 5 components of the CRISS are fully recorded at Week 52. If the subject discontinues from study drug before Week 52, or has one or more missing components of the CRISS at this visit, then a mixed model repeated measures (MMRM) analysis will be implemented to estimate the value or values for those components at Week 52. These estimates will then we used to calculate the CRISS score at Week 52 as given in Step 2. Subjects for which the predicted probability is greater or equal to 0.60 are considered improved, while subjects for which the predicted probability is below 0.60 are considered not improved. [41] Further details will be provided in the statistical analysis plan.

8.4.3 Safety - amended

The incidence of treatment-emergent AEs (TEAEs) will be tabulated by treatment group. Adverse events are considered to be treatment-emergent if they have started or worsened after first application of study drug up to 2 days after end of treatment with study drug. Further tables will be produced for serious and/or drug-related treatment-emergent AEs, also treatment-emergent AEs leading to discontinuation of study drug and of special safety interest. The incidence of AEs during pre-treatment and during follow-up (ie, AEs occurring more than 2 days after end of treatment with study drug) will be tabulated separately. In addition to the summary tables of AEs, listings of SAEs, AEs leading to discontinuation of study drug and AEs of special safety interest will be provided.

Mortality in the study will be summarized descriptively. Any deaths in the study period will be listed, with day of death relative to start and stop of study drug and cause of death. Mortality is also defined as an exploratory efficacy parameter, and so being a binary outcome the difference in occurrence between treatment groups will be analyzed as described in Section 8.4.2.

The safety evaluation of laboratory data will include:

- Incidence rates of treatment-emergent laboratory values outside of normal range
- Incidence rates of pre-specified laboratory data abnormalities
- Descriptive analysis of continuous laboratory parameters and their changes from baseline by treatment group and visit.

Descriptive analysis of vital signs and their changes from baseline will be performed by treatment group and visit. Descriptive statistics of pulse oximetry and blood pressure/heart

rate measurements for the assessment of orthostatic changes will be performed by treatment group and visit.[133]

For ECGs, the status pre-treatment and post treatment-initiation will be tabulated. The incidence rates of treatment-emergent ECG abnormalities will be tabulated by treatment group. A descriptive analysis of continuous ECG parameters and their changes from baseline by treatment group and visit will also be presented.

8.4.4 Pharmacokinetics

For the investigation of PK, the plasma concentrations of riociguat and its main metabolite M-1 (BAY 60-4552) will be determined at the times given in Section 7.4 using a sparse sampling approach.

The data processing and the statistical analysis will be performed in accordance with the sponsor's current guidelines.

Riociguat and its main metabolite M-1 (BAY 60-4552) peak and trough concentrations will be summarized by visit, separated according to actual dose.

8.5 Planned interim analyses - amended

A formal interim analysis of the main treatment phase is not planned. The main treatment phase will be unblinded for data analysis when the last patient completes the main treatment phase (Visit 12)[134] and the database for the main treatment phase is declared clean, whichever occurs last.

The first safety update will be performed on the long-term extension phase when the last patient completes 24 weeks of treatment (Visit 19)[135]. Safety updates then will be performed at least yearly until the last patient completes the entire study, when a final analysis will be conducted.

8.6 Determination of sample size - amended

There is lack of positive trials in dcSSc. Most trials have used change in mRSS to Month 6 or Month 12 as the primary endpoint, and 4 published articles are referenced for the estimate of variability in this study (Table 8–1).

[133] Per Amendment 6
[134] Per Amendment 5
[135] Per Amendment 5

Table 8–1 References for estimate of variability

<table>
<tr><th>Reference</th><th>Trial treatment</th><th>Active arm SD</th><th>Placebo arm SD</th></tr>
<tr><td>Postlethwaite (2008)[35]</td><td>Collagen 0 – 12 months</td><td>7.6</td><td>7.7</td></tr>
<tr><td>Khanna (2009)[26]</td><td>Relaxin 0 – 24 weeks</td><td>7.0</td><td>7.0</td></tr>
<tr><td>Merkel (2012)[36]</td><td>0 – 6 month meta-analysis</td><td colspan="2">7.2</td></tr>
<tr><td></td><td>0 – 12 month meta-analysis</td><td colspan="2">8.4</td></tr>
<tr><td>Amjadi (2009)[37]</td><td>D-Pen</td><td colspan="2">8.6</td></tr>
<tr><td></td><td>Relaxin</td><td colspan="2">7.0</td></tr>
<tr><td></td><td>Collagen</td><td colspan="2">7.1</td></tr>
</table>

Abbreviations: D-Pen = D-penicillamine; SD = standard deviation.

Taking these sources into account, a reasonable upper bound for the estimate of the standard deviation to be used in the sample size calculation of this study has been taken as 8.0.

One of the components of exploratory efficacy measure[136] mRSS progression is a 5 point or more deterioration from baseline to any assessment in the 12 month study period, this being considered a clinically meaningful change [14]. For the primary sample size calculation based on change in mRSS from baseline to Week 52, a slightly more conservative 4-point difference between riociguat and placebo has been taken, given that the trials referenced above never achieved a 5-point, or even a 4-point, superiority of active over placebo.

The primary efficacy estimand in this study will be change in mRSS from baseline to Week 52. Assuming a standard deviation of 8 points, a power of between 70% and 80% and a two-sided significance level of 5%, with a 1:1 randomization, then to detect a placebo-adjusted difference of 4 points, between 51 and 64 patients in each treatment group, a total of 102 to 128 patients, would be required to be valid for the ITT analysis (see Table 8–2). Allowing for up to two patients randomized and not treated, approximately 130 randomized patients are planned.[137]

Table 8–2 Power calculations: power varying from 70% to 80% in 2% stages[138]

N per group	51	53	56	58	61	64
Total N	102	106	112	116	122	128
Power (%)	70	72	74	76	78	80

N = number of patients

Sources of these sample size and power calculations are nQuery version 7.0, module MTT0-1.[139]

[136] Per Amendment 4
[137] Per Amendment 5
[138] Per Amendment 5
[139] Per Amendment 5

9. Data handling and quality assurance

9.1 Data recording

It is the expectation of the sponsor that all data entered into the eCRF has source documentation available at the site. The site must implement processes to ensure this happens. A source document checklist will be used at the site to identify the source data for all data points collected and the monitor will work with the site to complete this.

Specific data may be entered directly into the eCRF according to instructions provided in the eCRF completion manual.

Data recorded from "only screened patients (screening failures)"

Data of 'only screened patients' will be recorded at least as source data, as far as the reason for the premature discontinuation is identifiable. At minimum, data to be recorded in the eCRF are demographic information (patient number, date of birth/age, sex and ethnicity), inclusion and exclusion criteria, the reason for premature discontinuation and date of last visit. These data will be transferred to the respective database.

For screening failures with an SAE, the following additional data should be collected in the eCRF, in addition to demographic information, primary reason for discontinuation and date of last visit:

- All information about the SAE
- All information related to the SAE such as:
  - Concomitant medication
  - Medical history
  - Other information needed for SAE complementary page

9.2 Monitoring

In accordance with applicable regulations, GCP, and sponsor's/clinical research organization's (CRO's) procedures, monitors will contact the site prior to the start of the study to review with the site staff the protocol, study requirements, and their responsibilities to satisfy regulatory, ethical, and sponsor's requirements. When reviewing data collection procedures, the discussion will also include identification and documentation of source data items.

The sponsor/designee will monitor the site activity to verify that the:

- Data are authentic, accurate and complete
- Safety and rights of patients are being protected
- Study is conducted in accordance with the currently approved protocol (including study treatment being used in accordance with the protocol)
- Any other study agreements, GCP, and all applicable regulatory requirements are met.

The investigator and the head of the medical institution (where applicable) agrees to allow the monitor direct access to all relevant documents.

9.3 Data processing

The data collection tool for this study will be a validated electronic system called Medidata Rave. Patient data necessary for analysis and reporting will be entered/ transmitted into a validated database or data system (eg, TOSCA V4 3_1_1; SAS 9.2). Clinical data management will be performed in accordance with applicable sponsor's standards and data cleaning procedures. This is applicable for data recorded on eCRF as well as for data from other sources (eg, IxRS, laboratory, ECG).

For data coding (eg, AEs, medication), internationally recognized and accepted dictionaries will be used. After its initial release for biometrical analysis, the clinical database is planned to be re-opened for the inclusion of the following additional data: eg, PK data, biomarker[140] data.

9.4 Audit and inspection

To ensure compliance with GCP and regulatory requirements, a member of the sponsor's (or a designated CRO's) quality assurance unit may arrange to conduct an audit to assess the performance of the study at the study site and of the study documents originating there. The investigator/institution will be informed of the audit outcome.

In addition, inspections by regulatory health authority representatives and IEC(s)/IRB(s) are possible. The investigator should notify the sponsor immediately of any such inspection.

The investigator/institution agrees to allow the auditor or inspector direct access to all relevant documents and allocate his/her time and the time of his/her staff to the auditor/inspector to discuss findings and any issues. Audits and inspections may occur at any time during or after completion of the study.

9.5 Archiving

Essential documents shall be archived safely and securely in such a way that ensures that they are readily available upon authorities' request.

Patient (hospital) files will be archived according to local regulations and in accordance with the maximum period of time permitted by the hospital, institution or private practice. Where the archiving procedures do not meet the minimum timelines required by the sponsor, alternative arrangements must be made to ensure the availability of the source documents for the required period.

The investigator/institution notifies the sponsor if the archival arrangements change (eg, relocation or transfer of ownership).

The investigator site file is not to be destroyed without the sponsor's approval.

The contract with the investigator/institution will contain all regulations relevant for the study center.

[140] Per Amendment 5

10. Premature termination of the study

The sponsor has the right to close this study (or, if applicable, individual segments thereof [eg, treatment arms; dose steps; centers]) at any time, which may be due but not limited to the following reasons:

- If risk-benefit ratio becomes unacceptable owing to, for example,
  - Safety findings from this study (eg, SAEs)
  - Results of any interim analysis
  - Evaluation of risk-benefit at any time in the study where a lack of substantial treatment benefit is shown
  - Results of parallel clinical studies
  - Results of parallel animal studies
    (on eg, toxicity, teratogenicity, carcinogenicity or reproduction toxicity).
- If the study conduct (eg, recruitment rate; drop-out rate; data quality; protocol compliance) does not suggest a proper completion of the trial within a reasonable time frame.

The investigator has the right to close his/her center at any time.

For any of the above closures, the following applies:

- Closures should occur only after consultation between involved parties. Final decision on the closure must be in writing.
- All affected institutions (eg, IEC(s)/IRB(s); competent authority(ies); study center; head of study center) must be informed as applicable according to local law.
- All study materials (except documentation that has to remain stored at site) must be returned to the sponsor. The investigator will retain all other documents until notification given by the sponsor for destruction.
- In case of a partial study closure, ongoing patients, including those in post study follow-up, must be taken care of in an ethical manner.

Details for individual patient's discontinuation[141] can be found in Section 5.2.1.

11. Ethical and legal aspects

11.1 Ethical and legal conduct of the study

The procedures set out in this protocol, pertaining to the conduct, evaluation, and documentation of this study, are designed to ensure that the sponsor and investigator abide by GCP guidelines and under the guiding principles detailed in the Declaration of Helsinki. The study will also be carried out in keeping with applicable local law(s) and regulation(s).

Documented approval from appropriate IEC(s)/IRBs will be obtained for all participating centers/countries before start of the study, according to GCP, local laws, regulations and organizations. When necessary, an extension, amendment or renewal of the Ethics

[141] Per Amendment 4

Committee (EC)/IRB approval must be obtained and also forwarded to the sponsor. The responsible unit (eg, EC/IRB, head of the study center/medical institution) must supply to the sponsor, upon request, a list of the EC/IRB members involved in the vote and a statement to confirm that the EC/IRB is organized and operates according to GCP and applicable laws and regulations.

Strict adherence to all specifications laid down in this protocol is required for all aspects of study conduct; the investigator may not modify or alter the procedures described in this protocol.

Modifications to the study protocol will not be implemented by either the sponsor or the investigator without agreement by both parties. However, the investigator or the sponsor may implement a deviation from, or a change of, the protocol to eliminate an immediate hazard(s) to the trial subjects without prior IEC/IRB/sponsor approval/favorable opinion. As soon as possible, the implemented deviation or change, the reasons for it and if appropriate the proposed protocol amendment should be submitted to the IEC/IRB/head of medical institution/sponsor. Any deviations from the protocol must be explained and documented by the investigator.

Details on discontinuation of the entire study or parts thereof can be found in Section 10.

11.2 Patient information and consent

All relevant information on the study will be summarized in an integrated patient information sheet and informed consent form provided by the sponsor or the study center. A sample patient information and informed consent form is provided as a document separate to this protocol.

In addition there will be a Declaration of Objection to the Collection of Study Data after Withdrawal of Consent and the Information Sheet & Informed Consent Form for Collection of Data on Pregnancy and Birth.

Based on this patient information sheet, the investigator or designee will explain all relevant aspects of the study to each patient / legal representative or proxy consenter (if the patient is under legal protection), prior to his/her entry into the study (ie, before any examinations and procedures associated with the selection for the study are performed or any study-specific data is recorded on study-specific forms).

The investigator or designee will also mention that written approval of the IRB/IEC has been obtained.

Each patient / legal representative or proxy consenter will have ample time and opportunity to ask questions and will be informed about the right to withdraw from the study at any time without any disadvantage and without having to provide reasons for this decision.

Only if the patient / legal representative or proxy consenter voluntarily agrees to sign the informed consent form and has done so, may he/she enter the study. Additionally, the investigator and other information provider (if any) will personally sign and date the form. The patient / legal representative or proxy consenter will receive a copy of the signed and dated form.

The signed informed consent statement is to remain in the investigator site file or, if locally required, in the patient's note/file of the medical institution.

In the event that informed consent is obtained on the date that baseline study procedures are performed, the study record or patient's clinical record must clearly show that informed consent was obtained prior to these procedures.

1. If the patient is not capable of providing a signature, a verbal statement of consent can also be given in the presence of an impartial witness (independent of the sponsor and the investigator). This is to be documented by a signature from the informing physician as well as by a signature from the witness.
2. For adults under legal protection, consent shall be given by the legal guardian(s). The consent of an adult under legal protection shall also be requested where such a person is able to express his/her own will. His/her refusal or the withdrawal of his/her consent may not be disregarded.

The informed consent form and any other written information provided to patients / legal representatives or proxy consenters will be revised whenever important new information becomes available that may be relevant to the patient's consent, or there is an amendment to the protocol that necessitates a change to the content of the patient information and / or the written informed consent form. The investigator will inform the patient / legal representative or proxy consenter of changes in a timely manner and will ask the patient to confirm his/her participation in the study by signing the revised informed consent form. Any revised written informed consent form and written information must receive the IEC/IRB`s approval / favorable opinion in advance of use.

11.3 Publication policy

The sponsor is interested in the publication of the results of every study it performs.

All relevant aspects regarding publication will be part of the contract between the sponsor and the investigator/institution.

The sponsor has made the information regarding the study protocol publicly available on the internet at www.clinicaltrials.gov.

11.4 Compensation for health damage of patients / insurance

The sponsor maintains clinical trial insurance coverage for this study in accordance with the laws and regulations of the country in which the study is performed.

11.5 Confidentiality

All records identifying the patient will be kept confidential and, to the extent permitted by the applicable laws and/or regulations, will not be made publicly available.

Patient names will not be supplied to the sponsor. Only the patient number will be recorded in the eCRF, and if the patient name appears on any other document (eg, pathologist report), it must be obliterated before a copy of the document is supplied to the sponsor. Study findings stored on a computer will be stored in accordance with local data protection laws. As part of the informed consent process, the patients will be informed in writing that representatives of the sponsor, IEC/IRB, or regulatory authorities may inspect their medical records to verify the information collected, and that all personal information made available for inspection will be handled in strictest confidence and in accordance with local data protection laws.

If the results of the study are published, the patient's identity will remain confidential.

The investigator will maintain a list to enable patients to be identified.

12. Reference list - amended[142],[143]

1. Van den Hoogen F, Khanna D, Fransen J, Johnson SR, Baron M, Tyndall A, et al. 2013 Classification criteria for systemic sclerosis. Arthritis Rheum. 2013; 65:2737-2747.
2. Chatterjee S. Systemic scleroderma. Cleveland Clinic Center for Continuing Education. http://www.clevelandclinicmeded.com/medicalpubs/diseasemanagement/rheumatology/systemic-sclerosis/. Accessed on 29 Dec 2013.
3. LeRoy EC, Black C, Fleischmajer R, et al: Scleroderma (systemic sclerosis): Classification, subsets and pathogenesis. J Rheumatol. 1988; 15: 202-205.
4. Mayes MD, Lacey JV, Beebe-Dimmer K, Gillespie BW, Cooper B, Laing TK, Schottenfeld D. Prevalence, incidence, survival, and disease characteristics of systemic sclerosis in a large US population. Arthritis Rheum. 2003; 48(8):2246-2255.
5. Varga J, Bashey RI. Regulation of connective tissue synthesis in systemic sclerosis. Int Rev Immunol. 1995;12:187-199.
6. Hesselstrand R, Scheja A, Akesson A. Mortality and causes of death in a Swedish series of systemic sclerosis patients. Ann Rheum Dis. 1998; 57:682-686.
7. Subcommittee for Scleroderma Criteria of the American Rheumatism Association Diagnostic and Therapeutic Criteria Committee. Preliminary criteria for the classification of systemic sclerosis (scleroderma). Arthritis Rheum. 1980;23:581–590.
8. Beyer C, Reich N, Schindler SC, Akhmetshina A, Dees C, Tomcik M, et al. Stimulation of soluble guanylate cyclase reduces experimental dermal fibrosis. Ann Rheum Dis. 2012; 71:1019-1026.
9. Beyer C, Zenzmaier C, Palumbo-Zerr K, Mancuso R, Distler A, Dees C, Zerr P, et al. Stimulation of the soluble guanylate cyclase (sGC) inhibits fibrosis by blocking non-canonical TGFβ signalling. Ann Rheum Dis. 2014 Feb 23 [Epub ahead of print].
10. Chu AJ, Prasad JK. Up-regulation by human recombinant transforming growth factor β-1 of collagen production in cultured dermal fibroblasts is mediated by the inhibition of nitric oxide signaling. J Am Coll Surg. 1999; 188:271-280.
11. Evgenov OV, Zou L, Zhang M, Mino-Kenudson M, Mark EJ, Buys ES, et al. Nitric oxide-independent stimulation of soluble guanylate cyclase attenuates pulmonary fibrosis. BMC Pharmacol. 2011;11(Suppl 1):O9 (abstract).
12. Geschka S, Kretschmer A, Sharkovska Y, Evgenov O, Lawrenz B, Hucke A et al. Soluble guanylate cyclase stimulation prevents fibrotic tissue remodeling and improves survival in salt-sensitive Dahl rats. PLoD One. 2011;6(7):e21853.
13. Sharkovska Y, Kalk P, Lawrenz B, Godes M, Hoffman LS, Wellkisch K et al. Nitric oxide-independent stimulation of soluble guanylate cyclase reduces organ damage in experimental low-renin and high-renin models. J Hypertens. 2010 Aug;28(8):1666-1675.

[142] References 22, 23, 24, 39, and 40 were added per Amendment 1
[143] References 41 and 40 were added per Amendment 4

14. Maurer B, Graf N, Michel BA, Müller-Ladner U, Czirják L, Denton CP, Tyndall A, Metzig C, Lanius V, Khanna D, Distler O. Prediction of worsening of skin fibrosis in patients with diffuse cutaneous systemic sclerosis using the EUSTAR database. Ann Rheum Dis, 2014 - http://ard.bmj.com/content/early/2014/06/30/annrheumdis-2014-205226.full.

15. Khanna. Recombinant human relaxin in the treatment of systemic sclerosis with diffuse cutaneous involvement: a randomized, double-blind, placebo-controlled trial. Arthritis Rheum. 2009, 60(4):1102-11.

16. Pope JE, Bellamy N, Seibold JR, Baron M, Ellman M, Carette S, Smith CD, Chalmers IM, Hong P, O'Hanlon D, Kaminska E, Markland J, Sibley J, Catoggio L, Furst DE. A randomized, controlled trial of methotrexate versus placebo in early diffuse scleroderma. Arthritis Rheum. 2001, 44:1351-8.

17. Amjadi S, Maranian P, Furst DE, Clements PJ, Wong WK, Postlethwaite AE, Khanna PP, Khanna D. Course of the modified Rodnan skin thickness score in systemic sclerosis clinical trials: analysis of three large multicenter, double-blind, randomized controlled trials. Arthritis Rheum. 2009, 60(8):2490-8.

18. Khanna D, Lovell DJ, Giannini E, Clements PJ, Merkel PA, Seibold JR, Matucci-Cerinic M, Denton CP, Mayes MD, Steen VD, Varga J, Furst DE. Development of a provisional core set of response measures for clinical trials of systemic sclerosis. Ann Rheum Dis 2008;67:703–709.

19. Walker KM, Pope J, participating members of the Scleroderma Clinical Trials Consortium (SCTC) and Canadian Scleroderma Research Group (CSRG). Treatment of systemic sclerosis complications: what to use when first-line treatment fails—a consensus of systemic sclerosis experts. Semin Arthritis Rheum. 2012;42:42-55.

20. Kowal-Bielecka, Landewe R, Avouac J, et al. EULAR recommendations for the treatment of systemic sclerosis: a report from the EULAR Scleroderma Trials and Research group (EUSTAR). Ann Rheum Dis 2009;68:620-628.

21. Nagaraja V, Denton CP, Khanna D. Old medications and new targeted therapies in systemic sclerosis. Rheumatology. 2014 July 26 [Epub ahead of print].

22. Regev A, Seeff LB, Merz M, Ormarsdottir S, Aithal GP, Gallivan J, Watkins PB. Causality Assessment for Suspected DILI During Clinical Phases of Drug Development. Drug Saf (2014) 37 (Suppl 1):S47–S56.

23. NICE clinical guideline 182 "Chronic kidney disease: early identification and management of chronic kidney disease in adults in primary and secondary care", issued in July 2014 (guidance.nice.org.uk/cg182).

24. MacGregor MS and Taal MW. Clinical Practice Guidelines - Detection, Monitoring and Care of Patients with CKD. UK renal association, 5th edition, 2011 (www.renal.org/guidelines).

25. Thomas G. Pickering, J.E.H., Lawrence J. Appel, Bonita E. Falkner, John Graves, Martha N. Hill, Daniel W. Jones, Theodore Kurtz, Sheldon G. Sheps, Edward J. Roccella, AHA Scientific Statement: Recommendations for Blood Pressure Measurement in Humans and Experimental Animals. Hypertension. 2005;(45):142-161.

26. Khanna D, Merkel PA. Outcome measures in systemic sclerosis: an update on instruments and current research. Curr Rheumatol Rep. 2007;9:151-157.

27. Khanna D, Clements PJ, Furst DE, Korn JH, Ellman M, Rothfield N, et al. A randomized, double-blind, placebo-controlled trial of recombinant human relaxin in the treatment of systemic sclerosis with diffuse scleroderma. Arthritis Rheum 2009;60(4):1102-1111.

28. Denton C, Merkel PA, Furst DE, Khanna K, Emery P, et al. Recombinant human anti-transforming growth factor β1 antibody therapy in systemic sclerosis: a multicenter, randomized, placebo-controlled phase I/II trial of CAT-192. Arthritis Rheum 2007;56:323-333.

29. Gladue H, Maranian P, Paulus H, Khanna D. Evaluation of test characteristics for outcome measures used in Raynaud's phenomenon clinical trials. Arthritis Care & Res. 2013;65(4):630-636.

30. Merkel PA, Herlyn K, Martin RW, Anderson JJ, Mayes MD, Bell P, et al. Scleroderma Clinical Trials Consortium. Measuring Disease Activity and Functional Status in Patients with Scleroderma and Raynaud's Phenomenon. Arthritis Rheum 2002;46:2410-2420.

31. Ware JE, Sherbourne CD. The MOS 36-item short-form health survey (SF-36). I. Conceptual Framework and Item Selection. Med Care. 1992;30(6):473-483.

32. Steen VD, Medsger TA. The value of the Health Assessment Questionnaire and Special Patient-Generated Scales to Demonstrate Change in Systemic Sclerosis Patients Over Time. Arthritis Rheum 1997;40:1984-1991.

33. Khanna D, Hays RD, Maranian P, Seibold JR, Impens A, Mayes MD, et al. Reliability and validity of the University of California, Los Angeles Scleroderma Clinical Trial Consortium Gastrointestinal Tract Instrument. Arthritis Rheum 2009;61(9):1257-1263.

34. Hinchcliff M, Beaumont JL, Thavarajah K, Varga J, Chung A, Podulsky S, et al. Validity of two new patient-reported outcome measures in systemic sclerosis: Patient-Reported Outcomes Measurement Information System 29-Item Health Profile and Functional Assessment of Chronic Illness Therapy–Dyspnea Short Form. Arthritis Care & Res 2011;63(11):1620-1628.

35. Postlethwaite AE, Wong WK, Clements P, Chatterjee S, Fessler BJ, Kang AH, et al. A multicenter, randomized, double-blind, placebo-controlled trial of oral type I collagen treatment in patients with diffuse cutaneous systemic sclerosis: I. Oral type I collagen does not improve skin in all patients, but may improve skin in late-phase disease. Arthritis Rheum 2008;58 (6):1810-1822.

36. Merkel PA, Silliman NP, Clements PJ, Denton CP, Furst DE, Mayes MD, et al. Patterns and predictors of change in outcome measures in clinical trials in scleroderma an individual patient meta-analysis of 629 subjects with diffuse scleroderma. Arthritis Rheum 2012;64(10): 3420-3429.

37. Amjadi S, Maranian P, Furst DE, Clements PJ, Wong WK, Postlethwaite AE, et al. for the investigators of the D-Penicillamine, human recombinant relaxin, and oral bovine type I collagen clinical trials. Course of modified Rodnan skin score in systemic

sclerosis clinical trials: analysis of 3 large multicenter, randomized clinical trials. Arthritis Rheum 2009;60(8):2490-2498.

38. Pugh RN, Murray-Lyon IM, Dawson JL, Pietroni MC, Williams R. Transection of the oesophagus for bleeding oesophageal varices. Br J Surg.1973;60:646-649.
39. Steen VD and Medsger TA. Long-Term Outcomes of Scleroderma Renal Crisis. Ann Intern Med. 2000;133:600-603.
40. Shanmugam VK and Steen VD. Renal disease in scleroderma: an update on evaluation, risk stratification, pathogenesis and management. Curr Opin Rheumatol. 2012 November ; 24(6): 669–676.
41. Khanna D, Berrocal VJ, Giannini EH, Seibold JR, Merkel PA, Mayes MD, et al. The American College of Rheumatology Provisional Composite Response Index for Clinical Trials in Early Diffuse Cutaneous Systemic Sclerosis. Arthritis Rheumatol. 2016; 68(2):299-311.
42. Khanna D, Lovell DJ, Giannini E, Clements PJ, Merkel PA, Seibold JR, et al. Development of a provisional core set of response measures for clinical trials of systemic sclerosis. Ann.Rheum Dis. 2008;67(5):703-709.

13. Protocol amendments

13.1 Amendment 1

Amendment 1 was approved on 4 FEB 2015.

13.1.1 Overview of changes to the study

Due to regulatory feedback, additional safety laboratory monitoring is being included for patients entering the study with elevated liver transaminases or bilirubin, or with eGFR between 15-29 mL/min/1.73 m^2. These patients were not included in the riociguat pivotal trials in pulmonary hypertension. While there is no evidence of liver or renal toxicity with riociguat, there is an increased risk for adverse events related to the mechanism of action of the drug, such as hypotension, due to a higher plasma concentration caused by impaired riociguat metabolism/elimination by liver and kidneys.

The list of immunosuppressant therapies requiring washout before inclusion in the study was also expanded to account for standard of care treatment among the participating countries.

Safety laboratory tests were added at Visit 10, along with other safety assessments such as ECG and lung function tests to be performed. Measurement of glutamate dehydrogenase (GLDH) will be deleted from the list of standard safety parameters because the review of data from previous studies and the ongoing long-term studies did not show any clinically significant changes of this parameter in patients treated with riociguat.

Typos and inconsistencies were also corrected, as well as re-wording to make the protocol clearer.

13.1.2 Changes to the protocol text

Section – Title page

Old text:

Sponsor's medical expert: PPD
~~Bayer Healthcare UK~~
~~Strawberry Hill~~
~~Newbury, RG14 1JA~~
~~United Kingdom~~
~~Phone:~~ PPD

New text:

Sponsor's medical expert: PPD
<u>Bayer Healthcare Company Ltd</u>
<u>Bayer Center,</u>
<u>No.27, Dong San Huan North Road, Chaoyang District,</u>
<u>Beijing, 100020 , China</u>
<u>Tel:</u> PPD
<u>Fax:</u>PPD

1.1.2.2 Preclinical Data

Old text:

In summary, investigations of the anti-fibrotic efficacy of sGC stimulators riociguat and BAY 41-2272 showed that these agents act dose-dependently:

- reduced collagen production in human dermal fibroblasts
- reduced fibroblast-to-myofibroblast differentiation of human dermal fibroblasts
- prevented TGF-ß-induced skin fibrosis *in vivo*
- prevented skin fibrosis in bleomycin model
- reduced established skin fibrosis in genetic TSK-1 model
- promoted wound healing in TSK-1 mice.

New text:

In summary, investigations of the anti-fibrotic efficacy of sGC stimulators riociguat and BAY 41-2272, another sGC compound, showed that these agents act dose-dependently:

- reduced collagen production in human dermal fibroblasts
- reduced fibroblast-to-myofibroblast differentiation of human dermal fibroblasts
- prevented TGF-ß-induced skin fibrosis *in vivo*
- prevented skin fibrosis in bleomycin model
- reduced established skin fibrosis in genetic TSK-1 model
- promoted wound healing in TSK-1 mice.

2 Study objectives

Old text:

The secondary objectives of this study are to assess the efficacy of treatment with riociguat administered TID as compared with placebo in terms of:

- Efficacy:
    - Need for ~~Escape~~ Therapy (see definition in Section 6.9)

New text:

The secondary objectives of this study are to assess the efficacy of treatment with riociguat administered TID as compared with placebo in terms of:

- Efficacy:
    - Need for Rescue Therapy (see definition in Section 6.9)

3. Investigators and other study personnel

Old text:

Sponsor's medical expert:

Name: PPD
Address: ~~Bayer Healthcare UK~~
~~Strawberry Hill~~
~~Newbury, RG14 1JA~~
~~United Kingdom~~
~~Phone:~~ PPD

New text:

Sponsor's medical expert:

Name: PPD
Address: Bayer Healthcare Company Ltd
Bayer Center,
No.27, Dong San Huan North Road, Chaoyang District,
Beijing, 100020, China
Tel: PPD
Fax: PPD

4.1.2 Main treatment phase (Week 0 - Week 52)

Old text:

Maintenance period (Visits 7 to 12)

The overall duration of the main treatment phase is 52 weeks, including the dose titration period. At the end of the dose titration period (Week 10) the patient's maintenance dose will be determined using the treatment algorithm above. No further increase in dose will be allowed. The established dose will then be taken as the "optimal dose" to be administered for the remaining duration of the main treatment phase (up to Week 52).

Dose reductions for safety reasons (eg, in case of any treatment-emergent adverse event [AE]) are allowed, but a subsequent dose increase during the maintenance period is not permitted.

From Week 26 (Visit 10), patients will have the opportunity to add "~~escape~~ therapy", consisting of immunosuppressant drugs, to their randomized study medication if they meet certain criteria for worsening skin or pulmonary disease. Please refer to Section 6.9 for further details.

New text:

Maintenance period (Visits 7 to 12)

The overall duration of the main treatment phase is 52 weeks, including the dose titration period. At the end of the dose titration period (Week 10) the patient's maintenance dose will be determined using the treatment algorithm above. No further increase in dose will be

allowed. The established dose will then be taken as the “optimal dose” to be administered for the remaining duration of the main treatment phase (up to Week 52).

Dose reductions for safety reasons (eg, in case of any treatment-emergent adverse event [AE]) are allowed, but a subsequent dose increase during the maintenance period is not permitted.

From Week 26 (Visit 10), patients will have the opportunity to add “rescue therapy”, consisting of immunosuppressant drugs, to their randomized study medication if they meet certain criteria for worsening skin or pulmonary disease. Please refer to Section 6.9 for further details.

4.1.4 Termination visit and safety follow-up

Old text:

....

If the patient discontinues study ~~participation~~ prematurely during the main treatment phase (Week 0 – Week 52) he/she will be invited to come for limited assessments (mRSS, FVC, DL_{CO} and patient-reported outcomes [PROs]) at Week 12, Week 26, Week 39, and Week 52, depending on the timing of withdrawal. The collection of these assessments is important for endpoint analysis which considers all assessments post baseline up to Week 52.

New text:

...

If the patient discontinues study drug prematurely during the main treatment phase (Week 0 – Week 52) he/she will be invited to come for limited assessments (mRSS, FVC, DL_{CO} and patient-reported outcomes [PROs]) at Week 12, Week 26, Week 39, and Week 52, depending on the timing of withdrawal. The collection of these assessments is important for endpoint analysis which considers all assessments post baseline up to Week 52.

5.1.2 Exclusion criteria

Old text:

2. Hepatic-related criteria
   - Hepatic insufficiency classified as Child-Pugh C (see Appendix 14.1 for classification table)
3. Renal-related criteria
   - Estimated glomerular filtration rate (eGFR) < 15 mL/min/1.73m^2 (Modification of Diet in Renal Disease [MDRD] formula) or on dialysis at the screening visit
     - Because the MDRD formula is thought to cause significant bias for Japanese patients, the equation for Japanese patients is:
       194 x serum creatinine (mg/dL) -1.094 x Age -0.287 x 0.739 (if female).
7. Prior and concomitant therapy
   - Treatment with methotrexate, cyclophosphamide, hydroxychloroquine, cyclosporine A, azathioprine, mycophenolate mofetil, rapamycin, colchicine,

D-penicillamine, or intravenous immunoglobulin within 4 weeks before the screening visit

New text:

2. Hepatic-related criteria
    - Hepatic insufficiency classified as Child-Pugh C (see Appendix 14.1 for classification table)
        - Patients with isolated AST or ALT >3xULN or bilirubin >2xULN can be included in the trial under the condition of additional monitoring during the trial (see section 7.6.2)
3. Renal-related criteria
    - Estimated glomerular filtration rate (eGFR) < 15 mL/min/1.73m^2 (Modification of Diet in Renal Disease [MDRD] formula) or on dialysis at the screening visit. Patients entering the trial with eGFR 15-29 mL/min/1.73m^2 will be undergo additional monitoring of renal function (see section 7.6.2)
        - Because the MDRD formula is thought to cause significant bias for Japanese patients, the equation for Japanese patients is:
          194 x serum creatinine (mg/dL) -1.094 x Age -0.287 x 0.739 (if female).
7. Prior and concomitant therapy
    - Treatment with methotrexate, cyclophosphamide, hydroxychloroquine, cyclosporine A, azathioprine, mycophenolate mofetil, rapamycin, colchicine, D-penicillamine, tacrolimus, mizoribine or intravenous immunoglobulin within 4 weeks before the screening visit

5.2.1 Withdrawal

Old text:

Patients *must* be withdrawn from the study for the following reasons:

- If treatment is interrupted for > 3 consecutive days during the dose titration period of the main treatment phase

Patients *may* be withdrawn from the study for the following reasons:

- At the specific request of the sponsor and in liaison with the investigator (eg, safety concerns)
- A relative decline in FVC % predicted by ≥ 10% (eg, a change of FVC from 60% to 54%) or a relative decline in FVC % predicted between 5 to < 10% with associated relative decline in DL_{CO} % predicted by ≥ 15%, provided that the decline in FVC results in FVC <75% of predicted (confirmed by repeat pulmonary function testing within 1 month). Please refer to Section 6.9 for the option of adding ~~escape~~ therapy.
- An absolute increase in mRSS by > 5 units and ≥ 25% (Please refer to Section 6.9 for the option of adding ~~escape~~ therapy), life-threatening or organ-threatening event attributable to SSc (such as renal crisis, digital gangrene, development of new PAH on right heart catheterization) or not attributable to SSc.

A patient who, for any reason (eg, failure to satisfy the selection criteria), terminates the study before the time point used for the definition of "dropout" (see below) is regarded a "screening failure". Restarting the defined set of screening procedures to enable the "screening failure" patient's participation at a later time point is not allowed.

One re-assessment of laboratory parameters is allowed during the screening phase to re-assess the eligibility of patients.

A patient who discontinues study participation prematurely for any reason is defined as a "dropout" if the patient has already been randomized.

Any randomized patient removed from the trial should undergo the assessments at the termination visit. The patient will remain under medical supervision until discharge or transfer is medically acceptable and will complete the 30-day safety follow-up period.

If the patient discontinues study ~~participation~~ prematurely during the main treatment phase (Week 0 – Week 52) he/she will be invited to come for limited assessments (mRSS, FVC, DL_{CO} and patient-reported outcomes [PROs]) at Week 12, Week 26, Week 39, and Week 52, depending on the timing of withdrawal. This will be in addition to the termination visit assessments (see Section 7.1.2.8), provided that the patient does not object to collection of study data after withdrawal of consent. If the discontinuation occurs before Visit 8 (Week 14), the skin biopsy and blood samples for biomarkers are to be collected.

New text:

Patients *must* be withdrawn from the study for the following reasons:

- If treatment is interrupted for > 3 consecutive days <u>(9 doses)</u> during the dose titration period of the main treatment phase

Patients *may* be withdrawn from the study for the following reasons:

- At the specific request of the sponsor and in liaison with the investigator (eg, safety concerns)
- A relative decline in FVC % predicted by ≥ 10% (eg, a change of FVC from 60% to 54%) or a relative decline in FVC % predicted between 5 to < 10% with associated relative decline in DL_{CO} % predicted by ≥ 15%, provided that the decline in FVC results in FVC <75% of predicted (confirmed by repeat pulmonary function testing within 1 month). Please refer to Section 6.9 for the option of adding <u>rescue</u> therapy.
- An absolute increase in mRSS by > 5 units and ≥ 25% (Please refer to Section 6.9 for the option of adding <u>rescue</u> therapy), life-threatening or organ-threatening event attributable to SSc (such as renal crisis, digital gangrene, development of new PAH on right heart catheterization) or not attributable to SSc.

A patient who, for any reason (eg, failure to satisfy the selection criteria), terminates the study before the time point used for the definition of "dropout" (see below) is regarded a "screening failure". Restarting the defined set of screening procedures to enable the "screening failure" patient's participation at a later time point is not allowed.

One re-assessment of laboratory parameters is allowed during the screening phase to re-assess the eligibility of patients.

A patient who discontinues study participation prematurely for any reason is defined as a "dropout" if the patient has already been randomized.

Any randomized patient removed from the trial should undergo the assessments at the termination visit. The patient will remain under medical supervision until discharge or transfer is medically acceptable and will complete the 30-day safety follow-up period.

If the patient discontinues study drug prematurely during the main treatment phase (Week 0 – Week 52) he/she will be invited to come for limited assessments (mRSS, FVC, DL_{CO} and patient-reported outcomes [PROs]) at Week 12, Week 26, Week 39, and Week 52, depending on the timing of withdrawal. This will be in addition to the termination visit assessments (see Section 7.1.2.8), provided that the patient does not object to collection of study data after withdrawal of consent. If the discontinuation occurs before Visit 8 (Week 14), the skin biopsy and blood samples for biomarkers are to be collected.

6.9 Prior and concomitant medications

Old text:

Table 6-2 Prohibited prior and concomitant therapy

Therapy	Timeframe
Chlorambucil, bone marrow transplantation, or total lymphoid irradiation	Any previous therapy[a]
Rituximab or other anti-CD20 antibodies	Within 6 months prior to the screening visit[a]
Infliximab, certolizumab, golimumab, adalimumab, abatacept, leflunomide or tocilizumab	Within 8 weeks prior to the screening visit[a]
Methotrexate, cyclophosphamide, hydroxychloroquine, cyclosporine A, azathioprine, mycophenolate mofetil, rapamycin, colchicine, D penicillamine, or intravenous immunoglobulin	Within 4 weeks prior to the screening visit[a]
Etanercept	Within 2 weeks prior to the screening visit[a]
Anakinra	Within 1 week prior to the screening visit[a]
Nitrates or NO donors (such as amyl nitrate) in any form, including topical; phosphodiesterase (PDE) 5 (PDE5) inhibitors (such as sildenafil, tadalafil, vardenafil); and nonspecific PDE5 inhibitors (theophylline, dipyridamole)	Concomitant therapy with study drug
Prostacyclin analogs (oral beraprost for digital ulcers / Raynaud's disease and short-term / intermittent therapy of up to 21 days with intravenous prostacyclin analogs for digital / vascular lesions is allowed)	Concomitant therapy with study drug

a Concomitant use of these treatments with study medication is also prohibited, except for certain agents from Week 26 onwards; please see "~~Escape~~ Therapy" below.

Other medications and considerations (detailed information can be found in the current IB)

- Pre- and concomitant treatment with the proton pump inhibitor omeprazole (40 mg once daily) reduced riociguat mean area under the plasma concentration versus time curve (AUC) by 26% and mean maximum plasma concentration (C_{max}) by 35%. This is not considered clinically relevant.
- Co-administration of the antacid aluminum hydroxide / magnesium hydroxide reduced riociguat mean AUC by 34% and mean C_{max} by 56%. Antacids should be taken at least 1 hour after riociguat.
- Plasma concentrations in smokers are reduced by 50–60% compared with nonsmokers. A dose reduction should be considered in patients who stop smoking whilst participating in the study.

~~Escape~~ therapy

From Week 26 (Visit 10), patients with the following will have the opportunity to add rescue therapy to their randomized study medication. "~~Escape~~ therapy" is defined as treatment with an immunosuppressant drug, under the following situations:

- Worsening of skin disease (defined as > 5 units and ≥ 25% increase in mRSS), or
- Relative decline in FVC % predicted by ≥ 10%, or relative decline in FVC % predicted between ≥5% and < 10% with associated relative decline in DL_{CO} % predicted by ≥ 15%, provided that the decline in FVC results in FVC <75% of predicted (confirmed by repeat pulmonary function testing within 1 month).

The decision to initiate rescue therapy is based on investigator discretion in eligible patients. Rescue therapy may include any of the following 4 agents: methotrexate, mycophenolate mofetil, cyclophosphamide, or azathioprine. These treatments are not provided by the Sponsor.

New text:

Table 6-2 Prohibited prior and concomitant therapy - amended

Therapy	Timeframe
Chlorambucil, bone marrow transplantation, or total lymphoid irradiation	Any previous therapy[a]
Rituximab or other anti-CD20 antibodies	Within 6 months prior to the screening visit[a]
Infliximab, certolizumab, golimumab, adalimumab, abatacept, leflunomide or tocilizumab	Within 8 weeks prior to the screening visit[a]
Methotrexate, cyclophosphamide, hydroxychloroquine, cyclosporine A, azathioprine, mycophenolate mofetil, rapamycin, colchicine, D penicillamine, <u>tacrolimus, mizoribine</u> or intravenous immunoglobulin	Within 4 weeks prior to the screening visit[a]
Etanercept	Within 2 weeks prior to the screening visit[a]
Anakinra	Within 1 week prior to the screening visit[a]
Nitrates or NO donors (such as amyl nitrate) in any form, including topical; phosphodiesterase (PDE) 5 (PDE5) inhibitors (such as sildenafil, tadalafil, vardenafil); and nonspecific PDE5 inhibitors (theophylline, dipyridamole)	Concomitant therapy with study drug
Prostacyclin analogs (oral beraprost for digital ulcers / Raynaud's disease and short-term / intermittent therapy of up to 21 days with intravenous prostacyclin analogs for digital / vascular lesions is allowed)	Concomitant therapy with study drug

a Concomitant use of these treatments with study medication is also prohibited, except for certain agents from Week 26 onwards; please see "<u>Rescue</u> Therapy" below.

Other medications and considerations (detailed information can be found in the current IB)

- Pre- and concomitant treatment with the proton pump inhibitor omeprazole (40 mg once daily) reduced riociguat mean area under the plasma concentration versus time curve (AUC) by 26% and mean maximum plasma concentration (C_{max}) by 35%. This is not considered clinically relevant.
- Co-administration of the antacid aluminum hydroxide / magnesium hydroxide reduced riociguat mean AUC by 34% and mean C_{max} by 56%. Antacids should be taken at least 1 hour after riociguat.
- Plasma concentrations in smokers are reduced by 50−60% compared with nonsmokers. For this reason, it is highly recommended to strongly advise patients to stop smoking while taking this medication. A dose reduction should be considered in patients who stop smoking whilst participating in the study.

Rescue therapy

From Week 26 (Visit 10), patients with the following will have the opportunity to add rescue therapy to their randomized study medication. “Rescue therapy” is defined as treatment with an immunosuppressant drug, under the following situations:

- Worsening of skin disease (defined as > 5 units and ≥ 25% increase in mRSS), or
- Relative decline in FVC % predicted by ≥ 10%, or relative decline in FVC % predicted between ≥5% and < 10% with associated relative decline in DL_{CO} % predicted by ≥ 15%, provided that the decline in FVC results in FVC <75% of predicted (confirmed by repeat pulmonary function testing within 1 month).

The decision to initiate rescue therapy is based on investigator discretion in eligible patients. Rescue therapy may include any of the following 4 agents: methotrexate, mycophenolate mofetil, cyclophosphamide, or azathioprine. These treatments are not provided by the Sponsor.

7.1.1 Tabulated overview

Old text:

Table 7-1 Schedule of evaluations

	Scr	Main Treatment Phase (Double-blind)												Long-term Extension Phase							
	Scr Phase	Dose-Titration Period						Maintenance Period						Dose-Titration Period					Open-label Extension Period	Safety Follow-up	
**Visit**	**V0**	**V1**	**V2**	**V3**	**V4**	**V5**	**V6**	**V7**	**V8**	**V9**	**V10**	**V11**	**V12[a]**	**V13**	**V14**	**V15**	**V16**	**V17**	**V18 – Vn (q 12 wk)**	**TV[b]**	**SFU**
**Week**	-2	0	2	4	6	8	10	12	14	20	26	39	52	54	56	58	60	62	64 – n		
**Day**	**-14 to -1**	**0**	**14 ± 2**	**28 ± 2**	**42 ± 2**	**56 ± 2**	**70 ± 2**	**84 ± 2**	**98 ± 2**	**140 ± 2**	**182 ± 2**	**273 ± 14**	**364 ± 14**	**378 ± 2**	**392 ± 2**	**406 ± 2**	**420 ± 2**	**434 ± 2**	**448 – (n + 84) ± 14**		**30 + 5**
Written informed consent	X																				
Inclusion / exclusion criteria	X	X																			
Demographic data	X																				
Medical and surgical history	X	X																			
Other history (alcohol)	X																				
Smoking history	X	X									X		X						X	X	
Height and weight[c]	X	X						X			X		X						X	X	X
Physical examination	X	X						X			X		X						X	X	X
mRSS	X							X			X	X	X						X	X	
Assessment of Raynaud's attacks	X[d]	X[e]						X[f]	X[e]												
Digital ulcer net burden assessment	X	X	X	X	X	X	X	X	X	X	X	X	X	X	X	X	X	X	X	X	
Tender & swollen joint count	X												X							X[g]	

Table 7-1 Schedule of evaluations

	Scr	Main Treatment Phase (Double-blind)												Long-term Extension Phase							
	Scr Phase	Dose-Titration Period						Maintenance Period						Dose-Titration Period					Open-label Extension Period	Safety Follow-up	
**Visit**	**V0**	**V1**	**V2**	**V3**	**V4**	**V5**	**V6**	**V7**	**V8**	**V9**	**V10**	**V11**	**V12[a]**	**V13**	**V14**	**V15**	**V16**	**V17**	**V18 – Vn (q 12 wk)**	**TV[b]**	**SFU**
**Week**	-2	0	2	4	6	8	10	12	14	20	26	39	52	54	56	58	60	62	64 – n		
**Day**	**-14 to -1**	**0**	**14 ± 2**	**28 ± 2**	**42 ± 2**	**56 ± 2**	**70 ± 2**	**84 ± 2**	**98 ± 2**	**140 ± 2**	**182 ± 2**	**273 ± 14**	**364 ± 14**	**378 ± 2**	**392 ± 2**	**406 ± 2**	**420 ± 2**	**434 ± 2**	**448 – (n + 84) ± 14**		**30 + 5**
Vital signs (BP, HR)[h]	X	X	X	X	X	X	X	X	X	X	X	X	X	X	X	X	X	X	X	X	X
Vital signs post-dose (BP, HR)[i,j]		X[i]										X[j]	X[i]								
Blood sample for safety[k]	X	X					X					X	X					X	X	X	X
Blood sample for PK		X[l]	X[m]	X[m]	X[m]	X[m]	X[m]					X[n]	X[l]	X[m]	X[m]	X[m]	X[m]	X[m]			
Blood sample for biomarkers		X							X											X[r]	
Blood sample for Autoantibody screen	X																				
Pregnancy test (if applicable)[o]	X	X		X		X		X		X			X		X		X		X	X	X
12-lead ECG[p]	X	X						X			X	X	X						X	X	X
Pulmonary function tests[q]	X							X			X	X	X						X	X	
Skin biopsy		X							X											X[r]	
Questionnaires[s]		X							X		X	X	X						X	X	
Patient's and physician's global assessment		X									X		X							X[g]	
Patient interference with skin		X									X		X							X[g]	
Tendon Friction Rubs[t]		X							X												

Table 7-1 Schedule of evaluations

	Scr	Main Treatment Phase (Double-blind)												Long-term Extension Phase							
	Scr Phase	Dose-Titration Period						Maintenance Period						Dose-Titration Period					Open-label Extension Period	Safety Follow-up	
**Visit**	**V0**	**V1**	**V2**	**V3**	**V4**	**V5**	**V6**	**V7**	**V8**	**V9**	**V10**	**V11**	**V12[a]**	**V13**	**V14**	**V15**	**V16**	**V17**	**V18 – Vn (q 12 wk)**	**TV[b]**	**SFU**
**Week**	-2	0	2	4	6	8	10	12	14	20	26	39	52	54	56	58	60	62	64 – n		
**Day**	**-14 to -1**	**0**	**14 ± 2**	**28 ± 2**	**42 ± 2**	**56 ± 2**	**70 ± 2**	**84 ± 2**	**98 ± 2**	**140 ± 2**	**182 ± 2**	**273 ± 14**	**364 ± 14**	**378 ± 2**	**392 ± 2**	**406 ± 2**	**420 ± 2**	**434 ± 2**	**448 – (n + 84) ± 14**		**30 + 5**
Randomization (IxRS)		X																			
Dispensation of study drug		X	X	X	X	X	X	X	X	X	X	X	X	X	X	X	X	X	X		
Drug accountability			X	X	X	X	X	X	X	X	X	X	X	X	X	X	X	X	X	X	
Adverse events recording	X	X	X	X	X	X	X	X	X	X	X	X	X	X	X	X	X	X	X	X	X
Prior and concomitant therapy	X	X	X	X	X	X	X	X	X	X	X	X	X	X	X	X	X	X	X	X	X
Survival status																					X

Abbreviations: BP = blood pressure; ECG = electrocardiogram; TV = Termination Visit; SFU = 30-Day Safety Follow-up (30 [+5] days after the last dose of study drug); HR = heart rate; IxRS = telephone-based or web-based response system; mRSS = modified Rodnan skin score; PK = pharmacokinetics; q 12 wk = every 12 weeks; Scr = Screening.

Note: Day 0 (Visit 1) is the baseline visit.

a Visit 12 (Week 52) is the last visit in the Maintenance Period of the Main Treatment phase and the first (baseline) visit in the Long-term Extension phase.

b To be performed as soon as possible after withdrawal of study medication.

c Height to be measured only at the screening visit

d Patient to be provided with 7-day diary, to complete daily for 7 consecutive days leading up to Visit 1

e Patient and physician assessment of Raynaud's disease (see Section 7.6.12)

f Patient to be provided with 7-day diary, to complete daily for 7 consecutive days leading up to Visit 8.

g Must not be performed if TV is after V12.

h Blood pressure and heart rate will be measured at all visits with the patient in a sitting position, after the patient has been at rest for at least 5 minutes. The same arm is always used for these measurements (see Section 7.6.5).

i Vital signs to be measured within 20 minutes prior to each post-dose PK sampling (see Table 7–2)

Table 7-1 Schedule of evaluations

	Scr	Main Treatment Phase (Double-blind)											Long-term Extension Phase							
	Scr Phase	Dose-Titration Period						Maintenance Period						Dose-Titration Period					Open-label Extension Period	Safety Follow-up	
**Visit**	**V0**	**V1**	**V2**	**V3**	**V4**	**V5**	**V6**	**V7**	**V8**	**V9**	**V10**	**V11**	**V12[a]**	**V13**	**V14**	**V15**	**V16**	**V17**	**V18 – Vn (q 12 wk)**	**TV[b]**	**SFU**
**Week**	-2	0	2	4	6	8	10	12	14	20	26	39	52	54	56	58	60	62	64 – n		
**Day**	**-14 to -1**	**0**	**14 ± 2**	**28 ± 2**	**42 ± 2**	**56 ± 2**	**70 ± 2**	**84 ± 2**	**98 ± 2**	**140 ± 2**	**182 ± 2**	**273 ± 14**	**364 ± 14**	**378 ± 2**	**392 ± 2**	**406 ± 2**	**420 ± 2**	**434 ± 2**	**448 – (n + 84) ± 14**		**30 + 5**

j Vital signs to be measured within 20 minutes prior to PK sampling (see Table 7–2)
k Coagulation tests to be performed at a local laboratory
l Samples for PK to be taken 1 – 2 hours and 3 – 4 hours after the morning dose of study medication (see Table 7–2)
m Sample for PK to be taken up to 1 hour before the morning dose of study medication (trough) (see Table 7–3)
n Samples for PK to be taken up to 1 hour before the morning dose of study medication (trough) and 2 – 3 hours after the morning dose of study medication (peak) (see Table 7–4)
o For women of childbearing potential only. Serum pregnancy test is required only at screening; pregnancy testing (urine) will be done every 4 weeks and should continue until 30 days after the last dose of study drug. When pregnancy testing coincides with a visit, the test should be performed at the site, but otherwise should be performed at home.
p Standard ECG (12-lead ECG) will be recorded with the patient in the supine position, after the patient has been at rest for at least 5 minutes (see Section 7.6.7).
q Pulmonary function tests include forced vital capacity (FVC) and diffusion capacity of the lung for carbon monoxide (DL_{CO}).
r Must be done at a TV if this visit occurs before Visit 8.
s Questionnaires include Scleroderma Health Assessment Questionnaire (SHAQ), Short Form 36 (SF-36), Patient-Reported Outcomes Measurement Information System (PROMIS)-29, and University of California, Los Angeles, Scleroderma Clinical Trial Consortium Gastrointestinal Scale (UCLA SCTC GIT) 2.0.
t Assessment of the presence or absence of tendon friction rubs

New text:

Table 7-1 Schedule of evaluations - amended

	Scr	Main Treatment Phase (Double-blind)											Long-term Extension Phase							
	Scr Phase	Dose-Titration Period						Maintenance Period						Dose-Titration Period					Open-label Extension Period	Safety Follow-up	
**Visit**	**V0**	**V1**	**V2**	**V3**	**V4**	**V5**	**V6**	**V7**	**V8**	**V9**	**V10**	**V11**	**V12[a]**	**V13**	**V14**	**V15**	**V16**	**V17**	**V18 – Vn (q 12 wk)**	**TV[b]**	**SFU**
**Week**	-2	0	2	4	6	8	10	12	14	20	26	39	52	54	56	58	60	62	64 – n		
**Day**	**-14 to -1**	**0**	**14 ± 2**	**28 ± 2**	**42 ± 2**	**56 ± 2**	**70 ± 2**	**84 ± 2**	**98 ± 2**	**140 ± 2**	**182 ± 2**	**273 ± 14**	**364 ± 14**	**378 ± 2**	**392 ± 2**	**406 ± 2**	**420 ± 2**	**434 ± 2**	**448 – (n + 84) ± 14**		**30 + 5**
Written informed consent	X																				
Inclusion / exclusion criteria	X	X																			
Demographic data	X																				
Medical and surgical history	X	X																			
Other history (alcohol)	X																				
Smoking history	X	X									X		X						X	X	
Height and weight[c]	X	X						X			X		X						X	X	X
Physical examination	X	X						X			X		X						X	X	X
mRSS	X							X			X	X	X						X	X	
Assessment of Raynaud's attacks	X[d]	X[e]						X[f]	X[e]												
Digital ulcer net burden assessment	X	X	X	X	X	X	X	X	X	X	X	X	X	X	X	X	X	X	X	X	
Tender & swollen joint count	X												X							X[g]	
Vital signs (BP, HR)[h]	X	X	X	X	X	X	X	X	X	X	X	X	X	X	X	X	X	X	X	X	X

Table 7-1 Schedule of evaluations - amended

	Scr	Main Treatment Phase (Double-blind)												Long-term Extension Phase							
	Scr Phase	Dose-Titration Period						Maintenance Period						Dose-Titration Period					Open-label Extension Period	Safety Follow-up	
**Visit**	**V0**	**V1**	**V2**	**V3**	**V4**	**V5**	**V6**	**V7**	**V8**	**V9**	**V10**	**V11**	**V12[a]**	**V13**	**V14**	**V15**	**V16**	**V17**	**V18 – Vn (q 12 wk)**	**TV[b]**	**SFU**
**Week**	-2	0	2	4	6	8	10	12	14	20	26	39	52	54	56	58	60	62	64 – n		
**Day**	**-14 to -1**	**0**	**14 ± 2**	**28 ± 2**	**42 ± 2**	**56 ± 2**	**70 ± 2**	**84 ± 2**	**98 ± 2**	**140 ± 2**	**182 ± 2**	**273 ± 14**	**364 ± 14**	**378 ± 2**	**392 ± 2**	**406 ± 2**	**420 ± 2**	**434 ± 2**	**448 – (n + 84) ± 14**		**30 + 5**
Vital signs post-dose (BP, HR)[i,j]		X[i]										X[j]	X[i]								
Blood sample for safety[k, u]	X	X	X[u]	X[u]	X[u]	X[u]	X				X	X	X					X	X	X	X
Blood sample for PK		X[l]	X[m]	X[m]	X[m]	X[m]	X[m]					X[n]	X[l]	X[m]	X[m]	X[m]	X[m]	X[m]			
Blood sample for biomarkers		X							X											X[r]	
Blood sample for Autoantibody screen	X																				
Pregnancy test (if applicable)[o]	X	X		X		X		X		X			X		X		X		X	X	X
12-lead ECG[p]	X	X						X			X	X	X						X	X	X
Pulmonary function tests[q]	X							X			X	X	X						X	X	
Skin biopsy		X							X											X[r]	
Questionnaires[s]		X							X		X	X	X						X	X	
Patient's and physician's global assessment		X									X		X							X[g]	
Patient interference with skin		X									X		X							X[g]	
Tendon Friction Rubs[t]		X							X												
Randomization (IxRS)		X																			

Table 7-1 Schedule of evaluations - amended

	Scr	Main Treatment Phase (Double-blind)												Long-term Extension Phase							
	Scr Phase	Dose-Titration Period						Maintenance Period						Dose-Titration Period					Open-label Extension Period	Safety Follow-up	
**Visit**	**V0**	**V1**	**V2**	**V3**	**V4**	**V5**	**V6**	**V7**	**V8**	**V9**	**V10**	**V11**	**V12[a]**	**V13**	**V14**	**V15**	**V16**	**V17**	**V18 – Vn (q 12 wk)**	**TV[b]**	**SFU**
**Week**	-2	0	2	4	6	8	10	12	14	20	26	39	52	54	56	58	60	62	64 – n		
**Day**	**-14 to -1**	**0**	**14 ± 2**	**28 ± 2**	**42 ± 2**	**56 ± 2**	**70 ± 2**	**84 ± 2**	**98 ± 2**	**140 ± 2**	**182 ± 2**	**273 ± 14**	**364 ± 14**	**378 ± 2**	**392 ± 2**	**406 ± 2**	**420 ± 2**	**434 ± 2**	**448 – (n + 84) ± 14**		**30 + 5**
Dispensation of study drug		X	X	X	X	X	X	X	X	X	X	X	X	X	X	X	X	X	X		
Drug accountability			X	X	X	X	X	X	X	X	X	X	X	X	X	X	X	X	X	X	
Adverse events recording	X	X	X	X	X	X	X	X	X	X	X	X	X	X	X	X	X	X	X	X	X
Prior and concomitant therapy	X	X	X	X	X	X	X	X	X	X	X	X	X	X	X	X	X	X	X	X	X
Survival status																					X

Abbreviations: BP = blood pressure; ECG = electrocardiogram; TV = Termination Visit; SFU = 30-Day Safety Follow-up (30 [+5] days after the last dose of study drug); HR = heart rate; IxRS = telephone-based or web-based response system; mRSS = modified Rodnan skin score; PK = pharmacokinetics; q 12 wk = every 12 weeks; Scr = Screening.

Note: Day 0 (Visit 1) is the baseline visit.

a Visit 12 (Week 52) is the last visit in the Maintenance Period of the Main Treatment phase and the first (baseline) visit in the Long-term Extension phase.
b To be performed as soon as possible after withdrawal of study medication.
c Height to be measured only at the screening visit
d Patient to be provided with 7-day diary, to complete daily for 7 consecutive days leading up to Visit 1
e Patient and physician assessment of Raynaud's disease (see Section 7.6.12)
f Patient to be provided with 7-day diary, to complete daily for 7 consecutive days leading up to Visit 8.
g Must not be performed if TV is after V12.
h Blood pressure and heart rate will be measured at all visits with the patient in a sitting position, after the patient has been at rest for at least 5 minutes. The same arm is always used for these measurements (see Section 7.6.5).
i Vital signs to be measured within 20 minutes prior to each post-dose PK sampling (see Table 7–2)
j Vital signs to be measured within 20 minutes prior to PK sampling (see Table 7–2)

Table 7-1 Schedule of evaluations - amended

	Scr	Main Treatment Phase (Double-blind)												Long-term Extension Phase							
	Scr Phase	Dose-Titration Period						Maintenance Period						Dose-Titration Period					Open-label Extension Period	Safety Follow-up	
**Visit**	**V0**	**V1**	**V2**	**V3**	**V4**	**V5**	**V6**	**V7**	**V8**	**V9**	**V10**	**V11**	**V12[a]**	**V13**	**V14**	**V15**	**V16**	**V17**	**V18 – Vn (q 12 wk)**	**TV[b]**	**SFU**
**Week**	-2	0	2	4	6	8	10	12	14	20	26	39	52	54	56	58	60	62	64 – n		
**Day**	**-14 to -1**	**0**	**14 ± 2**	**28 ± 2**	**42 ± 2**	**56 ± 2**	**70 ± 2**	**84 ± 2**	**98 ± 2**	**140 ± 2**	**182 ± 2**	**273 ± 14**	**364 ± 14**	**378 ± 2**	**392 ± 2**	**406 ± 2**	**420 ± 2**	**434 ± 2**	**448 – (n + 84) ± 14**		**30 + 5**

k Coagulation tests to be performed at a local laboratory

l Samples for PK to be taken 1 – 2 hours and 3 – 4 hours after the morning dose of study medication (see Table 7–2)

m Sample for PK to be taken up to 1 hour before the morning dose of study medication (trough) (see Table 7–3)

n Samples for PK to be taken up to 1 hour before the morning dose of study medication (trough) and 2 – 3 hours after the morning dose of study medication (peak) (see Table 7–4)

o For women of childbearing potential only. Serum pregnancy test is required only at screening; pregnancy testing (urine) will be done every 4 weeks and should continue until 30 days after the last dose of study drug. When pregnancy testing coincides with a visit, the test should be performed at the site, but otherwise should be performed at home.

p Standard ECG (12-lead ECG) will be recorded with the patient in the supine position, after the patient has been at rest for at least 5 minutes (see Section 7.6.7).

q Pulmonary function tests include forced vital capacity (FVC) and diffusion capacity of the lung for carbon monoxide (DL_{CO}).

r Must be done at a TV if this visit occurs before Visit 8.

s Questionnaires include Scleroderma Health Assessment Questionnaire (SHAQ), Short Form 36 (SF-36), Patient-Reported Outcomes Measurement Information System (PROMIS)-29, and University of California, Los Angeles, Scleroderma Clinical Trial Consortium Gastrointestinal Scale (UCLA SCTC GIT) 2.0.

t Assessment of the presence or absence of tendon friction rubs

u Additional monitoring may apply for patients with impaired renal and liver function at Visit 2, 3, 4, 5 as needed (see section 7.6.2)

7.1.2.3 Visits 2 through to 6 - Main treatment phase – Dose-titration period

Old text:

At these study visits, the patient should attend the clinic without having taken the morning dose of study medication.

During the dose titration period of the main treatment phase, the following assessments will be performed according to the Schedule of evaluations (Section 7.1.1):

- Vital signs (blood pressure and heart rate). Blood pressure and heart rate will be measured at all visits with the patient in a sitting position, after the patient has been at rest for at least 5 minutes. The same arm is always used for these measurements (see Section 7.6.5).
- Digital ulcer net burden assessment (see Section 7.6.10)
- Recording and assessment of AEs (see Section 7.5)
- Blood samples for safety Visit 6 (see Section 7.6.2)
- Blood sample for PK up to 1 hour before the morning dose of study medication (see Section 7.4)
- Pregnancy test (urine) for all women of childbearing potential (Visit 3 and Visit 5) (see Section 7.6.1)
- Concomitant therapy
- Dose titration according to the titration algorithm (see Section 4.1.2)
- Dispensation of study drug and drug accountability

New text:

At these study visits, the patient should attend the clinic without having taken the morning dose of study medication.

During the dose titration period of the main treatment phase, the following assessments will be performed according to the Schedule of evaluations (Section 7.1.1):

- Vital signs (blood pressure and heart rate). Blood pressure and heart rate will be measured at all visits with the patient in a sitting position, after the patient has been at rest for at least 5 minutes. The same arm is always used for these measurements (see Section 7.6.5).
- Digital ulcer net burden assessment (see Section 7.6.10)
- Recording and assessment of AEs (see Section 7.5)
- Blood samples for additional safety for patients with impaired renal and liver function at Visit 2, 3, 4, 5 as needed (see Section 7.6.2)
- Blood samples for safety Visit 6 (see Section 7.6.2)
- Blood sample for PK up to 1 hour before the morning dose of study medication (see Section 7.4)

- Pregnancy test (urine) for all women of childbearing potential (Visit 3 and Visit 5) (see Section 7.6.1)
- Concomitant therapy
- Dose titration according to the titration algorithm (see Section 4.1.2)
- Dispensation of study drug and drug accountability

7.1.2.8 Termination visit

Old text:

The termination visit will be performed if the patient is withdrawn from study medication for any reason except death or lost to follow-up, and should occur as soon as possible after the patient receives his/her last dose of study drug. Assessments and procedures at this visit include the following:

- Questionnaires (see Section 7.6.13)
- Smoking history
- Physical examination, including weight
- Vital signs (blood pressure and heart rate). Blood pressure and heart rate will be measured at all visits with the patient in a sitting position, after the patient has been at rest for at least 5 minutes. The same arm is always used for these measurements (see Section 7.6.5)
- mRSS (see Section 7.6.6)
- Digital ulcer net burden assessment (see Section 7.6.10)
- Patient's and physician's global assessment (see Section 7.6.15)
- Patient interference with skin assessment (see Section 7.6.16)
- ~~Assessment for the presence or absence of tendon friction rubs (see Section 7.6.17)~~

New text:

The termination visit will be performed if the patient is withdrawn from study medication for any reason except death or lost to follow-up, and should occur as soon as possible after the patient receives his/her last dose of study drug. Assessments and procedures at this visit include the following:

- Questionnaires (see Section 7.6.13)
- Smoking history
- Physical examination, including weight
- Vital signs (blood pressure and heart rate). Blood pressure and heart rate will be measured at all visits with the patient in a sitting position, after the patient has been at rest for at least 5 minutes. The same arm is always used for these measurements (see Section 7.6.5)

- mRSS (see Section 7.6.6)
- Digital ulcer net burden assessment (see Section 7.6.10)
- Tender and swollen joint count assessment (not needed after V12, see Section 7.6.14)
- Patient's and physician's global assessment (not needed after V12, see Section 7.6.15)
- Patient interference with skin assessment (not needed after V12, see Section 7.6.16)

7.1.2.10 Premature discontinuation of study drug treatment

Old text:

In the event of premature discontinuation of study drug treatment, patients must undergo the same procedures as outlined for the termination visit (Section 7.1.2.8) and then undergo evaluation at the safety follow-up visit, 30 (+5) days later (Section 7.1.2.9).

The reason for premature discontinuation of study drug treatment or early withdrawal from the study must be recorded in the eCRF (please refer to Section 5.2).

If the patient discontinues study ~~participation~~ prematurely during the main treatment phase (Week 0 – Week 52) he/she will be invited to come for limited assessments (mRSS, FVC, DL_{CO} and PROs) at Week 12, Week 26, Week 39 and Week 52, depending on the timing of withdrawal. This will be in addition to the termination visit assessments (see Section 7.1.2.8), provided that the patient does not object to collection of study data after withdrawal of consent. If the discontinuation occurs before Visit 8 (Week 14), a skin biopsy will also be performed.

New text:

In the event of premature discontinuation of study drug treatment, patients must undergo the same procedures as outlined for the termination visit (Section 7.1.2.8) and then undergo evaluation at the safety follow-up visit, 30 (+5) days later (Section 7.1.2.9).

The reason for premature discontinuation of study drug treatment or early withdrawal from the study must be recorded in the eCRF (please refer to Section 5.2).

If the patient discontinues study drug prematurely during the main treatment phase (Week 0 – Week 52) he/she will be invited to come for limited assessments (mRSS, FVC, DL_{CO} and PROs) at Week 12, Week 26, Week 39 and Week 52, depending on the timing of withdrawal. This will be in addition to the termination visit assessments (see Section 7.1.2.8), provided that the patient does not object to collection of study data after withdrawal of consent. If the discontinuation occurs before Visit 8 (Week 14), a skin biopsy will also be performed.

7.6.2 Laboratory parameters

Old text:

The following safety laboratory parameters will be analyzed in a central laboratory (apart from coagulation tests, which will be analyzed at a local laboratory) at the screening visit (Visit 0), Visit 1 (Day 0), Visit 6 (Week 10), Visit 11 (Week 39), Visit 12 (Week 52), Visit 17 (Week 62), every 12 weeks during the Open-label extension period, termination visit, and safety follow-up visit:

- Hematology: WBC differential count, erythrocytes, hemoglobin, hematocrit and platelets
- Coagulation tests: activated partial thromboplastin time (aPTT) and international normalized ratio (INR) (to be performed at a local laboratory)
- Clinical chemistry: aspartate aminotransferase (AST), alanine aminotransferase (ALT), alkaline phosphatase (AP), gamma glutamyl transferase (GGT), ~~glutamate dehydrogenase (GLDH)~~, CK, sodium, potassium, magnesium, calcium, phosphate, creatinine, urea, uric acid, total bilirubin, total protein, serum albumin.

At latest, at the end of the study when all planned analyses are completed, all blood samples will be destroyed. The name and address for the central laboratory service provider can be found in the documentation supplied by the vendor.

Note: One re-assessment of laboratory parameters is allowed during the screening phase to re-assess the eligibility of patients.

New text:

The following safety laboratory parameters will be analyzed in a central laboratory (apart from coagulation tests, which will be analyzed at a local laboratory) at the screening visit (Visit 0), Visit 1 (Day 0), Visit 6 (Week 10), Visit 10 (Week 26), Visit 11 (Week 39), Visit 12 (Week 52), Visit 17 (Week 62), every 12 weeks during the Open-label extension period, termination visit, and safety follow-up visit:

- Hematology: WBC differential count, erythrocytes, hemoglobin, hematocrit and platelets
- Coagulation tests: activated partial thromboplastin time (aPTT) and international normalized ratio (INR) (to be performed at a local laboratory)
- Clinical chemistry: aspartate aminotransferase (AST), alanine aminotransferase (ALT), alkaline phosphatase (AP), gamma glutamyl transferase (GGT), CK, sodium, potassium, magnesium, calcium, phosphate, creatinine, urea, uric acid, total bilirubin, total protein, serum albumin.

Patients with isolated AST or ALT >3xULN or bilirubin >2xULN at screening and/or baseline will have additional analysis of coagulation tests, AST, ALT, AP, GGT, total bilirubin and serum albumin until normalization and/ or stabilization at Visit 2 (Day 14), Visit 3 (Day 28), Visit 4 (Day 42), Visit 5 (Day 56), as needed. Worsening of the parameters in the order of 2-fold from baseline values will be considered clinically significant [22] and additional diagnostic procedures may be requested.

Patients entering the trial with eGFR 15-29 mL/min/1.73m^2 will be undergo additional serum creatinine and eGFR calculation until normalization and/ or stabilization at Visit 2 (Day 14), Visit 3 (Day 28), Visit 4 (Day 42), and Visit 5 (Day 56), as needed. A decrease in eGFR equal or greater than 5ml/min/1.73m^2 in these patients (eGFR 15-29 ml/min/1.73m^2) during the study or a reduction of 25% or more in 12 months will be considered clinically significant [23,24].

At latest, at the end of the study when all planned analyses are completed, all blood samples will be destroyed. The name and address for the central laboratory service provider can be found in the documentation supplied by the vendor.

Note: One re-assessment of laboratory parameters is allowed during the screening phase to re-assess the eligibility of patients.

8.2 Analysis sets

Old text:

Efficacy and safety analyses will be performed in patients valid for the full analysis set (FAS). This is defined as all patients randomized and treated with study medication. Investigators will be instructed not to make the call to the IxRS system until they are certain the patient is valid to be treated with study medication, on the day that study medication is to start. In that way, we expect all patients who are randomized also to be treated, so the FAS population would then be consistent with the full intent-to-treat (ITT) definition.

For the primary and secondary exploratory efficacy and safety endpoints, as a supportive analysis, a per protocol set (PPS) will also be defined, including patients who meet the major inclusion and exclusion criteria at randomization that may affect efficacy, who are not taking excluded concomitant medications during the study that could have an effect on efficacy (not including ~~escape~~ medication after Week 26), have the mRSS assessed at baseline and at least once during the main treatment phase and who are at least 80% compliant with study medication. A full definition of valid for PPS will be given in the SAP.

New text:

Efficacy and safety analyses will be performed in patients valid for the full analysis set (FAS). This is defined as all patients randomized and treated with study medication. Investigators will be instructed not to make the call to the IxRS system until they are certain the patient is valid to be treated with study medication, on the day that study medication is to start. In that way, we expect all patients who are randomized also to be treated, so the FAS population would then be consistent with the full intent-to-treat (ITT) definition.

For the primary and secondary exploratory efficacy and safety endpoints, as a supportive analysis, a per protocol set (PPS) will also be defined, including patients who meet the major inclusion and exclusion criteria at randomization that may affect efficacy, who are not taking excluded concomitant medications during the study that could have an effect on efficacy (not including rescue medication after Week 26), have the mRSS assessed at baseline and at least once during the main treatment phase and who are at least 80% compliant with study medication. A full definition of valid for PPS will be given in the SAP.

12 Reference list

New text:

22. Regev A, Seeff LB, Merz M, Ormarsdottir S, Aithal GP, Gallivan J, Watkins PB. Causality Assessment for Suspected DILI During Clinical Phases of Drug Development. Drug Saf (2014) 37 (Suppl 1):S47–S56.

23. NICE clinical guideline 182 “Chronic kidney disease: early identification and management of chronic kidney disease in adults in primary and secondary care”, issued in July 2014 (guidance.nice.org.uk/cg182).

24. MacGregor MS and Taal MW. Clinical Practice Guidelines - Detection, Monitoring and Care of Patients with CKD. UK renal association, 5th edition, 2011 (www.renal.org/guidelines).

39. Steen VD and Medsger TA. Long-Term Outcomes of Scleroderma Renal Crisis. Ann Intern Med. 2000;133:600-603.

40. Shanmugam VK and Steen VD. Renal disease in scleroderma: an update on evaluation, risk stratification, pathogenesis and management. Curr Opin Rheumatol. 2012 November ; 24(6): 669–676.

14.2 Definition of Systemic Sclerosis Renal Crisis

Old text:

The definition of systemic sclerosis hypertensive renal crisis is independent of whether or not concomitant antihypertensive medications are used.

...

At least 1 of the following 5 features:

1. Serum Creatinine: increase of ≥ 50% above baseline*
2. Proteinuria: ≥ 2+ by dipstick confirmed by protein: creatinine ratio > ULN
3. Hematuria: ≥ 2+ by dipstick or > 10 RBCs (red blood cells) /HPF (high power field) (without menstruation)
4. Thrombocytopenia: < 100,000 platelets/mm^3
5. Hemolysis: by blood smear or increased reticulocyte count

**Historical baseline creatinine of the patient*

New text:

Scleroderma renal crisis is defined as the new onset of severe hypertension associated with a rapid increase in serum creatinine concentration, microangiopathic hemolytic anemia, or both [39]. Systemic sclerosis renal crisis usually manifests with acute onset of moderate-to-severe 'accelerated' hypertension and oliguric renal failure [40].

The definition of systemic sclerosis hypertensive renal crisis is independent of whether or not concomitant antihypertensive medications are used.

...

At least 1 of the following 5 features:

1. Serum Creatinine: increase of ≥ 50% above baseline*
2. Proteinuria: ≥ 2+ by dipstick confirmed by protein: creatinine ratio > ULN
3. Hematuria: ≥ 2+ by dipstick or > 10 RBCs/High Power Field [HPF] (without menstruation)
4. Thrombocytopenia: < 100,000 platelets/mm^3
5. Hemolysis: by blood smear or increased reticulocyte count

*Historical baseline creatinine of the patient

13.2 Amendment 4

Amendment 4 was approved on 24 FEB 2016.

13.2.1 Overview of changes to the study

With this amendment, the list of secondary endpoints was changed to include a hierarchy of testing. The chosen key secondary endpoint is the CRISS and the other endpoints in the hierarchy are components of this endpoint. Since 2008, researchers at 4 scleroderma centers in the US (United States) with the participation of the Scleroderma Clinical Trial Consortium (SCTC) have been developing a combined response index known as CRISS based on prospective, data-driven, consensus building techniques [42]. The result of this effort was given approval by the American Colleague of Rheumatology (ACR [41]). The use of CRISS in clinical trials is believed to greatly facilitate the interpretation of results and assess the impact of a pharmacological agent on overall disease activity or severity.

Based on feedback from the US Food and Drug Administration, the protocol was adjusted to clearly differentiate between withdrawal from the study (ie, withdrawal of informed consent) or discontinuation of study treatment (with continued data collection). This change mainly affected Section 5.2, but wording was also adapted in several other sections.

Further changes to the protocol, eg, based on feedback from investigators, included:

- Clarification that at Visit 6 no further dose increase is possible.
- Addition of a clearer definition for dcSSc disease criterion to inclusion criterion 4.
- Addition of local hemoglobin measurements to visits with limited assessments.
- Clarification of the timing of the vital status check for patients who prematurely discontinue study medication before Week 52.
- Correction of inconsistencies.

Correction of typographical errors and minor editorial changes are not specified in the list of changes below. New text is displayed as underlined; text that was deleted is presented as strike-through text.

13.2.2 Changes to the protocol text

General change

In several sections of the protocol, the term “withdrawal” was replaced by “discontinuation/ discontinuation of study medication” if it did not refer to withdrawal of informed consent. Individual changes are marked by footnotes in the body of the protocol, but due to the high number of changes, not all are provided in the list of changes below.

2. Study objectives - amended

Old text:

- Efficacy:
  - ~~mRSS progression rate (defined as increase in mRSS by > 5 units and ≥ 25% from baseline) and mRSS regression rate (defined as decrease in mRSS by > 5 units and ≥ 25% from baseline)~~
  - Patient’s ~~and~~ physician’s global assessment

- ~~HRQoL using Short Form 36 (SF-36) and the Scleroderma Health Assessment Questionnaire (SHAQ)~~
- ~~Digital ulcer net burden (defined as total number of ulcers at time point minus number of ulcers at baseline) and proportion of patients who do not develop new ulcers~~
- Change in FVC (forced vital capacity) % predicted ~~and percent DL_{CO} (carbon monoxide diffusing capacity) % predicted~~
- ~~Combined Response Index for Systemic Sclerosis (CRISS)~~
- ~~Need for Rescue Therapy (see definition in Section 6.9)~~

New text:

- Efficacy:
  - Key secondary objective
    - American College of Rheumatology Combined Response Index for Systemic Sclerosis (CRISS)
  - Further secondary objectives
    - Health Assessment Questionnaire disability index (HAQ-DI) domain (separately from the Scleroderma Health Assessment Questionnaire [SHAQ] as part of the calculation of the CRISS algorithm)
    - Patient's global assessment
    - Physician's global assessment
    - Change in FVC (forced vital capacity) % predicted

4.1.2 Main treatment phase (Week 0 to Week 52) – amended

Old text:

To maintain blinding of the treatment arms, patients randomized to the placebo group will undergo sham titration from Visit 1 onwards during the dose-titration period according to the rules of the dose titration scheme.

New text:

To maintain blinding of the treatment arms, patients randomized to the placebo group will undergo sham titration from Visit 1 onwards during the dose-titration period according to the rules of the dose titration scheme. At Visit 6, the last visit of the Titration Phase, no further increase in study medication will be possible (see Figure 4-1).

4.1.4 Dose Interruptions

Old text:

- Interruptions ~~of any length~~ in the open-label extension period: treatment can be restarted at the discretion of the investigator at 0.5 mg TID lower than the last dose.

New text:

- Interruptions in the **open-label extension period**:

- ≤28 days: treatment can be restarted at the discretion of the investigator at 0.5 mg TID lower than the last dose.
- >28 days: discontinue the patient from study medication.

4.1.5 Termination visit and safety follow-up visit - amended

Old text:

If the patient discontinues study drug prematurely during the main treatment phase (Week 0 – Week 52) he/she will be invited to come for limited assessments (mRSS, FVC, DL_{CO} and PROs) at Week 12, Week 26, Week 39 and Week 52, depending on the timing of withdrawal.

New text:

If the patient discontinues study drug prematurely during the main treatment phase (Week 0 – Week 52) he/she will be invited to come for limited assessments (mRSS, pulmonary function test [FVC and DL_{CO} including hemoglobin measurement at the local laboratory], and patient reported outcome [PROs]) at Week 12, Week 26, Week 39 and Week 52, depending on the timing of discontinuation.

5.1.1 Inclusion criteria

Old text:

4. dcSSc according to the LeRoy criteria[3]

New text:

4. dcSSc according to the LeRoy criteria[3], ie, skin fibrosis proximal to the elbows and knees in addition to acral fibrosis

5.1.2 Exclusion Criteria - amended

Old text:

5. Pulmonary-related criteria
   - Diagnosed ~~PAH~~ as determined by right heart catheterization

New text:

5. Pulmonary-related criteria
   - Diagnosed pulmonary hypertension as determined by right heart catheterization

5.2.1 Withdrawal - amended

Old text:

Patients *must* be withdrawn from the study for the following reasons:

- At their own request or at the request of their legally acceptable representative at any time during the study and without giving reasons, a patient may decline to participate further. The patient will not suffer any disadvantage as a result.
- If, in the investigator's opinion, continuation of the study would be harmful to the patient's well-being
- Occurrence of AEs or intercurrent diseases which the investigator judges unacceptable for continuation of participation in the study
- Occurrence of adverse drug reactions, which in the investigator's opinion have a negative impact on the patient's individual risk-benefit ratio. (Investigators are obliged to reassess the patient's individual risk-benefit ratio on a continuous basis. Factors such as anticipated treatment effect, progression of underlying disease, occurrence of side effects, and alternative treatment options must be considered.)
- Non-compliance with the conditions for the trial or instructions by the investigator
- If treatment is interrupted for > 3 consecutive days (9 doses)[144] during the dose titration period of the main treatment phase
- If treatment is interrupted for > 14 consecutive days during the maintenance period of the main treatment phase or the dose-titration period of the long-term extension phase
- In case of pregnancy or breast feeding.
- In case a female patient of childbearing potential is not compliant with 4-weekly pregnancy testing or with contraceptive measures.
- Participation in another clinical trial. Participation in observational registries is allowed.
- In case no further dose reduction is possible and the patient does not tolerate the lowest possible dosage (0.5 mg TID) of study drug

Patients *may* be ~~withdrawn from the study~~ for the following reasons:

- At the specific request of the sponsor and in liaison with the investigator (eg, safety concerns)
- A relative decline in FVC % predicted by ≥ 10% (eg, a change of FVC from 60% to 54%) or a relative decline in FVC % predicted between 5 to < 10% with associated relative decline in DL_{CO} % predicted by ≥ 15%, provided that the decline in FVC results in FVC <75% of predicted (confirmed by repeat pulmonary function testing within 1 month). Please refer to Section 6.9 for the option of adding rescue[145] therapy.

[144] Per Amendment 1
[145] Per Amendment 1

- An absolute increase in mRSS by > 5 units and ≥ 25% (Please refer to Section 6.9 for the option of adding rescue[146] therapy), life-threatening or organ-threatening event attributable to SSc (such as renal crisis, digital gangrene, development of new PAH on right heart catheterization) or not attributable to SSc.

A patient who, for any reason (eg, failure to satisfy the selection criteria), terminates the study before the time point used for the definition of "dropout" (see below) is regarded a "screening failure". Restarting the defined set of screening procedures to enable the "screening failure" patient's participation at a later time point is not allowed.

[…]

If the patient discontinues study drug[147] prematurely during the main treatment phase (Week 0 – Week 52) he/she will be invited to come for limited assessments (mRSS, FVC, DL_{CO} and patient-reported outcomes [PROs]) at Week 12, Week 26, Week 39, and Week 52, depending on the timing of ~~withdrawal~~.

[…]

A patient may withdraw from further participation in the study and, ~~unless he/she signs an objection form~~, the following information should be obtained and documented in the eCRF:

- Vital status
- Outcome of SAEs
- Efficacy endpoints at Weeks 12, 26, 39, and 52 (depending on timing of ~~withdrawal~~)

The information should be collected in the eCRF until the safety follow-up visit 30 (+5) days after the last intake of study drug. Serious adverse events that occur within 30 (+5) days after the last dose of study drug will be followed up until resolution, if possible.

~~For patients who object to further release of information, vital status only should be obtained.~~ The measures taken for follow up must be documented in the site medical record.

New text:

5.2.1.1 Withdrawal from the study

Patients *must* be withdrawn from the study for the following reasons:

- At their own request or at the request of their legally acceptable representative at any time during the study and without giving reasons, a patient may decline to participate further (withdrawal from study). The patient will not suffer any disadvantage as a result.
- Participation in another clinical trial after Visit 12. Participation in observational registries is allowed.

5.2.1.2 Discontinuation of study medication

[146] Per Amendment 1
[147] Per Amendment 1

Patients *must* be discontinued from the study medication for the following reasons:

- If, in the investigator's opinion, continuation of the study would be harmful to the patient's well-being
- Occurrence of AEs or intercurrent diseases which the investigator judges unacceptable for continuation of participation in the study
- Occurrence of adverse drug reactions, which in the investigator's opinion have a negative impact on the patient's individual risk-benefit ratio. (Investigators are obliged to reassess the patient's individual risk-benefit ratio on a continuous basis. Factors such as anticipated treatment effect, progression of underlying disease, occurrence of side effects, and alternative treatment options must be considered.)
- Non-compliance with the conditions for the trial or instructions by the investigator
- If treatment is interrupted for >3 consecutive days (9 doses) during the dose titration period of the main treatment phase
- If treatment is interrupted for >14 consecutive days during the maintenance period of the main treatment phase or the dose-titration period of the long-term extension phase
- If treatment is interrupted for >28 days during the open-label extension period (of the long-term extension phase)
- In case of pregnancy or breast feeding.
- In case a female patient of childbearing potential is not compliant with 4-weekly pregnancy testing or with contraceptive measures.
- ~~Participation in another clinical trial. Participation in observational registries is allowed.~~
- In case no further dose reduction is possible and the patient does not tolerate the lowest possible dosage (0.5 mg TID) of study drug

Patients *may* be discontinued from the study medication for the following reasons:

- At the specific request of the sponsor and in liaison with the investigator (eg, safety concerns)
- A relative decline in FVC % predicted by ≥ 10% (eg, a change of FVC from 60% to 54%) or a relative decline in FVC % predicted between 5 to < 10% with associated relative decline in DL_{CO} % predicted by ≥ 15%, provided that the decline in FVC results in FVC <75% of predicted (confirmed by repeat pulmonary function testing within 1 month). Please refer to Section 6.9 for the option of adding rescue therapy.
- An absolute increase in mRSS by > 5 units and ≥ 25% (Please refer to Section 6.9 for the option of adding rescue therapy), life-threatening or organ-threatening event attributable to SSc (such as renal crisis, digital gangrene, development of new PAH on right heart catheterization) or not attributable to SSc.

5.2.1.3 Handling of withdrawals or discontinuations from study medication

A patient who, for any reason (eg, failure to satisfy the selection criteria), terminates the study before the time point used for the definition of "dropout" (see below) is regarded a "screening

failure". Restarting the defined set of screening procedures to enable the "screening failure" patient's participation at a later time point is not allowed.

[…]

If the patient discontinues study drug prematurely during the main treatment phase (Week 0 – Week 52) he/she will be invited to come for limited assessments (mRSS, pulmonary function test [FVC and DL_{CO} including hemoglobin measurement at the local laboratory], and PROs) at Week 12, Week 26, Week 39 and Week 52, depending on the timing of discontinuation.

[…]

A patient may withdraw from further participation in the study and, unless he/she objects to further data collection, the following information should be obtained and documented in the eCRF:

- Vital status (at Week 52)
- Outcome of SAEs
- Efficacy endpoints at Weeks 12, 26, 39, and 52 (depending on timing of discontinuation of study medication)

The information should be collected in the eCRF until the safety follow-up visit 30 (+5) days after the last intake of study drug. Serious adverse events that occur within 30 (+5) days after the last dose of study drug will be followed up until resolution, if possible.

If patients refuse to be contacted via telephone (eg, patient objects to further release of information or are lost to follow-up), every effort should be made to obtain vital status (dead or alive) information at Week 52 through consultation of public databases, wherever allowed by local regulations.

5.2.2 Replacement

Old text:

There will be no replacement of randomized patients who withdraw from the study.

New text:

There will be no replacement of randomized patients who withdraw from the study or prematurely discontinue study medication.

6.4.1 Selection of doses in the study

Old text:

~~The respective single doses of riociguat should be taken at least 6 hours apart.~~

New text:

The intervals between drug intake should be 6 to 8 hours.

6.5 Blinding - amended

Old text:

Any patient ~~withdrawn from the trial~~ will remain under medical supervision until discharge or transfer is medically acceptable. Patients who withdraw should take part at the termination visit (refer to Section 7.1.2.8) and the safety follow-up visit (refer to Section 7.1.2.9).

New text:

Any patient who discontinues study medication will remain under medical supervision until discharge or transfer is medically acceptable. Patients who withdraw from the study or discontinue study medication should take part at the termination visit (refer to Section 7.1.2.8) and the safety follow-up visit (refer to Section 7.1.2.9).

7.1.1 Tabulated overview - amended

Old text:

Not applicable. A new footnote was added for the Main Treatment Phase to the schedule of events in Table 7.1.

The visit window for V12 was changed. Original visit window: ±14 days.

New text:

v All patients who discontinue study drug prematurely during the Main Treatment Phase (Week 0 – Week 52) will be invited to come for limited assessments (see Sections 7.1.2.10 and 7.6.2).

New visit window for V12: -14 to +2 days.

7.1.2.10 Premature discontinuation of study drug treatment - amended

Old text:

If the patient discontinues study drug prematurely during the main treatment phase (Week 0 – Week 52) he/she will be invited to come for limited assessments (mRSS, FVC~~,~~ DL_{CO} and PROs) at Week 12, Week 26, Week 39 and Week 52, depending on the timing of ~~withdrawal~~.

New text:

If the patient discontinues study drug prematurely during the main treatment phase (Week 0 – Week 52) he/she will be invited to come for limited assessments (mRSS, pulmonary function test [FVC and DL_{CO} including hemoglobin measurement at the local laboratory], and PROs) at Week 12, Week 26, Week 39 and Week 52, depending on the timing of discontinuation.

7.3 Efficacy - amended

Old text:

Secondary efficacy outcome measures

- ~~mRSS progression rate (defined as increase in mRSS by > 5 units and > 25% from baseline) and mRSS regression rate (defined as decrease in mRSS by > 5 units and > 25% from baseline)~~

- Patient's ~~and~~ physician's global assessment
- ~~HRQoL using SF-36 and the SHAQ~~
- ~~Digital ulcer net burden (defined as total number of ulcers at time point minus number of ulcers at baseline) and proportion of patients who do not develop new ulcers~~
- Change in FVC (forced vital capacity) % predicted ~~and DL_{CO} (carbon monoxide diffusing capacity) % predicted~~
- ~~Combined Response Index for Systemic Sclerosis (~~CRISS~~)~~, consisting of five variables: mRSS, FVC % predicted, physician and patient global assessments, and HAQ-DI score (from SHAQ patient reported outcome)
- ~~Need for Rescue Therapy (see definition in Section 6.9)~~

[…]

Other exploratory measures:

- All-cause Mortality
- Gastrointestinal involvement as assessed by University of California, Los Angeles, Scleroderma Clinical Trial Consortium Gastrointestinal Scale (UCLA SCTC GIT) 2.0 instrument
- Patient-Reported Outcomes Measurement Information System (PROMIS)-29 (subset of patients)
- Patient interference with skin
- Presence or absence of tendon friction rubs
- Pain VAS
- Change in BMI
- Joint involvement using tender and swollen joint counts
- New items for Raynaud's response index excluding items included in secondary outcome measures
    - Patient and physician assessment of Raynaud's phenomenon; pain, numbness, and tingling during a Raynaud's phenomenon attack; Raynaud's condition score, average number of attacks/day; and duration of attacks

New text:

Secondary efficacy outcome measures

Key secondary endpoint:

- CRISS, consisting of five variables: mRSS, FVC % predicted, physician and patient global assessments, and HAQ-DI score (from SHAQ patient reported outcome)

Further secondary endpoints:

- HAQ-DI domain (separately from the SHAQ as part of the calculation of the CRISS algorithm).
- Patient's global assessment

- Physician's global assessment
- Change in FVC (forced vital capacity) % predicted

[…]

Other exploratory measures[148]:

- mRSS progression rate (defined as increase in mRSS by > 5 units and > 25% from baseline) and mRSS regression rate (defined as decrease in mRSS by > 5 units and > 25% from baseline)
- HRQoL using SF-36 and the SHAQ.
- Digital ulcer net burden (defined as total number of ulcers at time point minus number of ulcers at baseline) and proportion of patients who do not develop new ulcers
- Change in DL_{CO} (carbon monoxide diffusing capacity) % predicted
- Need for Rescue Therapy (see definition in Section 6.9)
- All-cause Mortality
- Gastrointestinal involvement as assessed by University of California, Los Angeles, Scleroderma Clinical Trial Consortium Gastrointestinal Scale (UCLA SCTC GIT) 2.0 instrument
- Patient-Reported Outcomes Measurement Information System (PROMIS)-29 (subset of patients)
- Patient interference with skin
- Presence or absence of tendon friction rubs
- Pain VAS
- Change in BMI
- Joint involvement using tender and swollen joint counts
- New items for Raynaud's response index excluding items included in secondary outcome measures
    - Patient and physician assessment of Raynaud's phenomenon; pain, numbness, and tingling during a Raynaud's phenomenon attack; Raynaud's condition score, average number of attacks/day; and duration of attacks

7.6.2 Laboratory parameters - amended

Old text:

- Hematology: WBC differential count, erythrocytes, hemoglobin, hematocrit and platelets

[148] Per Amendment 4

New text:

- Hematology: WBC differential count, erythrocytes, hemoglobin, hematocrit and platelets
  The hemoglobin value required for the pulmonary function tests performed at visits with limited assessments (see Section 7.1.2.10) will be measured at the local laboratory. The hemoglobin value will be entered in the eCRF and used for the calculation of predicted DL_{CO} only.

7.6.8 Pulmonary function testing

Old text:

Printouts from the analyzer must be stored in the patient file and the measured values transferred to the eCRF.

New text:

Printouts from the analyzer must be stored in the patient file and the measured values transferred to the eCRF.

The hemoglobin value required for the pulmonary function tests performed at visits with limited assessments (see Section 7.1.2.10) will be measured at the local laboratory. The hemoglobin value will be entered in the eCRF and used for the calculation of predicted DL_{CO} only.

8.4.2 Efficacy - amended

Old text:

In the efficacy analyses, countries and centers will be clustered by geographic region. The decision on country pooling will be made before unblinding. Statistical analyses will be adjusted for these countries / geographic regions.

The primary analysis will be the change in mRSS from baseline to all assessments post baseline up to Week 52 using mixed model repeated measures (MMRM) with baseline mRSS as a covariate, fixed effects treatment arm and region, the interaction effect between study visit and treatment arm, and patient specific random effects to account for both heterogeneity among patients and correlation among measurements taken on the same patient with an unstructured covariance assumption.

[…]

As a sensitivity analysis, the MMRM using the above model will also be applied to assessments post-baseline up to termination of study medication or initiation of rescue medication. In addition, the MMRM on all assessments will be repeated including additional effects time on rescue medication and time on study medication. MMRM assumes missing at random; mRSS by time of patient ~~withdrawal~~ will be summarized and further methods using multiple imputation that allow for missing data not at random will be investigated. Details of such methods will be described in the SAP.

[…]

Rules for the imputation of missing values where the patient ~~withdraws or dies~~ for the univariate analysis, null / alternative hypothesis statements, and model statements in mathematical notation will be specified in the SAP. Two-sided 95% confidence intervals of treatment (riociguat compared to placebo) differences will be calculated in addition to the statistical significance testing.

The other secondary and exploratory efficacy variables measured on a continuous or semi-continuous scale will analyzed using the MMRM, ANCOVA, and stratified Wilcoxon test, as for the mRSS above.

The time to first mRSS progression and regression and clinical outcome parameters will be analyzed using the log-rank test comparing between treatment groups.

Any binary outcomes, such as the predicted CRISS outcome will be analyzed using Mantel-Haenzel weights, stratified by region. Also as a sensitivity analysis in addition to the time to event analysis, the occurrence of the events mRSS progression and regression and clinical outcome parameters will be analyzed using this method.

Details on any descriptive subgroup analyses will be specified in the SAP. These will include gender and region.

New text:

In the efficacy analyses, countries and centers will be clustered by geographic region. The decision on country pooling will be made before unblinding. Statistical analyses will be adjusted for these countries / geographic regions.

8.4.2.1 Primary endpoint

The primary analysis will be the change in mRSS from baseline to all assessments post baseline up to Week 52 using mixed model repeated measures (MMRM) with baseline mRSS as a covariate, fixed effects treatment arm and region, the interaction effect between study visit and treatment arm, and patient specific random effects to account for both heterogeneity among patients and correlation among measurements taken on the same patient with an unstructured covariance assumption.

[…]

As a sensitivity analysis, the MMRM using the above model will also be applied to assessments post-baseline up to termination of study medication or initiation of rescue medication. In addition, the MMRM on all assessments will be repeated including additional effects time on rescue medication and time on study medication. MMRM assumes missing at random; mRSS by time of patient discontinuation from study medication will be summarized and further methods using multiple imputation that allow for missing data not at random will be investigated. Details of such methods will be described in the SAP.

[…]

Rules for the imputation of missing values where the patient discontinues the study (eg, withdrawal of consent or death) or study medication for the univariate analysis, null / alternative hypothesis statements, and model statements in mathematical notation will be specified in the SAP. Two-sided 95% confidence intervals of treatment (riociguat compared to placebo) differences will be calculated in addition to the statistical significance testing.

8.4.2.2 Secondary and exploratory endpoints

General aspects

The other secondary and exploratory efficacy variables measured on a continuous or semi-continuous scale will analyzed using the MMRM, ANCOVA, and stratified Wilcoxon test, as for the mRSS above.

The time to first mRSS progression and regression and clinical outcome parameters will be analyzed using the log-rank test comparing between treatment groups.

Any binary outcomes, such as the predicted CRISS outcome will be analyzed using Mantel-Haenzel weights, stratified by region. Also as a sensitivity analysis in addition to the time to event analysis, the occurrence of the events mRSS progression and regression and clinical outcome parameters will be analyzed using this method.

Details on any descriptive subgroup analyses will be specified in the SAP. These will include gender and region.

Hierarchical testing of secondary endpoints

The following secondary endpoints will be tested in a hierarchical fashion at a 2-sided 5% level, only if the primary endpoint of mRSS is shown to be statistically significant at a 2-sided 5% level.

The following order of testing will be applied:

- CRISS
- HAQ-DI (health assessment questionnaire-disability index)
- Patient's global assessment
- Physician's global assessment
- FVC % predicted

For example, if CRISS is statistically significant at a 2-sided 5% level, then the HAQ-DI will be tested, if not the testing procedure will be stopped. This step is repeated further down the list of the 5 secondary endpoints until a non-statistically significant endpoint is reached.

These particular secondary endpoints were chosen to be tested in this hierarchical fashion, because the mRSS and the above additional, secondary variables were considered to have the greatest face validity when designing the CRISS [41].

American College of Rheumatology Combined Response Index for Systemic Sclerosis

Application of CRISS algorithm in a randomized clinical trial is a 2-step process. Firstly, evaluate if patients have met the criterion for not-improved. If yes, these patients are assigned a probability score of 0.0. For the remaining patients, calculate the probability based on change in 5 measures: mRSS, FVC %, HAQ-DI, patient’s global assessment, and physician's global assessment, where each measure has a probability score between 0 and 1 [41].

In Step 1, a patient is clinically evaluated to determine whether the patient has improved or not. Expert consensus on the definition of a patient who is not improved during a trial is the following:

A patient is considered not improved and is assigned a probability score of improving equal to 0.0, irrespective of improvement on other core items, if he/she develops:

1. New scleroderma renal crisis,
2. Decline in FVC % predicted ≥15% (relative), confirmed by another FVC % within a month, high resolution computed tomography (HRCT) to confirm interstitial lung disease (if previous HRCT did not show interstitial lung disease) and FVC % predicted below 80% predicted (attributable to SSc),
3. New onset of left ventricular failure (defined as ejection fraction ≤45%) or new onset of pulmonary arterial hypertension requiring treatment (attributable to SSc).

If the patient is determined to exhibit improvement in Step 1 (ie, not assigned a 0.0), Step 2 involves computing the predicted probability of improving (a score between 0.0 and 1.0, inclusive) for each patient using the equation:

$$\frac{exp[-5.54-0.81*\Delta_{MRSS}+0.21*\Delta_{FVC\%}-0.40*\Delta_{Pt-glob}-0.44*\Delta_{MD-glob}-3.41*\Delta_{HAQ-DI}]}{1+exp[-5.54-0.81*\Delta_{MRSS}+0.21*\Delta_{FVC\%}-0.40*\Delta_{Pt-glob}-0.44*\Delta_{MD-glob}-3.41*\Delta_{HAQ-DI}]}$$

where Δ_{MRSS} indicates the change in mRSS from baseline to follow-up, Δ_{FVC} denotes the change in FVC % predicted from baseline to follow-up, $\Delta_{Pt-glob}$ indicates the change in patient global assessment, $\Delta_{MD-glob}$ denotes the change in physician global assessment, and Δ_{HAQ-DI} is the change in HAQ-DI. Note that all changes are absolute changes ($Time_2 - Time_{baseline}$) and that the physician's and patient's global assessment was measured on the Likert scale ranging from 0 to 10, where 0 = excellent, and 10 = extremely poor.

Hence, the CRISS is comprised of 5 variables: mRSS, FVC % predicted, patient's global assessment, physician's global assessment, and HAQ-DI from the SHAQ patient reported outcome. Combined, the 5 variables explained 89.3% of the variability in the data. Individually, when used in a single-variable logistic regression model, mRSS explained 66.3% of the variation, FVC % predicted explained 36.1% of the variation, physician global assessment explained 24.5% of the variation, patient global assessment explained 23.7% of the variation, and HAQ-DI explained 28.5% of the variation. A change in mRSS, FVC % predicted and HAQ-DI are strong indicators of whether a patient is likely to be improved or not. In each scenario, a decrease of mRSS or HAQ-DI from baseline to follow-up and an increase in FVC % predicted corresponds to very high probabilities of improving. For patient's global and physician's global assessment, the association between probability of improving and change in these 2 core components is less evident. Subjects for which the predicted probability is greater or equal to 0.60 are considered improved, while subjects for which the predicted probability is below 0.60 are considered not improved. The 2 groups (riociguat vs. placebo) can then be compared in a 2x2 table using appropriate significance tests. The predicted probabilities obtained using the CRISS can also be assessed as a continuous variable and the distributions of the probability of improving for patients on drug vs. placebo can be compared using non-parametric tests [41]. In this study, the CRISS outcome will be analyzed using Mantel-Haenzel weights, stratified by region, and additionally, the stratified Wilcoxon test will be used as non-parametric test.

However, Step 2 assumes that there is complete data; ie, all 5 components of the CRISS are fully recorded at Week 52. If the subject discontinues from study drug from the study before Week 52, or has one or more missing components of the CRISS at this visit, then a mixed model repeated measures (MMRM) analysis will be implemented to estimate the value or values for those components at Week 52. These estimates will then we used to calculate the

CRISS score at Week 52 as given in Step 2. Subjects for which the predicted probability is greater or equal to 0.60 are considered improved, while subjects for which the predicted probability is below 0.60 are considered not improved. [41] Further details will be provided in the statistical analysis plan.

8.6 Determination of sample size

Old text:

One of the components of ~~secondary~~ efficacy measure mRSS progression is a 5 point or more deterioration from baseline to any assessment in the 12 month study period, this being considered a clinically meaningful change [14].

New text:

One of the components of exploratory efficacy measure mRSS progression is a 5 point or more deterioration from baseline to any assessment in the 12 month study period, this being considered a clinically meaningful change [14].

12. Reference list - amended

New text:

41. Khanna D, Berrocal VJ, Giannini EH, Seibold JR, Merkel PA, Mayes MD, et al. The American College of Rheumatology Provisional Composite Response Index for Clinical Trials in Early Diffuse Cutaneous Systemic Sclerosis. Arthritis Rheumatol. 2016; 68(2):299-311.

42. Khanna D, Lovell DJ, Giannini E, Clements PJ, Merkel PA, Seibold JR, et al. Development of a provisional core set of response measures for clinical trials of systemic sclerosis. Ann.Rheum Dis. 2008;67(5):703-709.

13.3 Amendment 5

Amendment 5 was approved on 18 Aug 2016.

13.3.1 Overview of changes to the study

This amendment was implemented to clarify that the benefit-risk balance for the population in this study (patients with dcSSc) remains positive, despite the potential safety issue that has been reported in a study in patients with PH-IIP leading to early termination of the concerned study. The benefit-risk assessment for patients with dcSSc was re-evaluated by the DMC in 3 meetings and was confirmed to remain positive.

Furthermore, the sample size determination in Section 8.6 was adapted to reflect

- The change in key secondary efficacy endpoint to CRISS (implemented with amendment 4).
- Appropriate ranges for the significance levels and number of patients required.

When scientific advice was initially sought from the Food and Drug Administration (FDA) and European Medicines Agency (EMA) during the set-up of this study, a one-sided significance level of 5%, equivalent to a two-sided significance level of 10%, was proposed, resulting in a total sample size of 102 patients. If such a significance level was achieved, together with a clinically significant effect on the mRSS, then this was considered a potential criterion for efficacy to proceed with the drug to Phase III studies. To make the study more robust, the final protocol specified a two-sided significance level of 5%, resulting in a total sample size of 128 patients.

Additionally, the time point of the CRISS assessment was clarified to be Week 52. The original term “follow-up”, as mentioned in Section 8.4.2.2, was taken from the paper describing the derivation of the CRISS score [41]. This was not referring to a specific visit, but a post-baseline follow-up assessment of a patient to be able to calculate the CRISS. Week 52 has now been specified, this being the planned final visit in the main treatment phase, and to avoid the interpretation that this might have been the post-treatment Safety Follow-up visit.

As of 01 July 2016, Bayer HealthCare AG merged with Bayer AG. Thereby, Bayer HealthCare AG ceased to exist and Bayer AG became its legal successor and automatically took over all of the Bayer HealthCare AG’s rights, obligations and liabilities by law, including the sponsorship for clinical studies currently sponsored by Bayer HealthCare AG.

- The sponsorship information for this study was changed on the title page.
- Additionally, the sponsor for US territory was added. Bayer HealthCare Pharmaceuticals Inc. is and has been the sponsor for the US territory for this study as set forth in FDA Investigational New Drug (IND) form 1571. The reference on the protocol was incomplete in this regard.

Further changes to the protocol included the following clarifications and additions:

- Update of permitted concomitant medication during the long-term extension phase.
- Update of allowed rescue therapy.
- Clarification that biomarker analyses will be reported separately.
- Addition of description of unblinding process for biomarker and PK analysis.

- Clarification of sampling time points for blood samples for PK analysis and documentation requirements.
- Addition of analysis of PK data in relationship of exposure parameters with treatment effects.
- Change in time point for first data cleaning and reporting.
- Clarification for adverse event reporting regarding conditions of SSc.
- Clarification of the wording for exclusion criterion 5.
- Addition of one criterion for discontinuation of the study medication in case patients develop renal disease and hepatic insufficiency in alignment with exclusion criteria 2 and 3.
- Addition of the patient's and physician's global assessment to the visits with limited assessments to ensure that all required variables are available at Week 52 for the analysis of the CRISS.
- Correction of visit window for Visit 18.
- Clarification of allowed dose interruptions in the open-label extension period.

Correction of typographical errors and minor editorial changes are not specified in the list of changes below. New text is displayed as underlined; text that was deleted is presented as strike-through text.

13.3.2 Changes to the protocol text

General change

Due to the change in the sponsor details, the header was adapted in the whole document: the old logo was deleted and replaced by the new logo.

Title page - amended

Old text:

Sponsor: **Bayer ~~HealthCare~~ AG, D-51368 Leverkusen, Germany**

New text:

Sponsor:
Non-US: Bayer AG, D-51368 Leverkusen, Germany
US territory: Bayer Healthcare Pharmaceuticals Inc.,
100 Bayer Boulevard, P.O. Box 915,
Whippany NJ 07981-0915, USA

Synopsis – Number of patients

Old text:

130 randomized patients.

New text:

Up to approximately 130 randomized patients.

1.3 Benefit-risk assessment – amended

Old text:

[Not applicable. New text added in the end of the section.]

New text:

Recently, a potential safety issue has been reported for patients with pulmonary hypertension associated with idiopathic interstitial pneumonias (PH-IIP) and, therefore, the benefit-risk assessment for this indication has changed from positive to unfavorable. Following the data monitoring committee (DMC) recommendation, the study treatment has been terminated. Also per DMC recommendation patients are continuing in a safety follow-up for 4 months after study drug discontinuation and the study is expected to close in September 2016.

A careful review, assessment and analysis will be done by the sponsor with unblinded and clean data originating from clinical study 13605 (RISE-IIP; final clinical study report expected in 2017). Information of health care professionals (via Dear Healthcare Professional Letter), label updates and an update of the risk management plan have been initiated in July 2016 in collaboration with the European Medicines Agency as well as national competent authorities.

The benefit-risk profile for conditions being studied in ongoing clinical studies also has been carefully re-evaluated. In the case of the present study in the dcSSc indication, 2 ad-hoc meetings of the DMC for this study took place, on 23 MAY 2016 and on 25 MAY 2016, followed by a third meeting on 15 AUG 2016. The DMC concluded that this study may continue without amendment or modification. Overall, the benefit-risk balance for this indication remains positive.

2. Study objectives - amended

Old text:

- Key secondary objective:
  - American College of Rheumatology Combined Response Index for Systemic Sclerosis (CRISS)

New text:

- Key secondary objective:
  - American College of Rheumatology Combined Response Index for Systemic Sclerosis (CRISS) at Week 52.

4.1 Design overview

Old text:

In this multinational, multicenter, randomized (1:1), double-blind, placebo-controlled, parallel-group study, a total of approximately 200 patients are planned for enrollment in order

to randomize approximately 130 patients to study drug treatment (approximately 65 patients to the riociguat group and 65 to the placebo group).

New text:

In this multinational, multicenter, randomized (1:1), double-blind, placebo-controlled, parallel-group study, a total of approximately 200 patients are planned for enrollment in order to randomize up to approximately 130 patients to study drug treatment (approximately 65 patients to the riociguat group and 65 to the placebo group).

4.1.4 Dose Interruptions - amended

Old text:

- ≤28 days: treatment can be restarted at the discretion of the investigator at 0.5 mg TID lower than the last dose.
- >28 days: discontinue the patient from study medication.

New text:

- >13 and ≤28 consecutive days: treatment can be restarted at the discretion of the investigator at 0.5 mg TID lower than the last dose.
- >28 consecutive days: discontinue the patient from study medication.

4.1.5 Termination visit and safety follow-up visit - amended

Old text:

If the patient discontinues study drug prematurely during the main treatment phase (Week 0 – Week 52) he/she will be invited to come for limited assessments (mRSS, pulmonary function test [FVC and DL_{CO} including hemoglobin measurement at the local laboratory], ~~and~~ patient-reported outcomes [PROs]) at Week 12, Week 26, Week 39, and Week 52, depending on the timing of discontinuation .

New text:

If the patient discontinues study drug prematurely during the main treatment phase (Week 0 – Week 52) he/she will be invited to come for limited assessments (mRSS, pulmonary function test [FVC and DL_{CO} including hemoglobin measurement at the local laboratory], patient-reported outcomes [PROs], and patient's and physician's global assessment) at Week 12, Week 26, Week 39, and Week 52, depending on the timing of discontinuation.

5.1.2 Exclusion Criteria - amended

Old text:

5. Pulmonary-related criteria
   - ~~Diagnosed~~ pulmonary hypertension as determined by right heart catheterization

New text:

Pulmonary-related criteria

- Any form of pulmonary hypertension as determined by right heart catheterization

5.2.1.2 Discontinuation of study medication

Old text:

Patients must be discontinued from the study medication for the following reasons:

[…]

- Occurrence of adverse drug reactions, which in the investigator's opinion have a negative impact on the patient's individual risk-benefit ratio. (Investigators are obliged to reassess the patient's individual risk-benefit ratio on a continuous basis. Factors such as anticipated treatment effect, progression of underlying disease, occurrence of side effects, and alternative treatment options must be considered.)
- […]
- If treatment is interrupted for >28 days during the open-label extension period (of the long-term extension phase)

New text:

Patients must be discontinued from the study medication for the following reasons:

[…]

- Occurrence of adverse drug reactions, which in the investigator's opinion have a negative impact on the patient's individual risk-benefit ratio. (Investigators are obliged to reassess the patient's individual risk-benefit ratio on a continuous basis. Factors such as anticipated treatment effect, progression of underlying disease, occurrence of side effects, and alternative treatment options must be considered.)
- If a subject, during the course of the study, develops
  a) renal disease that leads to eGFR <15/mL/min/1.73m^2,
  b) hepatic insufficiency classified as Child-Pugh C.
- […]
- If treatment is interrupted for >28 consecutive days during the open-label extension period (of the long-term extension phase)

5.2.1.3 Handling of withdrawals or discontinuations from study medication

Old text:

If the patient discontinues study drug prematurely during the main treatment phase (Week 0 – Week 52) he/she will be invited to come for limited assessments (mRSS, pulmonary function test [FVC and DL_{CO} including hemoglobin measurement at the local laboratory] , ~~and~~ PROs) at Week 12, Week 26, Week 39, and Week 52, depending on the timing of discontinuation.

New text:

If the patient discontinues study drug prematurely during the main treatment phase (Week 0 – Week 52) he/she will be invited to come for limited assessments (mRSS, pulmonary function test [FVC and DL_{CO} including hemoglobin measurement at the local laboratory], PROs, and patient's and physician's global assessment) at Week 12, Week 26, Week 39, and Week 52, depending on the timing of discontinuation.

6.5 Blinding - amended

Old text:

[Not applicable. New text added.]

New text:

Appropriate measures will be taken to maintain blinding of the study team while PK and biomarker analyses are ongoing. Data with a potential to unblind (eg, plasma concentrations) are not to be handled by or shown to the study team members and will only be uploaded into the clinical database after official unblinding. The process of data flow will be documented separately (see also Section 7.4 and Section 7.6.3.).

Statistical analysis as described in the SAP will be done after database lock and authorization of data release according to standard operating procedures.

6.9 Prior and concomitant therapy – amended

Old text:

[…] These treatments will also be allowed as new-onset during the study at the discretion of the investigator to treat SSc-specific adverse events (eg, Raynaud's phenomena, joint inflammation, new onset renal crisis).

[…]

Table 6-2 Prohibited prior and concomitant therapy - amended

Therapy	Timeframe
Chlorambucil, bone marrow transplantation, or total lymphoid irradiation	Any previous therapy[a]
Rituximab or other anti-CD20 antibodies	Within 6 months prior to the screening visit[a]
Infliximab, certolizumab, golimumab, adalimumab, abatacept, leflunomide or tocilizumab	Within 8 weeks prior to the screening visit[a,]
Methotrexate, cyclophosphamide, hydroxychloroquine, cyclosporine A, azathioprine, mycophenolate mofetil, rapamycin, colchicine, D penicillamine, tacrolimus, mizoribine or intravenous immunoglobulin	Within 4 weeks prior to the screening visit[a,]
Etanercept	Within 2 weeks prior to the screening visit[a,]
Anakinra	Within 1 week prior to the screening visit[a]
Nitrates or NO donors (such as amyl nitrate) in any form, including topical; phosphodiesterase (PDE) 5 (PDE5) inhibitors (such as sildenafil, tadalafil, vardenafil); and nonspecific PDE5 inhibitors (theophylline, dipyridamole)	Concomitant therapy with study drug
Prostacyclin analogs (oral beraprost for digital ulcers / Raynaud's disease and short-term / intermittent therapy of up to 21 days with intravenous prostacyclin analogs for digital / vascular lesions is allowed)	Concomitant therapy with study drug

a Concomitant use of these treatments with study medication is also prohibited, except for certain agents from Week 26 onwards; please see "Rescue Therapy" below.

Rescue therapy

[…] The decision to initiate rescue therapy is based on investigator discretion in eligible patients. Rescue therapy may include any of the following 4 agents: methotrexate, mycophenolate mofetil, cyclophosphamide, or azathioprine. These treatments are not provided by the Sponsor.

New text:

[…] These treatments will also be allowed as new-onset during the study at the discretion of the investigator to treat SSc-specific adverse events (eg, Raynaud's phenomena, joint inflammation, new onset renal crisis).

During the long-term extension phase (ie, after completion of all efficacy- and safety-related procedures at Visit 12), except for the contraindicated nitrates or NO donors and PDE5 inhibitors, the addition of any other concomitant medication is at the discretion of the investigator.

[….]

Table 6-2 Prohibited prior and concomitant therapy - amended

Therapy	Timeframe
Chlorambucil, bone marrow transplantation, or total lymphoid irradiation	Any previous therapy[a, b]
Rituximab or other anti-CD20 antibodies	Within 6 months prior to the screening visit[a, b]
Infliximab, certolizumab, golimumab, adalimumab, abatacept, leflunomide or tocilizumab	Within 8 weeks prior to the screening visit[a, b]
Methotrexate, cyclophosphamide, hydroxychloroquine, cyclosporine A, azathioprine, mycophenolate mofetil, rapamycin, colchicine, D penicillamine, tacrolimus, mizoribine or intravenous immunoglobulin	Within 4 weeks prior to the screening visit[a, b]
Etanercept	Within 2 weeks prior to the screening visit[a,b]
Anakinra	Within 1 week prior to the screening visit[a,b]
Nitrates or NO donors (such as amyl nitrate) in any form, including topical; phosphodiesterase (PDE) 5 (PDE5) inhibitors (such as sildenafil, tadalafil, vardenafil); and nonspecific PDE5 inhibitors (theophylline, dipyridamole)	Concomitant therapy with study drug
Prostacyclin analogs (oral beraprost for digital ulcers / Raynaud's disease and short-term / intermittent therapy of up to 21 days with intravenous prostacyclin analogs for digital / vascular lesions is allowed)	Concomitant therapy with study drug

a Concomitant use of these treatments with study medication is also prohibited, except for certain agents from Week 26 onwards; please see "Rescue Therapy" below.
b Concomitant use of these medications will be allowed during the long-term extension phase

Rescue therapy

[…] The decision to initiate rescue therapy is based on investigator discretion in eligible patients. Rescue therapy may include any of the following 4 agents: methotrexate, mycophenolate mofetil, cyclophosphamide, or azathioprine.

- Worsening of inflammatory joint disease or myositis.

Rescue therapy can be initiated at investigator's discretion in eligible patients. In addition to the 4 agents methotrexate, mycophenolate mofetil, cyclophosphamide, or azathioprine, hydroxychloroquine will be allowed for the treatment of inflammatory joint disease and myositis.

These treatments are not provided by the Sponsor.

7.1.1 Tabulated overview - amended

The visit window for Visit 18 was changed from ±14 days to ±2 days in the schedule of events (Table 7-1). The visit windows for all following visits (Vn) remains unchanged.

7.1.2.10 Premature discontinuation of study drug treatment - amended

Old text:

If the patient discontinues study drug prematurely during the main treatment phase (Week 0 – Week 52) he/she will be invited to come for limited assessments (mRSS, pulmonary function test [FVC and DL_{CO} including hemoglobin measurement at the local laboratory] , ~~and~~ PROs) at Week 12, Week 26, Week 39, and Week 52, depending on the timing of discontinuation.

New text:

If the patient discontinues study drug prematurely during the main treatment phase (Week 0 – Week 52) he/she will be invited to come for limited assessments (mRSS, pulmonary function test [FVC and DL_{CO} including hemoglobin measurement at the local laboratory], PROs, and patient's and physician's global assessment) at Week 12, Week 26, Week 39, and Week 52, depending on the timing of discontinuation.

7.3 Efficacy - amended

Old text:

Key secondary endpoint:

- CRISS, consisting of five variables: mRSS, FVC % predicted, physician and patient global assessments, and HAQ-DI score (from SHAQ patient reported outcome)

New text:

Key secondary endpoint:

- CRISS at Week 52, consisting of five variables: mRSS, FVC % predicted, physician and patient global assessments, and HAQ-DI score (from SHAQ patient reported outcome)

7.4 Pharmacokinetics / pharmacodynamics

Old text:

At these study visits the patients should present themselves to the hospital without taking the morning dose of study medication. The trough PK sample should always be obtained at up to 1 hour before administration of the morning dose of study medication.

It is essential that the exact time of blood sampling and time of dose administration of the 3 preceding doses of study medication are documented accurately in the eCRF.

For investigation of exposure behavior, plasma concentrations of BAY63-2521 and its metabolite (M1) will be determined at the times given below using a sparse sampling approach in all participating patients.

[…]

Plasma concentration time points will be compared to the available plasma concentration profiles in healthy volunteers and selected patient populations.

[…]

Pharmacokinetic (PK) samples will be analyzed under the responsibility of the Bayer HealthCare Bioanalytics Laboratory, Bayer Pharma AG, GDD-GED-DMPK Bioanalytics, 42096 Wuppertal, Germany.

New text:

At these study visits the patients should present themselves to the hospital without taking the morning dose of study medication. The trough PK sample should always be obtained at up to 1 hour before administration of the morning dose of study medication and approximately 5 to 15 hours after the evening dose.

It is essential that the exact time of blood sampling and time of dose administration of the 3 preceding doses of study medication are documented accurately in the eCRF. For samples taken before riociguat administration, also the time of the first dose taken at the visit needs to be documented.

For investigation of exposure behavior, plasma concentrations of BAY63-2521 and its metabolite (M1) will be determined at the times given below using a sparse sampling approach in all participating patients. If the investigator decides to take additional PK samples, those may be used for PK analysis as well.

[…]

Plasma concentration time points will be compared to the available plasma concentration profiles in healthy volunteers and selected patient populations. Pharmacokinetic and exposure-response analysis might be performed using population approaches (popPK and popPK/PD, eg, by non-linear mixed effect modeling). Analysis and report will be done under separate cover. This evaluation might be started prior to database lock. If this is applicable, appropriate measures will be taken to maintain blinding of the study team, eg, data will be stored separately, and members of the study team will neither have access to the randomization list nor to individual data.

[…]

Pharmacokinetic (PK) samples will be analyzed under the responsibility of the Bayer HealthCare Bioanalytics Laboratory, Bayer Pharma AG, GDD-GED-DMPK Bioanalytics, 42096 Wuppertal, Germany. The bioanalyst will be unblinded and have access to the randomization list.

7.5.1.1 Definitions

Old text:

- Conditions that started or deteriorated after signing of informed consent will be documented as AEs.

New text:

- Conditions that started or deteriorated after signing of informed consent will be documented as AEs. This includes any conditions related to the underlying disease.

7.6.2 Laboratory parameters - amended

Old text:

Patients with isolated AST or ALT >3xULN or bilirubin >2xULN at screening and/or baseline will have additional analysis of coagulation tests, AST, ALT, AP, GGT, total bilirubin and serum albumin until normalization and/ or stabilization at Visit 2 (Day 14), Visit 3 (Day 28), Visit 4 (Day 42), Visit 5 (Day 56), as needed. Worsening of the parameters in the order of 2-fold from baseline values will be considered clinically significant [22] and additional diagnostic procedures may be requested.

Patients entering the trial with eGFR 15-29 mL/min/1.73m^2 will be undergo additional serum creatinine and eGFR calculation until normalization and/ or stabilization at Visit 2 (Day 14), Visit 3 (Day 28), Visit 4 (Day 42), and Visit 5 (Day 56), as needed. A decrease in eGFR equal or greater than 5ml/min/1.73m^2 in these patients (eGFR 15-29 ml/min/1.73m^2) during the study or a reduction of 25% or more in 12 months will be considered clinically significant [23,24].

New text:

Patients with isolated AST or ALT >3xULN or bilirubin >2xULN at screening and/or baseline will have additional analysis of coagulation tests, AST, ALT, AP, GGT, total bilirubin and serum albumin until normalization and/ or stabilization at Visit 2 (Day 14), Visit 3 (Day 28), Visit 4 (Day 42), Visit 5 (Day 56), as needed. Worsening of the parameters in the order of 2-fold from baseline values will be considered clinically significant [22] and additional diagnostic procedures may be requested. See Section 5.2.1.2 for discontinuation criteria related to liver function.

Patients entering the trial with eGFR 15-29 mL/min/1.73m^2 will be undergo additional serum creatinine and eGFR calculation until normalization and/ or stabilization at Visit 2 (Day 14), Visit 3 (Day 28), Visit 4 (Day 42), and Visit 5 (Day 56), as needed. A decrease in eGFR equal or greater than 5ml/min/1.73m^2 in these patients (eGFR 15-29 ml/min/1.73m^2) during the study or a reduction of 25% or more in 12 months will be considered clinically significant [23,24]. See Section 5.2.1.2 for discontinuation criteria related to eGFR values.

7.6.3 Exploratory Biomarkers

Old text:

[Not applicable. New text added in the end of the section.]

New text:

The evaluation of biomarkers might be started prior to database lock; if this is applicable, appropriate measures will be taken to maintain blinding of the study team (see also Section 6.5 and Section 7.4).

The results of the biomarker analysis will be reported separately to the clinical study report.

7.6.15 Patient's and physician's global assessment

Old text:

Frequency: Visit 1 (Day 0), Visit 10 (Week 26) and Visit 12 (Week 52) ~~and~~ termination visit (not needed after V12).

New text:

Frequency: Visit 1 (Day 0), Visit 10 (Week 26) and Visit 12 (Week 52), termination visit (not needed after V12), and visits with limited assessments.

8.4.2.2 Secondary and exploratory endpoints

Old text:

American College of Rheumatology Combined Response Index for Systemic Sclerosis

Application of CRISS algorithm in a randomized clinical trial is a 2-step process.

[…]

If the patient is determined to exhibit improvement in Step 1 (ie, not assigned a 0.0), Step 2 involves computing the predicted probability of improving (a score between 0.0 and 1.0, inclusive) for each patient using the equation:

$$\frac{exp[-5.54-0.81*\Delta_{MRSS}+0.21*\Delta_{FVC\%}-0.40*\Delta_{Pt-glob}-0.44*\Delta_{MD-glob}-3.41*\Delta_{HAQ-DI}]}{1+exp[-5.54-0.81*\Delta_{MRSS}+0.21*\Delta_{FVC\%}-0.40*\Delta_{Pt-glob}-0.44*\Delta_{MD-glob}-3.41*\Delta_{HAQ-DI}]}$$

where Δ_{MRSS} indicates the change in mRSS from baseline to ~~follow-up~~, Δ_{FVC} denotes the change in FVC % predicted from baseline to ~~follow-up~~, $\Delta_{Pt-glob}$ indicates the change in patient global assessment, $\Delta_{MD-glob}$ denotes the change in physician global assessment, and Δ_{HAQ-DI} is the change in HAQ-DI.

[…]

In each scenario, a decrease of mRSS or HAQ-DI from baseline to ~~follow-up~~ and an increase in FVC % predicted corresponds to very high probabilities of improving.

New text:

American College of Rheumatology Combined Response Index for Systemic Sclerosis

The key secondary endpoint will be the CRISS at Week 52.

Application of CRISS algorithm in a randomized clinical trial is a 2-step process.

[…]

If the patient is determined to exhibit improvement in Step 1 (ie, not assigned a 0.0), Step 2 involves computing the predicted probability of improving (a score between 0.0 and 1.0, inclusive) for each patient using the equation:

$$\frac{exp[-5.54-0.81*\Delta_{MRSS}+0.21*\Delta_{FVC\%}-0.40*\Delta_{Pt-glob}-0.44*\Delta_{MD-glob}-3.41*\Delta_{HAQ-DI}]}{1+exp[-5.54-0.81*\Delta_{MRSS}+0.21*\Delta_{FVC\%}-0.40*\Delta_{Pt-glob}-0.44*\Delta_{MD-glob}-3.41*\Delta_{HAQ-DI}]}$$

where Δ_{MRSS} indicates the change in mRSS from baseline to Week 52, Δ_{FVC} denotes the change in FVC % predicted from baseline to Week 52, $\Delta_{Pt-glob}$ indicates the change in patient global assessment, $\Delta_{MD-glob}$ denotes the change in physician global assessment, and Δ_{HAQ-DI} is the change in HAQ-DI.

[…]

In each scenario, a decrease of mRSS or HAQ-DI from baseline to Week 52 and an increase in FVC % predicted corresponds to very high probabilities of improving.

8.5 Planned interim analyses

Old text:

The main treatment phase will be unblinded for data analysis when the last patient completes the ~~dose-titration period of the long-term extension phase~~ and the database for the main treatment phase is declared clean, whichever occurs last.

The first safety update will be performed on the long-term extension phase when the last patient completes ~~the dose-titration period after the first 10 weeks of this phase~~.

New text:

The main treatment phase will be unblinded for data analysis when the last patient completes the main treatment phase (Visit 12) and the database for the main treatment phase is declared clean, whichever occurs last.

The first safety update will be performed on the long-term extension phase when the last patient completes 24 weeks of treatment (Visit 19).

8.6 Determination of sample size

Old text:

The primary efficacy estimand in this study will be change in mRSS from baseline to Week 52. Assuming a standard deviation of 8 points, a power ~~of~~ 80% and a two-sided significance level of 5%, with a 1:1 randomization, then to detect a placebo-adjusted difference of 4 points, 64 patients in each treatment group, a total of 128 patients, would be required to be valid for the ITT analysis. Allowing for up to two patients randomized and not treated, ~~a total of~~ 130 randomized patients are planned.

~~For the secondary efficacy measure based on the mRSS progression rate, if the sample size is fixed at 128 patients with a 1:1 randomization, then with an event rate on placebo of 20% and on riociguat of 4% (a relative risk reduction of 80%), the study has a power of 71% to detect this as statistically significant at a two-sided p ≤ 0.05.~~

Sources of these sample size and power calculations are nQuery version 7.0, module~~s~~ MTT0-1 ~~and PTT2-1~~.

New text:

The primary efficacy estimand in this study will be change in mRSS from baseline to Week 52. Assuming a standard deviation of 8 points, a power between 70% and 80% and a two-sided significance level of 5% with a 1:1 randomization, then to detect a placebo-adjusted difference of 4 points, between 51 and 64 patients in each treatment group, a total of 102 to 128 patients, would be required to be valid for the ITT analysis (see Table 8–2). Allowing for up to two patients randomized and not treated, approximately 130 randomized patients are planned.

Table 8-2 Power calculations: power varying from 70% to 80% in 2% stages

N per group	51	53	56	58	61	64
Total N	102	106	112	116	122	128
Power (%)	70	72	74	76	78	80

N = number of patients

Sources of these sample size and power calculations are nQuery version 7.0, module MTT0-1.

9.3 Data processing

Old text:

For data coding (eg, AEs, medication), internationally recognized and accepted dictionaries will be used. After its initial release for biometrical analysis, the clinical database is planned to be re-opened for the inclusion of the following additional data: eg, PK data, ~~antibody~~ data.

New text:

For data coding (eg, AEs, medication), internationally recognized and accepted dictionaries will be used. After its initial release for biometrical analysis, the clinical database is planned to be re-opened for the inclusion of the following additional data: eg, PK data, biomarker data.

13.4 Amendment 6

Amendment 6 was approved on 02 MAY 2017.

13.4.1 Overview of changes to the study

This amendment was implemented based on the recommendation of the data monitoring committee (DMC) after review of the safety analysis on 01 MAR 2017. Due to increased complaints of both palpitations and dizziness in subjects with interstitial lung disease (ILD) noted by the DMC, the assessment of orthostatic changes in blood pressure and heart rate as well as the measurement of oxygen saturation (using forehead pulse oximetry) were added to the protocol assessments for all subjects. As recruitment for this study was already completed, the new assessments will not be implemented starting with the screening visit, but only for study visits, which will be performed after implementation of this amendment.

Additionally, an error in the description of the units provided in exclusion criterion 6 was corrected.

Correction of typographical errors and minor editorial changes are not specified in the list of changes below. New text is displayed as underlined; text that was deleted is presented as strike-through text.

13.4.2 Changes to the protocol text

4.1.2 Main treatment phase (Week 0 to Week 52) – amended

Old text:

Dose titration algorithm

The study medication dose for the next titration step will be determined every 2 weeks according to the patient's well-being and the peripheral systolic blood pressure (SBP) measured at trough before intake of the morning according to the following algorithm (dose titration scheme):

- If SBP is ≥ 95 mmHg and the patient has no signs or symptoms of hypotension, the dosage should be increased by 0.5 mg TID.
- If SBP is < 95 mmHg, the dosage should be maintained provided the patient does not show any signs or symptoms of hypotension.
- If SBP is < 95 mmHg, and the patient exhibits signs or symptoms of hypotension, the current dosage should be decreased by 0.5 mg TID.

New text:

Dose titration algorithm

The study medication dose for the next titration step will be determined every 2 weeks according to the patient's well-being and the peripheral systolic blood pressure (SBP) measured at trough before intake of the morning dose as well as based on any of the 2 measurements (supine or sitting) performed 2 hours after dose intake to assess orthostatic changes according to the following algorithm (dose titration scheme):

- If SBP is ≥ 95 mmHg and the patient has no signs or symptoms of hypotension, the dosage should be increased by 0.5 mg TID.
- If SBP is < 95 mmHg, the dosage should be maintained provided the patient does not show any signs or symptoms of hypotension.
- If SBP is < 95 mmHg, and the patient exhibits signs or symptoms of hypotension, the current dosage should be decreased by 0.5 mg TID.

5.1.2 Exclusion Criteria - amended

Old text:

6. Laboratory examinations

- Patients with: hemoglobin <9.0 g/dL, white blood cell (WBC) count <3000/mm^3 (<3 × 10^9/L), platelet count <100,000/mm^3 (<~~3~~ × 10^9/L)

New text:

6. Laboratory examinations

- Patients with: hemoglobin <9.0 g/dL, white blood cell (WBC) count <3000/mm^3 (<3 × 10^9/L), platelet count <100,000/mm^3 (<100 × 10^9/L)

7.1.1 Tabulated overview - amended

Old text:

Table 7–1 Schedule of evaluations - amended

	Scr	Main Treatment Phase (Double-blind) [v]												Long-term Extension Phase							
	Scr Phase	Dose-Titration Period						Maintenance Period						Dose-Titration Period					Open-label Extension Period	Safety Follow-up	
**Visit**	**V0**	**V1**	**V2**	**V3**	**V4**	**V5**	**V6**	**V7**	**V8**	**V9**	**V10**	**V11**	**V12[a]**	**V13**	**V14**	**V15**	**V16**	**V17**	**V18 – Vn (q 12 wk)**	**TV[b]**	**SFU**
**Week**	-2	0	2	4	6	8	10	12	14	20	26	39	52	54	56	58	60	62	64 – n		
**Day**	**-14 to -1**	**0**	**14 ± 2**	**28 ± 2**	**42 ± 2**	**56 ± 2**	**70 ± 2**	**84 ± 2**	**98 ± 2**	**140 ± 2**	**182 ± 2**	**273 ± 14**	**364 -14 to +2**	**378 ± 2**	**392 ± 2**	**406 ± 2**	**420 ± 2**	**434 ± 2**	**448 – (n + 84) V18 ± 2 Vn ± 14**		**30 + 5**
Written informed consent	X																				
Inclusion / exclusion criteria	X	X																			
Demographic data	X																				
Medical and surgical history	X	X																			
Other history (alcohol)	X																				
Smoking history	X	X									X		X						X	X	
Height and weight[c]	X	X						X			X		X						X	X	X
Physical examination	X	X						X			X		X						X	X	X
mRSS	X							X			X	X	X						X	X	
Assessment of Raynaud's attacks	X[d]	X[e]						X[f]	X[e]												
Digital ulcer net burden assessment	X	X	X	X	X	X	X	X	X	X	X	X	X	X	X	X	X	X	X	X	
Tender & swollen joint count	X												X							X[g]	

Table 7–1 Schedule of evaluations - amended

	Scr	Main Treatment Phase (Double-blind) [v]												Long-term Extension Phase							
	Scr Phase	Dose-Titration Period						Maintenance Period						Dose-Titration Period					Open-label Extension Period	Safety Follow-up	
**Visit**	**V0**	**V1**	**V2**	**V3**	**V4**	**V5**	**V6**	**V7**	**V8**	**V9**	**V10**	**V11**	**V12[a]**	**V13**	**V14**	**V15**	**V16**	**V17**	**V18 – Vn (q 12 wk)**	**TV[b]**	**SFU**
**Week**	-2	0	2	4	6	8	10	12	14	20	26	39	52	54	56	58	60	62	64 – n		
**Day**	**-14 to -1**	**0**	**14 ± 2**	**28 ± 2**	**42 ± 2**	**56 ± 2**	**70 ± 2**	**84 ± 2**	**98 ± 2**	**140 ± 2**	**182 ± 2**	**273 ± 14**	**364 -14 to +2**	**378 ± 2**	**392 ± 2**	**406 ± 2**	**420 ± 2**	**434 ± 2**	**448 – (n + 84) V18 ± 2 Vn ± 14**		**30 + 5**
Vital signs (BP, HR)[h]	X	X	X	X	X	X	X	X	X	X	X	X	X	X	X	X	X	X	X	X	X
Vital signs post-dose (BP, HR)[i,j]		X[i]										X[j]	X[i]								
Blood sample for safety[k, u]	X	X	X[u]	X[u]	X[u]	X[u]	X				X	X	X					X	X	X	X
Blood sample for PK		X[l]	X[m]	X[m]	X[m]	X[m]	X[m]					X[n]	X[l]	X[m]	X[m]	X[m]	X[m]	X[m]			
Blood sample for biomarkers		X							X											X[r]	
Blood sample for Autoantibody screen	X																				
Pregnancy test (if applicable)[o]	X	X		X		X		X		X			X		X		X		X	X	X
12-lead ECG[p]	X	X						X			X	X	X						X	X	X
Pulmonary function tests[q]	X							X			X	X	X						X	X	
Skin biopsy		X							X											X[r]	
Questionnaires[s]		X							X		X	X	X						X	X	
Patient's and physician's global assessment		X									X		X							X[g]	
Patient interference with skin		X									X		X							X[g]	
Tendon Friction Rubs[t]		X							X												

Table 7–1 Schedule of evaluations - amended

	Scr	Main Treatment Phase (Double-blind) [v]												Long-term Extension Phase							
	Scr Phase	Dose-Titration Period						Maintenance Period						Dose-Titration Period					Open-label Extension Period	Safety Follow-up	
**Visit**	**V0**	**V1**	**V2**	**V3**	**V4**	**V5**	**V6**	**V7**	**V8**	**V9**	**V10**	**V11**	**V12[a]**	**V13**	**V14**	**V15**	**V16**	**V17**	**V18 – Vn (q 12 wk)**	**TV[b]**	**SFU**
**Week**	-2	0	2	4	6	8	10	12	14	20	26	39	52	54	56	58	60	62	64 – n		
**Day**	**-14 to -1**	**0**	**14 ± 2**	**28 ± 2**	**42 ± 2**	**56 ± 2**	**70 ± 2**	**84 ± 2**	**98 ± 2**	**140 ± 2**	**182 ± 2**	**273 ± 14**	**364 -14 to +2**	**378 ± 2**	**392 ± 2**	**406 ± 2**	**420 ± 2**	**434 ± 2**	**448 – (n + 84) V18 ± 2 Vn ± 14**		**30 + 5**
Randomization (IxRS)		X																			
Dispensation of study drug		X	X	X	X	X	X	X	X	X	X	X	X	X	X	X	X	X	X		
Drug accountability			X	X	X	X	X	X	X	X	X	X	X	X	X	X	X	X	X	X	
Adverse events recording	X	X	X	X	X	X	X	X	X	X	X	X	X	X	X	X	X	X	X	X	X
Prior and concomitant therapy	X	X	X	X	X	X	X	X	X	X	X	X	X	X	X	X	X	X	X	X	X
Survival status																					X

Abbreviations: BP = blood pressure; ECG = electrocardiogram; TV = Termination Visit; SFU = 30-Day Safety Follow-up (30 [+5] days after the last dose of study drug); HR = heart rate; IxRS = telephone-based or web-based response system; mRSS = modified Rodnan skin score; PK = pharmacokinetics; q 12 wk = every 12 weeks; Scr = Screening.
Note: Day 0 (Visit 1) is the baseline visit.

a Visit 12 (Week 52) is the last visit in the Maintenance Period of the Main Treatment phase and the first (baseline) visit in the Long-term Extension phase.
b To be performed as soon as possible after discontinuation of study medication.
c Height to be measured only at the screening visit
d Patient to be provided with 7-day diary, to complete daily for 7 consecutive days leading up to Visit 1
e Patient and physician assessment of Raynaud's disease (see Section 7.6.12)
f Patient to be provided with 7-day diary, to complete daily for 7 consecutive days leading up to Visit 8.
g Must not be performed if TV is after V12.
h Blood pressure and heart rate will be measured at all visits with the patient in a sitting position, after the patient has been at rest for at least 5 minutes. The same arm is always used for these measurements (see Section 7.6.5).
i Vital signs to be measured within 20 minutes prior to each post-dose PK sampling (see Table 7–2)
j Vital signs to be measured within 20 minutes prior to PK sampling (see Table 7–2)
k Coagulation tests to be performed at a local laboratory
l Samples for PK to be taken 1 – 2 hours and 3 – 4 hours after the morning dose of study medication (see Table 7–2)

m Sample for PK to be taken up to 1 hour before the morning dose of study medication (trough) (see Table 7–3)
n Samples for PK to be taken up to 1 hour before the morning dose of study medication (trough) and 2 – 3 hours after the morning dose of study medication (peak) (see Table 7–4)
o For women of childbearing potential only. Serum pregnancy test is required only at screening; pregnancy testing (urine) will be done every 4 weeks and should continue until 30 days after the last dose of study drug. When pregnancy testing coincides with a visit, the test should be performed at the site, but otherwise should be performed at home.
p Standard ECG (12-lead ECG) will be recorded with the patient in the supine position, after the patient has been at rest for at least 5 minutes (see Section 7.6.7).
q Pulmonary function tests include forced vital capacity (FVC) and diffusion capacity of the lung for carbon monoxide (DL_{CO}).
r Must be done at a TV if this visit occurs before Visit 8.
s Questionnaires include Scleroderma Health Assessment Questionnaire (SHAQ), Short Form 36 (SF-36), Patient-Reported Outcomes Measurement Information System (PROMIS)-29, and University of California, Los Angeles, Scleroderma Clinical Trial Consortium Gastrointestinal Scale (UCLA SCTC GIT) 2.0.
t Assessment of the presence or absence of tendon friction rubs
u Additional monitoring may apply for patients with impaired renal and liver function at Visit 2, 3, 4, 5 as needed (see Section 7.6.2)[149]

v All patients who discontinue study drug prematurely during the main treatment phase (Week 0 – Week 52) will be invited to come for limited assessments (see Sections 7.1.2.10 and 7.6.2).[150]

[149] Per Amendment 1
[150] Per Amendment 4

New text:

Table 7–1 Schedule of evaluations - amended

	Scr	Main Treatment Phase (Double-blind) [v]												Long-term Extension Phase							
	Scr Phase	Dose-Titration Period						Maintenance Period						Dose-Titration Period					Open-label Extension Period	Safety Follow-up	
**Visit**	**V0**	**V1**	**V2**	**V3**	**V4**	**V5**	**V6**	**V7**	**V8**	**V9**	**V10**	**V11**	**V12[a]**	**V13**	**V14**	**V15**	**V16**	**V17**	**V18 – Vn (q 12 wk)**	**TV[b]**	**SFU**
**Week**	-2	0	2	4	6	8	10	12	14	20	26	39	52	54	56	58	60	62	64 – n		
**Day**	**-14 to -1**	**0**	**14 ± 2**	**28 ± 2**	**42 ± 2**	**56 ± 2**	**70 ± 2**	**84 ± 2**	**98 ± 2**	**140 ± 2**	**182 ± 2**	**273 ± 14**	**364 -14 to +2**	**378 ± 2**	**392 ± 2**	**406 ± 2**	**420 ± 2**	**434 ± 2**	**448 – (n + 84) V18 ± 2 Vn ± 14**		**30 + 5**
Written informed consent	X																				
Inclusion / exclusion criteria	X	X																			
Demographic data	X																				
Medical and surgical history	X	X																			
Other history (alcohol)	X																				
Smoking history	X	X									X		X						X	X	
Height and weight[c]	X	X						X			X		X						X	X	X
Physical examination	X	X						X			X		X						X	X	X
mRSS	X							X			X	X	X						X	X	
Assessment of Raynaud's attacks	X[d]	X[e]						X[f]	X[e]												
Digital ulcer net burden assessment	X	X	X	X	X	X	X	X	X	X	X	X	X	X	X	X	X	X	X	X	
Tender & swollen joint count	X												X							X[g]	
Oximetry[w]										X	X	X	X	X	X	X	X	X	X	X	X

Table 7–1 Schedule of evaluations - amended

	Scr	Main Treatment Phase (Double-blind) [v]												Long-term Extension Phase							
	Scr Phase	Dose-Titration Period						Maintenance Period						Dose-Titration Period					Open-label Extension Period	Safety Follow-up	
**Visit**	**V0**	**V1**	**V2**	**V3**	**V4**	**V5**	**V6**	**V7**	**V8**	**V9**	**V10**	**V11**	**V12[a]**	**V13**	**V14**	**V15**	**V16**	**V17**	**V18 – Vn (q 12 wk)**	**TV[b]**	**SFU**
**Week**	-2	0	2	4	6	8	10	12	14	20	26	39	52	54	56	58	60	62	64 – n		
**Day**	**-14 to -1**	**0**	**14 ± 2**	**28 ± 2**	**42 ± 2**	**56 ± 2**	**70 ± 2**	**84 ± 2**	**98 ± 2**	**140 ± 2**	**182 ± 2**	**273 ± 14**	**364 -14 to +2**	**378 ± 2**	**392 ± 2**	**406 ± 2**	**420 ± 2**	**434 ± 2**	**448 – (n + 84) V18 ± 2 Vn ± 14**		**30 + 5**
Vital signs (BP, HR)[h]	X	X	X	X	X	X	X	X	X	X	X	X	X	X	X	X	X	X	X	X	X
Vital signs post-dose (BP, HR)[i,j]		X[i]										X[j]	X[i]								
<u>BP and HR for assessment of orthostatic changes[x]</u>												<u>X</u>	<u>X</u>	<u>X</u>	<u>X</u>	<u>X</u>	<u>X</u>	<u>X</u>			
Blood sample for safety[k, u]	X	X	X[u]	X[u]	X[u]	X[u]	X				X	X	X					X	X	X	X
Blood sample for PK		X[l]	X[m]	X[m]	X[m]	X[m]	X[m]					X[n]	X[l]	X[m]	X[m]	X[m]	X[m]	X[m]			
Blood sample for biomarkers		X							X											X[r]	
Blood sample for Autoantibody screen	X																				
Pregnancy test (if applicable)[o]	X	X		X		X		X		X			X		X		X		X	X	X
12-lead ECG[p]	X	X						X			X	X	X						X	X	X
Pulmonary function tests[q]	X							X			X	X	X						X	X	
Skin biopsy		X							X											X[r]	
Questionnaires[s]		X							X		X	X	X						X	X	
Patient's and physician's global assessment		X									X		X							X[g]	
Patient interference with skin		X									X		X							X[g]	

Table 7–1 Schedule of evaluations - amended

	Scr	Main Treatment Phase (Double-blind) [v]												Long-term Extension Phase							
	Scr Phase	Dose-Titration Period						Maintenance Period						Dose-Titration Period					Open-label Extension Period	Safety Follow-up	
**Visit**	**V0**	**V1**	**V2**	**V3**	**V4**	**V5**	**V6**	**V7**	**V8**	**V9**	**V10**	**V11**	**V12[a]**	**V13**	**V14**	**V15**	**V16**	**V17**	**V18 – Vn (q 12 wk)**	**TV[b]**	**SFU**
**Week**	-2	0	2	4	6	8	10	12	14	20	26	39	52	54	56	58	60	62	64 – n		
**Day**	**-14 to -1**	**0**	**14 ± 2**	**28 ± 2**	**42 ± 2**	**56 ± 2**	**70 ± 2**	**84 ± 2**	**98 ± 2**	**140 ± 2**	**182 ± 2**	**273 ± 14**	**364 -14 to +2**	**378 ± 2**	**392 ± 2**	**406 ± 2**	**420 ± 2**	**434 ± 2**	**448 – (n + 84) V18 ± 2 Vn ± 14**		**30 + 5**
Tendon Friction Rubs[t]		X							X												
Randomization (IxRS)		X																			
Dispensation of study drug		X	X	X	X	X	X	X	X	X	X	X	X	X	X	X	X	X	X		
Drug accountability			X	X	X	X	X	X	X	X	X	X	X	X	X	X	X	X	X	X	
Adverse events recording	X	X	X	X	X	X	X	X	X	X	X	X	X	X	X	X	X	X	X	X	X
Prior and concomitant therapy	X	X	X	X	X	X	X	X	X	X	X	X	X	X	X	X	X	X	X	X	X
Survival status																					X

Abbreviations: BP = blood pressure; ECG = electrocardiogram; TV = Termination Visit; SFU = 30-Day Safety Follow-up (30 [+5] days after the last dose of study drug); HR = heart rate; IxRS = telephone-based or web-based response system; mRSS = modified Rodnan skin score; PK = pharmacokinetics; q 12 wk = every 12 weeks; Scr = Screening.
Note: Day 0 (Visit 1) is the baseline visit.

a Visit 12 (Week 52) is the last visit in the Maintenance Period of the Main Treatment phase and the first (baseline) visit in the Long-term Extension phase.
b To be performed as soon as possible after discontinuation of study medication.
c Height to be measured only at the screening visit
d Patient to be provided with 7-day diary, to complete daily for 7 consecutive days leading up to Visit 1
e Patient and physician assessment of Raynaud's disease (see Section 7.6.12)
f Patient to be provided with 7-day diary, to complete daily for 7 consecutive days leading up to Visit 8.
g Must not be performed if TV is after V12.
h Blood pressure and heart rate will be measured at all visits with the patient in a sitting position, after the patient has been at rest for at least 5 minutes. The same arm is always used for these measurements (see Section 7.6.5).
i Vital signs to be measured within 20 minutes prior to each post-dose PK sampling (see Table 7–2)
j Vital signs to be measured within 20 minutes prior to PK sampling (see Table 7–2)

k Coagulation tests to be performed at a local laboratory

l Samples for PK to be taken 1 – 2 hours and 3 – 4 hours after the morning dose of study medication (see Table 7–2)

m Sample for PK to be taken up to 1 hour before the morning dose of study medication (trough) (see Table 7–3)

n Samples for PK to be taken up to 1 hour before the morning dose of study medication (trough) and 2 – 3 hours after the morning dose of study medication (peak) (see Table 7–4)

o For women of childbearing potential only. Serum pregnancy test is required only at screening; pregnancy testing (urine) will be done every 4 weeks and should continue until 30 days after the last dose of study drug. When pregnancy testing coincides with a visit, the test should be performed at the site, but otherwise should be performed at home.

p Standard ECG (12-lead ECG) will be recorded with the patient in the supine position, after the patient has been at rest for at least 5 minutes (see Section 7.6.7).

q Pulmonary function tests include forced vital capacity (FVC) and diffusion capacity of the lung for carbon monoxide (DL_{CO}).

r Must be done at a TV if this visit occurs before Visit 8.

s Questionnaires include Scleroderma Health Assessment Questionnaire (SHAQ), Short Form 36 (SF-36), Patient-Reported Outcomes Measurement Information System (PROMIS)-29, and University of California, Los Angeles, Scleroderma Clinical Trial Consortium Gastrointestinal Scale (UCLA SCTC GIT) 2.0.

t Assessment of the presence or absence of tendon friction rubs

u Additional monitoring may apply for patients with impaired renal and liver function at Visit 2, 3, 4, 5 as needed (see Section 7.6.2)[151]

v All patients who discontinue study drug prematurely during the main treatment phase (Week 0 – Week 52) will be invited to come for limited assessments (see Sections 7.1.2.10 and 7.6.2).[152]

w Oxygen saturation will be measured using forehead pulse oximetry. At visits with blood samples for PK and blood pressure measurements for the assessment of orthostatic changes, post-dose measurements will be performed in addition to pre-dose measurements (see Section 7.6.18, Table 7–2, Table 7–3, Table 7–4)

x Measure supine and sitting blood pressure and heart rate to check for orthostatic changes approximately 2 hours after intake of study medication. (see Section 7.6.5, Table 7–2, Table 7–3, Table 7–4).

---

[151] Per Amendment 1
[152] Per Amendment 4

7.1.1 Tabulated overview - amended

Old text:

Pharmacokinetics sampling flow charts

Profile day (Visit 1 and Visit 12)

- ~~1 –~~ 2 hours **after** administration of the morning dose of study medication
- 3 – 4 hours **after** administration of the morning dose of study medication

Table 7–2: Profile Day PK Sampling (Visit 1 and Visit 12)

Visit 1 and Visit 12 (profile days)			Cycle of procedures			
**Order of procedures**	Starting point of procedures	Time interval (h)	**00**	00	**1 – 2 h**	**3 – 4 h**
	Blood pressure, heart rate		X			
	Administration of morning dose of study medication			X		
	Blood pressure, heart rate (within 20 minutes prior to PK sample)				X	
	Blood sample for PK				X	
	Blood pressure, heart rate (within 20 minutes prior to PK sample)					X
	Blood sample for PK					X

Abbreviations: h = hours; PK = pharmacokinetic.

Visits 2 to 6 and Visits 13 to 17

- Up to 1 hour **before** administration of the morning dose of study medication (trough)

Table 7–3: PK Sampling (Visits 2 – 6 and Visits 13 – 17)

Visit 2 to Visit 6 and Visit 13 to Visit 17			Cycle of procedures		
**Order of procedures**	Starting point of procedures	Time interval (h)	**–1 h – 00**	**00**	00
	Blood sample for PK		X		
	Blood pressure, heart rate			X	
	Administration of morning dose of study medication				X

Abbreviations: h = hours; PK = pharmacokinetic.

Visit 11

- Up to 1 hour **before** administration of morning dose of study medication (trough)
- 2 ~~– 3~~ hours **after** administration of morning dose of study medication (peak)

Table 7–4 PK Sampling (Visit 11)

Visit 11			Cycle of procedures			
**Order of procedures** ↓	Starting point of procedures	Time interval (h)	**–1 h – 00**	**00**	00	**2 – 3 h**
	Blood sample for PK		X			
	Blood pressure, heart rate			X		
	Administration of morning dose of study medication				X	
	~~Blood pressure, heart rate (within 20 minutes prior to PK sampling)~~					~~X~~
	Blood sample for PK					X

Abbreviations: h = hour; PK = pharmacokinetics.

New text:

Pharmacokinetics sampling flow charts

Profile day (Visit 1 and Visit 12)

- 2 hours **after** administration of the morning dose of study medication
- 3 – 4 hours **after** administration of the morning dose of study medication

Table 7–2 Profile Day PK Sampling (Visit 1 and Visit 12)[153]

Visit 1 and Visit 12 (profile days)			Cycle of procedures				
**Order of procedures** ↓	Starting point of procedures	Time interval (h)	**00**	00	**1:45 – 2 h**	**2h**	**3 – 4 h**
	At Visit 12 only: oxygen saturation using forehead pulse oximetry		X				
	Blood pressure, heart rate		X				
	Administration of morning dose of study medication			X			
	At Visit 12 only: oxygen saturation using forehead pulse oximetry				X[a]		
	Blood pressure, heart rate; at Visit 12 only: supine and sitting position				X[a]		
	Blood sample for PK					X	
	Blood pressure, heart rate (within 20 minutes prior to PK sample)						X
	Blood sample for PK						X

a At Visit 12 only: measure oxygen saturation, blood pressure and heart rate in supine and sitting position (see Section 7.6.5 and Section 7.6.18).
Abbreviations: h = hours; PK = pharmacokinetic.

Visits 2 to 6 and Visits 13 to 17

- Up to 1 hour **before** administration of the morning dose of study medication (trough)

[153] Per Amendment 6

Table 7-3 PK Sampling (Visits 2 – 6 and Visits 13 – 17)

Visit 2 to Visit 6 and Visit 13 to Visit 17			Cycle of procedures			
**Order of procedures**	Starting point of procedures	Time interval (h)	**–1 h – 00**	**00**	00	2h
	Blood sample for PK		X			
	Visit 13-17 only: oxygen saturation using forehead pulse oximetry			X		
	Blood pressure, heart rate			X		
	Administration of morning dose of study medication				X	
	Visit 13-17 only: oxygen saturation using forehead pulse oximetry					X
	Visits 13-17 only: Blood pressure, heart rate in supine and sitting position					X

Abbreviations: h = hours; PK = pharmacokinetic.

Visit 11

- Up to 1 hour **before** administration of morning dose of study medication (trough)
- 2 hours **after** administration of morning dose of study medication (peak)

Table 7–4 PK Sampling (Visit 11)[154]

Visit 11			Cycle of procedures				
**Order of procedures**	Starting point of procedures	Time interval (h)	**–1 h – 00**	**00**	00	**1:45-2 h**	**2 h**
	Blood sample for PK		X				
	Oxygen saturation using forehead pulse oximetry			X			
	Blood pressure, heart rate			X			
	Administration of morning dose of study medication				X		
	Oxygen saturation using forehead pulse oximetry					X[a]	
	Blood pressure, heart rate in supine and sitting position					X[a]	
	Blood sample for PK						X

a Measure oxygen saturation, blood pressure and heart rate in supine and sitting position (see Section 7.6.5 and Section 7.6.18).
Abbreviations: h = hour; PK = pharmacokinetics.

[154] Per Amendment 6

7.1.2.4 Visits 7 through to 11 – Main treatment phase - Maintenance period

Old text:

[…]

- Vital signs (blood pressure and heart rate). Blood pressure and heart rate will be measured at all visits with the patient in a sitting position, after the patient has been at rest for at least 5 minutes. The same arm is always used for these measurements (See Section 7.6.5)

[…]

New text:

[…]

- Oxygen saturation measurement using forehead pulse oximetry at Visits 9 and 10 only. At Visit 11 only, oxygen saturation will be measured before and after intake of study drug (see Section 7.6.18, Table 7–2, Table 7–3, and Table 7–4)
- Vital signs (blood pressure and heart rate). Blood pressure and heart rate will be measured at all visits with the patient in a sitting position, after the patient has been at rest for at least 5 minutes. The same arm is always used for these measurements (See Section 7.6.5)
- Assessment of orthostatic changes: measure blood pressure and heart rate in supine position and sitting position approximately 2 h after intake of study drug (Visit 11 only, see Section 7.6.5).

[…]

7.1.2.5 Visit 12 (Week 52)

Old text:

[…]

- Vital signs (blood pressure and heart rate). Blood pressure and heart rate will be measured at all visits with the patient in a sitting position, after the patient has been at rest for at least 5 minutes. The same arm is always used for these measurements (See Section 7.6.5)

[…]

New text:

[…]

- Oxygen saturation will be measured before and after intake of study drug (see Section 7.6.18, Table 7–2, Table 7–3, and Table 7–4)
- Vital signs (blood pressure and heart rate). Blood pressure and heart rate will be measured at all visits with the patient in a sitting position, after the patient has been at rest for at least 5 minutes. The same arm is always used for these measurements (See Section 7.6.5)
- Assessment of orthostatic changes: measure blood pressure and heart rate in supine position and sitting position approximately 2 h after intake of study drug (see Section 7.6.5).

[…]

7.1.2.6 Visits 13 through to 17 – Long-term extension phase - Dose-titration period

Old text:

[…]

- Vital signs (blood pressure and heart rate). Blood pressure and heart rate will be measured at all visits with the patient in a sitting position, after the patient has been at rest for at least 5 minutes. The same arm is always used for these measurements (See Section 7.6.5)

[…]

New text:

[…]

- Oxygen saturation will be measured before and after intake of study drug (see Section 7.6.18, Table 7–2, Table 7–3, and Table 7–4)
- Vital signs (blood pressure and heart rate). Blood pressure and heart rate will be measured at all visits with the patient in a sitting position, after the patient has been at rest for at least 5 minutes. The same arm is always used for these measurements (See Section 7.6.5)
- Assessment of orthostatic changes: measure blood pressure and heart rate in supine position and sitting position approximately 2 h after intake of study drug (see Section 7.6.5).

[…]

7.1.2.7 Visits 18 through Visit n (every 12 weeks) – Long-term extension phase – Open-label extension period

7.1.2.8 Termination Visit

7.1.2.9 Safety follow-up visit

The following change applies to all sections mentioned above.

Old text:

[…]

- Vital signs (blood pressure and heart rate). Blood pressure and heart rate will be measured at all visits with the patient in a sitting position, after the patient has been at rest for at least 5 minutes. The same arm is always used for these measurements (See Section 7.6.5)

[…]

New text:

[…]

- Oxygen saturation measurement using forehead pulse oximetry (see Section 7.6.18).
- Vital signs (blood pressure and heart rate). Blood pressure and heart rate will be measured at all visits with the patient in a sitting position, after the patient has been at rest for at least 5 minutes. The same arm is always used for these measurements (See Section 7.6.5)

[…]

7.4 Pharmacokinetics / pharmacodynamics – amended

Old text:

Profile day (Visit 1 and Visit 12)

- ~~1 –~~ 2 hours **after** administration of the morning dose of study medication
- 3 – 4 hours **after** administration of the morning dose of study medication

Visits 2 through to 6 and Visits 13 through to 17

- Up to 1 hour **before** administration of morning dose of study medication (trough)

Visit 11

- Up to 1 hour **before** administration of morning dose of study medication (trough)
- 2 ~~– 3~~ hours **after** administration of morning dose of study medication (peak)

New text:

Profile day (Visit 1 and Visit 12)

- 2 hours **after** administration of the morning dose of study medication

- 3 – 4 hours **after** administration of the morning dose of study medication

Visits 2 through to 6 and Visits 13 through to 17

- Up to 1 hour **before** administration of morning dose of study medication (trough)

Visit 11

- Up to 1 hour **before** administration of morning dose of study medication (trough)
- 2 hours **after** administration of morning dose of study medication (peak)

7.6.5 Blood pressure and heart rate measurement

New text was added in the end of the section.

New text:

Assessment of orthostatic changes

To assess orthostatic changes, blood pressure and heart rate will be measured with the patient in a supine position, after the patient has rested for 5 minutes. Immediately thereafter, the patient should sit up and blood pressure and heart rate will be measured after the patient has rested for 2 minutes.

These measurements should be performed approximately 2 hours after study medication intake.

Frequency: Visit 11 (Week 39), Visit 12 (Week 52), during the dose-titration period of the long-term extension phase (Visits 13 to 17).

7.6.18 Oxygen saturation measurement using forehead pulse oximetry

Section was newly inserted with Amendment 6.

New text:

Oxygen saturation measurements will be performed in all subjects using non-invasive forehead pulse oximetry expressing the SpO_2 (peripheral oxygen saturation) percentage. At visits with blood pressure measurements to assess orthostatic changes, oxygen saturation will be measured pre-dose and post-dose (see Table 7–2, Table 7–3, and Table 7–4). At all other visits, oxygen saturation will be measured pre-dose only.

If the patient receives supplemental oxygen, the amount [L/min] will be recorded in the eCRF.

Frequency: Visit 9 (Week 20), Visit 10 (Week 26), Visit 11 (Week 39), Visit 12 (Week 52), Visits 13 to 17 (Week 54 to 62), Visit 18 to Visit n (Open-label Extension Period), Termination Visit, and 30-day Safety Follow-up.

8.4.3 Safety

Old text:

[…]

Descriptive analysis of vital and their changes from baseline will be performed by treatment group and visit.

New text:

[…]

Descriptive analysis of vital signs and their changes from baseline will be performed by treatment group and visit. Descriptive statistics of pulse oximetry and blood pressure/heart rate measurements for the assessment of orthostatic changes will be performed by treatment group and visit.

13.5 Amendment 7

Amendment 7 is presented using a different approach to the previous amendments of this protocol. The rationale for changes in this amendment and all affected sections are provided in the "Protocol Amendment Summary of Changes Table" at the beginning of this document directly before the Table of Contents. The old/new text section detailing the changes compared to the last global CSP version has been replaced with a document edited in track change mode showing all changes, this document is available on request.

14. Appendices

14.1 Child-Pugh Classification of Liver Disease

Factor	+1	+2	+3
**Bilirubin (mg/dL)**	< 2	2 – 3	> 3
**Albumin (g/dL)**	> 3.5	2.8 – 3.5	< 2.8
**International Normalized Ratio**	< 1.7	1.7 – 2.3	> 2.3
**Ascites**	None	Mild	Moderate / Severe
**Encephalopathy**	None	Grade I – II or medically controlled	Grade III – IV or poorly controlled

Child-Pugh Class	A	B	C
**Points**	5 – 6	7 – 9	10 – 15

Source: Pugh RN, Murray-Lyon IM, Dawson JL, Pietroni MC, Williams R. Transection of the oesophagus for bleeding oesophageal varices. Br J Surg 1973;60:646-649.[38]

14.2 Definition of Systemic Sclerosis Renal Crisis - amended

Scleroderma renal crisis is defined as the new onset of severe hypertension associated with a rapid increase in serum creatinine concentration, microangiopathic hemolytic anemia, or both [39][155]. Systemic sclerosis renal crisis usually manifests with acute onset of moderate-to-severe 'accelerated' hypertension and oliguric renal failure [40].

The definition of systemic sclerosis hypertensive renal crisis is independent of whether or not concomitant antihypertensive medications are used.

History of new-onset hypertension, based on any of the following (measurements repeated and confirmed at least 2 hours apart within 3 days of first event-associated observation, with a change from baseline*). As a reminder, renal crisis is defined as meeting the first 4 signs:

1. Systolic blood pressure (SBP) ≥ 140 mmHg
2. Diastolic blood pressure (DBP) ≥ 90 mmHg
3. Rise in SBP ≥ 30 mmHg compared to baseline
4. Rise in DBP ≥ 20 mmHg compared to baseline

**Historical baseline blood pressure of the patient*

AND

At least 1 of the following 5 features:

1. Serum Creatinine: increase of ≥ 50% above baseline*
2. Proteinuria: ≥ 2+ by dipstick confirmed by protein: creatinine ratio > ULN
3. Hematuria: ≥ 2+ by dipstick or > 10 RBCs/High Power Field (HPF) (without menstruation)
4. Thrombocytopenia: < 100,000 platelets/mm^3
5. Hemolysis: by blood smear or increased reticulocyte count

**Historical baseline creatinine of the patient*

[155] References 39 and 40 added per Amendment 1

14.3 Tender and Swollen Joint Count Assessment Form

TENDER & SWOLLEN JOINT COUNT ASSESSMENTS				
	**Left**		**Right**	
	Swollen	Tender	Swollen	Tender
Shoulder				
Elbow				
Wrist				
MCP 1				
2				
3				
4				
5				
PIP 1				
2				
3				
4				
5				
Knee				
**Subtotal**				
**TOTAL**	Swollen		Tender	

0 = Absent 1 = Present 9 = Replaced/N.A.*

*Injected joints will not be assessed for a period of one month (30 days) and will be documented as "Replaced/N.A."
