# Supplementary material for: Riociguat in patients with early diffuse cutaneous systemic sclerosis (RISE-SSc): randomised, double-blind, placebo-controlled multicentre trial
Source: Ann Rheum Dis. 2020 Apr 15;79(5):618–25. doi: 10.1136/annrheumdis-2019-216823 (PMC7213318; doi:10.1136/annrheumdis-2019-216823)
Supplement: Supplementary data [file annrheumdis-2019-216823supp003.pdf]

**Document Type:**	Statistical Analysis Plan
**Official Title:**	A Randomized, Double-Blind, Placebo-Controlled Phase II Study to Investigate the Efficacy and Safety of Riociguat in Patients With Diffuse Cutaneous Systemic Sclerosis (dcSSc)
**NCT Number:**	NCT02283762
**Document Date:**	22 Dec 2017

Statistical Analysis Plan

Protocol No.: BAY 63-2521/16277 Page: 1 of 38:

A Randomized, Double-Blind, Placebo-Controlled Phase II Study to Investigate the Efficacy and Safety of Riociguat in Patients With Diffuse Cutaneous Systemic Sclerosis (dcSSc)

Riociguat in diffuse cutaneous systemic sclerosis (dcSSc)

**Bayer study drug**	BAY 63-2521 / Riociguat		
**Study purpose:**	To evaluate the efficacy and safety of 52 weeks of treatment with riociguat versus placebo in subjects with diffuse cutaneous systemic sclerosis (dcSSc)		
**Clinical study phase:**	IIb	**Date:**	22 Dec 2017
**Study No.:**	16277	**Version:**	4.0
**Author:**	PPD		

Confidential

The information provided in this document is strictly confidential and is intended solely for the guidance of the clinical investigation. Reproduction or disclosure of this document, whether in part or in full, to parties not associated with the clinical investigation or its use for any other purpose without the prior written consent of the sponsor is not permitted.

Throughout this document, symbols indicating proprietary names (®, TM) are not displayed. Hence, the appearance of product names without these symbols does not imply that these names are not protected.

This Statistical Analysis Plan is produced on a word-processing system and bears no signatures.

The approval of the Statistical Analysis Plan is documented in a separate Signature Document.

Reference Number: RD-OI-0119
Supplement Version: 8

Table of Contents

1. Introduction ... 6

2. Study Objectives ... 6

3. Study Design ... 7
3.1 Design overview ... 7
3.1.1 Screening phase (up to 2 weeks) ... 8
3.1.2 Main study treatment phase (Week 0 to Week 52) ... 8
3.1.3 Termination visit and safety follow-up visit ... 10
3.1.4 Definition of rescue therapy ... 10
3.2 Primary variable ... 11
3.3 Justification of the design ... 11
3.4 End of study ... 11

4. General Statistical Considerations ... 12
4.1 General Principles ... 12
4.2 Handling of Dropouts ... 12
4.3 Handling of Missing Data ... 13
4.4 Interim Analyses and Data Monitoring ... 13
4.5 Data Rules ... 14
4.5.1 Definition of baseline and handling of repeated measurements ... 14
4.5.2 Definition of treatment-emergence ... 14
4.5.3 Definition of regions ... 14
4.6 Blind Review ... 14

5. Analysis Sets ... 15
5.1 Assignment of analysis sets ... 15

6. Statistical Methodology ... 15
6.1 Population characteristics ... 16
6.1.1 Demographics and baseline characteristics ... 16
6.1.2 Medical history and concomitant medication ... 16
6.1.3 Study medication duration and exposure ... 17
6.2 Efficacy ... 18
6.2.1 Primary efficacy ... 19
6.2.2 Secondary efficacy ... 22
6.2.3 Further clinical outcomes ... 25
6.2.4 Other exploratory parameters ... 26
6.2.5 Subgroup analysis ... 27
6.3 Pharmacokinetics/pharmacodynamics ... 28
6.4 Safety ... 29
6.4.1 Adverse events and mortality ... 29
6.4.2 Laboratory data ... 30
6.4.3 Other safety parameters ... 30
6.4.4 Subgroup analysis ... 30

Khanna D, *et al. Ann Rheum Dis* 2020; 79:618–625. doi: 10.1136/annrheumdis-2019-216823

7. Document history and changes in the planned statistical analysis 30

8. References 31

9. Appendices 32
9.1 Appendix 1: Pattern-mixture modeling approach 33
9.2 Appendix 2: Tipping point analysis...37
9.3 Appendix 3: Calculation of confidence intervals using Mantel-Haenszel weighting scheme 38

Reference Number: RD-OI-0119
Supplement Version: 8

Abbreviations

ACE	Angiotensin converting enzyme
ACR	American College of Rheumatology
ADS	Analysis dataset
AE	Adverse event
ANCOVA	Analysis of covariance
AT1	Angiotensin type 1 receptor
ATC	Anatomical therapeutic chemical
BDG	Bayer Drug Grouping
BMI	Body mass index
CI	Confidence interval
CRISS	Combined Response Index for Systemic Sclerosis
CRF	Case report form
CRP	C-reactive protein
CSR	Clinical study report
CTEPH	Chronic thromboembolic pulmonary hypertension
CV	Coefficient of variation
dcSSc	Diffuse cutaneous systemic sclerosis
DL_{CO}	Diffusion capacity of the lung for carbon monoxide
DMC	Data Monitoring Committee
ECG	Electrocardiogram
ERA	Endothelin receptor antagonist
EU	European Union
EULAR	European League Against Rheumatism
FAS	Full analysis set
FVC	Forced vital capacity
HAQ-DI	Health Assessment Questionnaire disability index
HEOR	Health Economics & Outcomes Research
HRQoL	Health-related quality of life
IDT	Individual dose titration
ILD	Interstitial lung disease
ITT	Intent-to-treat
IxRS	Telephone- or web-based response system
lcSSc	Limited cutaneous systemic sclerosis
LOCF	Last observation carried forward
LOS	Listing only set
LOQ	Limit of quantification
LS means	Least square means
MAR	Missing at random
MedDRA	Medical Dictionary for Regulatory Activities
MMRM	Mixed model repeated measures
mRSS	modified Rodnan skin score
NSAID	Nonsteroidal anti-inflammatory drug
PAH	Pulmonary arterial hypertension
PPI	Proton pump inhibitor
PPS	Per-protocol set
PROs	Patient-reported outcomes
PT	Preferred term
SAE	Serious adverse event
SAP	Statistical analysis plan
SAS	Statistical analysis system
SBP	Systolic blood pressure

Khanna D, *et al. Ann Rheum Dis* 2020; 79:618–625. doi: 10.1136/annrheumdis-2019-216823

SF-36	Short Form 36
SHAQ	Scleroderma Health Assessment Questionnaire
SOC	System organ class
SSc	Systemic sclerosis
TEAE	Treatment-emergent adverse event
TLF	Tables, Listings and Figures
TID	*ter in die*, 3 times a day
TPN	Total parenteral nutrition
VAS	Visual analog scale
WHO-DD	World Health Organization Drug Dictionary

1. Introduction

Systemic sclerosis (SSc) is a rare, orphan disease featuring chronic, fibrosing, autoimmune responses characterized by small vessel vasculopathy, autoantibody production, and fibroblast dysfunction leading to increased deposition of extracellular matrix. Systemic sclerosis is further divided into 2 subtypes defined by the extent of skin involvement: limited cutaneous systemic sclerosis (lcSSc) and diffuse cutaneous systemic sclerosis (dcSSc).

Both dcSSc and lcSSc are associated with internal organ involvement; however, patients with dcSSc are at greater risk for clinically significant major organ dysfunction. Diffuse cutaneous SSc is one of the most fatal rheumatic diseases, and is associated with substantial morbidity and many detrimental effects on health-related quality of life (HRQoL).

Currently, no therapy has been proven to reverse the vascular and fibrotic damage in patients with scleroderma. However, due to the high medical need, a number of drugs, such as methotrexate, mycophenolate mofetil, cyclophosphamide, azathioprine, and cyclosporine, are used off-label in an attempt to slow the progression of fibrosis. Current treatment options only target various SSc-related symptoms. No disease-modifying drug is available for SSc. In the European Union (EU), only bosentan is approved "to reduce the number of new digital ulcers in patients with SSc and ongoing digital ulcer disease" thus addressing also only one aspect of the disease.

Based on the positive results of riociguat in patients with PAH and chronic thromboembolic pulmonary hypertension (CTEPH) together with the compound's known anti-proliferative and antifibrotic effects as seen in vitro and in animal models, patients with SSc may benefit from treatment with riociguat. The current study will be the first study testing riociguat in this indication. The efficacy and safety of riociguat in patients with dcSSc will be evaluated.

This Statistical Analysis Plan is based on the following document(s):

Final Clinical Study Protocol version 5.0 dated 02 MAY 2017

This SAP describes the double-blind placebo-controlled main study treatment phase (52 weeks plus 30 day safety follow-up if applicable). A separate SAP will be presented to describe the long-term extension phase. Both analyses will be presented in two separate Clinical Study Report (CSR). No formal interim analyses are currently planned. An independent data monitoring committee (DMC) will be involved in the review of data for safety. Blinded adjudication of further clinical outcomes will be performed by an independent Central Adjudication Committee as described in the Adjudication Committee charter.

2. Study Objectives

The overall objective of this study is to evaluate the efficacy and safety of 52 weeks of treatment with riociguat versus placebo in subjects with dcSSc.

The primary objective of this study is:

- To assess the efficacy of riociguat administered 3 times a day (TID) as compared with placebo in terms of change in the mRSS from baseline to Week 52

Khanna D, *et al. Ann Rheum Dis* 2020; 79:618–625. doi: 10.1136/annrheumdis-2019-216823

The secondary objectives of this study are to assess the efficacy of treatment with riociguat administered TID as compared with placebo in terms of:

- Efficacy:
    - Key Secondary Objective
        - American College of Rheumatology Combined Response Index for Systemic Sclerosis (CRISS)
    - Further secondary objectives:
        - Health Assessment Questionnaire disability index (HAQ-DI) domain (separately from the Scleroderma Health Assessment Questionnaire [SHAQ] as part of the calculation of the CRISS algorithm)
        - Patient's global assessment
        - Physician's global assessment
        - Change in FVC (forced vital capacity) % predicted
- Further clinical:
    - New renal crisis
    - Worsening of cardiac disease, defined as new or worsened clinically symptomatic and significant heart disease, considered secondary to dcSSc, including congestive heart failure requiring hospitalization, new onset pulmonary hypertension requiring treatment, pericardial disease requiring intervention or exhibiting clinical decompensation, and arrhythmias and/or conduction defects requiring treatment
    - Worsening of gastrointestinal disease requiring hospitalization or new requirement for parenteral nutrition
    - Critical digital ischemia requiring hospitalization, or digital gangrene

Additional information regarding the events to be adjudicated may be requested to be sent to the Adjudication Committee. All events listed above, in addition to all cases of death, regardless of causality or seriousness will be reviewed by the Adjudication Committee.

3. Study Design

3.1 Design overview

In this multinational, multicenter, randomized (1:1), double-blind, placebo-controlled, parallel-group study, a total of approximately 200 subjects are planned for enrollment in order to randomize 130 subjects to study drug treatment (approximately 65 subjects to the riociguat group and 65 to the placebo group).

The study design consists of a main study treatment phase followed by a long-term extension phase as follows:

- **Screening phase:** up to 2 weeks
- **Main study treatment phase:** 52 weeks of double-blind treatment, consisting of:
  - Dose titration period of up to 10 weeks
  - Maintenance period of up to 42 weeks
- **Long-term extension phase**
  - Dose titration phase of up to 10 weeks (double-blind)
  - Open-label extension period
- **Termination visit and safety follow-up visit:** A termination visit and a safety follow-up visit will take place for all patients who discontinue study drug or withdraw from the study.

Figure 3-1 Main Study Treatment phase design

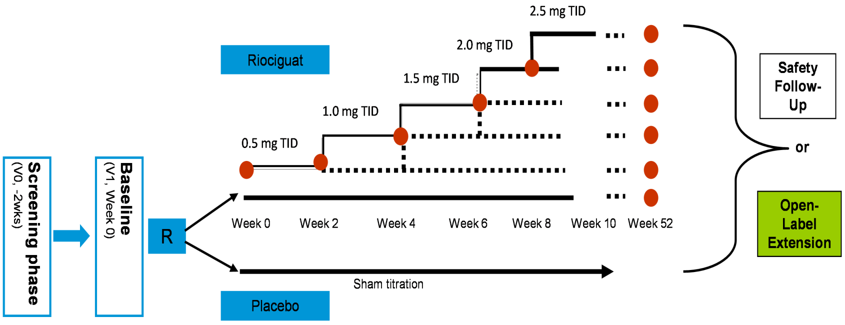

3.1.1 Screening phase (up to 2 weeks)

After providing written informed consent, subjects will undergo a screening evaluation to determine their eligibility (see Section 7.1 of protocol for a detailed schedule of assessments). Subjects will complete a diary detailing the number and duration of Raynaud's attacks per day for a period of 7 days leading up to the start of treatment / Day 0.

3.1.2 Main study treatment phase (Week 0 to Week 52)

At the baseline visit, subjects who have met all of the inclusion and none of the exclusion criteria will be randomized via a telephone-based or web-based response system (IxRS) to receive treatment with either active study drug (riociguat) or placebo (Day 0, Visit 1).

<u>Dose titration phase (Visits 1 to 6)</u>

During the first 10 weeks of the main study treatment phase, subjects will undergo individual dose titration.

The starting dosage is 0.5mg TID. The dose should be increased by 0.5 mg increments no sooner than 2 weeks apart to 1 mg, 1.5 mg, 2 mg, and 2.5 mg TID, resulting in a maximum total daily dose of 7.5 mg. Subjects will be maintained on a lower dose if higher doses are not tolerated (minimum dosage of 0.5 mg TID, total daily dose 1.5 mg; see Section 5.2.1 of protocol).

To maintain blinding of the treatment arms, patients randomized to the placebo group will undergo sham titration from Visit 1 onwards during the dose-titration period according to the rules of the individual dose titration scheme. At Visit 6, the last visit of the Titration Phase, no further increase in study medication will be possible.

<u>Dose titration algorithm</u>

The study medication dose for the next titration step will be determined every 2 weeks according to the patient's well-being and the peripheral systolic blood pressure (SBP) measured at trough before intake of the morning dose according to the following algorithm (dose titration scheme):

- If SBP is ≥ 95 mmHg and the subject has no signs or symptoms of hypotension, the dosage should be increased by 0.5 mg TID.
- If SBP is < 95 mmHg, the dosage should be maintained provided the subject <u>does not show any signs or symptoms of hypotension</u>.
- If SBP is < 95 mmHg, and the subject exhibits signs or symptoms of hypotension, the current dosage should be decreased by 0.5 mg TID.

During the dose-titration period a dose reduction can be performed according to patient well- being / safety concerns at investigator's discretion irrespective of SBP. If the investigator requests an increase or decrease in study medication dose via IxRS, the subsequent dose modification will not exceed ± 0.5 mg TID. Dose increases are allowed after a down-titration provided it is not done sooner than two weeks apart.

<u>Maintenance period (Visits 7 to 12)</u>

The overall duration of the main treatment phase is 52 weeks, including the dose titration period. At the end of the dose titration period (Week 10) the patient's maintenance dose will be determined using the treatment algorithm above. No further increase in dose will be allowed. The established dose will then be taken as the "optimal dose" to be administered for the remaining duration of the main treatment phase (up to Week 52).

Dose reductions for safety reasons (e.g., in case of any treatment-emergent adverse event [TEAE]) are allowed, but a subsequent dose increase during the maintenance period is not permitted.

From Week 26 (Visit 10), subjects will have the opportunity to add "rescue therapy", consisting of immunosuppressant drugs, to their randomized study medication if they meet certain criteria for worsening skin or pulmonary disease.

Dose Interruptions

Although not intended, patients may interrupt their intake of study medication for various reasons (e.g., hospitalization in a remote hospital without study medication access, safety reasons or side effects). If treatment is interrupted, the following rules should be applied:

Main treatment phase:

- ≤ 3 consecutive days without treatment (9 missing doses) in the dose-titration period: restart with last dose
- > 3 consecutive days in the dose-titration period: Discontinue, per amendment 4, the patient from study medication
- > 3 days but ≤ 14 consecutive days without treatment during the maintenance phase: treatment can be restarted at the discretion of the investigator at 0.5 mg TID lower than the last dose
- > 14 consecutive days without treatment in the maintenance phase: Discontinue, per amendment 4, the patient from study medication.

3.1.3 Termination visit and safety follow-up visit

A termination visit should be performed for patients who discontinue, per amendment 4, from study medication for any reason except death or lost to follow-up, and should occur as soon as possible after the patient receives his/her last dose of study drug. In general, at the Termination Visit the same safety and efficacy relevant measurements and procedures should be performed as at Visit 12. If the discontinuation occurs before Visit 8 (Week 14), the skin biopsy and the blood samples for biomarkers are to be collected. No blood samples for PK have to be taken and vital signs post dose are not needed because no new medication will be dispensed to the patient. If the Termination Visit will be performed after Visit 12 (Week 52) the patient's and physician's global assessment, patient interference with skin assessment, and tender and swollen joint count assessment must not be performed.

A safety follow-up visit (30 [+5] days after the last dose of study medication) will be performed for all the patients.

If the patient discontinues study drug prematurely during the main treatment phase (Week 0 – Week 52) he/she will be invited to come for limited assessments (mRSS, pulmonary function test [FVC and DL_{CO} including haemoglobin measurement at the local laboratory], and patient-reported outcomes [PROs] and patient's and physician's global assessment), at Week 12, Week 26, Week 39, and Week 52, depending on the timing of discontinuation.

The collection of these assessments is important for endpoint analysis which considers all assessments post baseline up to Week 52.

3.1.4 Definition of rescue therapy

Rescue therapy is defined as follows:

From Week 26 (Visit 10), patients with the following will have the opportunity to add rescue therapy to their randomized study medication. "Rescue therapy" is defined as treatment with an immunosuppressant drug, under the following situations:

- Worsening of skin disease (defined as > 5 units and ≥ 25% increase in mRSS), or
- Relative decline in FVC % predicted by ≥ 10%, or relative decline in FVC % predicted between ≥5% and < 10% with associated relative decline in DL_{CO} % predicted by ≥ 15%, provided that the decline in FVC results in FVC <75% of predicted (confirmed by repeat pulmonary function testing within 1 month).
- Worsening of inflammatory joint disease or myositis.

Rescue therapy may include any of the following 4 agents: methotrexate, mycophenolate mofetil, cyclophosphamide or azathioprine. In addition hydroxychloroquine will be allowed for the treatment of inflammatory joint disease and myositis.

3.2 Primary variable

Primary efficacy outcome measure is the change in mRSS from baseline to Week 52.

The mRSS is a validated physical examination method for estimating skin induration. It correlates with biopsy measures of skin thickness and reflects prognosis and visceral involvement, especially in early disease. It is scored on 0 (normal) to 3+ (severe induration) ordinal scales over 17 body areas, with a maximum score of 51 and is used to categorize severity of SSc. This assessment should be performed by a physician who is experienced and trained in skin scoring. To prevent inter-observer variability, the same physician must perform skin scoring for the same patient throughout the entire study.

Frequency: Screening Visit (Visit 0), Visit 7 (Week 12), Visit 10 (Week 26), Visit 11 (Week 39), Visit 12 (Week 52).

3.3 Justification of the design

Riociguat has been safe and well tolerated in previous clinical studies at multiple doses between 0.5 and 2.5 mg TID in subjects with PAH. Based on the positive results of riociguat in subjects with PAH and CTEPH together with the compound’s known antifibrotic effects as seen in in vitro and in animal models, subjects with symptomatic dcSSc may benefit from treatment with riociguat. Currently, there is no approved therapy for dcSSc and no suitable active comparator in this target population. Therefore, the purpose of this study is to evaluate the efficacy and safety of riociguat versus placebo in subjects with symptomatic dcSSc. The follow up time of 12 months has been chosen for the primary endpoint since it is considered an appropriate observation period to detect significant changes in the modified Rodnan Skin Score (mRSS) and is therefore used in many clinical studies with skin fibrosis [1, 2, 3]. Moreover, the 12 month trial duration is supported and validated by a 3-round Delphi consensus of measures for clinical trials of SSc [4].

3.4 End of study

For each participating EU country, the end of the study according to the EU Clinical Trial Directive will be reached when the last visit of the last subject for all centers in the respective country has occurred.

The end of the study as a whole will be reached as soon as the end of the study according to the above definition has been reached in all participating countries (EU and non-EU).

4. General Statistical Considerations

4.1 General Principles

The statistical evaluation will be performed by using the software package SAS release 9.2 or higher (SAS Institute Inc., Cary, NC, USA).

The statistical analysis will conform to Bayer Global Standard Tables version 3.0, 16 JAN 2017 with any riociguat project-specific options for these tables, and also any additional riociguat project-specific tables (Riociguat Standard Tables version 2.0, 19 JUN 2013). Subject data listings will conform to Bayer Global Standard Listings version 3.1, 16 JAN 2017. Additional data summaries not contained within these standards will be study specific.

All variables will be analyzed by descriptive statistical methods. The number of data available and missing data, mean, standard deviation, minimum, median, quartiles (if data are clearly non-normal) and maximum will be calculated for continuous data. Frequency tables will be generated for categorical data.

If not mentioned otherwise, all statistical tests will be performed two-sided with a type I error rate of α=5%.

Efficacy and safety analyses will be performed in patients valid for the full analysis set (FAS) and will be based on randomized treatment. Investigators were instructed not to make the call to the IxRS system until they were certain the patient is valid to be treated with study medication, on the day that study medication is to start. In that way, we expect all patients who are randomized also to be treated, so the FAS population would then be consistent with the full intent-to-treat (ITT) definition.

For the primary and secondary efficacy and safety endpoints (meaning further clinical outcomes), as a supportive analysis, a per protocol set (PPS) will also be defined. Definitions of the analysis sets are defined in Section 5.1.

4.2 Handling of Dropouts

The frequency of enrolled subjects not completing screening, treatment phase, and associated reasons will be summarized.

A patient who, for any reason (e.g., failure to satisfy the selection criteria), terminates the study before the time point used for the definition of “dropout” (see below) is regarded a “screening failure”. Restarting the defined set of screening procedures to enable the “screening failure” patient’s participation at a later time point is not allowed.

A patient who discontinues study participation prematurely for any reason is defined as a “dropout” if the patient has already been randomized.

Any randomized patient removed from the trial should undergo the assessments at the termination visit. There will be no replacement of randomized patients who withdraw from the study or prematurely discontinue study medication.

4.3 Handling of Missing Data

All missing or partial data will be presented in the subject data listing as they are recorded on the Case Report Form (CRF).

The number of subjects, who prematurely discontinue the study and study treatment for any reason, as well as the reasons for premature discontinuation of study and study treatment, will be reported. Kaplan-Meier plots for "Time to end of study" and "Time to end of study treatment" will be provided.

The number, timing and pattern of treatment discontinuations and dropouts affecting the primary efficacy variable will be displayed by visualization. All dropouts will be carefully evaluated with respect to demographics variables.

General rules

When appropriate, the following rules will be implemented so as not to exclude subjects from statistical analyses due to missing or incomplete data:

- **Efficacy Variables**
  See Section 6.2, for details of imputation for missing data regarding efficacy variables.
- **Safety Variables**
  When only partial dates are available, the following rules will be used for the derivation:

  If either the day or month of the start date of the adverse event is missing, then a worst case assumption is made for the treatment-emergent flag. For example, if study medication starts on 15 JAN 2016 and the adverse event start date is recorded as JAN 2016, then this is considered treatment-emergent, as it is possible the adverse event started while the patient is on study medication.

4.4 Interim Analyses and Data Monitoring

A formal interim analysis of the main study treatment phase is not planned. The main study treatment phase will be unblinded when the last subject completes main treatment phase and the database for the main study treatment phase is declared clean.

An independent Data Monitoring Committee (DMC) will meet periodically to review the safety data of enrolled subjects, as well as the continuing scientific merit of the trial. The DMC may recommend termination, temporary suspension, intervention of treatment arm or modification of the study for safety concern based on clinical judgment. In terms of a negative benefit/risk assessment, the DMC may recommend stopping the trial.

Blinded adjudication of further clinical outcomes as described in protocol Section 2, will be performed by an independent Central Adjudication Committee as described in the Adjudication Committee charter.

Khanna D, *et al. Ann Rheum Dis* 2020; 79:618–625. doi: 10.1136/annrheumdis-2019-216823

4.5 Data Rules

Analysis datasets (ADS) containing all derived variables needed for the statistical evaluation will be generated. The structure of the ADS and the contained variables will be described in a separate specification document.

Efficacy analysis datasets will be created that include key data, such as demography, flag for use of rescue medication, baseline efficacy, etc. that will be used as factors in the analysis models. These datasets will also include results from the imputations of missing values for the LOCF / analysis of covariance (ANCOVA) approach, such that the efficacy analyses can be performed without the need for major pre-processing within the statistical analysis programs. Relative days and flags for treatment emergent events are included in the databases.

The rules for data handling are described in detail in the Project Data Handling Rules, current version 1.1, dated 27 JAN 2014, and any updated versions becoming available during the course of this study.

4.5.1 Definition of baseline and handling of repeated measurements

Baseline is defined as the last set of non-missing measurements taken prior to the first study medication intake.

In case of multiple measurements per post baseline visit, the last non-missing value per visit will be taken for analysis.

4.5.2 Definition of treatment-emergence

Post-treatment values will be considered treatment-emergent if they start within 2 calendar days after the last day of study drug administration, i.e. the treatment-emergent window will be 2 days.

4.5.3 Definition of regions

The following regions are planned for regional analyses when there are at least 20 patients per grouping:

- Europe and Australia/New Zealand: Belgium, Netherlands, Switzerland, Germany, Czech Republic, Hungary, Turkey, Spain, Italy, France, UK, Australia, and New Zealand
- North America: US and Canada
- East Asia: Japan

4.6 Blind Review

The results of the validity review meeting will be documented in the Validity Review Report and may comprise decisions and details relevant for statistical evaluation. Any changes to the statistical analysis prompted by the results of the validity review meeting will be documented in an amendment or, if applicable, in a supplement to this SAP.

5. Analysis Sets

5.1 Assignment of analysis sets

Final decisions regarding the assignment of subjects to analysis sets will be made during the Validity Review Meeting and documented in the Validity Review Report (see Section 4.6) and stored in the clinical database prior to clean database.

Full analysis set (FAS)

The full analysis set is defined as all patients randomized and treated with study medication. Investigators will be instructed not to make the call to the IxRS system until they are certain the patient is valid to be treated with study medication, on the day that study medication is to start. In that way, we expect all patients who are randomized also to be treated, so the FAS population would then be consistent with the full intent-to-treat (ITT) definition.

Per protocol set (PPS)

As specified in Section 8.2 protocol, patients are valid for the PP analysis set (PPS), if they meet the major inclusion and exclusion criteria at randomization that may affect efficacy, are not taking excluded concomitant medications during the study that could have an effect on efficacy (not including rescue medication after Week 26), have the mRSS assessed at baseline and at least once during the main treatment phase and who are at least 80% compliant with study medication or do not have any additional major protocol deviations.

Additional major protocol deviations could include any of the following criteria not being satisfied:

1. Systemic sclerosis, as defined by ACR/EULAR 2013 criteria

2. dcSSc according to the LeRoy criteria, i.e. skin fibrosis proximal to the elbows and knees in addition to acral fibrosis

3. Disease duration of ≤ 18 months (defined as time from the first non−Raynaud's

phenomenon manifestation)

4. ≥ 10 and ≤ 22 mRSS units at the screening visit

5. The same physician evaluating mRSS at baseline and at Week 52 or at termination visit.

Final decisions regarding validity will be made during the Validity Review Meeting and documented in the Validity Review Report.

6. Statistical Methodology

Although dose titration is in use, the descriptive and inferential analyses will include just the riociguat and placebo groups.

6.1 Population characteristics

6.1.1 Demographics and baseline characteristics

Demographic variables and baseline characteristics will be summarized by treatment group and overall for both analysis populations (i.e., valid for FAS and PPS).

The following demographic data will be recorded:

- Date of birth (month and year) (age)
- Sex
- Ethnicity
- Smoking status, including number of cigarettes per day
- Alcohol consumption

Summary statistics (number of data available and missing data, mean, standard deviation, minimum, median and maximum) will be presented for continuous variables. Frequency tables will be presented for categorical variables. Smoking history and status will be summarized. Age will be summarized as a continuous variable and as a categorical variable categorized into two groups (< 65 years, ≥ 65 years).

6.1.2 Medical history and concomitant medication

Medical history and concomitant medication will be summarized by treatment group and overall for both analysis populations (i.e., valid for FAS and PPS).

Medical history findings will be coded by Medical Dictionary for Regulatory Activities (MedDRA) codes. System organ class (SOC) and preferred term (PT) will be used in tabulations.

Medical history findings (i.e., previous diagnoses, diseases or surgeries) meeting all criteria listed below will be collected:

- Pertaining to the study indication
- Started before signing of the informed consent
- Considered relevant to the study
- Medical history related to concomitant therapy

In the following differentiation between medical history and AEs, the term "condition" may include abnormal e.g., physical examination findings, symptoms, diseases, laboratory, ECG.

- Conditions that started before signing of informed consent and for which no symptoms or treatment are present until signing of informed consent are recorded as medical history (e.g., seasonal allergy without acute complaints).
- Conditions that started before signing of informed consent and for which symptoms or treatment are present after signing of informed consent, at unchanged intensity, are recorded as medical history (e.g., allergic pollinosis).

- Conditions that started or deteriorated after signing of informed consent will be documented as AEs.

Prior and concomitant medications will be coded by Anatomical Therapeutic Chemical (ATC) classification system according to the World Health Organization Drug Dictionary (WHO-DD). The ATC class is taken from the WHO-DD code (first character) and the ATC subclass is taken from the WHO-DD code (first 3 characters).

Bayer Drug Groupings (BDG) will be used to select concomitant medications of special interest. The selected concomitant medications will be summarized by parent BDG, BDG and substance name. The following medication groups are of special interest:

- Antacids and other gastrointestinally protective drugs, corticosteroids, phosphodiesterase inhibitors with unspecified type, proton pump inhibitors (PPIs)
- Antidepressants including adjunct antidepressant therapy, herbal antidepressants, monoamine oxidase A inhibitors, monoamine oxidase inhibitors (non-selective), non-selective monoamine reuptake inhibitors, other antidepressants, selective serotonin reuptake inhibitors
- Antihypertensives (subgroups) including ACE inhibitors and calcium channel blockers.
- CYP inducers including CYP3A4 inducers and CYP3A4 inhibitors
- Non-steroidal anti-inflammatory drugs (NSAIDs) including NSAIDS (excluding salicylic acid and derivatives), salicylic acid and derivatives, P-gp inhibitors and inducers including P-gp inducers and P-gp inhibitors.
- Vasodilators including endothelin receptor antagonists, NO donors including nitrates, natriuretic peptides, phosphodiesterase inhibitors with unspecified type (vasodilators), phosphodieterase type 1 inhibitors, phosphodieterase type 3 inhibitors, phosphodiesterase type 5 inhibitors, prostacyclins and prostacyclin analogues, prostaglandins with vasodilating effect (excluding prostacyclins), and vasodilators (others).
- Disease modifying anti-rheumatic drugs, including Biological DMARDs, for example tumour necrosis factor–blocking agents, anakinra, abatacept, rituximab, etc. and Non-biological DMARDs, for example sulfasalazine, hydroxychloroquine, methotrexate, leflunomide, etc.

6.1.3 Study medication duration and exposure

Study medication duration and exposure will be summarized overall for both analysis populations (i.e., valid for FAS and PPS).

The duration of study medication (in days) is derived by calculating the study medication durations by the following formula: period (last dose date – first dose date + 1). Descriptive statistics of treatment duration as well as frequency counts by treatment duration categories will be presented. Dose titration by visit, dose titration sequence and reasons for up- and down-titration by visit and dose will also be summarized using frequency counts.

The formula for compliance is:

(Total dispensed – total returned/ (Days between visits x dose[# of pills]) X 100

= What was taken / What should've been taken

x 100 = % Compliance

Example:

Subject comes in for study visit. The investigator dispenses 200 pills at this visit. When the subject returns to the clinic the study drug bottle has 120 pills remaining (i.e. 80 pills used). The subjects were instructed to take a dose of 3 pills daily. There were 31 days between the visits.

(200-120) / (31 x 3)

= 80 / 93

= 0.86 x 100 = 86% compliance

Compliance will be summarised descriptively and by using frequency counts by following categories: < 80%, 80 to 120%, > 120%. Separate compliance tables will be created for overall and main study phase.

6.2 Efficacy

In the main study treatment phase, a primary efficacy analysis by treatment group (descriptive and inferential) will be performed in patients valid for FAS and valid for PPS. The PPS is a sensitivity analysis, supportive of the FAS analysis. The PPS set will only be used for the primary efficacy endpoint analysis, namely the mixed model repeated measures (MMRM) approach (Method #1) for the mRSS. For the secondary efficacy and safety endpoints (meaning further clinical outcomes), a supportive analysis using PPS will be performed.

Other efficacy parameters will be analyzed using FAS.

In the efficacy analyses, countries and centers will be clustered by geographic region. Statistical analyses will be adjusted for these geographic regions. The aim is to have region groupings with a minimum sample size of 20 subjects, to be able to check for consistency of treatment effects across regions by analyzing the p-value from the main effect of region and treatment as well as the treatment by region interaction when fitting either an ANCOVA model or MMRM for change in mRSS from baseline to all assessments post baseline up to Week 52.

If the treatment by region interaction effect is statistically significant at alpha level of 5%, then this would imply that there is evidence of heterogeneity of treatment effects across regions. This would imply that the treatment effect depends upon the particular region. The purpose of investigating regional heterogeneity would be due to the possibility that there could be differences in patient or disease characteristics between regions. As a result, separate p-values for each region in addition to least square means with corresponding standard errors for the change in mRSS from baseline to Week 52 / end of study will be presented.

All the efficacy variables will be tabulated separately for all the observations and observations not on rescue medication, but on study treatment.

6.2.1 Primary efficacy

The primary efficacy variable mRSS, as well as absolute and relative change from baseline, will be summarized using descriptive statistics and produced for FAS and PPS.

In the following sub-sections several different estimands are described together with analysis techniques to estimate the respective estimands. According to common terminology (ICH E9 (R1) addendum on estimands and sensitivity analysis in clinical trials on the guideline on statistical principles for clinical trials [9]) an estimand requires the following:

a. Population
b. Variable
c. Measurement of intervention effect
d. Summary measure

In the following descriptions the population is omitted as the population is, for all estimands, described via the inclusion and exclusion criteria as stated in Section 7.2 protocol.

6.2.1.1 Method #1: MMRM modeling approach (primary approach)

The estimand of interest is the de facto or treatment policy estimand.

Variable: Change in mRSS from baseline to Week 52.

Measurement of intervention effect: Regardless of switching to rescue medication, stopping study treatment or adherence to study treatment.

Summary measure: Difference of (LS) (Least square) means at Week 52.

The model statement is:

$Y_{ijkl} = \mu + \beta x_i + t_k + r_l + v_j + (tv)_{jk} + s_i + \varepsilon_{ijkl}$

where Y_{ijkl} is the change from baseline in mRSS to visit j for subject i;
μ is the intercept,
β is the baseline covariate effect,
x_i is the baseline mRSS for subject i,
t_k is the fixed effect of treatment k,
r_l is the fixed effect of region l,
v_j is the fixed effect of visit j,
$(tv)_{jk}$ is the interaction effect of treatment k by visit j,
s_i is the random effect of subject i
$\varepsilon_{ijkl} \sim$ Normal(0, σ^2) represents the residual variance component with corr($\varepsilon_{ij}, \varepsilon_{ij'}) = \rho_{jj'}$, $j \neq j'$.

The hypothesis to be tested is the following:

Reference Number: RD-OI-0119
Supplement Version: 8

H_0: $\mu_{Riociguat} = \mu_{placebo}$ vs. H_1: $\mu_{Riociguat} \neq \mu_{placebo}$

LS mean estimates, estimates of treatment differences and corresponding 95% confidence intervals (CIs) will be computed by visit. Correlations among measurements taken on the same patient will be modelled with an unstructured covariance assumption. The degrees of freedom will be computed according to the method detailed by Kenward and Roger.

The core SAS-code to be used is the following:

```
proc mixed data=adqs;
    class subject treat region visit;
    model change =  treat region base visit treat*visit /ddfm=KR outp=resid s;
    repeated visit / subject=subject type=un group=treat;
    lsmeans treat*visit /cl diff;
    estimate 'Riociguat - Placebo'  treat 1 -1 ;
    estimate 'Riociguat - Placebo at Week 12' treat 1 -1 treat*visit  1 0 0 0 -1  0  0  0/cl;
    estimate 'Riociguat - Placebo at Week 26' treat 1 -1 treat*visit  0 1 0 0  0 -1  0  0/cl;
    estimate 'Riociguat - Placebo at Week 39' treat 1 -1 treat*visit  0 0 1 0  0  0 -1  0/cl;
    estimate 'Riociguat - Placebo at Week 52' treat 1 -1 treat*visit  0 0 0 1  0  0  0 -1/cl;
    ODS OUTPUT TESTS3=TYPE3 LSMeans=LSMEAN ESTIMATES=ESTIM;
run;
```

No imputations will be done, except if a patient has no post-baseline measurements, baseline will be used, meaning “no change” at Week 12. Missing data is assumed to be missing at random (MAR). The per protocol analysis will be supportive.

6.2.1.2 Method #2: MMRM modeling approach (secondary approach)

The estimand of interest is if all patients had adhered to study treatment and not taken rescue medication.

Variable: Change from baseline in mRSS from baseline to minimum of (Week 52, last measurement before stop of study medication or initiation of rescue medication).

Measurement of intervention effect: Not applicable, as contained in the definition of the variable.

Summary measure: Difference of (LS) means at Week 52 or stop of study medication or last visit before rescue medication.

This method will be a repeat of the MMRM modeling primary approach (Method #1), excluding those observations which occurred either after end of study treatment or while on rescue medication.

6.2.1.3 Method #3: ANCOVA modeling approach (LOCF for all observations regardless of study medication and/or rescue medication)

The estimand of interest is the de facto or treatment policy estimand at the last observation prior to dropout regardless of stop of study medication or rescue medication.

Variable: Change from baseline in mRSS from baseline to minimum of (Week 52, last observation before drop out).

Measurement of intervention effect: Not applicable, as contained in the definition of the variable.

Summary measure: Difference of (LS)means.

The primary ANCOVA model is specified as:

$Y_{ikl} = \mu + \beta x_i + t_k + r_l + \varepsilon_{ikl}$

where Y_{ikl} represents the change from baseline at Week 52/last visit before drop out in mRSS for subject *i,*
μ is the intercept,
x_i represents the baseline value for each subject *i*,
t_k represents the fixed effect of treatment *k,*
r_j is the fixed effect of region *j,*
$\varepsilon_{kli} \sim$ Normal(0, σ^2) represents random variation, for $i=1,\ldots,n$.

If the subject withdraws (i.e. drop out is present), the last observation will be used. The exception is death when a worst case imputation value (i.e., 51 for mRSS at a particular visit) will be used. If there is no measurement during the treatment phase, the baseline value will be used.

The hypothesis to be tested is the following:

H_0: $\mu_{Riociguat} = \mu_{placebo}$ vs. H_1: $\mu_{Riociguat} \neq \mu_{placebo}$

LS mean estimates, estimates of treatment difference and corresponding 95% CIs will be calculated.

The core SAS-code to be used is the following:

```
proc glm data=adqs;
  class treat region;
  model change = baseline treat region / solution ss3;
  lsmeans treat region/ cl pdiff stderr;
  ODS OUTPUT MODELANOVA = ANOVA LSMEANS = LSMEANS LSMEANCL = LSMEANSCL LSMEANDIFFCL = LSDIFF;
run;
```

6.2.1.4 Method #4: ANCOVA modeling approach (LOCF for on-study treatment but not on rescue medication)

The estimand of interest is the on-study treatment without rescue medication estimand at the last observation prior to dropout, stop of study medication or start of rescue medication, whatever comes first.

Variable: Change from baseline in mRSS from baseline to minimum of (drop out, stop of study medication, start of rescue medication).

Measurement of intervention effect: Not applicable, as contained in the definition of the variable

Summary measure: Difference of (LS)means.

This is the same analysis as repeated in the above section with the exception that if a subject stops taking study medication, the last observation while on study medication, but not on rescue

medication, will be used. More explicitly, a sensitivity analysis of change in mRSS to Week 52 / last visit will include all measurements recorded up to Week 52 or the termination visit, not including those measured while rescue medication was taken or study medication was stopped.

6.2.1.5 Method #5: Pattern-mixture modeling approach (sensitivity analysis)

The estimand of interest is the de facto or treatment policy estimand but with the value at Week 52 (if missing) estimated using multiple imputation. This is the mean difference in change from baseline in mRSS at Week 52 between riociguat and placebo in all randomized patients regardless of adherence to treatment or use of rescue medication. ANCOVA similar to Method #3 will be applied.

To address the concern regarding the possibility of missing data for mRSS not at random, a pattern-mixture model with control-based pattern imputation will be implemented. See technical details in Appendix 1.

6.2.1.6 Method #6: Tipping-point analysis (sensitivity analysis)

In addition to the sensitivity analyses specified above, tipping point sensitivity analyses that vary assumptions about the missing outcomes on the two treatment arms regarding the primary efficacy endpoint, i.e. change in mRSS from baseline to Week 52, will be conducted.

The estimand of interest is the de facto or treatment policy estimand but with the value at Week 52 (if missing) estimated using multiple imputation with various penalties. This is the mean difference in change from baseline in mRSS at Week 52 between riociguat and placebo in all randomized patients regardless of adherence to treatment or use of rescue medication. MMRM similar to Method#1 and ANCOVA similar to Method #3 will be applied.

To conduct this sensitivity analysis and taking into account the incompleteness of the data, multiple imputation will be used to draw sets of completed data. See technical details in Appendix 2.

6.2.2 Secondary efficacy

Key secondary endpoint:

- CRISS at Week 52, consisting of five variables: mRSS, FVC % predicted, physician and patient global assessments, and HAQ-DI score (from SHAQ patient reported outcomes)

Further secondary endpoints:

  - HAQ-DI domain (separately from the SHAQ as part of the calculation of the CRISS algorithm)
  - Patient’s global assessment
  - Physician’s global assessment
  - Change from baseline in FVC (forced vital capacity) % predicted

The following secondary endpoints will be tested in a hierarchical fashion at a 2-sided 5% level, only if the primary endpoint of mRSS is shown to be statistically significant at a 2-sided 5% level.

The following order of testing will be applied:

- CRISS
- Change in HAQ-DI from baseline to Week 52
- Change in Patient's global assessment from baseline to Week 52
- Change in Physician's global assessment from baseline to Week 52
- Change in FVC % predicted from baseline to Week 52

For example, if CRISS is statistically significant at a 2-sided 5% level, then the HAQ-DI will be tested; if not, the testing procedure will be stopped. This step is repeated further down the list of the 5 secondary endpoints until a non-statistically significant endpoint is reached. These particular secondary endpoints were chosen to be tested in this hierarchical fashion, because the mRSS and the above additional, secondary variables were considered to have the greatest face validity when designing the CRISS [7].

The primary estimand of interest for the key secondary efficacy variable is the de facto or treatment policy estimand, regardless of switching to rescue medication, stopping study treatment or adherence to study treatment.

Variable: CRISS at Week 52.

Measurement of intervention effect: Regardless of switching to rescue medication, stopping study treatment or adherence to study treatment.

Summary measure: The difference of proportions of improvers (analyzed with Mantel-Haesnzel weights)

All the further secondary variables will be analysed as the de facto or treatment policy estimand, regardless of switching to rescue medication, stopping study treatment or adherence to study treatment similar to Method#1 described in Section 6.2.1.1.

All the secondary efficacy variables will be summarized using descriptive statistics and produced for FAS and PPS.

American College of Rheumatology Combined Response Index for Systemic Sclerosis

Application of CRISS algorithm in a randomized clinical trial is a two-step process. Firstly, evaluate if any patient has met the criterion for "not-improved." If yes, these patients are assigned a probability score of 0.0. For the remaining patients, calculate probability based on change in five measures: mRSS, FVC%, HAQ-DI, patient global assessment, and physician's global assessment, where each measure has a probability score between 0 and 1. [7].

In Step 1, a patient is clinically evaluated to determine whether the patient has improved or not. Expert consensus on the definition of a patient who is not improved during a trial is the following:

Khanna D, *et al. Ann Rheum Dis* 2020; 79:618–625. doi: 10.1136/annrheumdis-2019-216823

A patient is considered not improved and is assigned a probability score of improving equal to 0.0, irrespective of improvement on other core items, if he/she develops any of the following:

1. new scleroderma renal crisis (AE term ‘scleroderma renal crisis’ to be used),

   Resolution: AE.AEDECOD (Coded term) = ‘scleroderma renal crisis’ will be used to pick these records.

2. decline in FVC% predicted ≥ 15 % (relative), confirmed by another FVC% within a month, HRCT to confirm ILD (if previous HRCT did not show ILD) and FVC% predicted below 80% predicted (attributable to SSC),

   Resolution: i) FVC% predicted < 80% AND ii) Change from baseline in FVC% predicted at Week 52 is ≥ -15% then subject meets criteria.

3. new onset of left ventricular failure (defined as ejection fraction ≤ 45%) or new onset of pulmonary arterial hypertension requiring treatment (attributable to SSC)

   Resolution: AE.AEDECOD (Coded term)=’ Left ventricular failure’ AND AE.AETERM (Reported term) should mention ejection fraction <=45 OR ii) AE.AEDECOD (Coded term)= ‘Pulmonary arterial hypertension’ will be used to pick up these records.

If the patient is determined to exhibit improvement in Step 1 (i.e., not assigned a 0.0), Step 2 involves computing the predicted probability of improving (a score between 0.0 and 1.0, inclusive) for each patient using the equation:

$$\frac{exp\left[-5.54 - 0.81 * \Delta_{MRSS} + 0.21 * \Delta_{FVC\%} - 0.40 * \Delta_{Pt-glob} - 0.44 * \Delta_{MD-glob} - 3.41 * \Delta_{HAQ-DI}\right]}{1 + exp\left[-5.54 - 0.81 * \Delta_{MRSS} + 0.21 * \Delta_{FVC\%} - 0.40 * \Delta_{Pt-glob} - 0.44 * \Delta_{MD-glob} - 3.41 * \Delta_{HAQ-DI}\right]}$$

where Δ_{MRSS} indicates the change in mRSS from baseline to follow-up, Δ_{FVC} denotes the change in FVC% predicted from baseline to follow-up, $\Delta_{Pt\text{-}glob}$ indicates the change in patient global assessment, $\Delta_{MD\text{-}glob}$ denotes the change in physician global assessment, and $\Delta_{HAQ\text{-}DI}$ is the change in HAQ-DI.

Note that all changes are absolute change ($Time_2$ –$Time_{baseline}$) and that physician’s and patient’s global assessment was measured on the Likert scale ranging from 0 to 10, where 0 = excellent, and 10 = extremely poor.

However, Step 2 assumes that there is complete data; that is, all five components of the CRISS are fully recorded at Week 52. If the patient has three or more missing components of the CRISS at this visit, a probability score of improving will be set equal to 0.0. If a patient has 1 or 2 missing components, then previous non-missing value of that particular component will be used. A table with number of non-responders with zero-imputation will be provided.

Subjects for which the CRISS score (predicted probability of improving) is greater or equal to 0.60 are considered improved, while subjects for which the CRISS score is below 0.60 are considered not

improved. The difference of proportions of improvers will be analyzed using Mantel-Haenszel weights, stratified by region. The method for calculating the standard errors and 95% confidence intervals is described in Appendix 3[8].

The predicted probabilities obtained using the CRISS at Week 52 will be assessed also as a continuous variable and the distributions of the probability of improving for patients on riociguat vs. placebo will be compared using a stratified Wilcoxon test [7].

The other secondary efficacy parameters (HAQ-DI domain, patient's global assessment, physician's global assessment, change in FVC % predicted will be analyzed using the same primary method as that for the mRSS; that is, MMRM (Method #1). In addition, descriptive statistics for the proportion of patients who experience worsening in FVC% predicted by 15% or more will be included.

For statistically significant secondary endpoints tipping point analyses will be performed. The analyses will be described in a supplemental SAP which will be prepared and signed prior the data base lock.

6.2.3 Further clinical outcomes

Further clinical outcomes based on observations from safety data are:

- New renal crisis
- Worsening of cardiac disease, defined as new or worsened clinically symptomatic and significant heart disease, considered secondary to dcSSc, including congestive heart failure requiring hospitalization, new onset pulmonary hypertension requiring treatment, pericardial disease requiring intervention or exhibiting clinical decompensation, and arrhythmias and/or conduction defects requiring treatment.
- Worsening of gastrointestinal disease requiring hospitalization or new requirement for parenteral nutrition
- Critical digital ischemia requiring hospitalization, or digital gangrene

These clinical outcomes will be summarized using descriptive statistics for FAS and PPS. Furthermore, these clinical outcomes, all being present or not present during the main study treatment phase, will be analyzed using Mantel-Haenszel weights as described in Section 6.2.2.

Time to clinical outcome variables (first occurrence) will be analyzed using the log-rank statistics for comparing the difference between treatment groups. Analyses will be stratified by region. Patients with no outcome in question will be censored at the last available visit date showing no evidence of outcome in respective analysis.

6.2.4 Other exploratory parameters

Other exploratory parameters are:

- mRSS progression rate (defined as increase in mRSS by > 5 units and ≥ 25% from baseline) and mRSS regression rate (defined as decrease in mRSS by > 5 units and ≥ 25% from baseline)
- Percentage of patients with ≥ 20%, 40%, or 60% improvement in mRSS from baseline to Week 52
- SF-36 (Variables: Bodily Pain, General Health, Mental Health, Physical Functioning, Role Emotional, Role Physical, Social Functioning, Vitality, Mental Component Score, Physical Component Score, Mental Health Enhanced score and Health Utility Index) [10.]
- SHAQ (VAS variables: pain, intestinal problems, breathing problems, Raynaud's symptoms, finger ulcers and overall severity). All the VAS scores will be converted to 0-3 scale by multiplying results in mm by factor 0.03 and rounding by 0.1 decimals [11.]
- Digital ulcer net burden (defined as total number of ulcers at time point minus number of ulcers at baseline) and proportion of patients who do not develop digital ulcers
- Proportion of patients who do not develop distal ulcers
- Change in DL_{CO} (carbon monoxide diffusing capacity) % predicted from baseline to Week 52
- Need for rescue therapy
- All-cause mortality (SSc associated and non-SSc associated deaths separately)
- Gastrointestinal involvement as assessed by University of California, Los Angeles, Scleroderma Clinical Trial Consortium Gastrointestinal Scale (UCLA SCTC GIT) 2.0 instrument
- Patient-Reported Outcomes Measurement Information System (PROMIS)-29 (subset of sites belonging to English-speaking countries) [12.]
- Patient interference with skin assessment (VAS)
- Presence or absence of tendon friction rubs
- Change in BMI from baseline to Week 52
- Joint involvement using tender and swollen joint counts
- Raynaud's attacks assessment:

  Patient and physician assessment of Raynaud's phenomenon (disease severity), average numbers over one week will be calculated for the following: pain, numbness, and tingling during a Raynaud's phenomenon attack, Raynaud's condition score, number of attacks/day and duration of attacks/day.
- Change in FVC (L) from baseline to Week 52

These exploratory variables will be summarized using descriptive statistics and FAS population. In particular, descriptive statistics for the number of subjects had progressed/regressed will be presented for both the full analysis set and per protocol set. In the case of death, this will be considered a progression and a non-regression.

The time to first mRSS progression and regression will be analyzed using the log-rank test, for comparing the difference between treatment groups. Analyses will be stratified by region. Patients with no progression or no regression will be censored at the last available visit date showing no evidence of progression or regression in respective analysis.

As a sensitivity analysis, the difference in incidences of mRSS progression and regression between treatment groups during the 52-week study period will be analyzed using Mantel-Haenszel weights, stratified by region similar to method explained in Section 6.2.2.

Percentage of patients with ≥ 20%, 40%, or 60% improvement in mRSS from baseline to Week 52 will be analyzed using Mantel-Haenszel weights as explained above.

In addition change from baseline to Week 52 of the following variables will be analyzed using the primary method (Method #1) for MMRM: SF-36, SHAQ (VAS scores), digital ulcer net burden, DL_{CO} (carbon monoxide diffusing capacity) % predicted, Gastrointestinal involvement total score (UCLA), PROMIS pain intensity score (0-10), patient interference with skin (VAS) score (0-10), BMI, and FVC (L).

ANCOVA similar to method #3 in Section 6.2.1.3 will be used in analysis of change from baseline to last visit of tender joint total score and swollen joint total score, Raynaud's condition score (0-10) as well as physician and patient assessment of Raynaud's disease severity (0-100).

The Mantel-Haenszel approach, similar to method explained in Section 6.2.2, will also be applied to the following explanatory efficacy variables: proportion of patients who do not develop digital ulcers, proportion of patients who do not develop distal ulcers, need for rescue therapy and all-cause mortality.

The time to first need for rescue therapy will be analyzed using the log-rank test, for comparing the difference between treatment groups. Analyses will be stratified by region. Patients with no rescue therapy will be censored at the last available visit date showing no evidence of need for rescue therapy.

6.2.5 Subgroup analysis

Descriptive analyses of the primary efficacy outcome measure (change in mRSS from baseline to Week 52 / end of study) will be performed for the following subgroups:

– region (North America, Europe and Australia/New Zealand, East Asia)
– gender (males/females)
– age (age < 65 years/age ≥ 65 years)
– mRSS at baseline (10 - 16 units/17 - 22 units)
– disease duration at baseline (0 - 6 months, 7 - 12 months, 13 - 18 months)

Khanna D, *et al. Ann Rheum Dis* 2020; 79:618–625. doi: 10.1136/annrheumdis-2019-216823

- antibody at baseline (SCL-70, RNA polymerase III, both, neither)
- ILD (defined with preferred terms: interstitial lung disease and pulmonaryfibrosis) at baseline (yes/no)
- FVC%, predicted at baseline (<50, 50-75, > 75)
- hsCRP elevated at baseline (≤ 3.0mg/l, > 3.0 mg/l; and ≤ 10.0 mg/l, > 10.0 mg/l)
- use of corticosteroids at baseline (yes/no)
- tendon friction at baseline (yes/no)

For each subgroup analysis, we will use the following general MMRM model:

$Y_{ijkl} = \mu + \beta x_i + t_k + g_l + v_j + (tv)_{jk} + (tg)_{kl} + s_i + \varepsilon_{ijkl}$

where Y_{ij} is the change from baseline in mRSS to visit j for subject i,
μ is the intercept,
β is the baseline covariate effect,
x_i is the baseline mRSS for subject i,
t_k is the fixed effect of treatment k,
g_l is the fixed effect of subgroup l,
v_j is the fixed effect of visit j,
$(tv)_{jk}$ is the interaction effect of treatment k by visit j,
$(tg)_{kl}$ is the treatment by subgroup interaction,
s_i is the random effect of subject *I,*
$\varepsilon_{ij} \sim \text{Normal}(0, \sigma^2)$ represents the residual variance component with $\text{corr}(\varepsilon_{ij}, \varepsilon_{ij'}) = \rho_{jj'}, \ j \neq j'$.

Analysis will be produced for both FAS and PPS sets. All the patients are included in the analyses regardless of switching to rescue medication , stopping study treatment or adherence to study treatment.

Forest plots showing the result of the primary efficacy variable (change in mRSS from baseline to Week 52 / end of study) with least square means and corresponding confidence intervals for each subgroup described above will be produced for both FAS and PPS.

6.3 Pharmacokinetics/pharmacodynamics

BAY 63-2521 and M1 peak and trough concentrations will be summarized per visit, separated according to actual dose for peak concentrations and according to previous dose for trough concentrations. The analyses will be focused on descriptive statistics. The following statistics will be calculated for each of the sampling points: arithmetic mean, standard deviation and coefficient of variation (CV), geometric mean, geometric standard deviation (re-transformed standard deviation of the logarithms), and CV, minimum, median, maximum value and the number of measurements. Boxplot of concentration of BAY 63-2521 by visit will be created.

Means at any time will only be calculated if at least 2/3 of the individual data were measured and were above the limit of quantification (LOQ). For the calculation of the mean value a data point below LOQ will be substituted by one half of the limit.

6.4 Safety

The safety analysis will be performed in the population of patients valid for FAS. All tabulations will be descriptive only.

6.4.1 Adverse events and mortality

The incidence of adverse events will be summarized using MedDRA preferred terms grouped by primary system organ class. The version number of MedDRA relevant for study evaluation will be stored in the study database.

The incidence of treatment-emergent AEs (TEAEs) will be tabulated by treatment group. More specifically, adverse events are considered to be treatment-emergent if they have started or worsened after first application of study drug up to 2 days after end of treatment with study drug. An overall summary of the number and percentage of patients with AEs and TEAEs will be presented by treatment arm. This summary will include the number and percent of patients with related AEs, related SAEs, worst intensity and AEs leading to permanent discontinuation.

Incidences of number of subjects with AEs will be summarized by treatment arm and MedDRA terms using frequency tables for the following AE types:

- Treatment-emergent adverse events (TEAEs)
- Treatment-emergent serious adverse events (TESAEs)
- Drug-related TEAEs
- Drug-related TESAEs
- TEAEs of special interests
- Drug-related TEAEs of special interest

Tables for maximum intensity and worst outcome for above categories will be provided.

The incidence of all AEs during pre-treatment and during the safety follow-up (that is, AEs occurring more than 2 days after end of treatment with study drug) will be tabulated separately.

Incidences of TEAEs and TESAEs per 100 person-years will be tabulated. The rate per 100 person-years is calculated as

Rate per 100 person-years = number of events / (total drug exposure in years / 100),

where 365.25 days are taken as one year.

Serious adverse events, deaths, adverse events leading to discontinuation and adverse events of special interest (as defined in the protocol Section 7.5.1.6) will be listed. The date, relative day (to study medication) and phase of the study (pre-treatment, during treatment, post-treatment) will be included.

Further summaries of adverse events by intensity and outcome, may be provided, consistent with Bayer Global Medical Standards.

Incidences of AEs of special interest will be tabulated. Currently the protocol Section 7.5.1.6 specifies symptomatic hypotension and serious hemoptysis as AEs of special interest.

6.4.2 Laboratory data

The safety evaluation of laboratory data will include:

- Incidence rates of treatment-emergent laboratory values outside of normal range.
- Incidence rates of pre-specified laboratory data abnormalities.
- Descriptive analysis of continuous laboratory parameters, and their changes from baseline by visit.
- Categorical analysis of transitions from low, normal, high from baseline to post baseline.

6.4.3 Other safety parameters

Descriptive analysis of weight and vital signs, their changes from baseline, and number of subjects with low/normal/high vital signs, will be performed by visit. Descriptive statistics of pulse oximetry will be performed by treatment group and visit. Assessments for orthostatic test will be listed. The number of subjects with abnormal ECG findings, as well as the number of subjects with treatment-emergent ECG abnormalities will be tabulated by treatment group.

6.4.4 Subgroup analysis

Descriptive analyses by baseline ILD (yes/no), defined with preferred terms: interstitial lung disease and pulmonaryfibrosis, will be performed as follows:

- Number of patients with ILD per treatment group (already listed as subgroup analysis in Section 6.2.5)
- TEAEs by treatment group
- TESAEs by treatment group
- Listing of SAEs in patients with ILD
- Lung function tests (FVC%, FVC, DLco%, DLco, DLco predicted) by treatment group
- Discontinuations of study medication by treatment group

7. Document history and changes in the planned statistical analysis

- Approval of the SAP version 3.0 dated 06 OCT 2017 (Update after CSP Amendment v6.0)
- Approval of the SAP version 2.0 dated 08 APR 2016 (Update after CSP Amendment v4.0)
- Approval of the SAP version 1.0 dated 03 SEP 2015

Khanna D, *et al. Ann Rheum Dis* 2020; 79:618–625. doi: 10.1136/annrheumdis-2019-216823

8. References

1. Khanna. Recombinant human relaxin in the treatment of systemic sclerosis with diffuse cutaneous involvement: a randomized, double-blind, placebo-controlled trial. Arthritis Rheum. 2009, 60(4):1102-11.

2. Pope JE, Bellamy N, Seibold JR, Baron M, Ellman M, Carette S, Smith CD, Chalmers IM, Hong P, O'Hanlon D, Kaminska E, Markland J, Sibley J, Catoggio L, Furst DE. A randomized, controlled trial of methotrexate versus placebo in early diffuse scleroderma. Arthritis Rheum. 2001, 44:1351-8.

3. Amjadi S, Maranian P, Furst DE, Clements PJ, Wong WK, Postlethwaite AE, Khanna PP, Khanna D. Course of the modified Rodnan skin thickness score in systemic sclerosis clinical trials: analysis of three large multicenter, double-blind, randomized controlled trials. Arthritis Rheum. 2009, 60(8):2490-8.

4. Khanna D, Lovell DJ, Giannini E, Clements PJ, Merkel PA, Seibold JR, Matucci-Cerinic M, Denton CP, Mayes MD, Steen VD, Varga J, Furst DE. Development of a provisional core set of response measures for clinical trials of systemic sclerosis. Ann Rheum Dis 2008;67:703–709.

5. Little, R, Yau.L. Intent-to-Treat Analysis for Longitudinal Studies with Drop-Outs. Biometrics, 1996, vol. 52, 1324-1333.

6. Ratitch, B, O'Kelly, M. Implementation of Pattern-Mixture Models Using Standard SAS/STAT Procedures. Proceedings of PharmaSUG2011 Conference – Paper SP04. http://www.pharmasug.org/proceedings/2011/SP/PharmaSUG-2011-SP04.pdf

7. Khanna D, Berrocal VJ, Giannini EH, Seibold JR, Merkel PA, Mayes MD, et al. The American College of Rheumatology Provisional Composite Response Index for Clinical Trials in Early Diffuse Cutaneous Systemic Sclerosis. Arthritis Rheumatol. 2016; 68(2):299-311.

8. Koch GG, Carr GJ, Amara IA, Stokes ME, Uryniac TJ, "Categorical Data Analysis" in Statistical methodology in the pharmaceutical science / edited by DA Berry, Marcel Dekker, 1990

9. EMA/CHMP/ICH/436221/2017. ICH E9 (R1) addendum on estimands and sensitivity analysis in clinical trials to the guideline on statistical principles for clinical trials, 30 August 2017)

10. SF-36v2® Advanced Scoring Guidelines:

How to submit data, what you will receive back, and scoring timeline Final. Revised: VL, KM, MW, AY, 11/13/2014

11. IMACS Form 04a: Instructions for the Health Assessment Questionnaire

12. PROMIS ADULT PROFILE INSTRUMENTS

9. Appendices

9.1 Appendix 1: Pattern-mixture modeling approach

The main idea of this method was introduced by Little and Yau (1996) [5] and later refined by Ratitch and O'Kelly (2011) [6]. The Ratitch and O'Kelly (2011) approach suggests using sequential regression and multiple imputation methodology to impute missing values after subject's discontinuation from the trial based on "as treated" model, using actual dose after drop-out if it is known, or based on some plausible assumptions if unknown. In most clinical trials, patients stop taking experimental medication after discontinuation. In this case, the "as treated" model for discontinued subjects would be based on the idea that patients are taking a zero dose of the experimental treatment. Hence, it might be reasonable to surmise that after the patient drops out from the study, patients from the experimental treatment arm will show the same future trend as those on the control treatment arm. Patients that discontinue from the control arm are assumed to evolve in the same way as control subjects that remain in the study.

Furthermore, this method will also address those patients who dropout and could come back for treatment later. As mentioned in the protocol, if the patient discontinues study drug prematurely during the main treatment phase (Week 0 – Week 52) he/she will be invited to come for limited assessments (mRSS, FVC, DL_{CO} and patient-reported outcomes [PROs]) at Week 12, Week 26, Week 39, and Week 52, depending on the timing of withdrawal.

This method uses PROC MI's methodology for imputation of monotone missing data patterns (available with the MONOTONE statement) to impute the outcome variable at consecutive visits in a sequential manner. The advantages of this approach are the following:

1. The method allows for modeling and imputation of either continuous or categorical response variables using methods appropriate for either continuous or categorical variables instead of approximations to the multivariate normal distribution.
2. Practically, availability of a CLASS statement with the MONOTONE statement in PROC MI also provides for an easier modeling of the categorical predictor variables and does not require additional programing with IML.

In control-based pattern imputation, the intent is to make no direct use of observed data from the experimental treatment arm for estimating the imputation model. Hence, PROC MI is called in such a way that it builds its imputation model only on data from the control arm, while it imputes missing data in both control and experimental treatment arms using a single control-based imputation model. This is achieved with a sequence of calls to PROC MI.

The general procedure for control-based pattern imputation is the following (where each call to PROC MI is intended to impute values at one time-point only):

(a) Impute the non-monotone data with the MCMC method and store the result of this partial imputation. In this study, the non-monotone data would represent those patients that discontinued study drug prematurely during the main treatment phase (Week 0- Week 52), but was able to comeback at a later visit for a limited assessment (e.g., Week 12, Week 26, Week 39, and/or Week 52). Classification variable (region) will be included in the MI step by using indicator variables).

Next, the patients that discontinued the study but were not able to come back at a later visit for a limited assessment will have their assessments for mRSS imputed in the following manner:

(b) With each call to PROC MI, only one time-point is imputed. This means to include only one variable corresponding to the time-point that needs to be imputed in the VAR statement (plus predictors), while respecting the order in which PROC MI would have done it in a single call (all time-points in chronological order).

(c) When imputing missing values for a specific time-point *t*, the input dataset should include all control subjects, but only those subjects from the experimental arm (riociguat) that have values at time-point *t* missing (that is, only those that need imputation at time-point *t*). Since subjects from the riociguat arm with non-missing values at time-point *t* are not included in the input dataset, they will not contribute to the estimation of an imputation model for time-point *t*. The imputation model will be estimated using control subjects only, while this call to PROC MI will impute missing data at time-point *t* for all subjects who need imputation at that time-point. Hence, subjects form the riociguat arm will be imputed based on the control subjects' (placebo) model. Note that treatment arm should not be included as an effect in this model.

(d) Repeat (c) for all other time-points sequentially. Subjects whose missing values were imputed in the last call to PROC MI will be included in the input dataset for the next call to PROC MI. Thus data for time-point *t*, filled in during the last call, will be used for predictor variables in the next call to PROC MI (for time point *t* + 1), which is consistent with the internal workings of a single call to PROC MI to impute all time-points automatically.

This procedure is illustrated below by the following example. To partially impute the non-monotone missing data (the intermediate missing visits for those subjects that dropout and return later for limited assessments) via the MCMC method, here is some example code:

```
proc mi data = DATAIN out = DATAIN_MONO nimpute =100  seed = 1234;
    var TRT region1 region2 SCORE_0 SCORE_1 SCORE_2 SCORE_3 SCORE_4;
    mcmc chain = multiple impute = monotone;
run;
```

where SCORE_0 , SCORE_1, SCORE_2, SCORE_3, SCORE_4 represent efficacy scores for mRSS at time-points 0 (baseline) and post-baseline visits 1, 2, 3 and 4, TRT represents treatment arm (0 = placebo, 1 =riociguat) and region1 and region2 are indicator variables for East Asia and North America (Europe and Australia/New Zealand being as a reference region).

For example, if a patient drops out at visit 1, and had an intermediate missing visit at visit 2, but then came back for a limited assessment at visit 3, this step would impute only the intermediate missing value of SCORE_2 for a single subject. This partial imputation will be stored in dataset DATAIN_MONO, which contains a variable _Imputation_ to distinguish between multiple copies of the original input data.

The next step is to create a monotone imputation method to impute the rest of the missing data (pertaining to those patients that dropped out at a particular visit, and were not able to have any subsequent limited assessments at future visits).

First, the missing data is imputed at the first time point that has some missing data (defined as time-point 1). In order to use the control-based imputation method properly, the data will be separated into two datasets: DATAIN_MONO_IMP1, containing all control (placebo) subjects and those subjects from the experimental arm (riociguat) that have values at time-point 1 missing; and DATAIN_MONO_REST1, containing the rest of the subjects from the experimental arm (those

with non-missing SCORE_1). Note that LASTVIS represents the last study visit attended by a subject (takes values 0, 1, 2 3 or 4)

```
data DATAIN_MONO_IMP1 DATAIN_MONO_REST1;
    set DATAIN_MONO;
    if treat = 1 and LASTVIS >= 1 then output DATAIN_MONO_REST1;
    else output DATAIN_MONO_IMP1;
run;   /*LASTVIS >= 1 represents the time-point 1 missing */
```

Next, PROC MI is called to impute missing data at time-point 1 based on the model estimated exclusively from control (placebo) subjects with non-missing values at time-point 1.

```
proc mi data = DATAIN_MONO_IMP1 out = DATAIN_REG_IMP1 nimpute = 1 seed = 234;
    by _Imputation_;
    var region1 region2 SCORE_0 SCORE_1;
    monotone reg(SCORE_1);
run;
```

The syntax monotone reg(SCORE_1) requests a default regression model for imputing SCORE_1 (that is, a model that includes all variables preceding SCORE_1 in the VAR statement as effects (SCORE_0 in this case)).

The next data step assembles back a dataset containing all subjects.

```
data DATAIN_IMP1;
    set DATAIN_MONO_REST1 DATAIN_REG_IMP1;
run;
```

Now imputing missing values for time-point 2 will commence. Dataset DATAIN_IMP1 will be used for input to the next PROC MI, but it will need to be separated into the following two datasets: 1. DATAIN_MONO_IMP2 (contains all control subjects and those subjects from the experimental arm that have values at time-point 2 missing; and DATAIN_MONO_REST2, containing the rest of the subjects from the experimental arm.

```
data DATAIN_MONO_IMP2 DATAIN_MONO_REST2;
    set DATAIN_IMP1;
    if treat = 1 and LASTVIS >= 2 then output DATAIN_MONO_REST2;
    else output DATAIN_MONO_IMP2;
run;
```

PROC MI will be called to impute missing data at time-point 2 based on the model estimated exclusively from control subjects with non-missing data at time-point 2. Similar as to time-point 1, a monotone reg(SCORE_2) statement below will result in using an imputation model for SCORE_2 with SCORE_0 and SCORE_1 as predictors.

```
proc sort data = DATAIN_MONO_IMP2; by _Imputation_; run;
proc mi data = DATAIN_MONO_IMP2 out = DATAIN_REG_IMP2 nimpute=1 seed=345;
    by _Imputation_;
    var region1 region2 SCORE_0 SCORE_1 SCORE_2;
    monotone reg(SCORE_2);
run;
```

As before, a dataset is assembled containing all subjects:

```
data DATAIN_IMP2;
    set DATAIN_MONO_REST2 DATAIN_REG_IMP2;
run;
```

The same procedure is then repeated until all time-points are imputed.

In the main treatment phase mRSS will be measured at baseline and 4 times post baseline (Weeks 12, 26, 39 and 52). As a result, dataset DATAIN_IMP4 would be the final dataset containing 100 imputed datasets where all missing values are filled and ready for analysis.

After the imputations are complete, the ANCOVA model described above in Method #3 will be performed at Week 52 for each completed data set.

Results of the ANCOVA analysis on 100 imputed datasets can now be combined to derive an overall result. This is done by applying PROC MIANALYZE.

```
proc sort data=DIFF_MI; by _Imputation_; run;
proc mianalyze parms(classvar=full)=DIFF_MI;
    class treat ;
    modeleffects treat;
    ods output ParameterEstimates=DIFF_MIAN;
run;
```

9.2 Appendix 2: Tipping point analysis

Multiple imputation will be done using SAS PROC MI under MNAR assumption using the following generic code, where regions are coded as dummy variables using Europe as respective reference group:

```
proc mi data=mRSS_data seed=1234 nimpute=100 out=mRSS_mi;
    by treat;
    MCMC niter=500 nbiter=200;
    var mRSSbase mRSSchange_week12 - mRSSchange_week52 region1  region2;
run;
```

All tipping-point analyses will be conducted after database lock. At this point delta method is utilized. Each imputed observation in the data set (here mRSS_mi) will be modified according to the required delta shift. Different delta patterns allow different sensitivity scenarios to be explored.

The following scenarios will be considered in this analysis:

Assume that at Week 52, there is no penalty in mRSS score in the placebo group. Then for those missing outcomes, we increase the mRSS score in increments of 2; that is 2, 4, 6, in the riociguat treatment arm until a p-value of 0.05 or higher is produced (beyond the tipping point). The actual penalty (to 1 decimal place) that gives a p-value above and closest to 0.05 will then be identified.

After each increment, the MMRM described in Method #1 and ANCOVA described in Method #3 will be performed at Week 52 for each completed data set. The results are then combined using SAS PROC MIANALYSE with the following generic code:.

```
proc mianalyze parms=diff_mi;
    class treat;
    modeleffects treat;
    ods output parameterestimates=out;
run;
```

For each scenario, the treatment difference at Week 52 will be given with a 95%-confidence interval and a p-value. In addition, the minimum and maximum of observed treatment differences over the imputed data sets will be presented.

Khanna D, *et al. Ann Rheum Dis* 2020; 79:618–625. doi: 10.1136/annrheumdis-2019-216823

9.3 Appendix 3: Calculation of confidence intervals using Mantel-Haenszel weighting scheme

The confidence intervals using Mantel-Haenszel weighting scheme will be calculated according to formulas given by Koch et al. (1990, p. 415ff.), i.e. to compute confidence interval for the difference in two binomial proportions obtained from a multicenter trial, we calculate a weighted difference and its associated variance using Mantel-Haenszel weighting scheme. For a multicenter study with 2x2 tables, the weighted difference is:

$$d = \frac{\sum w_h (p_{he} - p_{hc})}{\sum w_h},$$

where $$w_h = \frac{n_{he} * n_{hc}}{(n_{he} + n_{hc})},$$

and

p_{he} = success rate for experimental treatment in stratum *h*
p_{hc} = success rate for control treatment in stratum *h*
n_{he} = number of patient under experimental treatment in stratum *h*
n_{hc} = number of patient under control treatment in stratum *h.*

The variance of difference is:

$$var\ (d) = \frac{\sum w_h^2 \left[\frac{p_{hc} * (1 - p_{hc})}{n_{hc} - 1} + \frac{p_{he} * (1 - p_{he})}{n_{he} - 1}\right]}{(\sum w_h)^2}$$

Then a large sample approximation is used to compute the confidence interval:

$$CI = d \pm z_{\alpha/2} \sqrt{var(d)}$$

**Document Type:**	Statistical Analysis Plan
**Official Title:**	A Randomized, Double-Blind, Placebo-Controlled Phase II Study to Investigate the Efficacy and Safety of Riociguat in Patients With Diffuse Cutaneous Systemic Sclerosis (dcSSc)
**NCT Number:**	NCT02283762
**Document Date:**	12 Jan 2018

Study Protocol Title (incl. version no. and date):

A Randomized, Double-Blind, Placebo-Controlled Phase II Study to Investigate the Efficacy and Safety of Riociguat in Patients With Diffuse Cutaneous Systemic Sclerosis (dcSSc),

Version 5.0, 02 MAY 2017

SAP (incl. Version no. and date): Version 4.0, 22 DEC 2017

**Analysis purpose:**	Tipping point analyses for secondary efficacy endpoints		
**Clinical study phase:**	IIb	**Date:**	12 Jan 2018
**Study No.:**	16277	**Version:**	1.0
**Author:**	PPD		

Confidential

The information provided in this document is strictly confidential and is intended solely for the guidance of the clinical investigation. Reproduction or disclosure of this document, whether in part or in full, to parties not associated with the clinical investigation or its use for any other purpose without the prior written consent of the sponsor is not permitted.

Throughout this document, symbols indicating proprietary names (®, TM) are not displayed unless quotations from other documents are given. Hence, the appearance of product names without these symbols does not imply that these names are not protected.

This “Additional Statistical Analysis Plan” is produced on a word-processing system and bears no signatures.

Table of contents

1. Analyses to address local regulatory requirements 3
1.1 Tipping point analysis for key secondary efficacy endpoint, i.e. CRISS 4
1.2 Tipping point analysis for further secondary efficacy endpoints 5
1.2.1 Scenarios used in analysis of secondary efficacy endpoints 6

Table of text tables

Table 1–1 6

List of abbreviations

CRISS	Combined Response Index for Systemic Sclerosis
FVC	Forced vital capacity
HAQ-DI	Health Assessment Questionnaire disability index
MI	Multiple imputation
MMRM	Mixed model repeated measures
MNAR	Missing not at random
SAP	Statistical analysis plan
SAS	Statistical analysis system

1. Analyses to address local regulatory requirements

This supplementary SAP describes additional sensitivity analyses, i.e. tipping point analyses for secondary efficacy variables. Analyses will be performed only if primary analyses of these variables are statistically significant (see Statistical Analysis Plan Version 4.0, Section 6.2.2). All tipping-point analyses described in this document will be conducted after database lock.

Tipping point sensitivity analyses that vary assumptions about the missing outcomes on the two treatment arms regarding the secondary efficacy endpoint, i.e. CRISS and its components (change from baseline to Week 52 in HAQ-DI, patient's global assessment, physician's global assessment and FVC% predicted), will be conducted.

The estimand of interest is the de facto or treatment policy estimand but with the value at Week 52 (if missing) estimated using multiple imputation with various penalties. For CRISS that is difference of proportions of improvers at Week 52. For HAQ-DI, patient's global assessment, physician's global assessment and FVC % predicted that is mean difference in change from baseline to Week 52 for each endpoint between riociguat and placebo.

1.1 Tipping point analysis for key secondary efficacy endpoint, i.e. CRISS

Primary analysis described in Statistical Analysis Plan Version 4.0 assumes that there is complete data in step 2 of derivation of CRISS. That is when all five components of the CRISS are fully recorded at Week 52. If the patient has three or more missing components of the CRISS at this visit, a probability score of improving will be set equal to 0.0. If a patient has 1 or 2 missing components, then multiple imputation will be used for CRISS score. The following generic SAS-code will be used, where regions are coded as dummy variables using Europe as respective reference group:

```
proc mi data=criss_data seed=9876 nimpute=100 minimum=0 maximum=1 out=out_mi;
    by treatmgr;
    var  aval region1 region2;
    MCMC niter=500 nbiter=200;
run;
```

Thus the imputed values will be generated by a multivariate normal distribution. Therefore, a delta shift is reasonable to create the scenarios for the tipping point analysis. The distribution, out of which the imputed values for the missing CRISS in the riociguat treatment arm will be drawn, will be varied by a delta shift in increments of -0.1; that is -0.1, -0.2, -0.3. There is no penalty in CRISS score in the placebo group.

As the CRISS ranges from zero to one, also the imputed values will be restricted to that range. The first generated value between zero and one will be used for imputation. In order for this to apply in all scenarios, the delta shift has to be done before the imputation. For each scenario, the observations in the riociguat treatment arm will be shifted. Then the multiple imputation will be done. Thereafter the *observed* values in the riociguat treatment arm need to be shifted back (but not the imputed values).

CRISS scores (predicted probability of improving) are classified as improved (scores ≥ 0.60) and not improved (scores < 0.60), and the Mantel-Haenszel method described in Section 6.2.2 and

Appendix 3 (SAP version 4.0) will be performed for each completed data set. The results are then combined using SAS PROC MIANALYSE with the following generic code:

```
proc mianalyze data=results_mi(where=(_stratum="POOLED"));
   modeleffects  _estimate;
   stderr _std_est;
  ods output parameterestimates=out_mi;
run;
```

The procedure (increasing CRISS score, classifying CRISS scores as improved and notimproved, producing and combining Mantel-Haesnzel statistics) will be repeated until a p-value of 0.05 or higher is produced (beyond the tipping point). The actual penalty (to 2 decimal places) that gives a p-value above and closest to 0.05 will then be identified.

For each scenario, the estimate of treatment difference will be given with a 95%-confidence interval and a p-value. In addition, the minimum and maximum of observed treatment differences over the imputed data sets will be presented.

1.2 Tipping point analysis for further secondary efficacy endpoints

Similar method as described in SAP v4.0 Method #6 will be performed for exploring missing data assumption in components of CRISS (change from baseline to Week 52 in HAQ-DI, patient's global assessment, physician's global assessment and FVC% predicted).

Multiple imputation will be done using SAS PROC MI under MNAR assumption using the following generic code, where regions are coded as dummy variables using Europe as respective reference group separately for every endpoint:

```
proc mi data=endpoint_data seed=5678 nimpute=100 out=out_mi;
    by treat;
    MCMC niter=500 nbiter=200;
    var endp_base endp_change_week12 - endp_change_week52 region1 region2;
run;
```

All tipping-point analyses will be conducted after database lock. At this point delta method is utilized. Each imputed observation in the data set (here out_mi) will be modified according to the required delta shift for each parameter. Different delta patterns allow different sensitivity scenarios to be explored. See Section 1.2.1 below.

After each increment, the MMRM described in Method #1 (Section 6.2.1 SAP version 4.0) will be performed at week 52 for each completed data set of each secondary efficacy parameter. The results are then combined using SAS PROC MIANALYSE with the following generic code:

```
proc mianalyze parms=diff_mi;
    class treatmgr;
    modeleffects treatmgr;
    ods output parameterestimates=out_mi;
run;
```

For each scenario of each parameter, the estimate of treatment difference will be given with a 95%-confidence interval and a p-value. In addition, the minimum and maximum of observed treatment differences over the imputed data sets will be presented.

1.2.1 Scenarios used in analysis of secondary efficacy endpoints

The following scenarios will be considered in this analysis:

Assume that at Week 52 there is no penalty in the placebo group. Then for those missing outcomes, the values in the riociguat treatment arm will be increased/decreased until a p-value of 0.05 in MMRM Method #1 or higher is produced (beyond the tipping point) according to Table 1–1. The actual penalty (with an accuracy of 1 decimal place more than the actual value) that gives a p-value above and closest to 0.05 will then be identified.

Table 1–1

Variable	Penalty in Placebo	Penalty in Riociguat	Increment
HAQ-DI	0	0.2, 0.4, 0.6,….	0.2
Patient's global assessment	0	1, 2, 3, ….	1
Physician's global assessment	0	1, 2, 3, ….	1
FVC%, predicted	0	-2, -4, -6	-2
